# Revision of the Afrotropical Oberthuerellinae (Cynipoidea, Liopteridae)

**DOI:** 10.3897/zookeys.202.2136

**Published:** 2012-06-15

**Authors:** Matthew L. Buffington, Simon van Noort

**Affiliations:** 1 Systematic Entomology Lab, USDA, c/o Smithsonian NMNH, 10th & Constitution Ave NW, Washington DC 20013; 2 Natural History Department, Iziko South African Museum, PO Box 61, Cape Town, 8000, South Africa; 3 Department of Zoology, University of Cape Town, Private Bag, Rondebosch, 7701

**Keywords:** Africa, Afrotropical region, Cynipoidea, Hymenoptera, identification key, Liopteridae, Madagascar, systematics

## Abstract

The Afrotropical Oberthuerellinae are revised, and new dichotomous and multi-entry keys to the species of *Oberthuerella*, *Tessmannella*, and *Xenocynips* are provided. All previously described species in these genera are redescribed; descriptions are augmented by color images of the holotype for each species. The following 11 species are described as new: *Oberthuerella cyclopia* Buffington & van Noort; *Oberthuerella eschara* Buffington & van Noort; *Oberthuerella kibalensis* van Noort & Buffington; *Oberthuerella pardolatus* Buffington & van Noort; *Oberthuerella sharkeyi* Buffington & van Noort; *Oberthuerella simba* Buffington & van Noort; *Tessmannella copelandi* Buffington & van Noort; *Tessmannella kiplingi* Buffington & van Noort; *Tessmannella roberti* Buffington & van Noort; *Xenocynips rhothion* Buffington & van Noort; and *Xenocynips ronquisti* Buffington & van Noort. We provide identification keys to the genera and species occurring in the Afrotropical region. Online dichotomous and interactive Lucid keys to genera and species are available at http://www.waspweb.org/Cynipoidea/Keys/index.htm

## Introduction

The Afrotropical Cynipoidea are taxonomically and biologically poorly known, a situation typical for the majority of wasp taxa from this region. The lack of knowledge on cynipoid systematics is exemplified by the recent revision of the Pycnostigminae (Figitidae) (Buffington and van Noort 2007), where species richness of the Afrotropical members of this subfamily was elevated by 86%. As a consequence of the under-documentation of the region's diversity, the process of unraveling the biology of the Afrotropical cynipoid wasps is also in its infancy. Some recent headway has been made with a recent biological study of *Rhoophilus loewi* (van Noort et al. 2006) and the discovery of two true indigenous gall formers, *Phanacis neserorum* (Melika & Prinsloo, 2007), and *Qwaqwaia scolopiae* Liljeblad, Nieves-Aldrey and Melika, the latter meriting the description of a new genus and establishment of a new tribe (Liljeblad et al. 2011). However, the biology of the Liopteridae is even more poorly known, with only a few published rearing records: two species of *Kiefferiella* Ashmead were reared from buprestids (*Acmaeodera pulchella* (Herbst)) in infested logs (Weld 1956); a *Kieferiella* species and a *Paramablynotus* Cameron species were reared from trees in the family Fabaceae, *Prosopis glandulosa* Torr. and *Dalberghia fusca* Pierre, respectively (Ronquist, 1995). These associations are all for representatives of the subfamily Mayrellinae with no records available for the Liopterinae or Oberthuerellinae.

Ronquist (1995) regarded the Afrotropical Liopteridae to be poorly sampled, which was confirmed by a recent revision of *Paramblynotus* where species richness for the region was elevated from three previously described species to 26 (Liu et al. 2007). Prior to the revision of *Paramblynotus*, only 19 species of Liopteridae were known from the Afrotropical Region. We elevate the current total to 53 species with the description of 11 new species in this paper.

The original description of *Oberthuerella lenticularis* Saussure, the type species of *Oberthuerella* Saussure, was based solely on an illustration and a name (Saussure, 1890), and no holotype was ever designated. Kieffer (1904) provided a more robust description of *Oberthuerella lenticularis*, and Weld (1952) suggested a specimen in MHNG (Museum d'Histoire Naturelle, Geneva, Switzerland) might be the type; Quinlan (1979), however, provided convincing evidence against this latter specimen being the type (based on collection date), and to this date, no holotype specimen has been discovered for *Oberthuerella lenticularis*. Hedicke and Kerrich (1940) were the first to propose the subfamily group-name Oberthuerellinae, and included the subfamily in an updated key to the liopterids. Quinlan (1979) provided further diagnosis of the subfamily, and Ronquist (1995) provided not only eight synapomorphies supporting the monophyly of the subfamily, but also recovered the subfamily as sister-group to Liopterinae. Benoit (1955) added greatly to our knowledge of *Oberthuerella* by describing the majority of species we recognize today. *Tessmannella* Hedicke (1912) remained monotypic until Benoit (1955) added two species, and Quinlan (1979) added one additional species. *Xenocynips* Kieffer (1910) was redescribed by Quinlan (1979) and Ronquist (1995), but up to this writing, no new species have been proposed.

Oberthuerellines are rarely collected cynipoids. Intensive collections in South Africa, Congo, Central African Republic and Kenya have yielded precious few of these uncommon insects. Many of the specimens representing new taxa presented here are based on these freshly collected field samples, underscoring that further fieldwork would undoubtedly result in additional new species. We are hopeful that the descriptions, diagnoses and keys presented herein and on www.waspweb.org will provide the foundation for future research on these enigmatic wasps.

## Materials and methods

Freshly collected specimens were point-mounted on black or white, acid-free cards for examination (using a Wild M-5 stereomicroscope with incandescent and fluorescent light sources), photography and long-term preservation. Images were acquired using the EntoVision multiple-focus imaging system. This system comprises a Leica M16 microscope with a JVC KY-75U 3-CCD digital video camera attached that fed image data to a notebook computer. The program Cartograph 5.6.0 was then used to merge an image series (representing typically 10–15 focal planes) into a single in-focus image. Diffused lighting was achieved using techniques summarized in Buffington et al. (2005), Kerr et al. (2009) and Buffington and Gates (2009). Morphological terminology follows that of Fontal-Cazalla et al. (2002) and Nordlander and Ronquist (1989); cuticular surface terminology follows Harris (1979). Descriptions and maps were generated using vSyslab (Johnson 2008). Identification keys were produced in 3 formats to facilitate accessibility by a range of end-users (Penev et al. 2009): 1. Traditional dichotomous keys that include incorporation of colour annotated images above each couplet facilitating the recognition of diagnostic characters. These are published below and made available as static keys on www.waspweb.org; 2. Online interactive Lucid Phoenix keys were produced and are hosted on www.waspweb.org; 3. Online interactive Lucid matrix keys were produced using output from the vSyslab and hosted on www.waspweb.org. Although Lucid Phoenix keys are interactive keys they are still dichotomous and a choice needs to be made at each key couplet to continue. Lucid matrix keys, on the other hand, use a different approach where relevant states from multiple character features can be selected independently until identification is achieved (www.lucidcentral.org).

Morphological terms used in this revision were matched to the Hymenoptera Anatomy Ontology (HAO, Yoder et al. 2010) (Appendix I). Identifiers (URIs) in the format http://purl.obolibrary.org/obo/HAO_XXXXXXX represent anatomical concepts in HAO version http://purl.obolibrary.org/obo/hao/2011-05-18/hao.owl. They are provided to enable readers to confirm their understanding of the anatomical structures being referenced. To find out more about a given structure, including, images, references, and other metadata, use the identifier as a web-link, or use the HAO:XXXXXXX (note colon replaces underscore) as a search term at http://glossary.hymao.org. All images presented in this paper are freely available through http://morphbank.net and http://www.waspweb.org using the link to individual collections found at the beginning of each species description.

### List of depositories

BMNH The Natural History Museum, London: Curator David Notton

DEI Senckenberg Deutches Entomologisches Institut, Eberswalde, Germany. Curator: Andreas Taeger

MHNG Museum d'Histoire Naturelle, Geneva, Switzerland. Curator: John Hollier

MNHN Natural History Museum, Paris, France. Curator: Claire Villemant

MRAC Africa Museum, Tervuren, Belgium. Curator: Eliane de Conick

SAMC Iziko South African Museum, Cape Town, South Africa. Curator: Simon van Noort

SANC South African National Collection of Insects, Pretoria, South Africa. Curator: Janine Kelly.

USNM National Museum of Natural History, Washington DC, USA. Curator: Matthew Buffington.

### Key to Afrotropical liopterid genera both sexes (modified from Ronquist 1995)

(Available online at http://www.waspweb.org/Cynipoidea/Keys/index.htm)

**Table:** 

	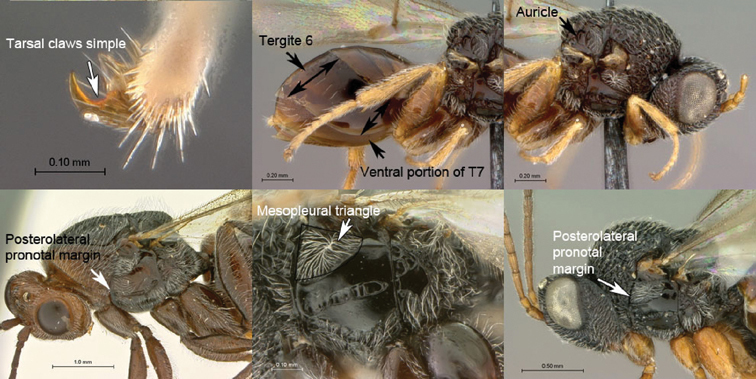
1A	Claws simple, without basal lobes. Metasomal tergite 6 of females longer dorsally than ventrally in lateral view, posteroventral margin sinuate, strongly curving forward in lateral view, and not covering ventral portion of T7. Scutellum with auricula (laterally with semilunar, slightly impressed area set off by distinct carina). Posterolateral pronotal margin not incised, mesopleural triangle not deeply impressed anteriorly (Mayrellinae)	*Paramblynotus*
	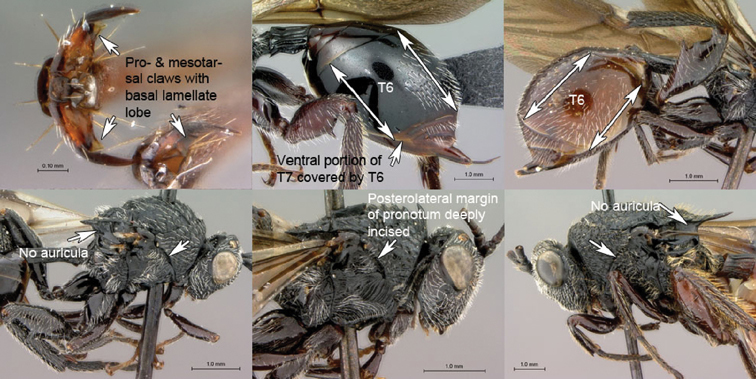
1B	Pro- and mesotarsal claws with basal, lamellate lobe. Metasomal tergite 6 of females as long ventrally as dorsally in lateral view; posterior margin straight to gently curved forward in lateral view and covering ventral portion of T7. Scutellum laterally without auricula. Posterolateral pronotal margin distinctly inflected in front of mesopleural triangle, the latter deeply impressed anteriorly (Oberthuerellinae)	2
	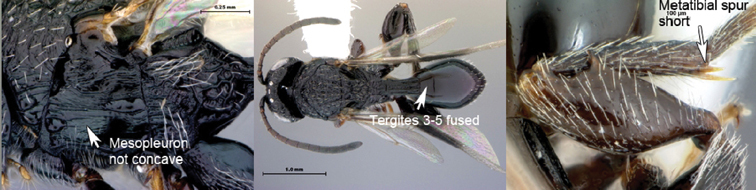
2A	Mesopleural surface not concave; mesopleural impression present; mesopleuron at least partly horizontally strigate ventrally. Anterior metatibial spur shorter than posterior metatibial spur. Metasomal terga 3–5 fused, inter-tergal sutures at least partly invisible	*Xenocynips*
	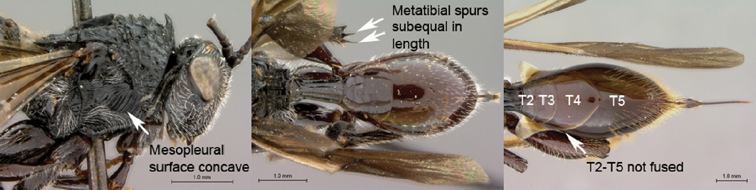
2B	Mesopleural surface distinctly concave, the concavity forming oblique, shallow femoral groove; mesopleural impression absent; mesopleuron not horizontally strigate ventrally. Metatibial spurs subequal in length, elongate. Metasomal terga 3–5 not fused, inter-tergal sutures distinct	3
	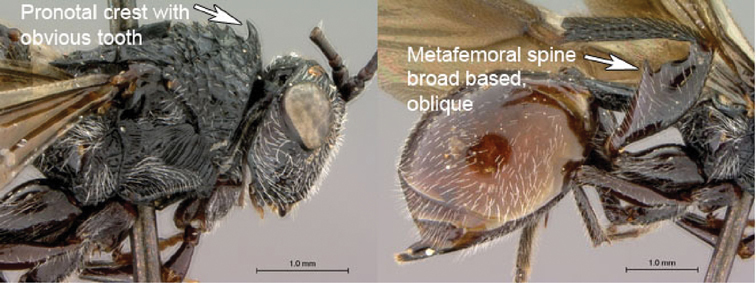
3A	Pronotal crest produced into conspicuous tooth-like process. Ventral margin of mesopleural impression visible as well-defined ventral margin of obliquely costate area of mesopleuron. Metanotal trough absent. Metafemoral spine triangular, broad-based, oblique	*Tessmannella*
	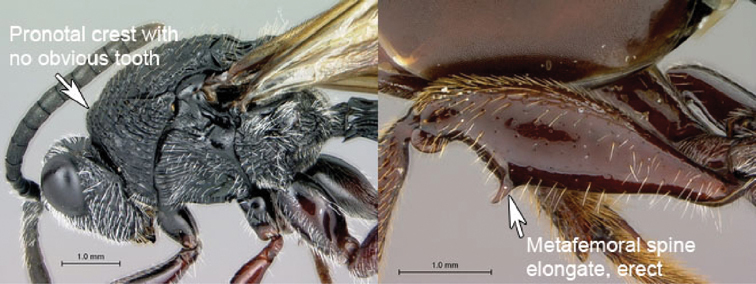
3B	Pronotal crest not produced into conspicuous tooth-like process, but occasionally produced into small, triangular process. Ventral margin of mesopleural impression not marked. Metanotal trough clearly indicated. Metafemoral spine elongate, narrow-based, erect	*Oberthuerella*

## Taxonomy

### Subfamily Oberthuerellinae

#### Oberthuerella

Saussure

http://www.waspweb.org/Cynipoidea/Liopteridae/Oberthuerellinae/Oberthuerella/index.htm

http://species-id.net/wiki/Oberthuerella

Oberthuerella Saussure, 1890: plate 20, fig. 20. Type species: *Oberthuerella lenticularis* Saussure, by monotypy.

##### Diagnosis.

*Oberthuerella* can be readily distinguished from *Xenocynips* by having distinct metasomal terga (tergites 3–5) with the inter-tergal sutures not fused. Mesopleuron is also distinctly concave, the concavity forming an oblique, shallow femoral groove; the mesopleural impression is absent and the ventral part of the mesopleuron is without horizontal, linear sculpture; the metatibial spurs are subequal in length, elongate. The lack of a pronotal crest produced into a conspicuous tooth-like process easily distinguishes *Oberthuerella* from *Tessmannella*.

##### Distribution.

Cameroon, Congo, Democratic Republic of Congo, Equatorial Guinea, Gabon, Ivory Coast, Kenya, Liberia, Madagascar, Malawi, South Africa, Tanzania, Uganda, Zambia, Zimbabwe.

##### Biology.

Unknown.

##### Included species.

*Oberthuerella abscinda* Quinlan, 1979: 111

*Oberthuerella aureopilosa* Benoit, 1955: 290

*Oberthuerella breviscutellaris* Benoit, 1955: 286

*Oberthuerella crassicornis* Benoit, 1955: 289

*Oberthuerella compressa* Benoit, 1955: 292. Synonymy by Quinlan (1979).

*Oberthuerella cyclopia* Buffington & van Noort, sp. n.

*Oberthuerella eschara* Buffington & van Noort, sp. n.

*Oberthuerella kibalensis* van Noort & Buffington, sp. n.

*Oberthuerella lenticularis* Saussure, 1890: plate 20; fig. 20

*Oberthuerella longicaudata* Benoit, 1955: 291

*Oberthuerella longispinosa* Benoit, 1955: 290

*Oberthuerella nigra* Kieffer, 1910a: 110. Holotype male previously in ZMHB, now missing (Ronquist, 1995). Not examined.

*Oberthuerella nigrescens* Benoit, 1955 : 288

*Oberthuerella pardolatus* Buffington & van Noort, sp. n.

*Oberthuerella sharkeyi* Buffington & van Noort, sp. n.

*Oberthuerella simba* Buffington & van Noort, sp. n.

*Oberthuerella tibialis* Kieffer, 1904: 107

*Oberthuerella transiens* (Benoit); new combination by Ronquist (1995).

*Tessmannella transiens* Benoit, 1955: 283.

*Oberthuerella triformis* Quinlan, 1979: 115

##### Key to *Oberthuerella* species (both sexes; modified from Quinlan 1979)

### 

(Available online at http://www.waspweb.org/Cynipoidea/Keys/index.htm)

**Table:** 

	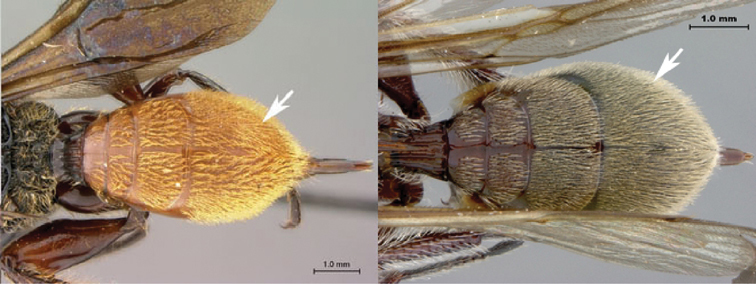
1A	Metasoma with terga 3–5 conspicuously setose	2
	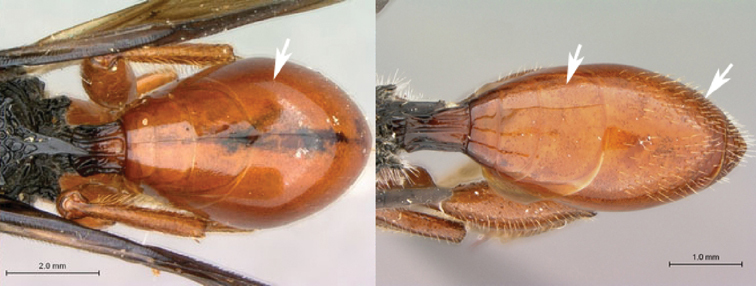
1B	Metasoma with terga 3–5 glabrous or with sparse setae on tergite 5	3
	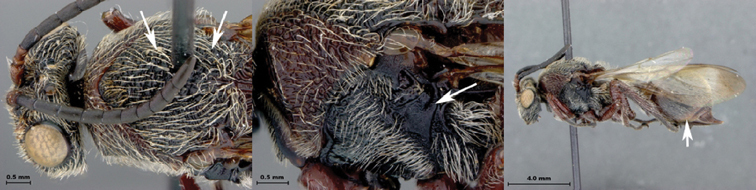
2A	Mesoscutum covered with long, dense, pale setae; speculum shagreened; metasoma reddish-brown with white setae	*Oberthuerella simba* sp. n.
	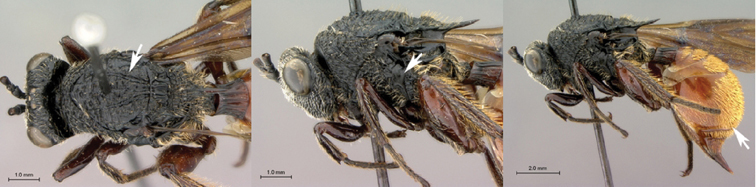
2B	Mesoscutum covered with long, sparse, dark setae; speculum smooth; metasoma burnt-orange with orange setae	*Oberthuerella aureopilosa*
	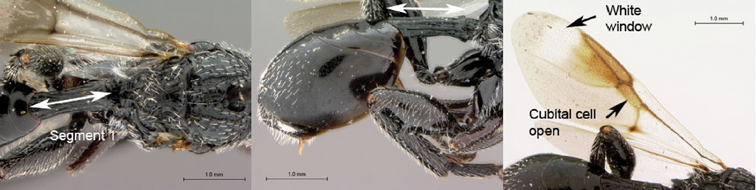
3A	Petiole slightly more than 3× longer than as long as wide; forewing with a non-infuscate, distal subquadrate window; areolet absent, cubital cell open along ventral margin	*Oberthuerella transiens*
	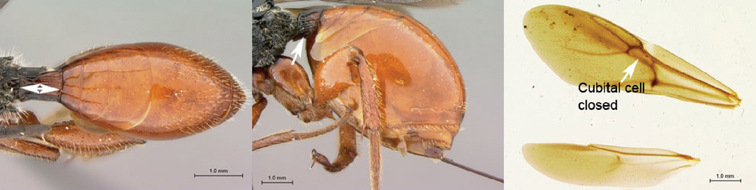
3B	Petiole not more than 2× longer than wide. Forewing more evenly infuscate; areolet present or absent, cubital cell open or closed along ventral margin	4
	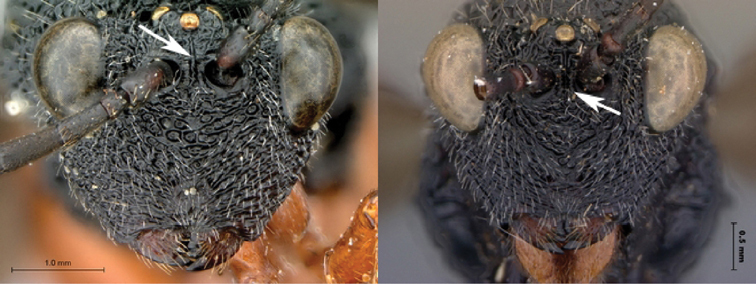
4A	Median keel of face very short, not extending ventrally of horizontal line drawn between ventral margins of toruli	5
	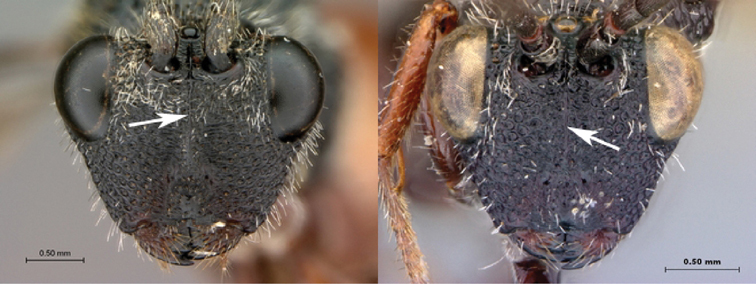
4B	Median keel long, extending ventrally of horizontal line drawn between ventral margins of toruli	6
	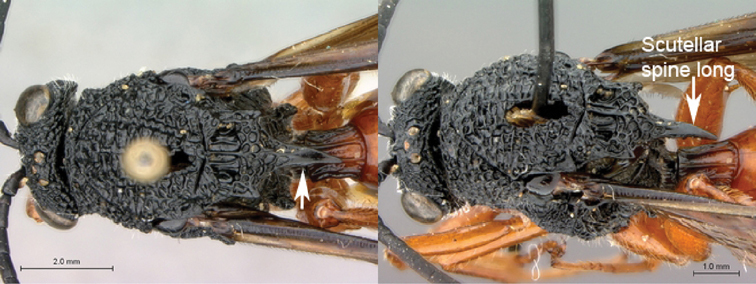
5A	Scutellar spine as long as petiole in dorsal view	*Oberthuerella lenticularis*
	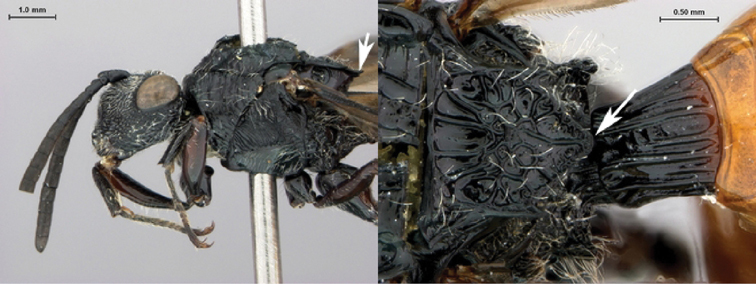
5B	Scutellar spine distinctly shorter than petiole in dorsal view	*Oberthuerella triformis*
	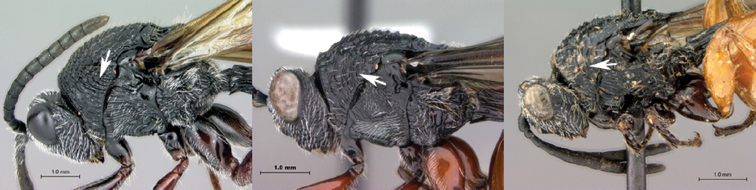
6A	Pronotum dorsally and laterally distinctly striate, wave-like, with some irregular fovea	7
	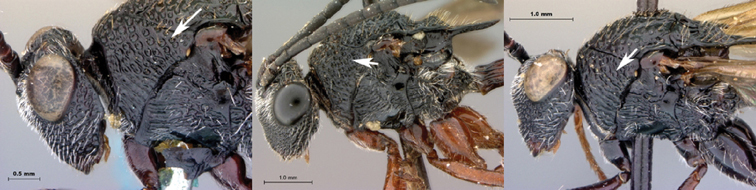
6B	Pronotum dorsally and laterally foveate, with only slight striations present	9
	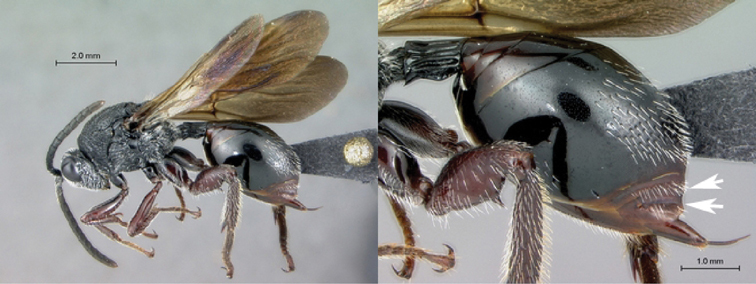
7A	Metasoma all black to dark brownish/red in color; peg-like setae absent on posterior margin of metasomal terga 6, 7	*Oberthuerella kibalensis* sp. n.
	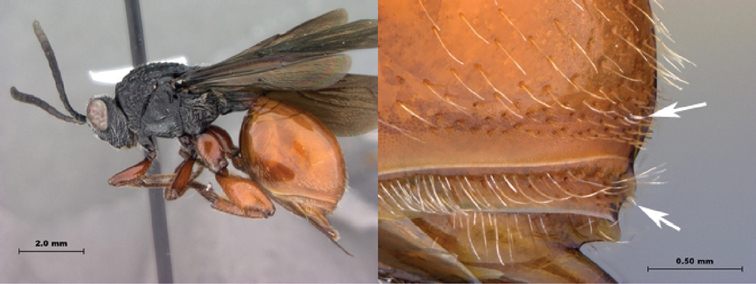
7B	Metasoma burnt orange in color; peg-like setae (arrowed) present on posterior margin of metasomal terga 6, 7	8
	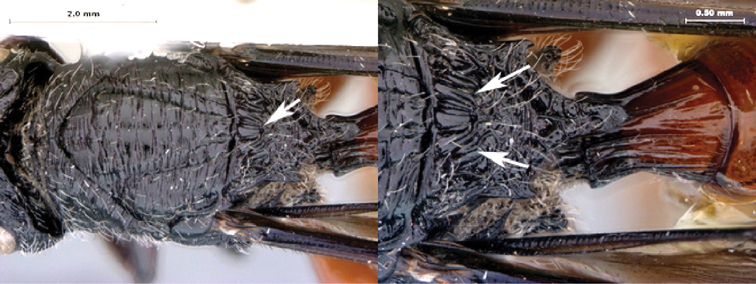
8A	Median scutellar fovea absent, two distinct scutellar foveae subdivided into 10 subfoveae	*Oberthuerella breviscutellaris*
	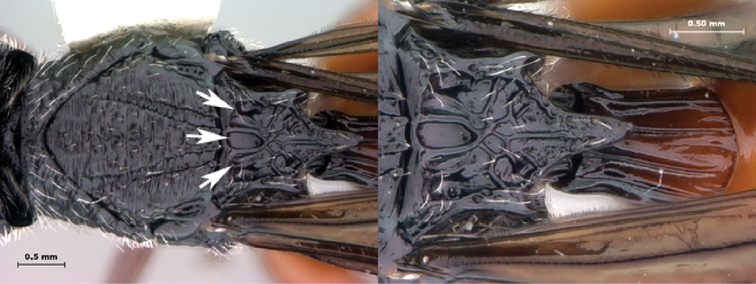
8B	Median scutellar fovea present, with two lateral foveae subdivided into 2 subfovea	*Oberthuerella sharkeyi* sp. n.
	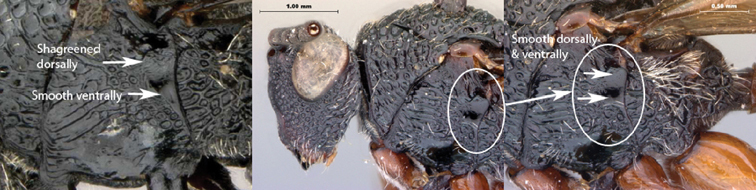
9A	Speculum smooth ventrally	10
	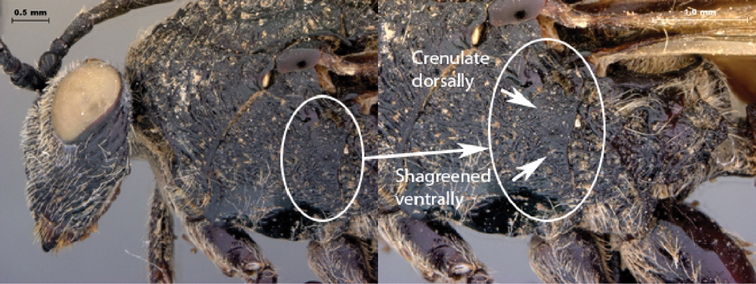
9B	Speculum gently shagreened ventrally	*Oberthuerella cyclopia* sp. n.
	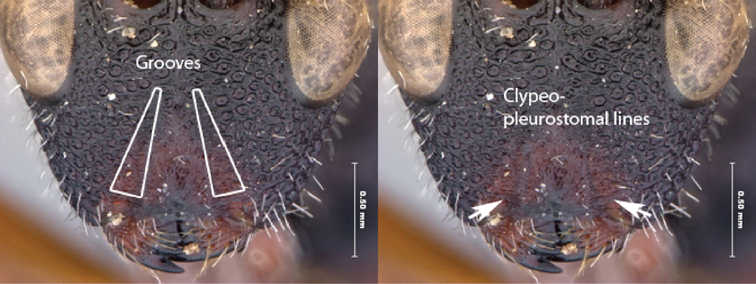
10A	Dorso-ventral grooves of face present; clypeo-pleurostomal line present	*Oberthuerella eschara* sp. n.
	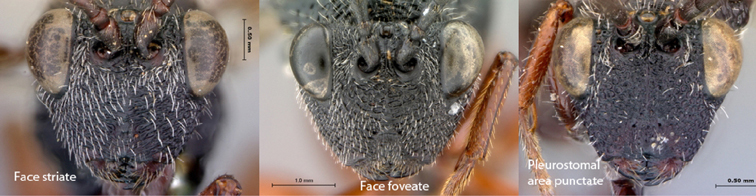
10B	Middle to lower face horizontally striate or foveate, dorso-ventral grooves of face absent; clypeo-pleurostomal line absent.	11
	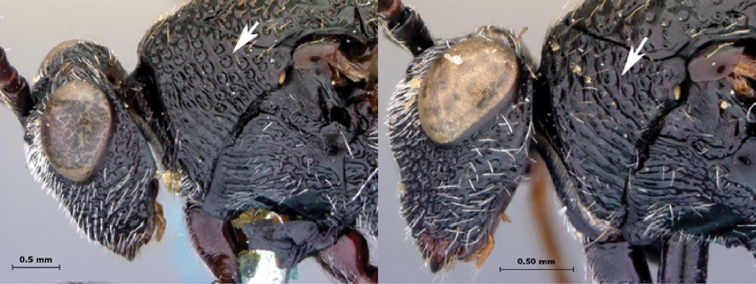
11A	Pronotum and mesoscutum with smooth to shagreened space between individual fovea	15
	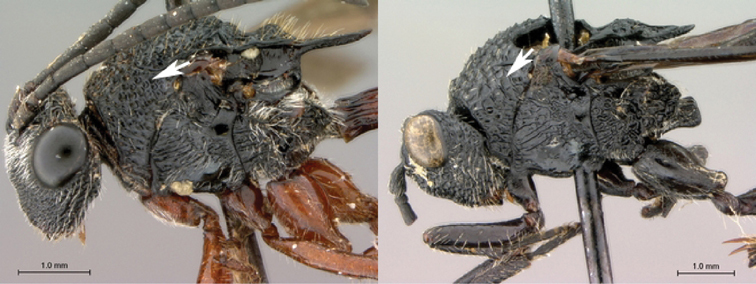
11B	Pronotum and mesoscutum with fovea abutting each other, resulting in a craggy appearance	12
	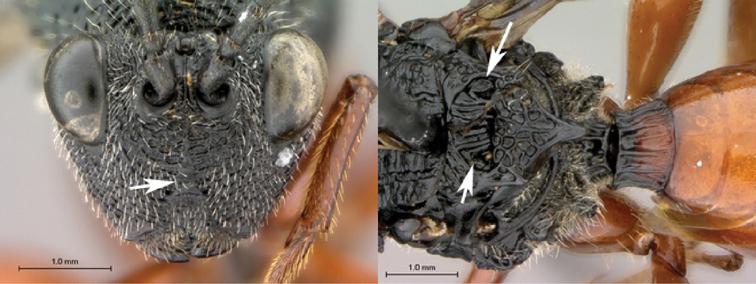
12A	Facial triangle present along midline of face, terminating at dorsal margin of clypeus; Scutellar foveae divided into 3 subfoveae	*Oberthuerella longicaudata*
	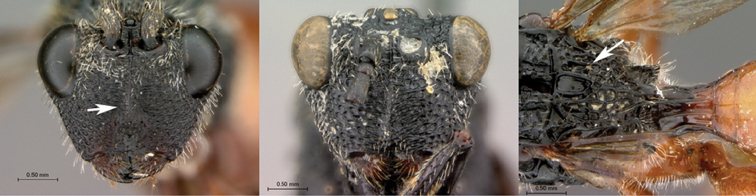
12B	Facial triangle absent, instead, area is deeply costate-foveate, with or without thin longitudinal keel; scutellar foveae subdivided into 5–6 subfovea	13
	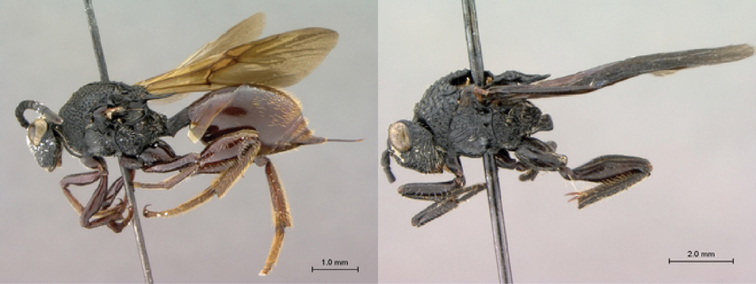
13A	Head, mesosoma, and legs black to very dark brown	16
	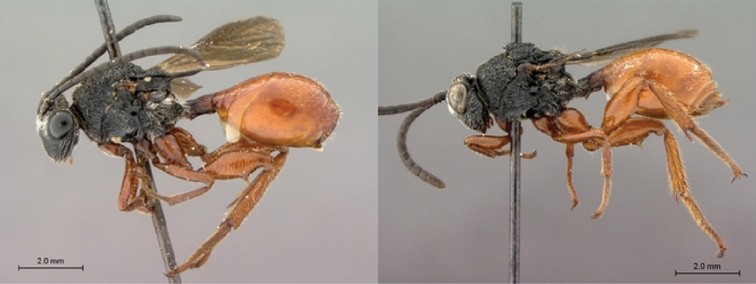
13B	Legs and metasoma orange/yellow	14
	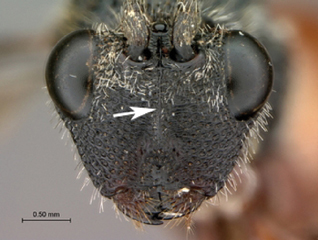
14A	Longitudinal facial keel narrow, extending ventrally to clypeu	*Oberthuerella longispinosa*
	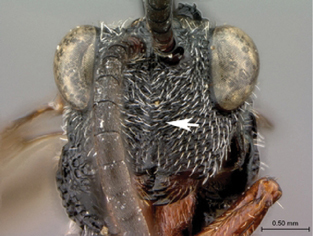
14B	Longitudinal facial keel narrow dorsally, becoming irregularly widened ventrally, ending dorsally of clypeus	*Oberthuerella crassicornis*
	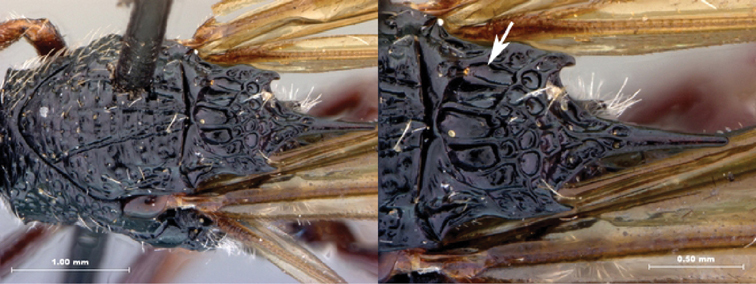
15A	Anterior base of scutellum with 5 fovea; admedial lines indistinct to absent; mesoscutal surface dominated by shallow fovea, with more distinct transverse striations	*Oberthuerella pardolatus* sp. n.
	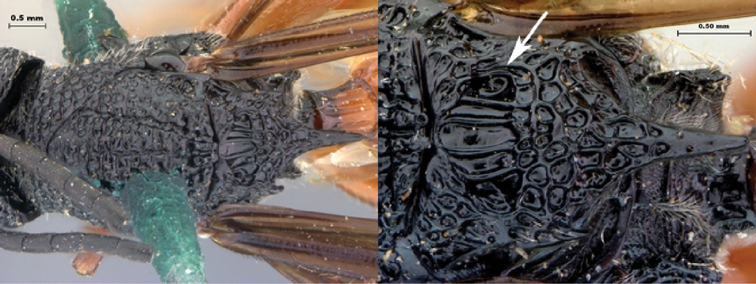
15B	Anterior base of scutellum with 7 fovea; admedial lines present, distinct; mesoscutal surface with distinct fovea	*Oberthuerella tibialis*
	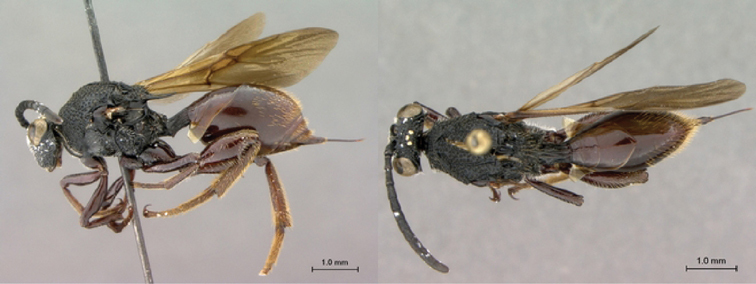
16A	Petiole black, remaining metasoma entirely dark brown or black	*Oberthuerella nigrescens*
	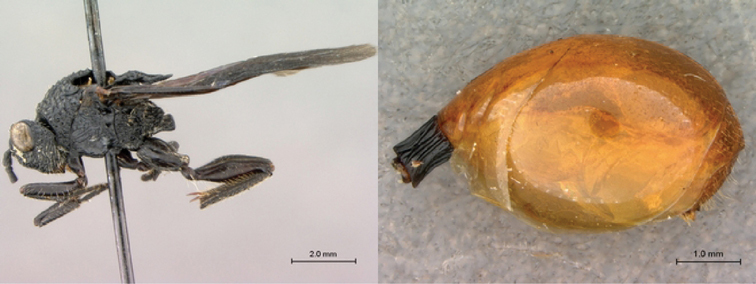
16B	Petiole black, remaining metasoma entirely orange or yellow	*Oberthuerella abscinda*

#### Oberthuerella abscinda

Quinlan

urn:lsid:biosci.ohio-state.edu:osuc_concepts:181552

Morphbank accession: 704704–704714

http://www.waspweb.org/Cynipoidea/Liopteridae/Oberthuerellinae/Oberthuerella/Oberthuerella_abscinda.htm

http://species-id.net/wiki/Oberthuerella_abscinda

Figures 1 –2

Oberthuerella abscinda Quinlan, 1979: 111

##### Description.

Coloration of head and mesosoma black to dark brown, metasoma yellow-orange; legs reddish brown. Sculpture on vertex, lateral surface of pronotum and mesoscutum present, deeply foveate laterally on head; pronotum, mesoscutum striate-foveate.

*Head*. Broadly triangular, wider than high, in anterior view. Pubescence on head present, dense setae covering head. Sculpture along lateral margin of occiput with one costula. Gena (measured from compound eye to posterolateral margin of head) long, ratio of length of gena length/to length of compound eye in dorsal view > 0.3, in dorsal view. Sculpture of gena present, with distinct fovea. Lateral margin of occiput defined by distinctly angled, raised, sharp carina. Occiput (except extreme lateral margin) with some weak subvertical, irregular strigae. Ocelli large, ratio of maximum diameter of a lateral ocellus > 0.4. Anterior ocellus close to posterior ocelli, posterior margin of anterior ocellus behind or subcontiguous with a transverse line running through anterior margins of posterior ocelli. Relative position of toruli close to ocelli, ratio of vertical distance between inner margin of torulus and ventral margin of clypeus to vertical distance between anterior ocellus and torulus < 2.0. Median keel of face present, extending to posterior margin of clypeus. Vertical carina adjacent to ventral margin of torulus absent. Facial sculpture present, punctate-rugose, transversely striate; striations meeting at medial keel. Facial impression absent, face flat. Antennal scrobe absent. Anterior tentorial pits large. Vertical delineations on lower face absent. Ventral clypeal margin laterally, close to anterior mandibular articulation, distinctly angled. Ventral clypeal margin medially emarginate. Clypeus foveate-punctate. Malar space adjacent to anterior articulation of mandible evenly rounded, foveate. Malar sulcus absent. Compound eye close to posterior ocellus, ratio of distance between compound eye and posterior mandibular articulation to distance between posterior ocellus and compound eye > 1.2. Compound eye, in dorsal view, distinctly protruding from the surface of the head, particularly laterally; glabrous. Orbital furrows absent. Lateral frontal carina of face present. Dorsal aspect of vertex variously strigate. Posterior aspect of vertex foveate. Hair punctures on lateral aspect of vertex absent. Posterior surface of head almost flat, not deeply impressed.

*Antenna*.Articulation between flagellomeres connate with flagellomeres broadly joined. Female antenna composed of 11 flagellomeres. Male antenna composed of 12 flagellomeres. Female F1 shorter than F2, black. Flagellomeres of female antenna cylindrical, distinctly widened towards apex, non-clavate. Placoid sensillae absent. Distal flagellomeres of female not conspicuously enlarged compared to proximal flagellomeres.

*Pronotum*. Macrosculpture on lateral surface of pronotum present, dorsomedially foveate, laterally foveate-costate. Pubescence on lateral surface of pronotum present, sparse, composed of few short hairs. Carinae extending posteriorly from lateral margin of pronotal plate absent. Lateral pronotal carina present. Pronotal crest absent. Dorsal margin of pronotal plate (in anterior view) rounded. Lateral margin of pronotal plate defined all the way to the dorsal margin of the pronotum. Pronotal plate wide, almost as wide as head.

*Mesoscutum*. Mesoscutal surface convex, evenly curved. Sculpture on mesoscutum present, foveate-punctate, with remnants of transverse costae. Notaulus present, marked by series of deep subcontiguous pits of uniform width. Median mesoscutal carina absent. Anterior admedial lines present, flat, indistinct, with adjacent cuticular surface foveate. Median mesoscutal impression present, long, reaching over 1/2 length of mesoscutum. Parascutal carina distinctly sinuate, posteriorly ending in posteroventrally directed projection.

*Mesopleuron*. Dorsally irregularly horizontally costate with occasional fovea, ventrally smooth. Subpleuron entirely smooth, glabrous. Lower mesopleuron micro-pitted anteriorly, smooth and glabrous posteriorly. Epicnemial carina present on ventral half of mesopleuron; shagreened, ventrally bulbous near mesosternum. Lateroventral mesopleural carina present, marking abrupt change of slope of mesopectus. Mesopleural triangle absent. Subalar pit large and well defined, lying in posterior end of subalar groove. Speculum present, striate. Mesopleural carina absent.

*Scutellum*. Dorsal surface of scutellum foveate-areolate. Circumscutellar carina absent. Posterior margin of axillula marked by distinct ledge, axillula distinctly impressed adjacent to ledge. Lateroventral margin of scutellum posterior to auricula smooth, becoming dorsoventrally striate posteriorly. Scutellar spine less than 1.0× as long as petiole. Dorsal part of scutellum entirely rugose. Scutellar plate absent. Scutellar foveae present, three, with lateral fovea bissected by longitudinal carina, resulting in five longitudinally elongate subfoveae. Longitudinal scutellar carinae absent. Single longitudinal carina separating scutellar foveae absent. Posterolateral margin of scutellum drawn out into distinct protuberance. Lateral bar narrow, with strong strigate, foveate sculpture.

*Metapectal-propodial complex*. Metapectal cavity anterodorsal to metacoxal base present, ill-defined. Anterior margin of metapectal-propodeal complex separated from mesopleuron by deep, broad, uninterrupted marginal impression. Posteroventral corner of metapleuron (in lateral view) rounded, not drawn out posteriorly. Anterior impression of metepimeron present, narrow, linear impression, not broadened ventrally. Posterior margin of metepimeron distinct, separating metepimeron from propodeum. Subalar area slightly broadened anteriorly, without longitudinal division indicated. Calyptra present, blunt, lobe-like, polished posteriorly with setiferous punctures anteriorly. Anterior impression of metepisternum, immediately beneath anterior end of metapleural carina, present, small and narrow. Pubescence consisting of few scattered hairs on posterior part of metapleuron and lateral part of propodeum. Propodeal spurs present, foveate. Petiolar foramen removed from metacoxae, directed posteriorly. Calyptra, in lateral view, elongate. Propodeum relatively short, not drawn out posteriorly.

*Legs*. Pubescence posterolaterally on metacoxa sparse to moderately dense, confined dense hair patch absent. Microsculpture on hind coxa absent, hind coxa smooth. Longitudinal carina on the posterior surface of metatibia absent. Metafemoral spine present, elongate, extending distally as low keel along ventral femoral margin. Distal metatibial spurs equal in length to medial metatibial spurs.

*Forewing*. Pubescence of forewing absent on basal half of wing, sparse distally. Apical margin of female forewing rounded. Forewing Rs+M of forewing tubular. Mesal end of forewing Rs+M vein situated closer to posterior margin of wing, directed towards posterior end of forewing basalis. Vein R1 tubular along at least basal part of anterior margin of marginal cell. Basal abscissa of forewing R1 (the abscissa between forewing 2r and the wing margin) of forewing as broad as adjacent wing veins. Forewing entirely infuscate. Marginal cell of forewing membranous, similar to other wing cells. Areolet present, complete. Hair fringe along, apical margin of forewing absent glabrous.

*Petiole*. Slightly elongate, 1.5–2× longer than wide. Surface of petiole longitudinally costate, ventral keel of petiole absent.

*Metasoma*. Setal band (hairy ring) at base of tergum 3 absent, base of metasoma glabrous. Tergum 3 distinctly smaller than tergum 4. Posterior margin of tergum 3 smoothly rounded. Posterior margin of tergum 4 arcuate. In lateral view, sternum 3 exposed, ventral border of T2–T7 visible. Sculpture on metasomal terga present, finely punctate laterally and dorsally; posteriorly with large setal pits. Syntergum absent, all postpetiolar terga free. Annulus absent. Peg-like setae on T6–T7 absent. Posteroventral cavities of female metasoma T7 absent. Female posteroventral margin of T6–T7 straight, parallel.

**Figure 1.**
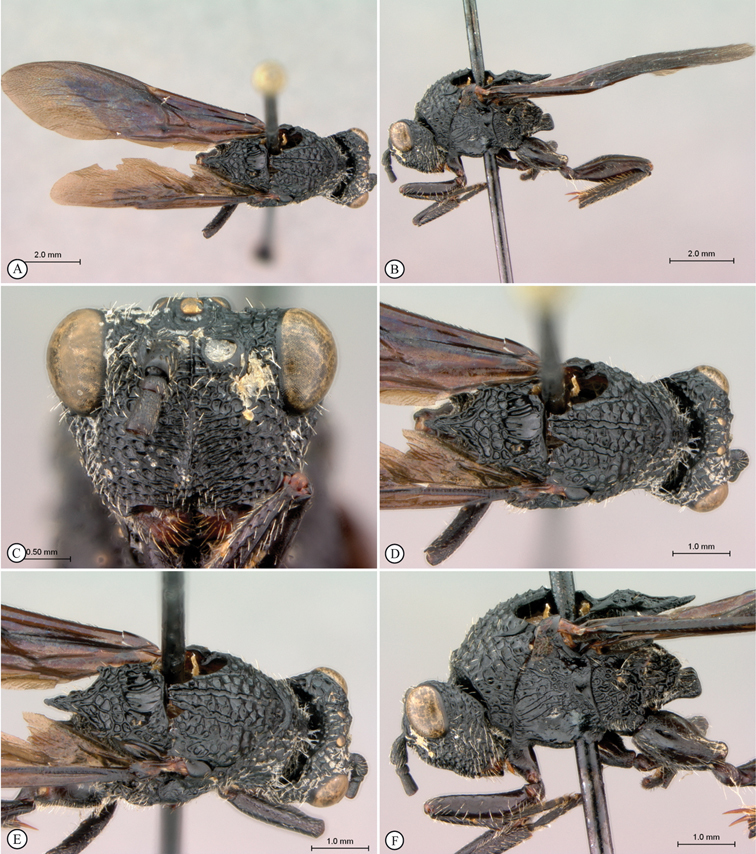
*Oberthuerella abscinda* Quinlan, holotype **A** dorsal habitus **B** lateral habitus **C** head, anterior view **D** head and mesosoma, dorsal view **E** head and mesosoma, dorsolateral view **E** head and mesosoma, lateral view.

**Figure 2.**
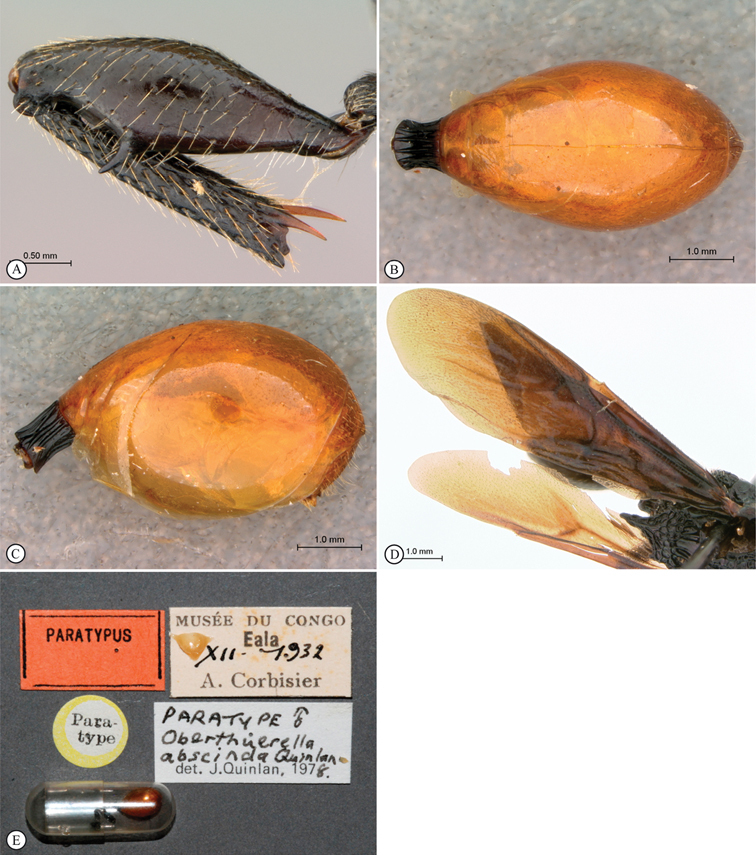
*Oberthuerella abscinda* Quinlan, holotype **A** hind femur and tibia **B** metasoma, dorsal view **C** metasoma, lateral view **D** fore and hind wings **E** labels.

##### Diagnosis.

This species most closely resembles *Oberthuerella longispinosa* and *Oberthuerella crassicornis*, and can be separated from the former by having an incomplete median keel on the face (median keel reaching the dorsal margin of the clypeus present in *Oberthuerella longispinosa* with a keel that reaches the dorsal clypeal margin), and from the latter by having yellow/orange legs (legs dark brown/black in *Oberthuerella abscinda*).

##### Distribution.

Democratic Republic of the Congo, Zambia**. Link to Distribution Map.** [http://hol.osu.edu/map-full.html?id=181552]

##### Material examined.

*Holotype* female: **ZAMBIA:** Mbala (‘Abercorn'), 31.XII.1943 (BMNH). *Paratype*: **DEMOCRATIC REPUBLIC OF THE CONGO:** Eala, XII-1932, A. Corbisier (1 female, MRAC 0014 (MRAC)); **ZAMBIA:** Mbala (‘Abercorn'), 31.XII.1943 (BMNH).

#### Oberthuerella aureopilosa

Benoit

urn:lsid:biosci.ohio-state.edu:osuc_concepts:181553

Morphbank accession: 704715–704728

http://www.waspweb.org/Cynipoidea/Liopteridae/Oberthuerellinae/Oberthuerella/Oberthuerella_aureopilosa.htm

http://species-id.net/wiki/Oberthuerella_aureopilosa

Figures 3 –4

Oberthuerella aureopilosa Benoit, 1955: 290.

##### Description.

Coloration of head and mesosoma black to dark brown, metasoma yellow-orange; legs reddish brown. Sculpture on vertex, lateral surface of pronotum and mesoscutum present, deeply foveate laterally on head, pronotum; deeply horizontally striate on mesoscutum.

*Head*. Broadly triangular, wider than high, in anterior view. Pubescence on head present, dense setae covering head. Sculpture along lateral margin of occiput absent. Gena (measured from compound eye to posterolateral margin of head) short, ratio of length of gena to length of compound eye in dorsal view < 0.3 in dorsal view. Sculpture of gena deeply striate. Lateral margin of occiput defined by distinctly angled, raised, sharp carina. Occiput (except extreme lateral margin) with some weak subvertical, irregular strigae. Ocelli small, ratio of maximum diameter of a lateral ocellus to shortest distance between lateral ocelli 0.2–0.4. Anterior ocellus close to posterior ocelli, posterior margin of anterior ocellus behind or subcontiguous with a transverse line running through anterior margins of posterior ocelli. Relative position of toruli close to ocelli, ratio of vertical distance between inner margin of torulus and ventral margin of clypeus to vertical distance between anterior ocellus and torulus < 2.0. Median keel of face present, short, not extending beyond toruli. Vertical carina adjacent to ventral margin of torulus absent. Facial sculpture present, punctate-rugose, transversely striate; striations meeting at midline of face. Facial impression absent, face flat. Antennal scrobe absent. Anterior tentorial pits large. Vertical delineations on lower face absent. Ventral clypeal margin laterally, close to anterior mandibular articulation, straight. Ventral clypeal margin medially straight, not projecting. Clypeus foveate-punctate. Malar space adjacent to anterior articulation of mandible evenly rounded, striate. Malar sulcus absent. Compound eye close to posterior ocellus, ratio of distance between compound eye and posterior mandibular articulation to distance between posterior ocellus and compound eye > 1.2. Compound eye, in dorsal view, distinctly protruding from the surface of the head, particularly laterally. Pubescence on compound eye absent. Orbital furrows absent. Lateral frontal carina of face absent. Dorsal aspect of vertex variously strigate. Posterior aspect of vertex foveate. Hair punctures on lateral aspect of vertex absent. Posterior surface of head almost flat, not deeply impressed.

*Labial-maxillary complex*. Apical segment of maxillary palp with pubescence, consisting only of erect setae. Apical seta on apical segment of maxillary palp shorter than twice length of second longest apical seta. Erect setae medially on apical segment of maxillary palp present. Last two segments of maxillary palp (in normal repose) straight.

*Pronotum*. Macrosculpture on lateral surface of pronotum present, deeply costulate with remnants of foveae. Pubescence on lateral surface of pronotum present, sparse, composed of few short hairs. Anterior flange of pronotal plate distinctly protruding anteriorly, smooth. Carinae extending posteriorly from lateral margin of pronotal plate absent. Lateral pronotal carina present. Pronotal crest absent. Dorsal margin of pronotal plate (in anterior view) rounded. Lateral margin of pronotal plate defined all the way to the dorsal margin of the pronotum. Pronotal plate wide, almost as wide as head.

*Mesoscutum*. Mesoscutal surface convex, evenly curved. Sculpture on mesoscutum present, transversely costate with dorsally projected serrations. Notaulus present, marked by deep furrows of uniform width. Median mesoscutal carina absent. Anterior admedial lines present, with adjacent cuticular surface horizontally striate. Median mesoscutal impression present, medium in length, reaching 1/4 length of mesoscutum. Parascutal carina distinctly sinuate, posteriorly ending in posteroventrally directed projection.

*Mesopleuron*. Horizontally strigulate, with striae converging along posterior margin of sclerite. Epicnemial carina present on ventral half of mesopleuron; shagreened, ventrally bulbous near mesosternum. Mesopleural triangle absent. Subalar pit large and well defined, lying in posterior end of subalar groove. Speculum present, smooth to micro-pitted.

*Scutellum*. Dorsal surface of scutellum foveate-areolate. Circumscutellar carina absent. Posterior margin of axillula marked by distinct ledge, axillula distinctly impressed adjacent to ledge. Lateroventral margin of scutellum posterior to auricula entirely smooth. Scutellum spine less than 1.0× length of petiole. Dorsal part of scutellum entirely rugose. Scutellar plate absent. Scutellar foveae present, three, each lateral fovea with two longitudinal divisions, central fovea smooth, resulting in transverse row of 7 longitudinally elongate subfovea. Longitudinal scutellar carinae absent. Single longitudinal carina separating scutellar foveae present, short, ending at posterior margin of foveae. Posterolateral margin of scutellum drawn out into distinct protuberance. Lateral bar narrow, with strong strigate, foveate sculpture.

*Metapectal-propodeal complex*. Metapectal cavity anterodorsal to metacoxal base present, ill-defined. Anterior margin of metapectal-propodeal complex separated from mesopleuron by deep, broad, uninterrupted marginal impression. Posteroventral corner of metapleuron (in lateral view) rounded, not drawn out posteriorly. Posterior margin of metepimeron distinct, separating metepimeron from propodeum. Calyptra present, blunt, lobe-like, polished posteriorly with setiferous punctures anteriorly. Dorsellum present, two strong medial fovea, laterally strongly excavated with fine pubescence in lateral depressions. Pubescence present along posterior and ventral margins of metapleuron, long, dense; long and thin on propodeum. Propodeal spurs present, foveate. Lateral propodeal carinae present, not reaching scutellum. Ventral end of lateral propodeal carina reaching nucha, carinae separated from each other. Inter propodeal carinae space densely setose. Petiolar foramen removed from metacoxae, directed posteriorly. Horizontal carina running anteriorly from lateral propodeal carina present. Lateral propodeal carina straight, sub-parallel. Calyptra, in lateral view, elongate. Propodeum relatively short, not drawn out posteriorly. Calyptra, in posterior view, rounded.

*Legs*. Pubescence posterolaterally on metacoxa sparse to moderately dense, confined dense hair patch absent. Metafemoral spine present, elongate, extending distally as low keel along ventral femoral margin. Ratio of first metatibial segment to remaining 4 segments greater than 1.0. Pubescence on outer surface of metatarsal claw sparse, consisting of few setae.

*Forewing*. Pubescence of forewing present, long, dense on most of surface. Apical margin of female forewing rounded. Rs+M of forewing tubular. Mesal end of Rs+M vein situated closer to posterior margin of forewing, directed towards posterior end of basalis. Vein R1 tubular along at least basal part of anterior margin of marginal cell. Basal abscissa of R1 (the abscissa between 2r and the forewing margin) of forewing as broad as adjacent wing veins. Forewing entirely infuscate. Marginal cell of forewing membranous, similar to other wing cells. Areolet present, complete. Hair fringe along apical margin of forewing absent.

*Petiole*. Petiole about as long as wide. Surface of petiole longitudinally costate, ventral keel absent. Posterior part of female petiole not abruptly widened. Ventral flange of annulus of female petiole absent.

*Metasoma*. Setal band (hairy ring) at base of tergum 3 present, interrupted dorsally, extending laterally to middle of sclerite. Tergum 3 distinctly smaller than tergum 4. Posterior margin of tergum 3 smoothly rounded. Posterior margin of tergum 4 straight. Sternum 3 exposed, ventral border of T2–T7 visible. Sculpture on metasomal terga present, composed to dense seta bearing punctures, interrupted dorsally on T3–5; dense setae present across entire metasomal surface. Syntergum absent, all postpetiolar terga free. Annulus absent. Peg-like setae on T6–T7 absent. Posteroventral cavities of female metasoma T7 present, setose. Female posteroventral margin of T6–T7 gently sinuate. Terebrum and hypopygium (in lateral view) straight, pointing posteriorly.

**Figure 3.**
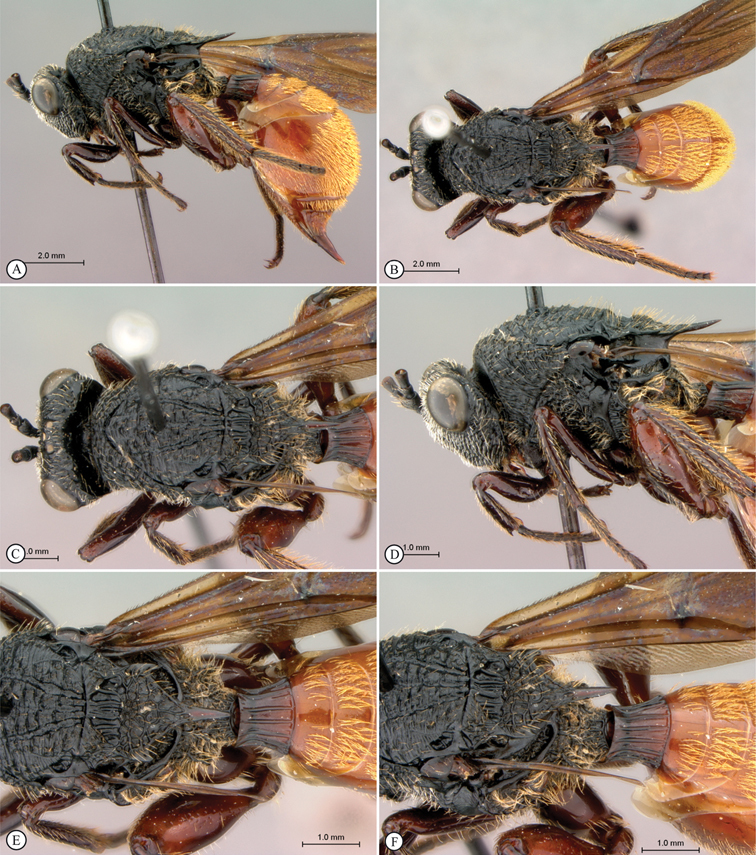
*Oberthuerella aureopilosa* Benoit, holotype **A** lateral habitus **B** dorsal habitus **C** head and mesosoma, dorsal view **D** head and mesosoma, lateral view **E** scutellum and petiole, dorsal view **F** scutellum and petiole, dorsolateral view.

**Figure 4.**
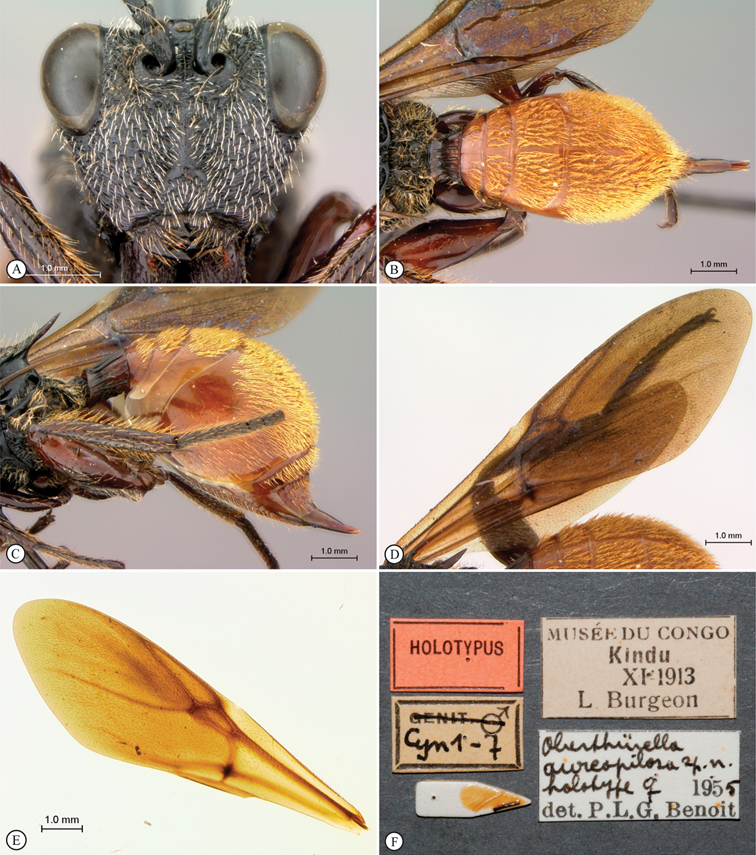
*Oberthuerella aureopilosa* Benoit, holotype **A** head, anterior view **B** metasoma, dorsal view **C** metasoma, lateral view **D** fore and hind wing **E** forewing **F** labels.

##### Diagnosis.

Easily distinguished from all other *Oberthuerella* by the predominance of golden setae on head, mesosoma, and metasoma; this feature is only shared with *Oberthuerella simba*, but this latter species has the speculum shagreened (smooth in *Oberthuerella aureopilosa*).

##### Distribution.

Democratic Republic of the Congo. **Link to Distribution Map.** [http://hol.osu.edu/map-full.html?id=181553]

##### Material examined.

Holotype, female: **DEMOCRATIC REPUBLIC OF THE CONGO:** Maniema Prov., Kindu, XI-1913, L. Burgeon, Mus. Cong. Cyn1-7 (deposited in MRAC).

#### Oberthuerella breviscutellaris

Benoit

urn:lsid:biosci.ohio-state.edu:osuc_concepts:181554

Morphbank accession: 704729–704740

http://www.waspweb.org/Cynipoidea/Liopteridae/Oberthuerellinae/Oberthuerella/Oberthuerella_breviscutellaris.htm

http://species-id.net/wiki/Oberthuerella_breviscutellaris

Figures 5 –6

Oberthuerella breviscutellaris Benoit, 1955: 286.

##### Description.

Coloration of head and mesosoma black to dark brown; metasoma, legs yellow-orange. Sculpture on vertex, lateral surface of pronotum and mesoscutum present, deeply striate on head, costate with remnants of foveae on pronotum, mesoscutum.

*Head*. Broadly triangular in anterior view. Pubescence on head present, sparse setae scattered over head. Sculpture along lateral margin of occiput absent. Gena (measured from compound eye to posterolateral margin of head) short, ratio of length of gena to length of compound eye in dorsal view < 0.3, in dorsal view. Sculpture of gena deeply striate. Lateral margin of occiput defined by distinctly angled, raised, sharp carina. Occiput (except extreme lateral margin) smooth. Ocelli small, ratio of maximum diameter of a lateral ocellus to shortest distance between lateral ocelli 0.2–0.4. Anterior ocellus close to posterior ocelli, posterior margin of anterior ocellus behind or subcontiguous with a transverse line running through anterior margins of posterior ocelli. Relative position of toruli close to ocelli, ratio of vertical distance between inner margin of torulus and ventral margin of clypeus to vertical distance between anterior ocellus and torulus < 2.0. Median keel of face present, extending to middle of face, not reaching clypeus. Vertical carina adjacent to ventral margin of torulus absent. Facial sculpture present, punctate-rugose, transversely striate; striations meeting at medial keel. Facial impression absent, face flat. Antennal scrobe absent. Anterior tentorial pits large. Vertical delineations on lower face absent. Ventral clypeal margin laterally, close to anterior mandibular articulation, straight. Ventral clypeal margin medially straight, not projecting. Clypeus horizontally striate. Malar space adjacent to anterior articulation of mandible evenly rounded, striate. Malar sulcus absent. Compound eye close to posterior ocellus, ratio of distance between compound eye and posterior mandibular articulation to distance between posterior ocellus and compound eye > 1.2. Compound eye, in dorsal view, distinctly protruding from the surface of the head, particularly laterally. Pubescence on compound eye absent. Orbital furrows absent. Lateral frontal carina of face absent. Dorsal aspect of vertex variously strigate. Posterior aspect of vertex foveate. Hair punctures on lateral aspect of vertex absent. Posterior surface of head almost flat, not deeply impressed.

*Labio-maxillary complex*. Apical segment of maxillary palp with pubescence, consisting only of erect setae. Apical seta on apical segment of maxillary palp shorter than twice length of second longest apical seta. Erect setae medially on apical segment of maxillary palp present. Last two segments of maxillary palp (in normal repose) straight. Distal margin of subapical segment of maxillary palp straight, apical segment bending outwards. Apical segment of maxillary palp more than 1.5 times as long as preceding segment.

*Antenna*. Articulation between flagellomeres in antenna connate with articles broadly joined. Female antenna composed of 11 flagellomeres. Female F1 shorter than F2; black. Flagellomeres of female antenna cylindrical, not widened towards apex, non-clavate. Placoidal sensilla absent. Distal flagellomeres of female antenna not conspicuously enlarged compared to proximal.

*Pronotum*. Macrosculpture on lateral surface of pronotum present, deeply costulate with remnants of foveae. Pubescence on lateral surface of pronotum present, sparse, composed of few short hairs. Anterior flange of pronotal plate distinctly protruding anteriorly, transversely striate. Carinae extending posteriorly from lateral margin of pronotal plate absent. Lateral pronotal carina present. Pronotal crest absent. Dorsal margin of pronotal plate (in anterior view) rounded. Submedian pronotal depressions closed laterally, deep. Lateral margin of pronotal plate defined all the way to the dorsal margin of the pronotum. Pronotal plate wide, almost as wide as head.

*Mesoscutum*. Mesoscutal surface convex, evenly curved. Sculpture on mesoscutum present, deeply transversely costate. Notaulus present, marked by series of deep subcontiguous pits of uniform width. Median mesoscutal carina absent. Anterior admedial lines present, with adjacent cuticular surface horizontally striate. Median mesoscutal impression present, short, indicated by notch. Parascutal carina distinctly sinuate, posteriorly ending in posteroventrally directed projection.

*Mesopleuron*. Horizontally strigulate, with striae converging along posterior margin of sclerite. Subpleuron entirely smooth with long, white setae over entire surface. Lower mesopleuron micro-pitted anteriorly, smooth and glabrous posteriorly. Epicnemial carina present, running from mesoscutum to anterior margin of mesopleural carina, narrow ventrally, costate. Lateroventral mesopleural carina present, marking abrupt change of slope of mesopectus. Mesopleural triangle absent. Subalar pit large and well defined, lying in posterior end of subalar groove. Speculum present, distinctly reticulate. Mesopleural carina absent.

*Scutellum*. Dorsal surface of scutellum foveate-areolate. Circumscutellar carina absent. Posterior margin of axillula marked by distinct ledge, axillula distinctly impressed adjacent to ledge. Lateroventral margin of scutellum posterior to auricula smooth, becoming dorsoventrally striate posteriorly. Dorsoposterior part of scutellum produced posteriorly into sharp spine, less than 1.0× length of petiole. Dorsal part of scutellum entirely rugose. Scutellar plate absent. Scutellar foveae present, two, each with four longitudinal divisions resulting in transverse row of 10 longitudinally elongate subfovea. Longitudinal scutellar carinae absent. Single longitudinal carina separating scutellar foveae present, short, ending at posterior margin of foveae. Posterolateral margin of scutellum drawn out into distinct protuberance. Lateral bar with strong strigate sculpture, narrow.

*Metapectal-propodeal complex*. Metapectal cavity anterodorsal to metacoxal base present, ill-defined. Anterior margin of metapectal-propodeal complex separated from mesopleuron by deep, broad, uninterrupted marginal impression. Posteroventral corner of metapleuron (in lateral view) rounded, not drawn out posteriorly. Anterior impression of metepimeron present, narrow, linear impression, not broadened ventrally. Posterior margin of metepimeron distinct, separating metepimeron from propodeum. Subalar area abruptly broadened anteriorly, with an indicated longitudinal division. Calyptra present, blunt, lobe-like, polished posteriorly with setiferous punctures anteriorly. Dorsellum present, two strong medial fovea, laterally strongly excavated with fine pubescence in lateral depressions. Anterior impression of metepisternum, immediately beneath anterior end of metapleural carina, absent. Pubescence thin, evenly covering entire metapectal-propodeal complex. Propodeal spurs present, crenulate. Lateral propodeal carinae present, not reaching scutellum. Ventral end of lateral propodeal carina terminating before reaching nucha. Inter propodeal carinae space lightly setose, foveate. Petiolar foramen removed from metacoxae, directed posteriorly. Horizontal carina running anteriorly from lateral propodeal carina present. Lateral propodeal carina straight, sub-parallel. Calyptra, in lateral view, elongate. Propodeum relatively short, not drawn out posteriorly. Calyptra, in posterior view, dorsoventrally elongate.

*Legs*. Pubescence posterolaterally on metacoxa sparse to moderately dense, confined dense hair patch absent. Microsculpture on hind coxa absent. Longitudinal carina on the posterior surface of metatibia absent. Metafemoral spine present, elongate, extending distally as low keel along ventral femoral margin. Distal mesotibial spurs shorter than medial spurs. Distal metatibial spurs shorter than medial spurs. Ratio of first metatibial segment to remaining 4 segments greater than 1.0. Pubescence on outer surface of metatarsal claw sparse, consisting of few setae. Outer surface of metatarsal claw microcarinate. Apical seta of metatarsal claw positioned on outer surface below dorsal margin. Base of metatarsal claw weakly expanded, apex slightly bent, ratio width of base to length of apex <0.6.

*Forewing*. Pubescence of forewing absent on basal half of wing, sparse distally. Apical margin of female forewing rounded. Rs+M of forewing tubular. Mesal end of Rs+M vein situated closer to posterior margin of forewing, directed towards posterior end of basalis. Vein R1 tubular along at least basal part of anterior margin of marginal cell. Basal abscissa of R1 (the abscissa between 2r and the forewing margin) of forewing as broad as adjacent forewing veins. Forewing entirely infuscate. Marginal cell of forewing membranous, similar to other wing cells. Areolet absent. Hair fringe along apical margin of forewing absent.

*Petiole*. Slightly elongate, 1.5–2× longer than wide. Surface of petiole longitudinally costate, ventral keel absent. Posterior part of female petiole not abruptly widened. Ventral flange of annulus of female petiole absent.

*Metasoma*. Setal band (hairy ring) at base of tergum 3 absent, base of metasoma glabrous. Tergum 3 distinctly smaller than tergum 4. Posterior margin of tergum 3 smoothly rounded. Posterior margin of tergum 4 arcuate. In lateral view, sternum 3 exposed, ventral border of T2–T7 visible. Sculpture on metasomal terga present, dorsally finely punctate, posteriorly with distinct bands of setiferous pits. Syntergum absent, all postpetiolar terga free. Annulus absent. Peg-like setae on T6–T7 present. Posteroventral cavities of female metasoma T7 present, setose. Female posteroventral margin of T6–T7 distinctly sinuate. Terebrum and hypopygium (in lateral view) straight, pointing posteriorly.

*Ovipositor*. First valvula of ovipositor narrowing gradually, not broadened apically, serrate at tip. Ovipositor clip absent.

**Figure 5.**
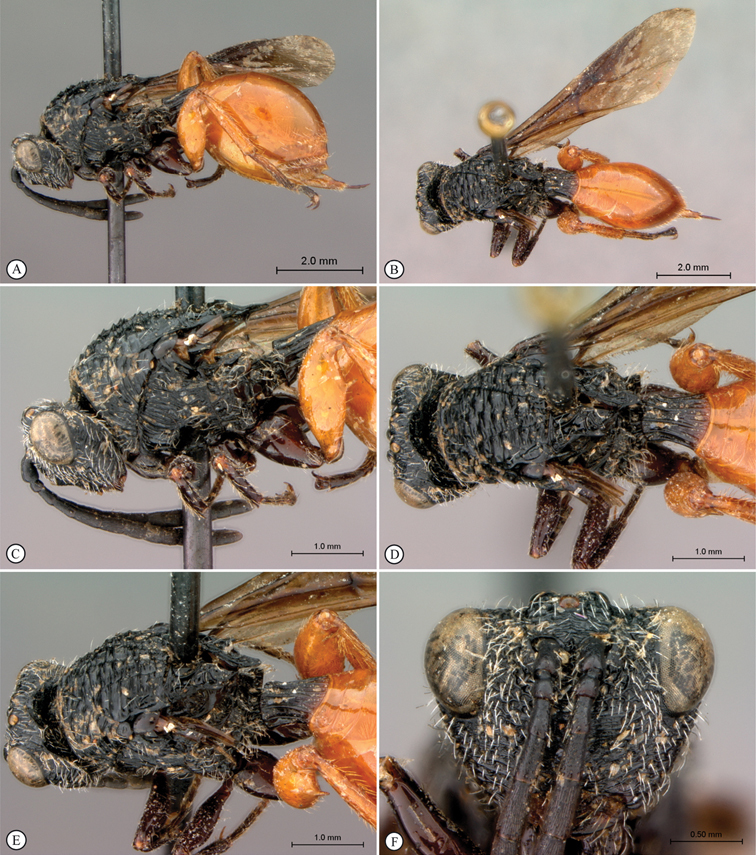
*Oberthuerella breviscutellaris* Benoit, holotype **A** lateral habitus **B** dorsal habitus **C** head and mesosoma, lateral view; D. head and mesosoma, dorsal view **E** head and mesosoma, dorsolateral view **F** head, anterior view.

**Figure 6.**
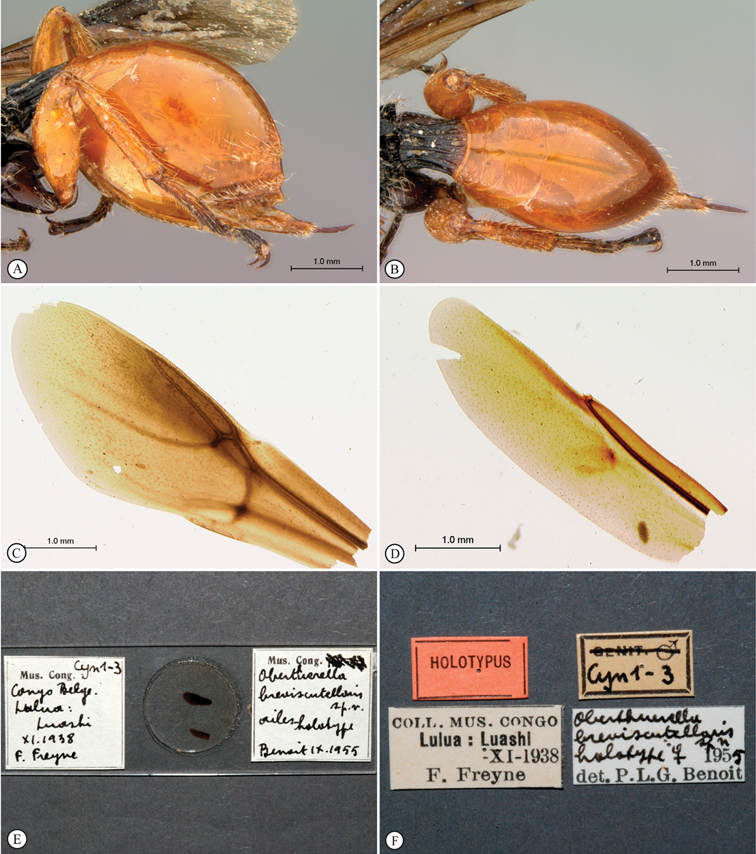
*Oberthuerella breviscutellaris* Benoit, holotype **A** metasoma, lateral view **B** metasoma, dorsal view **C** forewing **D** hindwing **E** wing slide overview **F** labels.

##### Diagnosis.

This species has a distinctly striate lateral aspect of the pronotum, as well as a horizontally striate mesopleuron; these features are shared with *Oberthuerella kibalensis* and *Oberthuerella sharkeyi*, but differs from the former by having an entirely orange metasoma (dark brown/black in *Oberthuerella kibalensis*), and differs from the latter having 10 subfovea present at the anterior base of the scutellum (4 subfovea in *Oberthuerella sharkeyi*).

##### Distribution.

Democratic Republic of Congo, Kenya, Zimbabwe. **Link to Distribution Map.** [http://hol.osu.edu/map-full.html?id=181554]

##### Material examined.

Holotype, female: **DEMOCRATIC REPUBLIC OF THE CONGO:** Lulua Prov., Luatshi (Luashi), XI-1938, F. Freyne, Mus. Cong. Cyn1-3 (deposited in MRAC). *Other material*: **KENYA:** Coast Prov., Shimba Hills, coastal rainforest, Makadara Forest, 04°13.651'S, 39°25.910'E, 22.X–5.XI.2008, R. Copeland (1 female, USNM ENT 00764776 (USNM)). (Further material listed in Quinlan, 1979).

#### Oberthuerella crassicornis

Benoit

urn:lsid:biosci.ohio-state.edu:osuc_concepts:181565

Morphbank accession: 704741–704753 (Oberthuerella compressa holotype); 704754–704766 (Oberthuerella crassicornis holotype)

http://www.waspweb.org/Cynipoidea/Liopteridae/Oberthuerellinae/Oberthuerella/Oberthuerella_crassicornis.htm

http://www.waspweb.org/Cynipoidea/Liopteridae/Oberthuerellinae/Oberthuerella/Oberthuerella_compressa.htm

http://species-id.net/wiki/Oberthuerella_crassicornis

**Figure 7.**
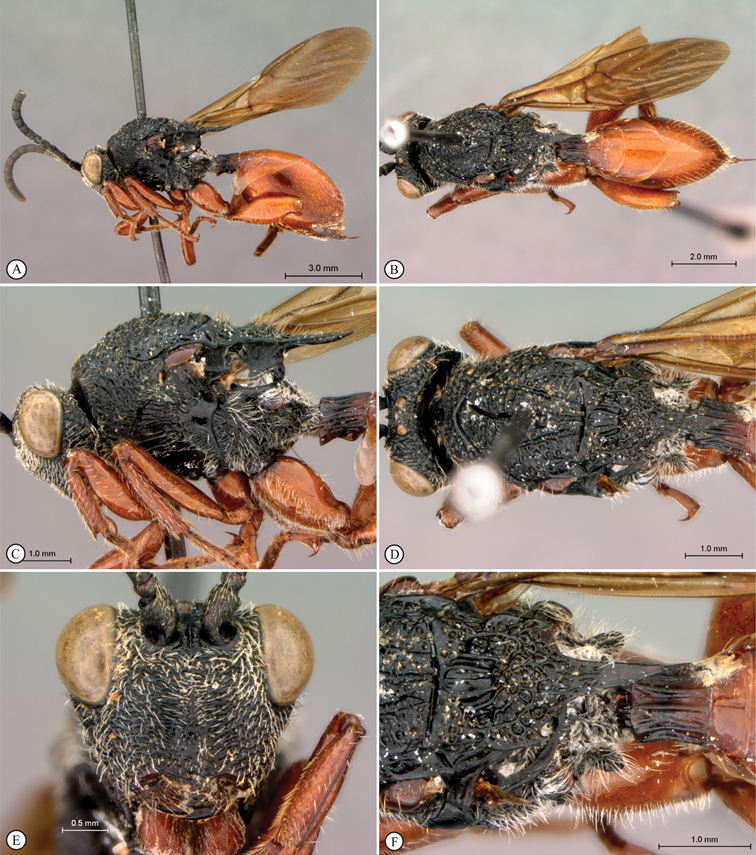
*Oberthuerella compressa* Benoit, holotype **A** lateral habitus **B** dorsal habitus **C** head and mesosoma, lateral view **D** head and mesosoma, dorsal view **E** head, anterior view **F** scutellum and petiole, dorsal view.

Figures 7 [Fig F8] [Fig F9] –10

Oberthuerella crassicornis Benoit, 1955: 289
Oberthuerella compressa Benoit, 1955: 292. Synonymy by Quinlan (1979).

##### Description.

Coloration of head and mesosoma black to dark brown; metasoma, legs yellow-orange. Sculpture on vertex, lateral surface of pronotum and mesoscutum present, deeply foveate laterally on head, pronotum; deeply horizontally striate on mesoscutum.

*Head*. Broadly triangular, in anterior view. Pubescence on head present, sparse setae scattered over head. Sculpture along lateral margin of occiput absent. Gena (measured from compound eye to posterolateral margin of head) short, ratio of length of gena to length of compound eye in dorsal view < 0.3, in dorsal view. Sculpture of gena present, with distinct fovea. Lateral margin of occiput defined by distinctly angled, raised, sharp carina. Occiput (except extreme lateral margin) smooth. Ocelli small, ratio of maximum diameter of a lateral ocellus to shortest distance between lateral ocelli 0.2–0.4. Anterior ocellus close to posterior ocelli, posterior margin of anterior ocellus behind or subcontiguous with a transverse line running through anterior margins of posterior ocelli. Relative position of toruli close to ocelli, ratio of vertical distance between inner margin of torulus and ventral margin of clypeus to vertical distance between anterior ocellus and torulus < 2.0. Median keel of face present, extending to middle of face, not reaching clypeus. Vertical carina adjacent to ventral margin of torulus absent. Facial sculpture almost entirely foveate, slightly horizontally striate along median keel. Facial impression absent, face flat. Antennal scrobe absent. Anterior tentorial pits large. Vertical delineations on lower face absent. Ventral clypeal margin laterally, close to anterior mandibular articulation, straight. Ventral clypeal margin medially straight, not projecting. Clypeus foveate-punctate; horizontally striate. Malar space adjacent to anterior articulation of mandible evenly rounded, foveate. Malar sulcus absent. Compound eye close to posterior ocellus, ratio of distance between compound eye and posterior mandibular articulation to distance between posterior ocellus and compound eye > 1.2. Compound eye, in dorsal view, distinctly protruding from the surface of the head, particularly laterally. Pubescence on compound eye absent. Orbital furrows absent. Lateral frontal carina of face absent. Dorsal aspect of vertex deeply foveate. Posterior aspect of vertex foveate. Hair punctures on lateral aspect of vertex absent. Posterior surface of head almost flat, not deeply impressed.

*Antenna*. Articulation between flagellomeres in antenna connate with articles broadly joined. Female antenna composed of 11 flagellomeres. Female F1 shorter than F2; black. Flagellomeres of female antenna cylindrical, not widened towards apex, non-clavate. Placoidal sensilla absent. Distal flagellomeres of female antenna not conspicuously enlarged compared to proximal flagellomeres.

*Pronotum*. Macrosculpture on lateral surface of pronotum present, dorsomedially foveate, laterally foveate-costate. Pubescence on lateral surface of pronotum present, sparse, composed of few short hairs. Carinae extending posteriorly from lateral margin of pronotal plate absent. Lateral pronotal carina present. Pronotal crest absent. Dorsal margin of pronotal plate (in anterior view) rounded. Lateral margin of pronotal plate defined all the way to the dorsal margin of the pronotum. Pronotal plate wide, almost as wide as head.

*Mesoscutum*. Mesoscutal surface convex, evenly curved. Sculpture on mesoscutum present, foveate-punctate, with remnants of transverse costae. Notaulus present, makred by deep furrows, slightly increasing in width posteriorly. Median mesoscutal carina absent. Anterior admedial lines present, flat, indistinct, with adjacent cuticular surface foveate. Median mesoscutal impression present, medium in length, reaching 1/4 length of mesoscutum. Parascutal carina distinctly sinuate, posteriorly ending in posteroventrally directed projection.

*Mesopleuron*. Horizontally strigulate, with striae converging on remnant fovea along posterior margin of sclerite. Subpleuron anteriorly smooth, polished, posteriorly with remnants of fovea. Lower mesopleuron medially smooth, glabrous; costate laterally, ventrally. Mesopleural triangle absent. Subalar pit large and well defined, lying in posterior end of subalar groove. Speculum present, smooth. Mesopleural carina absent.

*Scutellum*. Dorsal surface of scutellum foveate-areolate. Circumscutellar carina absent. Posterior margin of axillula marked by distinct ledge, axillula distinctly impressed adjacent to ledge. Lateroventral margin of scutellum posterior to auricula smooth, becoming dorsoventrally striate posteriorly. Dorsoposterior part of scutellum produced posteriorly into sharp spine, greater than 1.0× length of petiole. Dorsal part of scutellum entirely foveate. Scutellar plate absent. Scutellar foveae present, three, with lateral foveal bissected by longitudinal carina, resulting in five longitudinally elongate subfovea. Longitudinal scutellar carinae absent. Single longitudinal carina separating scutellar foveae absent. Posterolateral margin of scutellum drawn out into distinct protuberance. Lateral bar with strong strigate sculpture, narrow.

*Metapectal-propodeal complex*. Metapectal cavity anterodorsal to metacoxal base present, ill-defined. Anterior margin of metapectal-propodeal complex separated from mesopleuron by deep, broad, uninterrupted marginal impression. Posteroventral corner of metapleuron (in lateral view) rounded, not drawn out posteriorly. Anterior impression of metepimeron present, narrow, linear impression, not broadened ventrally. Posterior margin of metepimeron distinct, separating metepimeron from propodeum. Subalar area abruptly broadened anteriorly, with an indicated longitudinal division. Calyptra present, blunt, lobe-like, polished posteriorly with setiferous punctures anteriorly. Dorsellum present, two strong medial fovea, laterally strongly excavated with fine pubescence in lateral depressions. Anterior impression of metepisternum, immediately beneath anterior end of metapleural carina, present, small and narrow. Pubescence consisting of few scattered hairs on posterior part of metapleuron and lateral part of propodeum. Propodeal spurs present, foveate. Lateral propodeal carinae present, not reaching scutellum. Ventral end of lateral propodeal carina reaching nucha, carinae separated from each other. Inter propodeal carinae space densely setose. Petiolar foramen removed from metacoxae, directed posteriorly. Horizontal carina running anteriorly from lateral propodeal carina present. Lateral propodeal carina straight, sub-parallel. Calyptra, in lateral view, elongate. Propodeum relatively short, not drawn out posteriorly. Calyptra, in posterior view, dorsoventrally elongate.

*Legs*. Pubescence posterolaterally on metacoxa sparse to moderately dense, confined dense hair patch absent. Microsculpture on hind coxa absent. Longitudinal carina on the posterior surface of metatibia absent. Metafemoral spine present, elongate, extending distally as low keel along ventral femoral margin.

*Forewing*. Pubescence of forewing present, long, dense on most of surface. Apical margin of female forewing rounded. Rs+M of forewing tubular. Mesal end of Rs+M vein situated closer to posterior margin of forewing, directed towards posterior end of basalis. Vein R1 tubular along at least basal part of anterior margin of marginal cell. Basal abscissa of R1 (the abscissa between 2r and the forewing margin) of forewing as broad as adjacent wing veins. Forewing entirely infuscate. Marginal cell of forewing membranous, similar to other wing cells. Areolet absent. Hair fringe along apical margin of forewing absent.

*Petiole*. About as long as wide. Surface of petiole longitudinally costate, ventral keel absent. Posterior part of female petiole not abruptly widened. Ventral flange of annulus of female petiole absent.

*Metasoma*. Setal band (hairy ring) at base of tergum 3 absent, base of metasoma glabrous. Tergum 3 distinctly smaller than tergum 4. Posterior margin of tergum 3 smoothly rounded. Posterior margin of tergum 4 arcuate. In lateral view, sternum 3 exposed, ventral border of T2–T7 visible. Sculpture on metasomal terga present, finely punctate laterally, dorsally; posteriorly with large setal pits. Syntergum absent, all postpetiolar terga free. Annulus absent. Peg-like setae on T6–T7 absent. Posteroventral cavities of female metasoma T7 present, setose. Female posteroventral margin of T6–T7 distinctly sinuate. Terebrum and hypopygium (in lateral view) straight, pointing posteriorly.

*Ovipositor*. First valvula of ovipositor narrowing gradually, not broadened apically, smooth at tip. Ovipositor clip absent.

**Figure 8. F8:**
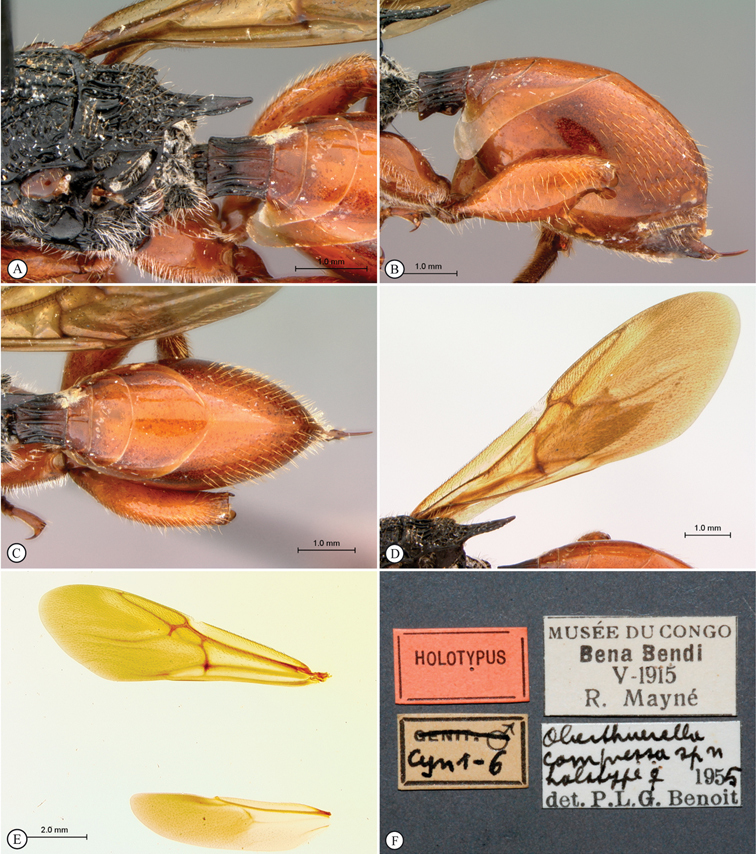
*Oberthuerella compressa* Benoit, holotype **A** scutellum and petiole, lateral view **B** metasoma, lateral view **C** metasoma, dorsal view **D** fore and hind wings **E** fore and hind wings **F** labels.

**Figure 9. F9:**
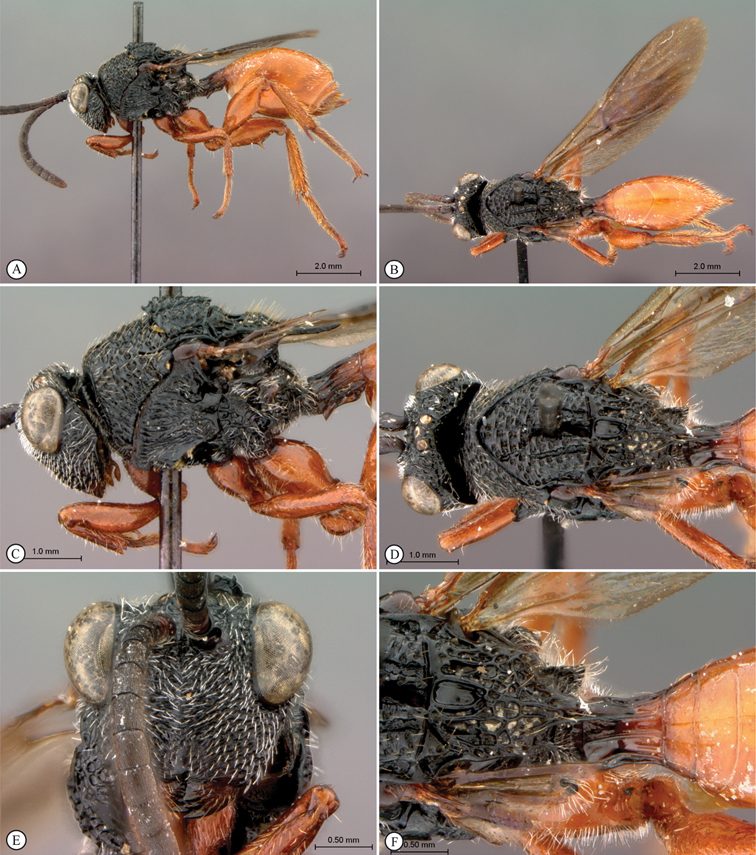
*Oberthuerella crassicornis* Benoit, holotype **A** lateral habitus **B** dorsal habitus **C** head and mesosoma, lateral view **D** head and mesosoma, dorsal view **E** head, anterior view **F** scutellum and petiole, dorsal view.

**Figure 10.**
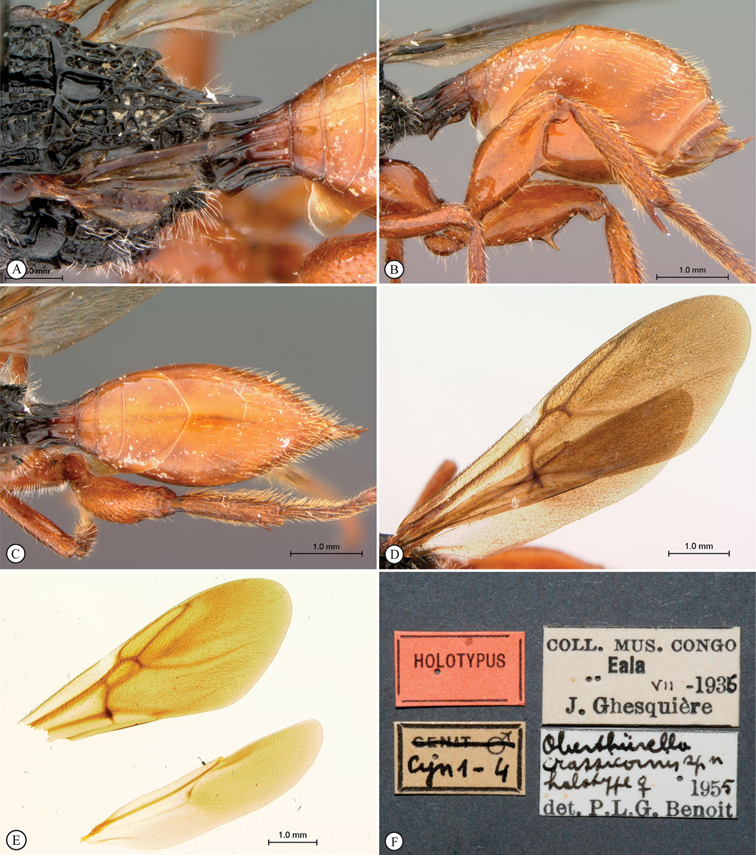
*Oberthuerella crassicornis* Benoit, holotype **A** scutellum and petiole, dorsolateral view **B** metasoma, lateral view **C** metasoma, dorsal view **D** fore and hind wings **E** fore and hind wings **F** labels.

##### Diagnosis.

Most easily confused with *Oberthuerella longispinosa* and *Oberthuerella abscinda*, but can be separated from the former by the incomplete median keel on the face (complete and of consistent width from the toruli to the dorsal margin of the clypeus), and from the latter by the legs being all yellow/orange (dark brown/black in *Oberthuerella abscinda*).

##### Distribution.

Democratic Republic of Congo, Malawi**. Link to Distribution Map.** [http://hol.osu.edu/map-full.html?id=181565]

##### Material examined.

Holotype, female: **DEMOCRATIC REPUBLIC OF THE CONGO:** Mai-Ndombe Prov., Bena-Bendi, V-1915, R. Mayné, Mus. Cong. Cyn1-6 (deposited in MRAC). Holotype, female, *Oberthuerella crassicornis*: **DEMOCRATIC REPUBLIC OF THE CONGO:** Eala, VII-1935, J. Ghesquière, Mus. Cong. Cyn1-4 (deposited in MRAC).

(Further material listed in Quinlan, 1979)

#### Oberthuerella cyclopia

Buffington & van Noort sp. n.

urn:lsid:zoobank.org:act:F1FACF31-3079-489C-8E8A-C067767BB267

urn:lsid:biosci.ohio-state.edu:osuc_concepts:300215

Morphbank accession: 704617–704625

http://www.waspweb.org/Cynipoidea/Liopteridae/Oberthuerellinae/Oberthuerella/Oberthuerella_cyclopia.htm

http://species-id.net/wiki/Oberthuerella_cyclopia

Figures 11 , 12A–C

##### Description.

Coloration of head and mesosoma black to dark brown; metasoma and legs yellow-orange. Sculpture on vertex, lateral surface of pronotum and mesoscutum present, deeply foveate laterally on head, pronotum; deeply horizontally striate on mesoscutum.

*Head*. Broadly triangular, in anterior view. Pubescence on head present, dense setation covering head. Sculpture along lateral margin of occiput absent. Gena (measured from compound eye to posterolateral margin of head) short, ratio of length of gena to length of compound eye in dorsal view < 0.3, in dorsal view. Sculpture of gena absent, smooth. Lateral margin of occiput defined by distinctly angled, raised, sharp carina. Occiput (except extreme lateral margin) with distinct subvertical, slightly and evenly curved costulae. Carina issuing from lateral margin of postocciput absent. Ocelli small, ratio of maximum diameter of a lateral ocellus to shortest distance between lateral ocelli 0.2-0.4. Anterior ocellus close to posterior ocelli, posterior margin of anterior ocellus behind or subcontiguous with a transverse line running through anterior margins of posterior ocelli. Relative position of toruli close to ocellus, ratio of vertical distance between inner margin of torulus and ventral margin of clypeus to vertical distance between anterior ocellus and torulus < 2.0. Median keel of face present, short, not extending beyond toruli. Vertical carina adjacent to ventral margin of torulus absent. Facial sculpture present, punctate-rugose, transversely striate; striations meeting at midline of face. Facial impression absent, face flat. Antennal scrobe absent. Anterior tentorial pits large. Vertical delineations on lower face present, with several parallel or subparallel carinae. Ventral clypeal margin laterally, close to anterior mandibular articulation, distinctly angled. Ventral clypeal margin medially emarginate. Clypeus horizontally striate. Malar space adjacent to anterior articulation of mandible evenly rounded, striate. Malar sulcus absent. Compound eye close to posterior ocellus, ratio of distance between compound eye and posterior mandibular articulation to distance between posterior ocellus and compound eye > 1.2. Compound eye, in dorsal view, distinctly protruding from the surface of the head, particularly laterally. Pubescence on compound eye absent. Orbital furrows absent. Lateral frontal carina of face present. Dorsal aspect of vertex shagreened with faint remnants of carinae. Posterior aspect of vertex smooth. Hair punctures on lateral aspect of vertex absent. Posterior surface of head almost flat, not deeply impressed.

*Labial-maxillary complex*. Apical segment of maxillary palp with pubescence, consisting only of erect setae. First segment of labial palp as long as apical segment. Labial palp composed of three segments. Apical seta on apical segment of maxillary palp shorter than twice length of second longest apical seta. Erect setae medially on apical segment of maxillary palp present. Maxillary palp composed of three segments. Last two segments of maxillary palp (in normal repose) curved inwards. Distal margin of subapical segment of maxillary palp slanting inwards, apical segment bending inwards. Apical segment of maxillary palp 1–1.5 times as long as preceding segment.

*Antenna*. Articulation between flagellomeres in antenna connate with articles broadly joined. Female antenna composed of 11 flagellomeres. Female F1 as long as F2; black. Flagellomeres of female antenna cylindrical, not widened towards apex, non-clavate. Placoidal sensilla absent. Distal flagellomeres of female antenna not conspicuously enlarged compared to proximal flagellomeres.

*Pronotum*. Macrosculpture on lateral surface of pronotum present, foveate. Anteroventral inflection of pronotum broad, particularly adjacent to anterior part of pronotal plate. Pubescence on lateral surface of pronotum absent. Carinae extending posteriorly from lateral margin of pronotal plate absent. Lateral pronotal carina present. Pronotal crest absent. Dorsal margin of pronotal plate (in anterior view) rounded. Submedian pronotal depressions closed laterally, deep. Lateral margin of pronotal plate defined all the way to the dorsal margin of the pronotum. Pronotal plate wide, almost as wide as head.

*Mesoscutum*. Mesoscutal surface convex, evenly curved. Sculpture on mesoscutum present, foveate-punctate, with remnants of transverse costae. Notaulus present, marked by series of deep subcontiguous pits of uniform width. Median mesoscutal carina absent. Anterior admedial lines present, flat, indistinct, with adjacent cuticular surface foveate. Median mesoscutal impression present, long, reaching over 1/2 length of mesoscutum. Parascutal carina distinctly sinuate, posteriorly ending in posteroventrally directed projection.

*Mesopleuron*. Dorsally irregularly horizontally costate with occasional fovea, ventrally smooth. Subpleuron entirely smooth, glabrous. Lower mesopleuron medially smooth, glabrous; costate laterally, ventrally. Epicnemial carina present, running from mesoscutum to anterior margin of mesopleural carina, ventrally bulbous. Lateroventral mesopleural carina present, marking abrupt change of slope of mesopectus. Mesopleural triangle absent. Subalar pit large and well defined, lying in posterior end of subalar groove. Speculum present, distinctly reticulate. Mesopleural carina present, complete, composed of several long, irregular, curved carinae. Anterior end of mesopleural carina inserting above notch in anterior margin of mesopleuron.

*Scutullum*. Dorsal surface of scutellum irregularly rugulose; foveate-areolate. Circumscutellar carina absent. Posterior margin of axillula marked by distinct ledge, axillula distinctly impressed adjacent to ledge. Lateroventral margin of scutellum posterior to auricula entirely smooth. Dorsoposterior part of scutellum produced posteriorly into sharp spine, greater than 1.0× length of petiole. Dorsal part of scutellum entirely rugose. Scutellar plate absent. Scutellar foveae present, two, each with two longitudinal divisions resulting in transverse row of 6 longitudinally elongate subfovea. Longitudinal scutellar carinae absent. Single longitudinal carina separating scutellar foveae present, short, ending before scutellar spine. Posterolateral margin of scutellum drawn out into distinct protuberance. Lateral bar with strong strigate sculpture, narrow.

*Metapectal-propodeal complex*. Metapectal cavity anterodorsal to metacoxal base present, ill-defined. Anterior margin of metapectal-propodeal complex separated from mesopleuron by deep, broad, uninterrupted marginal impression. Posteroventral corner of metapleuron (in lateral view) rounded, not drawn out posteriorly. Anterior impression of metepimeron present, narrow, linear impression, not broadened ventrally. Posterior margin of metepimeron distinct, separating metepimeron from propodeum. Subalar area slightly broadened anteriorly, with distinct laterally protruding lobe ventrally. Calyptra present, blunt, lobe-like, polished posteriorly with setiferous punctures anteriorly. Dorsellum present, smooth, glabrous. Anterior impression of metepisternum, immediately beneath anterior end of metapleural carina, present, small and narrow. Pubescence thin, evenly covering entire metapectal-propodeal complex. Propodeal spurs present, crenulate. Lateral propodeal carinae present, not reaching scutellum. Ventral end of lateral propodeal carina terminating before reaching nucha. Inter propodeal carinae space densely setose. Petiolar foramen removed from metacoxae, directed posteriorly. Horizontal carina running anteriorly from lateral propodeal carina present. Lateral propodeal carina straight, sub-parallel. Calyptra, in lateral view, elongate. Propodeum relatively short, not drawn out posteriorly. Calyptra, in posterior view, dorsoventrally elongate.

*Legs*. Pubescence posterolaterally on metacoxa sparse to moderately dense, confined dense hair patch absent. Microsculpture on hind coxa absent. Longitudinal carina on the posterior surface of metatibia absent. Metafemoral spine present, elongate, extending distally as low keel along ventral femoral margin. Distal mesotibial spurs shorter than medial spurs. Distal metatibial spurs shorter than medial spurs. Ratio of first metatibial segment to remaining 4 segments greater than 1.0. Pubescence on outer surface of metatarsal claw sparse, consisting of few setae. Outer surface of metatarsal claw entirely smooth. Apical seta of metatarsal claw positioned on outer surface below dorsal margin. Base of metatarsal claw weakly expanded, apex slightly bent, ratio width of base to length of apex <0.6.

*Forewing*. Pubescence of forewing present, sparse across entire wing surface. Apical margin of female forewing rounded. Rs+M of forewing tubular. Mesal end of Rs+M vein situated closer to posterior margin of forewing, directed towards posterior end of basalis. Vein R1 tubular along at least basal part of anterior margin of marginal cell. Basal abscissa of R1 (the abscissa between 2r and the forewing margin) of forewing as broad as adjacent wing veins. Forewing entirely lightly infuscate. Marginal cell of forewing membranous, similar to other wing cells. Areolet present, complete. Hair fringe along apical margin of forewing present, very short.

*Petiole*. Stout, 1.5–1.75× wider than long. Surface of petiole longitudinally costate, ventral keel absent. Posterior part of female petiole not abruptly widened. Ventral flange of annulus of female petiole absent.

*Metasoma*. Setal band (hairy ring) at base of tergum 3 absent, base of metasoma glabrous. Tergum 3 distinctly smaller than tergum 4. Posterior margin of tergum 3 smoothly rounded. Posterior margin of tergum 4 arcuate. In lateral view, sternum 3 exposed, ventral border of T2–T7 visible. Sculpture on metasomal terga present, dorsally finely punctate, posteriorly with distinct bands of setiferous pits. Syntergum absent, all postpetiolar terga free. Annulus absent. Peg-like setae on T6–T7 absent. Posteroventral cavities of female metasoma T7 present, glabrous save for few, long setae. Female posteroventral margin of T6–T7 gently sinuate. Terebrum and hypopygium (in lateral view) straight, pointing posteriorly.

*Ovipositor*. Ovipositor clip absent.

**Figure 11.**
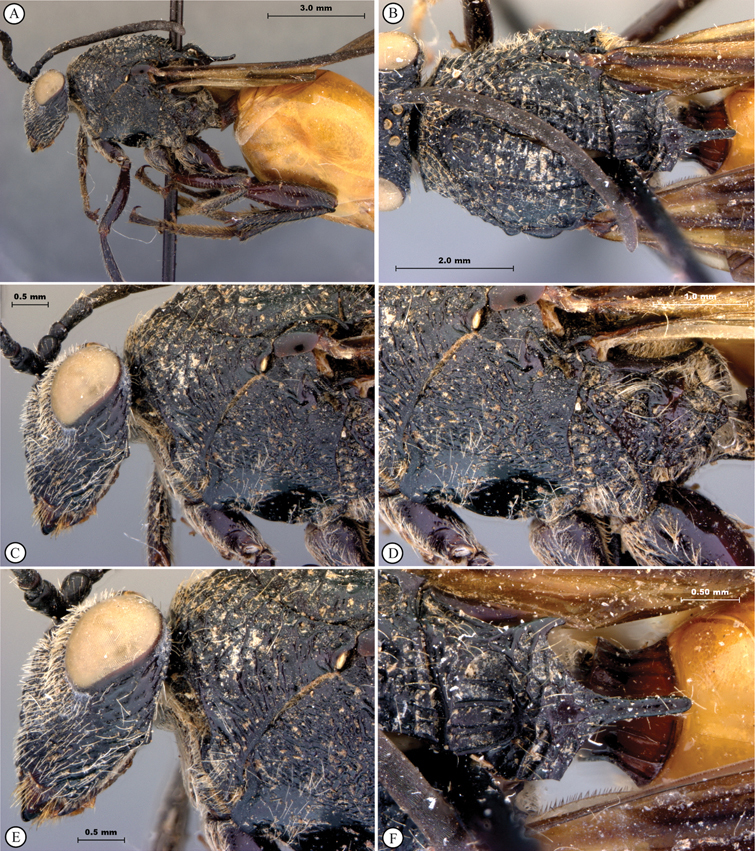
*Oberthuerella cyclopia* Buffington & van Noort, sp. n., holotype **A** lateral habitus **B** mesosoma, dorsal view **C** head and mesosoma, lateral view **D** meso- and metapleurae, lateral view **E**  head and pronotum, lateral view **F** scutellum and petiole, dorsal view.

**Figure 12.**
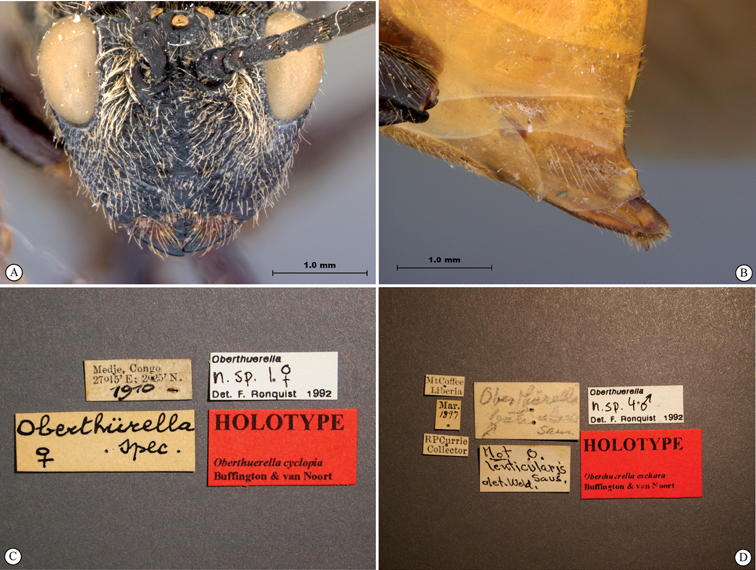
*Oberthuerella cyclopia* Buffington & van Noort, sp. n., holotype **A** head anterior view **B** posterior margin of metasoma, lateral view **C** labels **D** *Oberthuerella eschara* Buffington & van Noort, sp. n., holotype label.

##### Diagnosis.

Distinguished from other *Oberthuerella* by dorsally crenulate, ventrally shagreened speculum. Most easily confused with *Oberthuerella lenticularis*, but separated by the median keel on face extending beyond the toruli (ending before ventral margin of toruli in *Oberthuerella lenticularis*).

##### Etymology.

*Cyclops* is a Greek name for a mythical, giant primordial race of humanoids with a single eye in their forehead; the name refers to the unusually large size of this species.

##### Distribution.

Democratic Republic of the Congo. **Link to Distribution Map.** [http://hol.osu.edu/map-full.html?id=300215]

##### Material examined.

Holotype, female: **DEMOCRATIC REPUBLIC OF THE CONGO:** Medje, 02°25'N, 27°15'E, 1910, USNM ENT 00764781 (deposited in MCZC).

#### Oberthuerella eschara

Buffington & van Noort sp .n.

urn:lsid:zoobank.org:act:5DCA6558-ECAA-4622-8C9E-D47C90C15A76

urn:lsid:biosci.ohio-state.edu:osuc_concepts:300216

Morphbank accession: 704626–704636

http://www.waspweb.org/Cynipoidea/Liopteridae/Oberthuerellinae/Oberthuerella/Oberthuerella_eschara.htm

http://species-id.net/wiki/Oberthuerella_eschara

**Figure 13.**
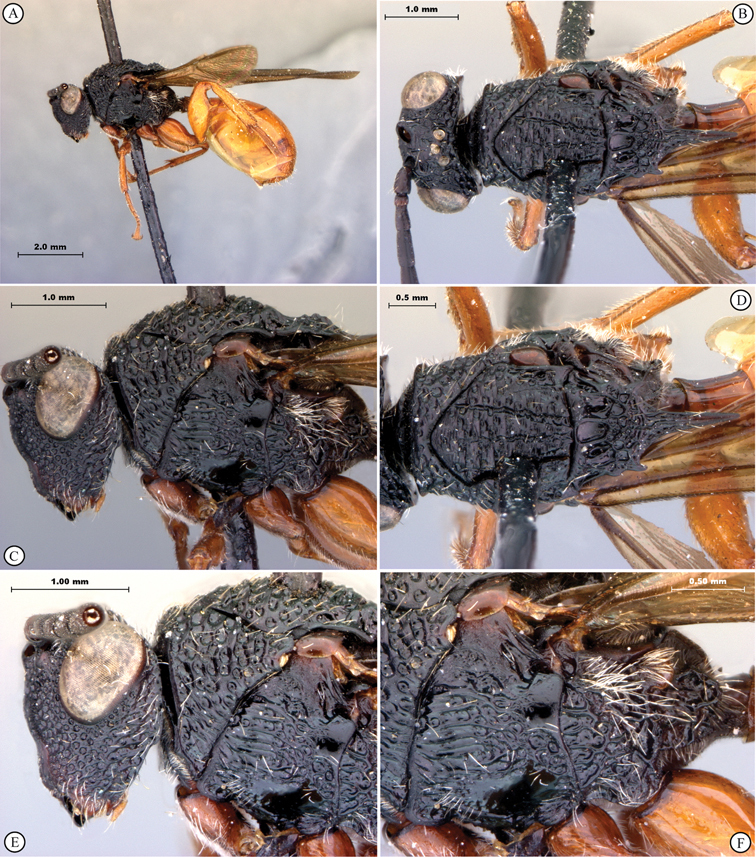
*Oberthuerella eschara* Buffington & van Noort, sp. n., holotype **A** lateral habitus **B** head and mesosoma, dorsal view **C** head and mesosoma, lateral view **D** mesosoma, dorsal view, lateral view **E** head and pronotum, lateral view **F** meso and metapleurae.

**Figure 14.**
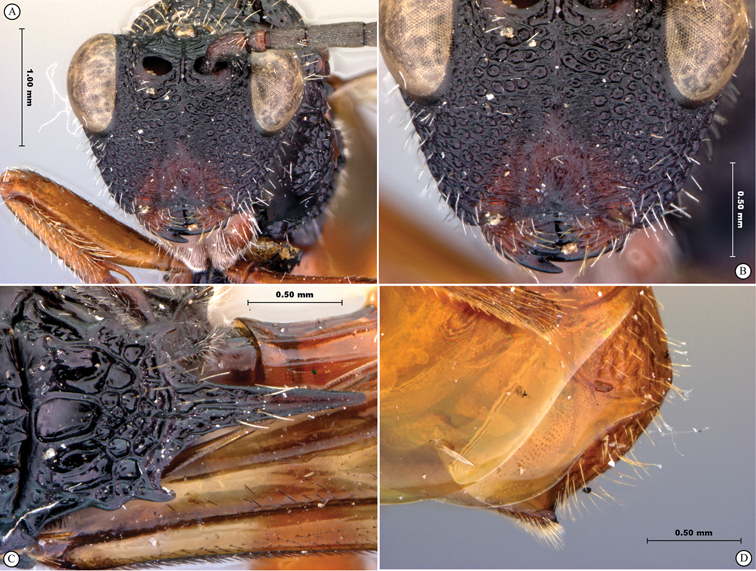
*Oberthuerella eschara* Buffington & van Noort, sp. n., holotype **A** head anterior view **B** face **C** scutellum, dorsal view **D** posterior margin of metasoma, lateral view.

Figures 12D , 13 –14

##### Description.

Coloration of head and mesosoma, black to dark brown; metasoma, legs yellow-orange. Sculpture on vertex, lateral surface of pronotum and mesoscutum present, deeply striate on head, costate with remnants of foveae on pronotum, mesoscutum.

*Head*. Broadly triangular, in anterior view. Pubescence on head present, sparse setae scattered over head. Sculpture along lateral margin of occiput absent. Gena (measured from compound eye to posterolateral margin of head) short, ratio of length of gena to length of compound eye in dorsal view < 0.3, in dorsal view. Sculpture of gena deeply striate with remnants of fovea. Lateral margin of occiput defined by distinctly angled, raised, sharp carina. Occiput (except extreme lateral margin) smooth. Ocelli small, ratio of maximum diameter of a lateral ocellus to shortest distance between lateral ocelli 0.2–0.4. Anterior ocellus close to posterior ocelli, posterior margin of anterior ocellus behind or subcontiguous with a transverse line running through anterior margins of posterior ocelli. Relative position of toruli close to ocelli, ratio of vertical distance between inner margin of torulus and ventral margin of clypeus to vertical distance between anterior ocellus and torulus < 2.0. Median keel of face present, extending to middle of face, not reaching clypeus. Vertical carina adjacent to ventral margin of torulus absent. Facial sculpture almost entirely foveate, slightly horizontally striate along median keel. Facial impression absent, face flat. Antennal scrobe absent. Anterior tentorial pits large. Vertical delineations on lower face present, represented by series of tight lateral striations along median. Ventral clypeal margin laterally, close to anterior mandibular articulation, straight. Ventral clypeal margin medially emarginate. Clypeus circumscribed by clypeal carina; surface striate, converging ventro-medially. Malar space adjacent to anterior articulation of mandible evenly rounded, foveate. Malar sulcus absent. Compound eye close to posterior ocellus, ratio of distance between compound eye and posterior mandibular articulation to distance between posterior ocellus and compound eye > 1.2. Compound eye, in dorsal view, distinctly protruding from the surface of the head, particularly laterally. Pubescence on compound eye absent. Orbital furrows absent. Lateral frontal carina of face absent. Dorsal aspect of vertex deeply foveate. Posterior aspect of vertex punctate. Hair punctures on lateral aspect of vertex present, indistinct. Posterior surface of head almost flat, not deeply impressed.

*Labial-maxillary complex*. Apical segment of maxillary palp with pubescence, consisting only of erect setae. Apical seta on apical segment of maxillary palp shorter than twice length of second longest apical seta. Erect setae medially on apical segment of maxillary palp present. Maxillary palp composed of three segments. Last two segments of maxillary palp (in normal repose) straight. Distal margin of subapical segment of maxillary palp straight, apical segment bending outwards. Apical segment of maxillary palp more than 1.5 times as long as preceding segment.

*Antenna*. Articulation between flagellomeres in antenna connate with articles broadly joined. Placoidal sensilla absent. Second flagellomere of male antenna cylindrical; black. Length of second flagellomere of male antenna longer than first flagellomere.

*Pronotum*. Macrosculpture on lateral surface of pronotum present, dorsomedially foveate, laterally foveate-costate. Pubescence on lateral surface of pronotum present, sparse, composed of few short hairs. Anterior flange of pronotal plate distinctly protruding anteriorly, transversely striate. Carinae extending posteriorly from lateral margin of pronotal plate absent. Lateral pronotal carina present. Pronotal crest absent. Dorsal margin of pronotal plate (in anterior view) straight. Submedian pronotal depressions closed laterally, deep. Lateral margin of pronotal plate defined all the way to the dorsal margin of the pronotum. Pronotal plate wide, almost as wide as head.

*Mesoscutum*. Mesoscutal surface convex, evenly curved. Sculpture on mesoscutum present, deeply transversely costate. Notaulus present, marked by series of deep subcontiguous pits of uniform width. Median mesoscutal carina absent. Anterior admedial lines present, with adjacent cuticular surface horizontally striate. Median mesoscutal impression present, long, reaching over 1/2 length of mesoscutum. Parascutal carina distinctly sinuate, posteriorly ending in posteroventrally directed projection.

*Mesopleuron*. Dorsally irregularly horizontally costate with occasional fovea, ventrally smooth. Subpleuron entirely smooth, glabrous. Lower mesopleuron micro-pitted anteriorly, smooth and glabrous posteriorly. Epicnemial carina present, running from mesoscutum to anterior margin of mesopleural carina, spread out ventrally, shagreened. Lateroventral mesopleural carina present, marking abrupt change of slope of mesopectus. Mesopleural triangle absent. Subalar pit large and well defined, lying in posterior end of subalar groove. Speculum present, smooth to micro-pitted. Mesopleural carina present, complete, composed of several long, parallel, straight carinae. Anterior end of mesopleural carina inserting above notch in anterior margin of mesopleuron.

*Scutellum*. Dorsal surface of scutellum foveate-areolate. Circumscutellar carina absent. Posterior margin of axillula marked by distinct ledge, axillula distinctly impressed adjacent to ledge. Lateroventral margin of scutellum posterior to auricula smooth, becoming dorsoventrally striate posteriorly. Dorsoposterior part of scutellum produced posteriorly into sharp spine, greater than 1.0× length of petiole. Dorsal part of scutellum entirely rugose. Scutellar plate absent. Scutellar foveae present, three, with lateral foveal bissected by longitudinal carina, resulting in five longitudinally elongate subfovea. Longitudinal scutellar carinae absent. Single longitudinal carina separating scutellar foveae absent. Posterolateral margin of scutellum drawn out into distinct protuberance. Lateral bar with strong strigate sculpture, narrow.

*Metapectal-propodeal complex*. Metapectal cavity anterodorsal to metacoxal base present, ill-defined. Anterior margin of metapectal-propodeal complex separated from mesopleuron by deep, broad, uninterrupted marginal impression. Posteroventral corner of metapleuron (in lateral view) rounded, not drawn out posteriorly. Anterior impression of metepimeron present, narrow, linear impression, not broadened ventrally. Posterior margin of metepimeron distinct, separating metepimeron from propodeum. Subalar area abruptly broadened anteriorly, with an indicated longitudinal division. Calyptra present, blunt, lobe-like, polished posteriorly with setiferous punctures anteriorly. Dorsellum present with two strong medial fovea, glabrous. Anterior impression of metepisternum, immediately beneath anterior end of metapleural carina, present, small and narrow. Pubescence thin, evenly covering entire metapectal-propodeal complex. Propodeal spurs present, crenulate. Lateral propodeal carinae present, not reaching scutellum. Ventral end of lateral propodeal carina terminating before reaching nucha. Inter propodeal carinae space lightly setose, horizontally striate. Petiolar foramen removed from metacoxae, directed posteriorly. Horizontal carina running anteriorly from lateral propodeal carina present. Lateral propodeal carina straight, sub-parallel. Calyptra, in lateral view, elongate. Propodeum relatively short, not drawn out posteriorly. Calyptra, in posterior view, dorsoventrally elongate.

*Legs*. Pubescence posterolaterally on metacoxa sparse to moderately dense, confined dense hair patch absent. Microsculpture on hind coxa absent. Longitudinal carina on the posterior surface of metatibia absent. Metafemoral spine present, elongate, extending distally as low keel along ventral femoral margin. Distal mesotibial spurs equal in length to medial spurs. Distal metatibial spurs equal in length to medial spurs. Ratio of first metatibial segment to remaining 4 segments uncertain, greater than 1.0.

*Forewing*. Pubescence of forewing absent on basal half of wing, sparse distally. Apical margin of female forewing rounded. Rs+M of forewing tubular. Mesal end of Rs+M vein situated closer to posterior margin of forewing, directed towards posterior end of basalis. Vein R1 tubular along at least basal part of anterior margin of marginal cell. Basal abscissa of R1 (the abscissa between 2r and the forewing margin) of forewing as broad as adjacent wing veins. Forewing entirely lightly infuscate. Marginal cell of forewing membranous, similar to other wing cells. Areolet absent. Hair fringe along apical margin of forewing absent.

*Petiole*. Slightly elongate, 1.5–2× longer than wide. Surface of petiole longitudinally costate, ventral keel absent. Posterior part of female petiole not abruptly widened. Ventral flange of annulus of female petiole absent.

*Metasoma*. Setal band (hairy ring) at base of tergum 3 absent, base of metasoma glabrous. Tergum 3 distinctly smaller than tergum 4. Posterior margin of tergum 3 smoothly rounded. Posterior margin of tergum 4 arcuate. In lateral view, sternum 3 exposed, ventral border of T2–T7 visible. Sculpture on metasomal terga present, dorsally finely punctate, laterally and ventrally smooth. Syntergum absent, all postpetiolar terga free. Annulus absent. Peg-like setae on T6–T7 absent. Female posteroventral margin of T6–T7 straight, parallel.

##### Diagnosis.

Distinguished from all other species of *Oberthuerella* by the unique lower facial sculpturing of the head, with two convergent, densely horizontally striate bands that meet mid-face. In other species, the face ranges from entirely foveate to horizontally striate, but never with two distinct bands of striations. This species is further characterized by the circum clypeal carina; this character has not been observed in any other species of *Oberthuerella*.

##### Etymology.

Latin for *scar*, in reference to the scar-like dorso-ventral bands on the face.

**Distribution.** Liberia. **Link to Distribution Map.** [http://hol.osu.edu/map-full.html?id=300216]

##### Material examined.

Holotype, male: **LIBERIA:** Montserrado Co., Mount Coffee, III-1897, R. P. Currie, USNM ENT 00764774 (deposited in USNM).

#### Oberthuerella kibalensis

van Noort & Buffington sp. n.

urn:lsid:zoobank.org:act:7B2D6FC2-230C-43B3-B9F5-0D3ACAF4FBA9

urn:lsid:biosci.ohio-state.edu:osuc_concepts:300217

Morphbank accession: 704767–704776

http://www.waspweb.org/Cynipoidea/Liopteridae/Oberthuerellinae/Oberthuerella/Oberthuerella_kibalensis.htm

http://species-id.net/wiki/Oberthuerella_kibalensis

Figures 15 –16

##### Description.

Coloration of head, mesosoma, and metasoma black to dark brown; legs dark reddish brown. Sculpture on vertex, lateral surface of pronotum and mesoscutum present, deeply striate on head, costate with remnants of foveae on pronotum, mesoscutum.

*Head*. Broadly triangular in anterior view; wider than high. Pubescence on head present, dense setae covering head. Sculpture along lateral margin of occiput absent. Gena (measured from compound eye to posterolateral margin of head) short, ratio of length of gena to length of compound eye in dorsal view < 0.3, in dorsal view. Sculpture of gena deeply striate. Lateral margin of occiput defined by distinctly angled, raised, sharp carina. Occiput (except extreme lateral margin) smooth. Ocelli small, ratio of maximum diameter of a lateral ocellus to shortest distance between lateral ocelli 0.2–0.4. Anterior ocellus close to posterior ocelli, posterior margin of anterior ocellus behind or subcontiguous with a transverse line running through anterior margins of posterior ocelli. Relative position of toruli close to ocelli, ratio of vertical distance between inner margin of torulus and ventral margin of clypeus to vertical distance between anterior ocellus and torulus < 2.0. Median keel of face present, short, not extending beyond toruli. Vertical carina adjacent to ventral margin of torulus absent. Facial sculpture transversely striate with remnants of foveae. Facial impression absent, face flat. Antennal scrobe absent. Anterior tentorial pits large. Vertical delineations on lower face absent. Ventral clypeal margin laterally, close to anterior mandibular articulation, distinctly angled. Ventral clypeal margin medially emarginate. Clypeus horizontally striate. Malar space adjacent to anterior articulation of mandible evenly rounded, striate-foveate. Malar sulcus absent. Compound eye close to posterior ocellus, ratio of distance between compound eye and posterior mandibular articulation to distance between posterior ocellus and compound eye > 1.2. Compound eye, in dorsal view, distinctly protruding from the surface of the head, particularly laterally. Pubescence on compound eye absent. Orbital furrows absent. Lateral frontal carina of face absent. Dorsal aspect of vertex deeply foveate. Posterior aspect of vertex foveate. Hair punctures on lateral aspect of vertex present, indistinct. Posterior surface of head almost flat, not deeply impressed.

*Antenna*. Articulation between flagellomeres in antenna connate with articles broadly joined. Female antenna composed of 11 flagellomeres. Female F1 shorter than F2; black. Flagellomeres of female antenna cylindrical, not widened towards apex, non-clavate. Placoidal sensilla absent. Distal flagellomeres of female antenna not conspicuously enlarged compared to proximal flagellomeres.

*Pronotum*. Macrosculpture on lateral surface of pronotum present, deeply costulate with remnants of foveae. Pubescence on lateral surface of pronotum present, sparse, composed of few short hairs. Anterior flange of pronotal plate distinctly protruding anteriorly, smooth. Carinae extending posteriorly from lateral margin of pronotal plate absent. Lateral pronotal carina present. Pronotal crest absent. Dorsal margin of pronotal plate (in anterior view) rounded. Submedian pronotal depressions closed laterally, shallow. Lateral margin of pronotal plate defined all the way to the dorsal margin of the pronotum. Pronotal plate wide, almost as wide as head.

*Mesoscutum*. Mesoscutal surface convex, evenly curved. Sculpture on mesoscutum present, foveate-punctate, with remnants of transverse costae. Notaulus present, marked by deep furrows, slightly increasing in width posteriorly. Median mesoscutal carina absent. Anterior admedial lines absent. Median mesoscutal impression present, long, reaching over 1/2 length of mesoscutum. Parascutal carina distinctly sinuate, posteriorly ending in posteroventrally directed projection.

*Mesopleuron*. Horizontally strigulate, with striae converging on rugose sculpture on posterior one-third of sclerite. Subpleuron anteriorly strigate, posteriorly smooth; medially with sparse, long setae. Lower mesopleuron medially smooth, setose; costate laterally, ventrally. Epicnemial carina absent. Lateroventral mesopleural carina present, marking abrupt change of slope of mesopectus. Mesopleural triangle absent. Subalar pit large and well defined, lying in posterior end of subalar groove. Speculum present, smooth. Mesopleural carina absent.

*Scutellum*. Dorsal surface of scutellum foveate-areolate. Circumscutellar carina absent. Posterior margin of axillula marked by distinct ledge, axillula distinctly impressed adjacent to ledge. Lateroventral margin of scutellum posterior to auricula smooth, with single shallow fovea dorsomedially. Dorsoposterior part of scutellum produced posteriorly into sharp spine, less than 1.0× length of petiole. Dorsal part of scutellum entirely foveate. Scutellar plate absent. Scutellar foveae present, three, with lateral foveal bissected by longitudinal carina, resulting in five longitudinally elongate subfovea. Longitudinal scutellar carinae absent. Single longitudinal carina separating scutellar foveae absent. Posterolateral margin of scutellum drawn out into distinct protuberance. Lateral bar narrow, with strong strigate, foveate sculpture.

*Metapectal-propodeal complex*. Metapectal cavity anterodorsal to metacoxal base present, ill-defined. Anterior margin of metapectal-propodeal complex separated from mesopleuron by deep, broad, uninterrupted marginal impression. Posteroventral corner of metapleuron (in lateral view) rounded, not drawn out posteriorly. Anterior impression of metepimeron present, narrow, linear impression, not broadened ventrally. Posterior margin of metepimeron distinct, separating metepimeron from propodeum. Subalar area slightly broadened anteriorly, without longitudinal division indicated. Calyptra present, blunt, lobe-like, polished posteriorly with setiferous punctures anteriorly. Dorsellum present, two strong medial fovea, laterally strongly excavated with fine pubescence in lateral depressions. Anterior impression of metepisternum, immediately beneath anterior end of metapleural carina, present, small and narrow. Pubescence consisting of few scattered hairs on posterior part of metapleuron and lateral part of propodeum. Propodeal spurs present, crenulate. Lateral propodeal carinae present, not reaching scutellum. Ventral end of lateral propodeal carina reaching nucha, carinae separated from each other. Inter propodeal carinae space glabrous, horizontally striate. Petiolar foramen removed from metacoxae, directed posteriorly. Horizontal carina running anteriorly from lateral propodeal carina present. Lateral propodeal carina straight, sub-parallel. Calyptra, in lateral view, elongate. Propodeum relatively short, not drawn out posteriorly. Calyptra, in posterior view, dorsoventrally elongate.

*Legs*. Pubescence posterolaterally on metacoxa sparse to moderately dense, confined dense hair patch absent. Microsculpture on hind coxa present antero-laterally, smooth posterolaterally. Longitudinal carina on the posterior surface of metatibia absent. Metafemoral spine present, elongate, extending distally as low keel along ventral femoral margin. Distal mesotibial spurs longer than medial spurs. Distal metatibial spurs equal in length to medial spurs. Ratio of first metatibial segment to remaining 4 segments equal to 1.0. Pubescence on outer surface of metatarsal claw sparse, consisting of few setae. Outer surface of metatarsal claw microcarinate. Apical seta of metatarsal claw positioned on outer surface below dorsal margin. Base of metatarsal claw weakly expanded, apex slightly bent, ratio width of base to length of apex <0.6.

*Forewing*. Pubescence of forewing present, long, dense on most of surface. Apical margin of female forewing rounded. Rs+M of forewing tubular. Mesal end of Rs+M vein situated closer to posterior margin of forewing, directed towards posterior end of basalis. Vein R1 tubular along at least basal part of anterior margin of marginal cell. Basal abscissa of R1 (the abscissa between 2r and the forewing margin) of forewing as broad as adjacent wing veins. Forewing entirely lightly infuscate. Marginal cell of forewing membranous, similar to other wing cells. Areolet absent. Hair fringe along apical margin of forewing absent.

*Petiole*. Petiole slightly elongate, 1.5–2× longer than wide. Surface of petiole longitudinally costate, ventral keel absent. Posterior part of female petiole not abruptly widened. Ventral flange of annulus of female petiole absent.

*Metasoma*. Setal band (hairy ring) at base of tergum 3 absent, base of metasoma glabrous. Tergum 3 distinctly smaller than tergum 4. Posterior margin of tergum 3 slightly but distinctly concave. Posterior margin of tergum 4 arcuate. In lateral view, sternum 3 exposed, ventral border of T2–T7 visible. Sculpture on metasomal terga present, dorsally finely punctate, posteriorly with distinct bands of setiferous pits. Syntergum absent, all postpetiolar terga free. Annulus absent. Peg-like setae on T6–T7 present. Posteroventral cavities of female metasoma T7 present, setose. Female posteroventral margin of T6–T7 distinctly sinuate. Terebrum and hypopygium (in lateral view) straight, pointing posteriorly.

*Ovipositor*. First valvula of ovipositor narrowing gradually, not broadened apically, smooth at tip. Ovipositor clip absent.

**Figure 15.**
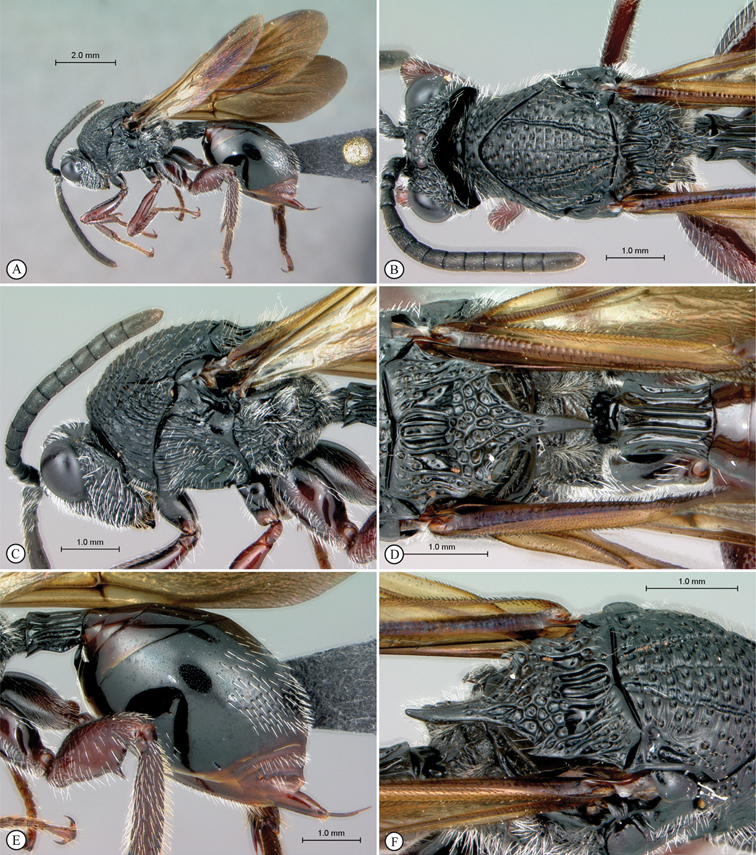
*Oberthuerella kibalensis* van Noort & Buffington, sp. n., holotype **A** lateral habitus **B** head and mesosoma, dorsal view **C** head and mesosoma, lateral view **D** scutellum and petiole, dorsal view **E** metasoma, lateral view **F** scutellum, dorsolateral view.

**Figure 16.**
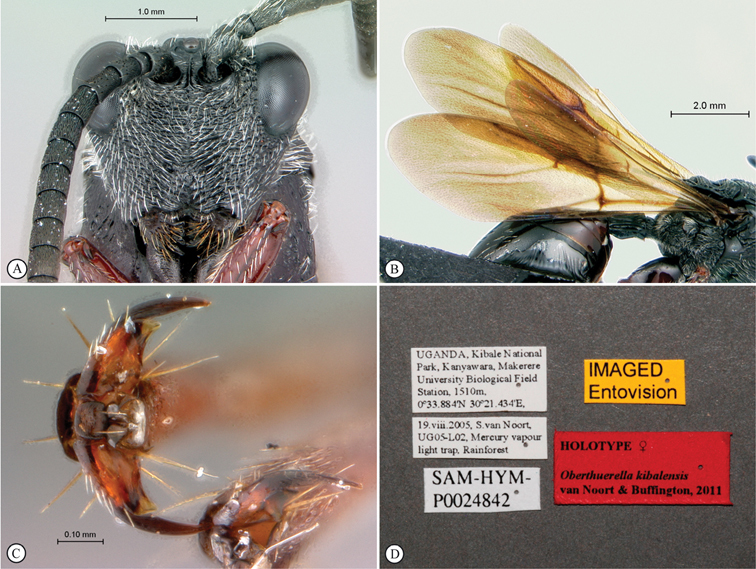
*Oberthuerella kibalensis* van Noort & Buffington, sp. n., holotype **A** head, anterior view **B** fore and hind wings **C** hind claw **D** labels.

##### Diagnosis.

Similar to *Oberthuerella nigrescens* Benoit, but pubescence on metasoma and legs silvery white instead of golden orange, and face, pronotal and mesopleural sculpture different: pronotum laterally with longitudinal striations; mesopleuron anteriorly with strong longitudinal striations grading into weak punctures in dorso-posterior half; metasoma (except for tergites 1,2 and 6, which are black) and legs reddish brown; fine scattered white pubescence on upper lateral surface of metasomal segments 5–7, as well as on hind femur and all tibia and tarsi; face with strong striations meeting between anterior tentorial pits; eye length equal to cheek length, whereas *Oberthuerella nigrescens* eye length is distinctly longer (1.23×) than length of gena.

##### Etymology.

Named after Kibale Forest, Uganda, the type locality.

##### Distribution.

Uganda. **Link to Distribution Map.** [http://hol.osu.edu/map-full.html?id=300217]

##### Material examined.

Holotype, female: **UGANDA:** Kibale National Park, Kanyawara, Makerere University Biological Field Station (MUBFS), 00°33.884'N, 30°21.434'E, 1510m, 19.VIII.2005, S. van Noort, mercury vapor light, UG05-L02, rainforest, SAM-HYM-P0024842 (deposited in SAMC).

#### Oberthuerella lenticularis

Saussure

urn:lsid:biosci.ohio-state.edu:osuc_concepts:181556

Morphbank accession: 704637–704645

http://www.waspweb.org/Cynipoidea/Liopteridae/Oberthuerellinae/Oberthuerella/Oberthuerella_lenticularis.htm

http://species-id.net/wiki/Oberthuerella_lenticularis

Figures 17 –18

Oberthuerella lenticularis Saussure, 1890: plate 20; fig. 8. Redescription by Kieffer (1904).

##### Description.

Coloration of head and mesosoma, black to dark brown; metasoma and legs yellow-orange. Sculpture on vertex, lateral surface of pronotum and mesoscutum present, deeply foveate laterally on head, pronotum; deeply horizontally striate on mesoscutum.

*Head*. Broadly triangular, in anterior view. Pubescence on head present, sparse setae scattered over head. Sculpture along lateral margin of occiput many costulae. Gena (measured from compound eye to posterolateral margin of head) short, ratio of length of gena to length of compound eye in dorsal view < 0.3, in dorsal view. Sculpture of gena present, gently striate. Lateral margin of occiput defined by distinctly angled, raised, sharp carina. Occiput (except extreme lateral margin) smooth. Ocelli small, ratio of maximum diameter of a lateral ocellus to shortest distance between lateral ocelli 0.2–0.4. Anterior ocellus close to posterior ocelli, posterior margin of anterior ocellus behind or subcontiguous with a transverse line running through anterior margins of posterior ocelli. Relative position of toruli close to ocelli, ratio of vertical distance between inner margin of torulus and ventral margin of clypeus to vertical distance between anterior ocellus and torulus < 2.0. Median keel of face present, short, not extending beyond toruli. Vertical carina adjacent to ventral margin of torulus absent. Facial sculpture present, punctate-rugose, transversely striate; striations meeting at midline of face. Facial impression absent, face flat. Antennal scrobe absent. Anterior tentorial pits large. Vertical delineations on lower face absent. Ventral clypeal margin laterally, close to anterior mandibular articulation, distinctly angled. Ventral clypeal margin medially emarginate. Clypeus foveate-punctate. Malar space adjacent to anterior articulation of mandible evenly rounded, striate. Malar sulcus absent. Compound eye close to posterior ocellus, ratio of distance between compound eye and posterior mandibular articulation to distance between posterior ocellus and compound eye > 1.2. Compound eye, in dorsal view, distinctly protruding from the surface of the head, particularly laterally. Pubescence on compound eye absent. Orbital furrows absent. Lateral frontal carina of face absent. Dorsal aspect of vertex deeply foveate. Posterior aspect of vertex smooth. Hair punctures on lateral aspect of vertex absent. Posterior surface of head almost flat, not deeply impressed.

*Labial-maxillary complex*. Apical segment of maxillary palp with pubescence, consisting only of erect setae.

*Antenna*. Articulation between flagellomeres in antenna connate with articles broadly joined. Female antenna composed of 11 flagellomeres. Female F1 shorter than F2; black. Flagellomeres of female antenna cylindrical, not widened towards apex, non-clavate. Placoidal sensilla absent. Distal flagellomeres of female antenna not conspicuously enlarged compared to proximal flagellomeres.

*Pronotum*. Macrosculpture on lateral surface of pronotum present, foveate. Pubescence on lateral surface of pronotum absent. Anterior flange of pronotal plate distinctly protruding anteriorly, centrally smooth, longitudinally striate laterally. Carinae extending posteriorly from lateral margin of pronotal plate absent. Lateral pronotal carina present. Pronotal crest absent. Dorsal margin of pronotal plate (in anterior view) rounded. Submedian pronotal depressions closed laterally, deep. Lateral margin of pronotal plate defined all the way to the dorsal margin of the pronotum. Pronotal plate wide, almost as wide as head.

*Mesoscutum*. Mesoscutal surface convex, evenly curved. Sculpture on mesoscutum present, foveate-punctate, with remnants of transverse costae. Notaulus present, marked by series of deep subcontiguous pits of uniform width. Median mesoscutal carina absent. Anterior admedial lines present, with adjacent cuticular surface foveate. Median mesoscutal impression present, medium in length, reaching 1/4 length of mesoscutum. Parascutal carina distinctly sinuate, posteriorly ending in posteroventrally directed projection.

*Mesopleuron*. Dorsally irregularly horizontally costate with occasional fovea, ventrally smooth. Subpleuron entirely smooth with long, white setae on ventral half. Lower mesopleuron medially smooth, glabrous; costate laterally, ventrally. Epicnemial carina present, running from mesoscutum to anterior margin of mesopleural carina, ventrally bulbous. Lateroventral mesopleural carina present, marking abrupt change of slope of mesopectus. Mesopleural triangle absent. Subalar pit large and well defined, lying in posterior end of subalar groove. Speculum present, distinctly reticulate. Mesopleural carina present, incomplete, composed of one straight carina indicated anteriorly, otherwise smooth. Anterior end of mesopleural carina inserting above notch in anterior margin of mesopleuron.

*Scutellum*. Dorsal surface of scutellum foveate-areolate. Circumscutellar carina absent. Posterior margin of axillula marked by distinct ledge, axillula distinctly impressed adjacent to ledge. Lateroventral margin of scutellum posterior to auricula entirely smooth. Dorsoposterior part of scutellum produced posteriorly into sharp spine, greater than 1.0× length of petiole. Dorsal part of scutellum entirely rugose. Scutellar plate absent. Scutellar foveae present, two, each with two longitudinal divisions resulting in transverse row of 6 longitudinally elongate subfovea. Longitudinal scutellar carinae absent. Single longitudinal carina separating scutellar foveae present, long, continuing posteriorly to scutellar spine. Lateral bar smooth, narrow.

*Metapectal-propodeal complex*. Metapectal cavity anterodorsal to metacoxal base present, ill-defined. Anterior margin of metapectal-propodeal complex separated from mesopleuron by deep, broad, uninterrupted marginal impression. Posteroventral corner of metapleuron (in lateral view) rounded, not drawn out posteriorly. Anterior impression of metepimeron present, narrow, linear impression, not broadened ventrally. Posterior margin of metepimeron distinct, separating metepimeron from propodeum. Subalar area slightly broadened anteriorly, with distinct laterally protruding lobe ventrally. Calyptra present, blunt, lobe-like, polished posteriorly with setiferous punctures anteriorly. Dorsellum present, smooth, glabrous. Anterior impression of metepisternum, immediately beneath anterior end of metapleural carina, present, small and narrow. Pubescence consisting of few scattered hairs on posterior part of metapleuron and lateral part of propodeum. Propodeal spurs present, crenulate. Lateral propodeal carinae present, not reaching scutellum. Ventral end of lateral propodeal carina terminating before reaching nucha. Inter propodeal carinae space glabrous, costulate. Petiolar foramen removed from metacoxae, directed posteriorly. Horizontal carina running anteriorly from lateral propodeal carina absent. Lateral propodeal carina curved distally. Calyptra, in lateral view, elongate. Propodeum relatively short, not drawn out posteriorly. Calyptra, in posterior view, dorsoventrally elongate.

*Legs*. Pubescence posterolaterally on metacoxa sparse to moderately dense, confined dense hair patch absent. Microsculpture on hind coxa absent. Longitudinal carina on the posterior surface of metatibia absent. Metafemoral spine present, elongate, extending distally as low keel along ventral femoral margin. Distal mesotibial spurs shorter than medial spurs. Distal metatibial spurs shorter than medial spurs. Ratio of first metatibial segment to remaining 4 segments greater than 1.0. Pubescence on outer surface of metatarsal claw sparse, consisting of few setae. Outer surface of metatarsal claw entirely smooth. Apical seta of metatarsal claw positioned on outer surface below dorsal margin. Base of metatarsal claw weakly expanded, apex slightly bent, ratio width of base to length of apex <0.6.

*Forewing*. Pubescence of forewing present, sparse across entire wing surface. Apical margin of female forewing rounded. Rs+M of forewing tubular. Mesal end of Rs+M vein situated closer to posterior margin of forewing, directed towards posterior end of basalis. Vein R1 tubular along at least basal part of anterior margin of marginal cell. Basal abscissa of R1 (the abscissa between 2r and the forewing margin) of forewing as broad as adjacent wing veins. Forewing entirely lightly infuscate. Marginal cell of forewing membranous, similar to other wing cells. Areolet present, incomplete, open posteriorly. Hair fringe along apical margin of forewing present, very short.

*Petiole*. Slightly elongate, 1.5–2× longer than wide. Surface of petiole longitudinally costate, ventral keel absent. Posterior part of female petiole not abruptly widened. Ventral flange of annulus of female petiole absent.

*Metasoma*. Setal band (hairy ring) at base of tergum 3 absent, base of metasoma glabrous. Tergum 3 distinctly smaller than tergum 4. Posterior margin of tergum 3 smoothly rounded. Posterior margin of tergum 4 arcuate. In lateral view, sternum 3 exposed, ventral border of T2–T7 visible. Sculpture on metasomal terga present, dorsally finely punctate, posteriorly with distinct bands of setiferous pits. Syntergum absent, all postpetiolar terga free. Annulus absent. Peg-like setae on T6–T7 present. Posteroventral cavities of female metasoma T7 present, glabrous save for few, long setae. Female posteroventral margin of T6–T7 distinctly sinuate. Terebrum and hypopygium (in lateral view) straight, pointing posteriorly.

*Ovipositor*. First valvula of ovipositor narrowing gradually, not broadened apically, serrate at tip. Ovipositor clip absent.

**Figure 17.**
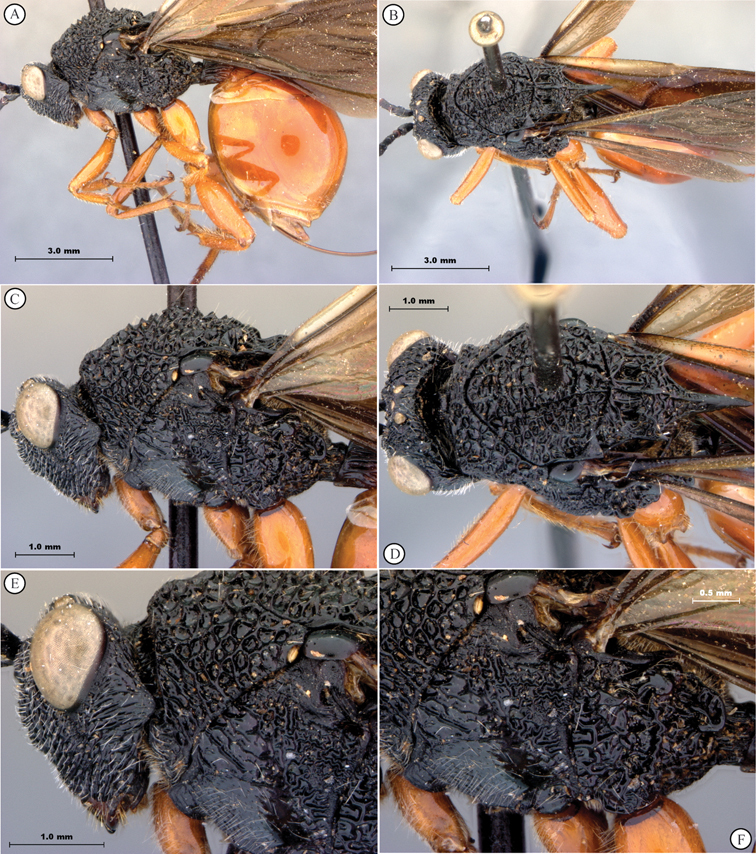
*Oberthuerella lenticularis* Saussure **A** lateral habitus **B** habitus, dorsal view **C** head and mesosoma, lateral view **D** mesosoma, dorsal view **E** head and pronotum, lateral view **F** meso- and metapleurae.

**Figure 18.**
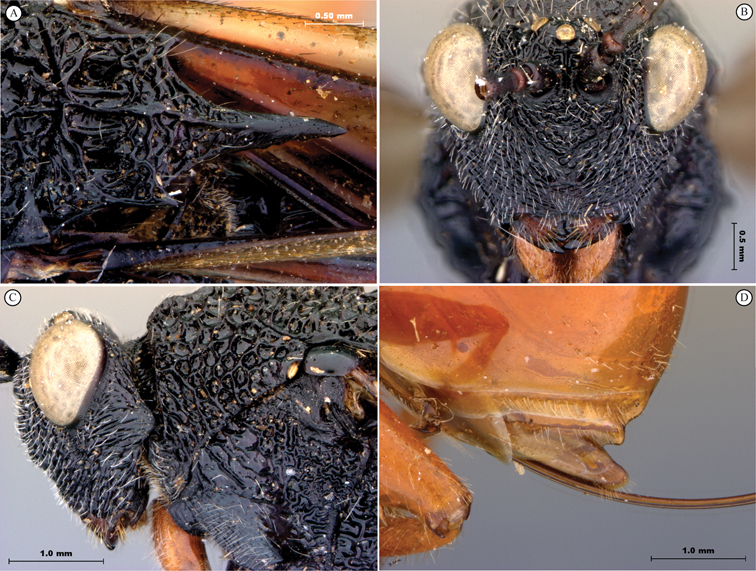
*Oberthuerella lenticularis* Saussure **A** scutellum, dorsal view **B** head, anterior view **C** head and pronotum, lateral view **D** posterior margin or metasoma, lateral view.

##### Diagnosis.

Distinguished from other species of *Oberthuerella* by the extremely short vertical keel on the face, not extending beyond the toruli; this feature is also shared with *Oberthuerella triformis*, but in this latter species, the scutellar spine is remarkably short, whereas in *Oberthuerella lenticularis*, the scutellar spine is roughly equal in length to the petiole. Finally, the pronotal and mesoscutal sculpture in *Oberthuerella lenticularis* is deeply foveate, so much so that the general appearance of this species is craggy.

##### Note.

This name was originally published as an image with an associated name in a larger volume on Madagascar (Saussure, 1890). Article 12.2.7 of IZCN (2000) stipulates the image of *Oberthuerella lenticularis* satisfies indication of a new genus and species, even though no description nor holotype was provided. Thus, the genus and species names are available, and the illustration of Saussure (1890) serves as the holotype.

##### Distribution.

Ivory Coast, Madagascar, Malawi, South Africa. **Link to Distribution Map.** [http://hol.osu.edu/map-full.html?id=181556]

##### Material examined.

*Other material*: (4 females) **AFRICA:** no date, J. Wahlberg (1 female, MCZC 0011 (MCZC). **IVORY COAST:** Adiopodoume [05°20'28"N, 04°07'54"W] , 8.6.49, H.B Jover (1 female, MNHN).**MADAGASCAR:** Toamasina Auto. Prov., Rogez, XI-1931, A. Seyrig (1 female, USNM ENT 00764775 (USNM); Region du Sud-Est, Vallee du Fanjahira Isaka, [24°54'42"S, 046°53'08"E], Ch. Alluaud, Dec 1901, *Oberthuerella lenticularis* Sauss. Det Weld 1931; *Oberthuerella lenticularis* S. Female, det J. Quinlan, 1978 (1 female MNHN). (Further material listed in Quinlan, 1979)

#### Oberthuerella longicaudata

Benoit

urn:lsid:biosci.ohio-state.edu:osuc_concepts:181557

Morphbank accession: 704777–704790

http://www.waspweb.org/Cynipoidea/Liopteridae/Oberthuerellinae/Oberthuerella/Oberthuerella_longicaudata.htm

http://species-id.net/wiki/Oberthuerella_longicaudata

Figures 19 –20

Oberthuerella longicaudata Benoit, 1955: 291.

##### Description.

Coloration of head and mesosoma black to dark brown; metasoma and legs yellow-orange. Sculpture on vertex, lateral surface of pronotum and mesoscutum present, deeply foveate laterally on head, pronotum; deeply horizontally striate on mesoscutum.

*Head*. Broadly triangular, in anterior view. Pubescence on head present, sparse setae scattered over head. Sculpture along lateral margin of occiput absent. Gena (measured from compound eye to posterolateral margin of head) short, ratio of length of gena to length of compound eye in dorsal view < 0.3, in dorsal view. Sculpture of gena deeply striate with remnants of fovea. Lateral margin of occiput defined by distinctly angled, raised, sharp carina. Occiput (except extreme lateral margin) smooth. Ocelli small, ratio of maximum diameter of a lateral ocellus to shortest distance between lateral ocelli 0.2–0.4. Anterior ocellus close to posterior ocelli, posterior margin of anterior ocellus behind or subcontiguous with a transverse line running through anterior margins of posterior ocelli. Relative position of toruli close to ocelli, ratio of vertical distance between inner margin of torulus and ventral margin of clypeus to vertical distance between anterior ocellus and torulus < 2.0. Median keel of face present, extending to middle of face, not reaching clypeus. Vertical carina adjacent to ventral margin of torulus absent. Facial sculpture almost entirely foveate, with smooth, narrow, dorso-ventral triangular area along midline. Facial impression absent, face flat. Antennal scrobe absent. Anterior tentorial pits large. Vertical delineations on lower face absent. Ventral clypeal margin laterally, close to anterior mandibular articulation, distinctly angled. Ventral clypeal margin medially emarginate. Clypeus foveate-punctate; horizontally striate. Malar space adjacent to anterior articulation of mandible evenly rounded, striate-foveate. Malar sulcus absent. Compound eye close to posterior ocellus, ratio of distance between compound eye and posterior mandibular articulation to distance between posterior ocellus and compound eye > 1.2. Compound eye, in dorsal view, distinctly protruding from the surface of the head, particularly laterally. Pubescence on compound eye absent. Orbital furrows absent. Lateral frontal carina of face absent. Dorsal aspect of vertex deeply foveate. Posterior aspect of vertex foveate. Hair punctures on lateral aspect of vertex absent. Posterior surface of head almost flat, not deeply impressed.

*Antenna*. Articulation between flagellomeres in antenna connate with articles broadly joined. Female F1 as long as F2. Flagellomeres of female antenna cylindrical, not widened towards apex, non-clavate. Placoidal sensilla absent.

*Pronotum*. Macrosculpture on lateral surface of pronotum present, dorsomedially foveate, laterally foveate-costate. Pubescence on lateral surface of pronotum present, sparse, composed of few short hairs. Carinae extending posteriorly from lateral margin of pronotal plate absent. Lateral pronotal carina present. Pronotal crest absent. Dorsal margin of pronotal plate (in anterior view) rounded. Lateral margin of pronotal plate defined all the way to the dorsal margin of the pronotum. Pronotal plate wide, almost as wide as head.

*Mesoscutum*. Mesoscutal surface convex, evenly curved. Sculpture on mesoscutum present, transversely costate with dorsally projected serrations. Notaulus present, marked by series of deep subcontiguous pits of uniform width. Median mesoscutal carina absent. Anterior admedial lines present, with adjacent cuticular surface horizontally striate. Parascutal carina distinctly sinuate, posteriorly ending in posteroventrally directed projection.

*Mesopleuron*. Horizontally strigulate, with striae converging on rugose sculpture on posterior one-third of sclerite. Subpleuron entirely smooth, glabrous. Lower mesopleuron medially smooth, glabrous; costate laterally, ventrally. Epicnemial carina present on ventral half of mesopleuron; shagreened, ventrally bulbous near mesosternum. Lateroventral mesopleural carina present, marking abrupt change of slope of mesopectus. Mesopleural triangle absent. Subalar pit large and well defined, lying in posterior end of subalar groove. Speculum present, gently rugose anteriorly, smooth posteriorly. Mesopleural carina absent.

*Scutellum*. Dorsal surface of scutellum foveate-areolate. Circumscutellar carina absent. Posterior margin of axillula marked by distinct ledge, axillula distinctly impressed adjacent to ledge. Lateroventral margin of scutellum posterior to auricula entirely smooth. Dorsoposterior part of scutellum produced posteriorly into sharp spine, less than 1.0× length of petiole. Dorsal part of scutellum entirely foveate. Scutellar plate absent. Scutellar foveae uncertain, present, three. Longitudinal scutellar carinae absent. Single longitudinal carina separating scutellar foveae absent. Posterolateral margin of scutellum drawn out into distinct protuberance. Lateral bar distinctly foveate, wide.

*Metapectal-propodeal complex*. Metapectal cavity anterodorsal to metacoxal base present, ill-defined. Anterior margin of metapectal-propodeal complex separated from mesopleuron by deep, broad, uninterrupted marginal impression. Posteroventral corner of metapleuron (in lateral view) rounded, not drawn out posteriorly. Anterior impression of metepimeron present, narrow, linear impression, not broadened ventrally. Posterior margin of metepimeron distinct, separating metepimeron from propodeum. Subalar area abruptly broadened anteriorly, with an indicated longitudinal division. Calyptra present, blunt, lobe-like, polished. Dorsellum present, two strong medial fovea, laterally strongly excavated with fine pubescence in lateral depressions. Anterior impression of metepisternum, immediately beneath anterior end of metapleural carina, present, small and narrow. Pubescence consisting of few scattered hairs on posterior part of metapleuron and lateral part of propodeum. Propodeal spurs present, foveate. Lateral propodeal carinae present, not reaching scutellum. Ventral end of lateral propodeal carina reaching nucha, carinae separated from each other. Inter propodeal carinae space lightly setose, foveate. Petiolar foramen removed from metacoxae, directed posteriorly. Horizontal carina running anteriorly from lateral propodeal carina present. Lateral propodeal carina distinctly angled. Calyptra, in lateral view, elongate. Propodeum relatively short, not drawn out posteriorly. Calyptra, in posterior view, dorsoventrally elongate.

*Legs*. Pubescence posterolaterally on metacoxa sparse to moderately dense, confined dense hair patch absent. Microsculpture on hind coxa absent. Longitudinal carina on the posterior surface of metatibia absent. Metafemoral spine present, elongate, extending distally as low keel along ventral femoral margin. Distal mesotibial spurs equal in length to medial spurs. Distal metatibial spurs equal in length to medial spurs. Ratio of first metatibial segment to remaining 4 segments greater than 1.0.

*Forewing*. Pubescence of forewing absent on basal half of wing, sparse distally. Apical margin of female forewing rounded. Rs+M of forewing tubular. Mesal end of Rs+M vein situated closer to posterior margin of forewing, directed towards posterior end of basalis. Vein R1 tubular along at least basal part of anterior margin of marginal cell. Basal abscissa of R1 (the abscissa between 2r and the forewing margin) of forewing as broad as adjacent wing veins. Forewing entirely infuscate. Marginal cell of forewing membranous, similar to other wing cells. Areolet absent. Hair fringe along apical margin of forewing absent.

*Petiole*. Stout, 1.5–1.75× wider than long. Surface of petiole longitudinally costate, ventral keel absent. Posterior part of female petiole not abruptly widened. Ventral flange of annulus of female petiole absent.

*Metasoma*. Setal band (hairy ring) at base of tergum 3 absent, base of metasoma glabrous. Tergum 3 distinctly smaller than tergum 4. Posterior margin of tergum 3 slightly arcuate. Posterior margin of tergum 4 arcuate. In lateral view, sternum 3 exposed, ventral border of T2–T7 visible. Sculpture on metasomal terga present, finely punctate laterally, dorsally; posteriorly with large setal pits. Syntergum absent, all postpetiolar terga free. Annulus absent. Peg-like setae on T6–T7 present. Posteroventral cavities of female metasoma T7 present, setose. Female posteroventral margin of T6–T7 distinctly sinuate. Terebrum and hypopygium (in lateral view) straight, pointing posteriorly.

*Ovipositor*. First valvula of ovipositor narrowing gradually, not broadened apically, smooth at tip. Ovipositor clip absent.

**Figure 19.**
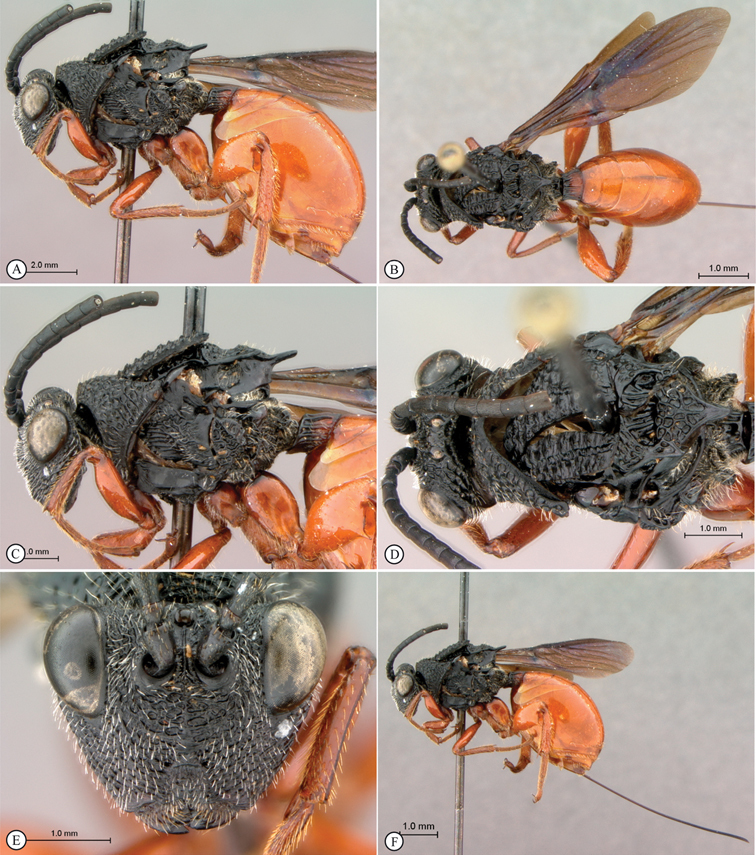
*Oberthuerella longicaudata* Benoit, holotype **A** lateral habitus **B** habitus, dorsal view **C** head and mesosoma, lateral view **D** mesosoma, dorsal view **E** head, anterior view **F** lateral habitus.

**Figure 20.**
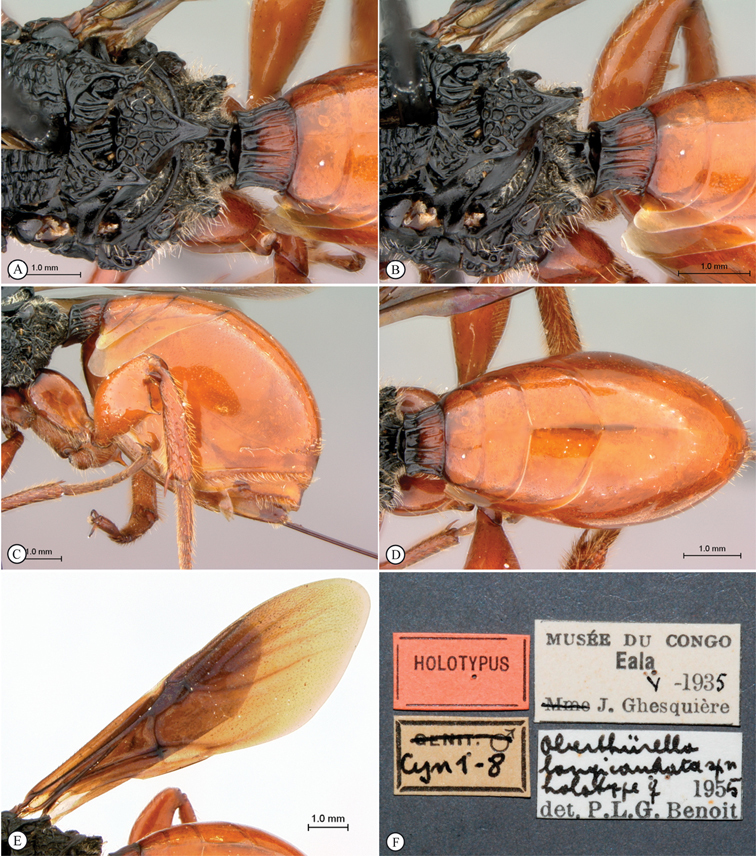
*Oberthuerella longicaudata* Benoit, holotype **A** scutellum, dorsal view **B** scutellum, dorsolateral view **C** metasoma, lateral view **D** metasoma, dorsal view **E** fore and hind wings **F** labels.

##### Diagnosis.

This species can be differentiated from other species of *Oberthuerella* by the morphology of the lower face: the median keel is gradually but distinctly broadened as it extends to the dorsal clypeal margin, resulting in a raised, triangular area; in other *Oberthuerella*, there is either no median keel (does not reach clypeal margin), or the keel is consistently narrow along its length.

##### Distribution.

Democratic Republic of Congo. **Link to Distribution Map.** [http://hol.osu.edu/map-full.html?id=181557]

##### Material examined.

Holotype, female: **DEMOCRATIC REPUBLIC OF THE CONGO:** Eala, V-1935, J. Ghesquière, Mus. Cong. Cyn1-8 (deposited in MRAC).

#### Oberthuerella longispinosa

Benoit

urn:lsid:biosci.ohio-state.edu:osuc_concepts:181558

Morphbank accession: 704791–704802

http://www.waspweb.org/Cynipoidea/Liopteridae/Oberthuerellinae/Oberthuerella/Oberthuerella_longispinosa.htm

http://species-id.net/wiki/Oberthuerella_longispinosa

Figures 21 –22

Oberthuerella longispinosa Benoit, 1955: 290.

##### Description.

Coloration of head and mesosoma black to dark brown; metasoma yellow-orange; legs reddish brown. Sculpture on vertex, lateral surface of pronotum and mesoscutum present, deeply foveate laterally on head, pronotum; deeply horizontally striate on mesoscutum.

*Head*. Broadly triangular, in anterior view. Pubescence on head present, sparse setae scattered over head. Sculpture along lateral margin of occiput absent. Gena (measured from compound eye to posterolateral margin of head) short, ratio of length of gena to length of compound eye in dorsal view < 0.3, in dorsal view. Sculpture of gena deeply striate with remnants of fovea. Lateral margin of occiput defined by distinctly angled, raised, sharp carina. Occiput (except extreme lateral margin) smooth. Ocelli small, ratio of maximum diameter of a lateral ocellus to shortest distance between lateral ocelli 0.2–0.4. Anterior ocellus close to posterior ocelli, posterior margin of anterior ocellus behind or subcontiguous with a transverse line running through anterior margins of posterior ocelli. Relative position of toruli close to ocelli, ratio of vertical distance between inner margin of torulus and ventral margin of clypeus to vertical distance between anterior ocellus and torulus < 2.0. Median keel of face present, extending to middle of face, not reaching clypeus. Vertical carina adjacent to ventral margin of torulus absent. Facial sculpture almost entirely foveate, slightly horizontally striate along median keel. Facial impression absent, face flat. Antennal scrobe absent. Anterior tentorial pits large. Vertical delineations on lower face absent. Ventral clypeal margin laterally, close to anterior mandibular articulation, distinctly angled. Ventral clypeal margin medially emarginate. Clypeus foveate-punctate; horizontally striate. Malar space adjacent to anterior articulation of mandible evenly rounded, foveate. Malar sulcus absent. Compound eye close to posterior ocellus, ratio of distance between compound eye and posterior mandibular articulation to distance between posterior ocellus and compound eye > 1.2. Compound eye, in dorsal view, distinctly protruding from the surface of the head, particularly laterally. Pubescence on compound eye absent. Orbital furrows absent. Lateral frontal carina of face absent. Dorsal aspect of vertex deeply foveate. Posterior aspect of vertex foveate. Hair punctures on lateral aspect of vertex absent. Posterior surface of head almost flat, not deeply impressed.

*Labial-maxillary complex*. Apical segment of maxillary palp with pubescence, consisting only of erect setae. Apical seta on apical segment of maxillary palp shorter than twice length of second longest apical seta. Erect setae medially on apical segment of maxillary palp present.

*Antenna*. Articulation between flagellomeres in antenna connate with articles broadly joined. Male antenna composed of 12 flagellomeres. Placoidal sensilla absent. Second flagellomere of male antenna cylindrical. Length of second flagellomere of male antenna longer than first flagellomere.

*Pronotum*. Macrosculpture on lateral surface of pronotum present, dorsomedially foveate, laterally foveate-costate. Pubescence on lateral surface of pronotum present, sparse, composed of few short hairs. Carinae extending posteriorly from lateral margin of pronotal plate absent. Lateral pronotal carina present. Pronotal crest absent. Dorsal margin of pronotal plate (in anterior view) rounded. Lateral margin of pronotal plate defined all the way to the dorsal margin of the pronotum. Pronotal plate wide, almost as wide as head.

*Mesoscutum*. Mesoscutal surface convex, evenly curved. Sculpture on mesoscutum present, foveate-punctate, with remnants of transverse costae. Notaulus present, marked by deep furrows, slightly increasing in width posteriorly. Median mesoscutal carina absent. Anterior admedial lines present, flat, indistinct, with adjacent cuticular surface foveate. Parascutal carina distinctly sinuate, posteriorly ending in posteroventrally directed projection.

*Mesopleuron*. Horizontally strigulate, with striae converging on remnant fovea along posterior margin of sclerite. Subpleuron anteriorly smooth, polished, posteriorly with remnants of fovea. Lower mesopleuron medially smooth, glabrous; costate laterally, ventrally. Epicnemial carina present on ventral half of mesopleuron; shagreened, of even width throughout. Lateroventral mesopleural carina present, marking abrupt change of slope of mesopectus. Mesopleural triangle absent. Subalar pit large and well defined, lying in posterior end of subalar groove. Speculum present, smooth. Mesopleural carina absent.

*Scutellum*. Dorsal surface of scutellum foveate-areolate. Circumscutellar carina absent. Posterior margin of axillula marked by distinct ledge, axillula distinctly impressed adjacent to ledge. Lateroventral margin of scutellum posterior to auricula smooth, becoming dorsoventrally striate posteriorly. Dorsoposterior part of scutellum produced posteriorly into sharp spine, greater than 1.0× length of petiole. Dorsal part of scutellum entirely foveate. Scutellar plate absent. Scutellar foveae present, three, with lateral foveal bissected by longitudinal carina, resulting in five longitudinally elongate subfovea. Longitudinal scutellar carinae absent. Single longitudinal carina separating scutellar foveae absent. Posterolateral margin of scutellum drawn out into distinct protuberance. Lateral bar with strong strigate sculpture, narrow.

*Metapectal-propodeal complex*. Metapectal cavity anterodorsal to metacoxal base present, ill-defined. Anterior margin of metapectal-propodeal complex separated from mesopleuron by deep, broad, uninterrupted marginal impression. Posteroventral corner of metapleuron (in lateral view) rounded, not drawn out posteriorly. Anterior impression of metepimeron present, narrow, linear impression, not broadened ventrally. Posterior margin of metepimeron distinct, separating metepimeron from propodeum. Subalar area abruptly broadened anteriorly, with an indicated longitudinal division. Calyptra present, blunt, lobe-like, polished posteriorly with setiferous punctures anteriorly. Dorsellum present, two strong medial fovea, laterally strongly excavated with fine pubescence in lateral depressions. Anterior impression of metepisternum, immediately beneath anterior end of metapleural carina, present, small and narrow. Pubescence consisting of few scattered hairs on posterior part of metapleuron and lateral part of propodeum. Propodeal spurs present, foveate. Lateral propodeal carinae present, not reaching scutellum. Ventral end of lateral propodeal carina reaching nucha, carinae separated from each other. Inter propodeal carinae space lightly setose, foveate. Petiolar foramen removed from metacoxae, directed posteriorly. Horizontal carina running anteriorly from lateral propodeal carina present. Lateral propodeal carina straight, sub-parallel. Calyptra, in lateral view, elongate. Propodeum relatively short, not drawn out posteriorly. Calyptra, in posterior view, dorsoventrally elongate.

*Legs*. Pubescence posterolaterally on metacoxa sparse to moderately dense, confined dense hair patch absent. Microsculpture on hind coxa absent. Longitudinal carina on the posterior surface of metatibia absent. Metafemoral spine present, elongate, extending distally as low keel along ventral femoral margin. Ratio of first metatibial segment to remaining 4 segments equal to 1.0.

*Forewings*. Pubescence of forewing present, long, dense on most of surface. Apical margin of female forewing rounded. Rs+M of forewing tubular. Mesal end of Rs+M vein situated closer to posterior margin of Forewing, directed towards posterior end of basalis. Vein R1 tubular along at least basal part of anterior margin of marginal cell. Basal abscissa of R1 (the abscissa between 2r and the Forewing margin) of forewing as broad as adjacent wing veins. Forewing entirely infuscate. Marginal cell of forewing membranous, similar to other wing cells. Areolet absent. Hair fringe along apical margin of forewing absent.

*Petiole*. Slightly elongate, 1.5–2× longer than wide. Surface of petiole longitudinally costate, ventral keel absent.

*Metasoma*. Setal band (hairy ring) at base of tergum 3 absent, base of metasoma glabrous. Tergum 3 distinctly smaller than tergum 4. Posterior margin of tergum 3 smoothly rounded. Posterior margin of tergum 4 evenly rounded. In lateral view, sternum 3 exposed, ventral border of T2–T7 visible. Sculpture on metasomal terga present, finely punctate laterally, dorsally; posteriorly with large setal pits. Syntergum absent, all postpetiolar terga free. Annulus absent. Peg-like setae on T6–T7 absent. Posteroventral cavities of female metasoma T7 absent.

**Figure 21.**
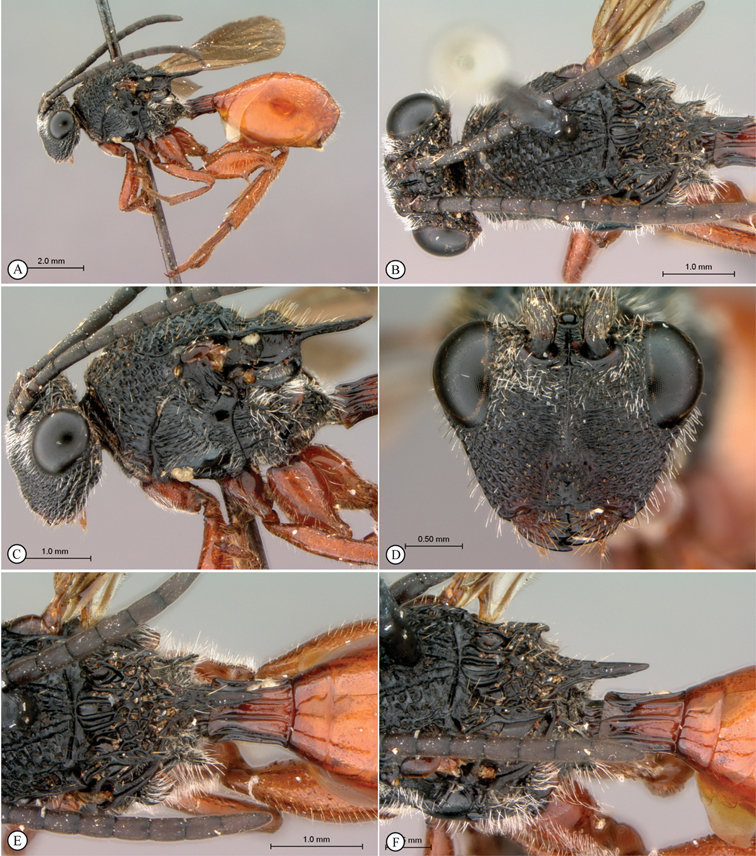
*Oberthuerella longispinosa* Benoit, holotype **A** lateral habitus **B** head and mesosoma, dorsal view **C** head and mesosoma, lateral view **D** head, anterior view **E** scutellum and petiole, dorsal view **F** scutellum and petiole, dorsolateral view.

**Figure 22.**
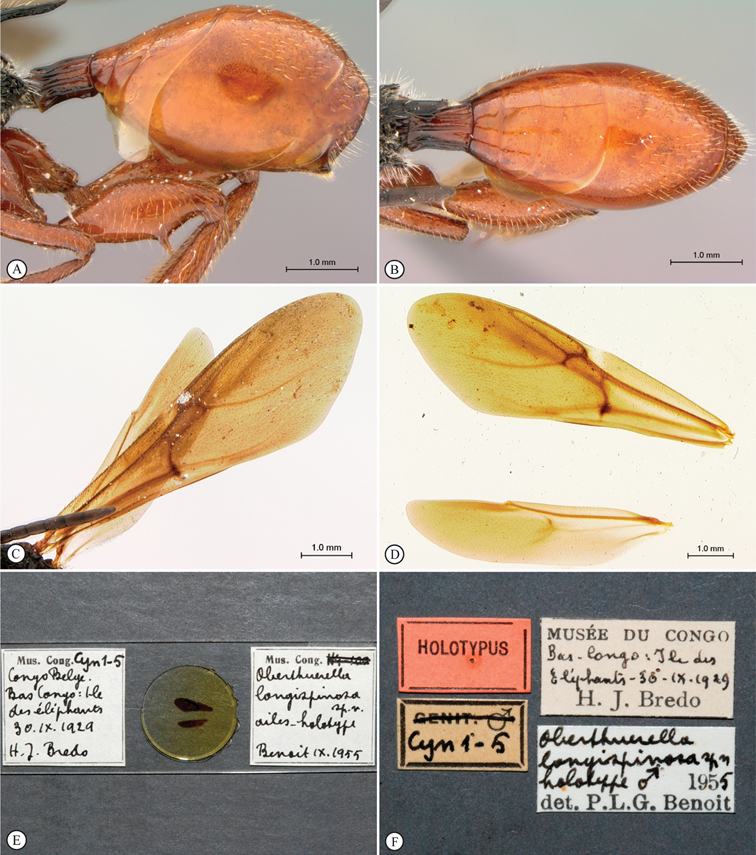
*Oberthuerella longispinosa* Benoit, holotype **A** metasoma, lateral view **B** metasoma, dorsal view **C** fore and hind wings **D** fore and hind wings **E** fore and hindwing slide overview **F** labels.

##### Diagnosis.

This species has the most complete and consistently narrow median keel of the face; in other species, either the keel is distinctly broadened into a raised triangular area (*Oberthuerella longicaudata*) or the keel becomes indistinct before meeting the clypeal margin (*Oberthuerella crassicornis*). This species resembles *Oberthuerella abscinda* and *Oberthuerella crassicornis*, and can be separated from the former by having yellow/orange legs (*Oberthuerella abscinda* with dark brown/black legs), and the latter by the morphology of the median keel of the face mentioned above.

##### Distribution.

Democratic Republic of Congo; Gabon; Ivory Coast; Malawi. **Link to Distribution Map.** [http://hol.osu.edu/map-full.html?id=181558]

##### Material examined.

Holotype, male: **DEMOCRATIC REPUBLIC OF CONGO:** Pool Dépt., Éléphants Island, 30.IX.1929, H. J. Bredo, Mus. Cong. Cyn1-5 (deposited in MRAC). (Further material listed in Quinlan, 1979)

#### Oberthuerella nigra

Kieffer

Oberthuerella nigra Kieffer, 1910a: 110. Holotype male previously in (ZMHB). Now missing (Ronquist, 1995). Not examined.

##### Distribution.

Equatorial Guinea.

#### Oberthuerella nigrescens

Benoit

urn:lsid:biosci.ohio-state.edu:osuc_concepts:181560

Morphbank accession: 704803–704818

http://www.waspweb.org/Cynipoidea/Liopteridae/Oberthuerellinae/Oberthuerella/Oberthuerella_nigrescens.htm

http://species-id.net/wiki/Oberthuerella_nigrescens

**Figure 23.**
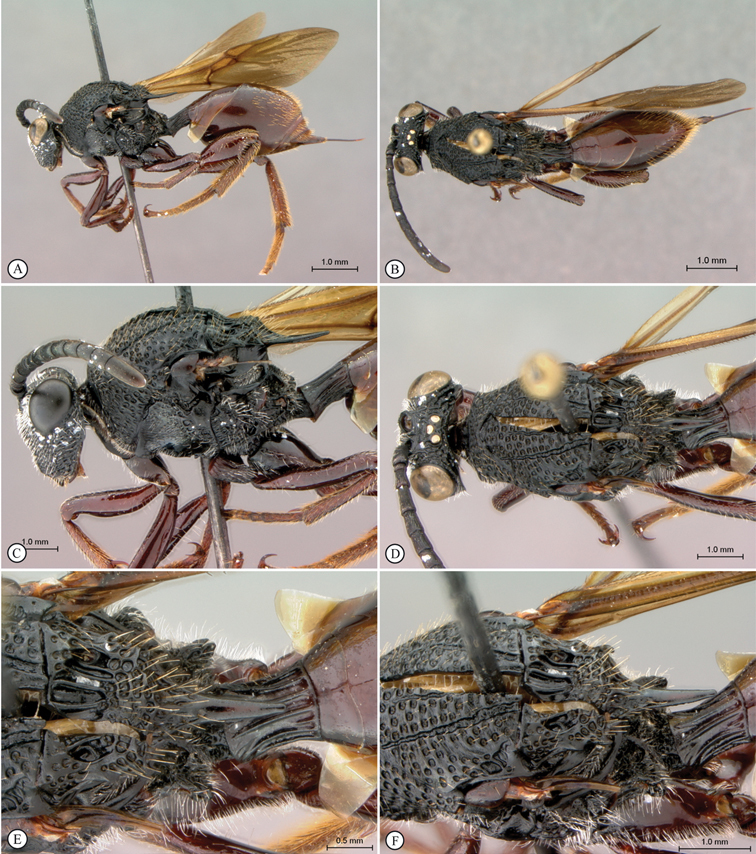
*Oberthuerella nigrescens* Benoit, holotype **A** lateral habitus **B** dorsal habitus **C** head and mesosoma, lateral view **D** head and mesosoma, dorsal view **E** scutellum and petiole, dorsal view **F** scutellum and petiole, dorsolateral view.

Figures 23 [Fig F24] –25

Oberthuerella nigrescens Benoit, 1955: 288

##### Description.

Coloration of head, mesosoma, and metasoma black to dark brown; legs reddish brown. Sculpture on vertex, lateral surface of pronotum and mesoscutum present, deeply foveate laterally on head, pronotum, mesoscutum.

*Head*. Broadly triangular, in anterior view. Pubescence on head present, dense setae covering head. Sculpture along lateral margin of occiput absent. Gena (measured from compound eye to posterolateral margin of head) short, ratio of length of gena to length of compound eye in dorsal view < 0.3, in dorsal view. Sculpture of gena present, with distinct fovea. Lateral margin of occiput defined by distinctly angled, raised, sharp carina. Occiput (except extreme lateral margin) smooth. Ocelli small, ratio of maximum diameter of a lateral ocellus to shortest distance between lateral ocelli 0.2–0.4. Anterior ocellus close to posterior ocelli, posterior margin of anterior ocellus behind or subcontiguous with a transverse line running through anterior margins of posterior ocelli. Relative position of toruli close to ocelli, ratio of vertical distance between inner margin of torulus and ventral margin of clypeus to vertical distance between anterior ocellus and torulus < 2.0. Median keel of face present, short, not extending beyond toruli. Vertical carina adjacent to ventral margin of torulus absent. Facial sculpture almost entirely foveate, with smooth, narrow, dorso-ventral triangular area along midline. Facial impression absent, face flat. Antennal scrobe absent. Anterior tentorial pits large. Vertical delineations on lower face absent. Ventral clypeal margin laterally, close to anterior mandibular articulation, distinctly angled. Ventral clypeal margin medially emarginate. Clypeus horizontally striate. Malar space adjacent to anterior articulation of mandible evenly rounded, foveate. Malar sulcus absent. Compound eye close to posterior ocellus, ratio of distance between compound eye and posterior mandibular articulation to distance between posterior ocellus and compound eye > 1.2. Compound eye, in dorsal view, distinctly protruding from the surface of the head, particularly laterally. Pubescence on Compound eye absent. Orbital furrows absent. Lateral frontal carina of face absent. Dorsal aspect of vertex deeply foveate. Posterior aspect of vertex foveate. Hair punctures on lateral aspect of vertex absent. Posterior surface of head almost flat, not deeply impressed.

*Antenna*. Articulation between flagellomeres in antenna connate with articles broadly joined. Female antenna composed of 11 flagellomeres. Female F1 shorter than F2; black. Flagellomeres of female antenna cylindrical, not widened towards apex, non-clavate. Placoidal sensilla absent. Distal flagellomeres of female antenna not conspicuously enlarged compared to proximal flagellomeres.

Macrosculpture on lateral surface of pronotum present, dorsomedially foveate, laterally foveate-costate. Pubescence on lateral surface of pronotum present, sparse, consisting of few short hairs. Anterior flange of pronotal plate distinctly protruding anteriorly, smooth. Carinae extending posteriorly from lateral margin of pronotal plate absent. Lateral pronotal carina present. Pronotal crest absent. Dorsal margin of pronotal plate (in anterior view) rounded. Lateral margin of pronotal plate defined all the way to the dorsal margin of the pronotum. Pronotal plate wide, almost as wide as head.

*Mesoscutum*. Mesoscutal surface convex, evenly curved. Sculpture on mesoscutum present, foveate-punctate, with remnants of transverse costae. Notaulus present, marked by deep furrows, slightly increasing in width posteriorly. Median mesoscutal carina absent. Anterior admedial lines present, flat, indistinct, with adjacent cuticular surface foveate. Median mesoscutal impression present, long, reaching over 1/2 length of mesoscutum. Parascutal carina distinctly sinuate, posteriorly ending in posteroventrally directed projection.

*Mesopleuron*. Horizontally strigulate, with striae converging on remnant fovea along posterior margin of sclerite. Subpleuron entirely smooth with few long white setae along ventral, posterior margins. Lower mesopleuron medially smooth, setose; costate laterally, ventrally. Epicnemial carina present on ventral half of mesopleuron; shagreened, ventrally bulbous near mesosternum. Lateroventral mesopleural carina present, marking abrupt change of slope of mesopectus. Mesopleural triangle absent. Subalar pit large and well defined, lying in posterior end of subalar groove. Speculum present, smooth anteriorly, microcarinate posteriorly. Mesopleural carina absent.

*Scutellum*. Dorsal surface of scutellum foveate-areolate. Circumscutellar carina absent. Posterior margin of axillula marked by distinct ledge, axillula distinctly impressed adjacent to ledge. Lateroventral margin of scutellum posterior to auricula entirely smooth. Dorsoposterior part of scutellum produced posteriorly into sharp spine, greater than 1.0× length of petiole. Dorsal part of scutellum entirely foveate. Scutellar plate absent. Scutellar foveae present, three, each lateral fovea with two longitudinal divisions, central fovea smooth, resulting in transverse row of 7 longitudinally elongate subfovea. Longitudinal scutellar carinae absent. Single longitudinal carina separating scutellar foveae absent. Posterolateral margin of scutellum rounded. Lateral bar distinctly foveate, wide.

*Metapectal-propodeal complex*. Metapectal cavity anterodorsal to metacoxal base present, ill-defined. Anterior margin of metapectal-propodeal complex separated from mesopleuron by deep, broad, uninterrupted marginal impression. Posteroventral corner of metapleuron (in lateral view) rounded, not drawn out posteriorly. Anterior impression of metepimeron present, narrow, linear impression, not broadened ventrally. Posterior margin of metepimeron distinct, separating metepimeron from propodeum. Subalar area slightly broadened anteriorly, without longitudinal division indicated. Calyptra present, blunt, lobe-like, polished posteriorly with setiferous punctures anteriorly. Dorsellum present, two strong medial fovea, laterally strongly excavated with fine pubescence in lateral depressions. Anterior impression of metepisternum, immediately beneath anterior end of metapleural carina, absent. Pubescence consisting of few scattered hairs on posterior part of metapleuron and lateral part of propodeum. Propodeal spurs present, foveate. Lateral propodeal carinae present, not reaching scutellum. Ventral end of lateral propodeal carina reaching nucha, carinae separated from each other. Inter propodeal carinae space lightly setose, foveate. Petiolar foramen removed from metacoxae, directed posteriorly. Horizontal carina running anteriorly from lateral propodeal carina present. Lateral propodeal carina straight, sub-parallel. Calyptra, in lateral view, elongate. Propodeum relatively short, not drawn out posteriorly. Calyptra, in posterior view, dorsoventrally elongate.

*Legs*. Pubescence posterolaterally on metacoxa sparse to moderately dense, confined dense hair patch absent. Microsculpture on hind coxa absent. Longitudinal carina on the posterior surface of metatibia absent. Metafemoral spine present, elongate, extending distally as low keel along ventral femoral margin. Ratio of first metatibial segment to remaining 4 segments greater than 1.0.

*Forewing*. Pubescence of forewing absent on basal half of wing, sparse distally. Apical margin of female forewing rounded. Rs+M of forewing tubular. Mesal end of Rs+M vein situated closer to posterior margin of forewing, directed towards posterior end of basalis. Vein R1 tubular along at least basal part of anterior margin of marginal cell. Basal abscissa of R1 (the abscissa between 2r and the forewing margin) of forewing as broad as adjacent wing veins. Forewing entirely lightly infuscate. Marginal cell of forewing membranous, similar to other wing cells. Areolet present, complete. Hair fringe along apical margin of forewing absent.

*Petiole*. Slightly elongate, 1.5–2× longer than wide. Surface of petiole longitudinally costate, ventral keel absent. Posterior part of female petiole not abruptly widened. Ventral flange of annulus of female petiole absent.

*Metasoma*. Setal band (hairy ring) at base of tergum 3 absent, base of metasoma glabrous. Tergum 3 distinctly smaller than tergum 4. Posterior margin of tergum 3 slightly but distinctly concave. Posterior margin of tergum 4 arcuate. In lateral view, sternum 3 exposed, ventral border of T2–T7 visible. Sculpture on metasomal terga present, dorsally finely punctate, posteriorly with distinct bands of setiferous pits. Syntergum absent, all postpetiolar terga free. Annulus absent. Peg-like setae on T6–T7 absent. Posteroventral cavities of female metasoma T7 present, setose. Female posteroventral margin of T6–T7 gently sinuate. Terebrum and hypopygium (in lateral view) straight, pointing posteriorly.

*Ovipositor*. First valvula of ovipositor narrowing gradually, not broadened apically, smooth at tip. Ovipositor clip absent.

**Figure 24. F24:**
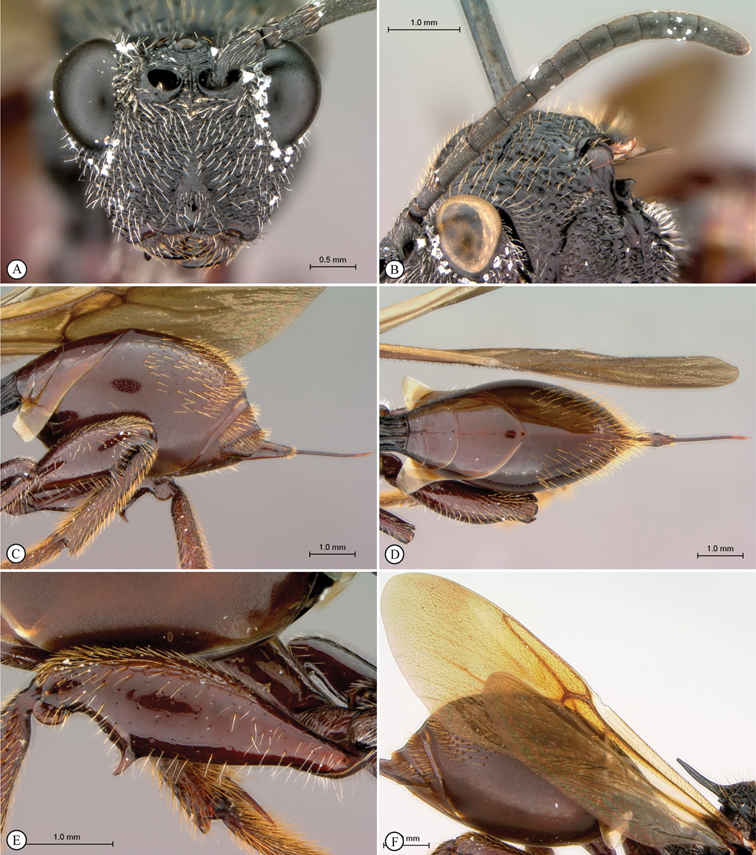
*Oberthuerella nigrescens* Benoit, holotype **A** head, anterior view **B** antenna **C** metasoma, lateral view **D** metasoma, dorsal view **E** hind femur **F** fore and hind wing.

**Figure 25.**
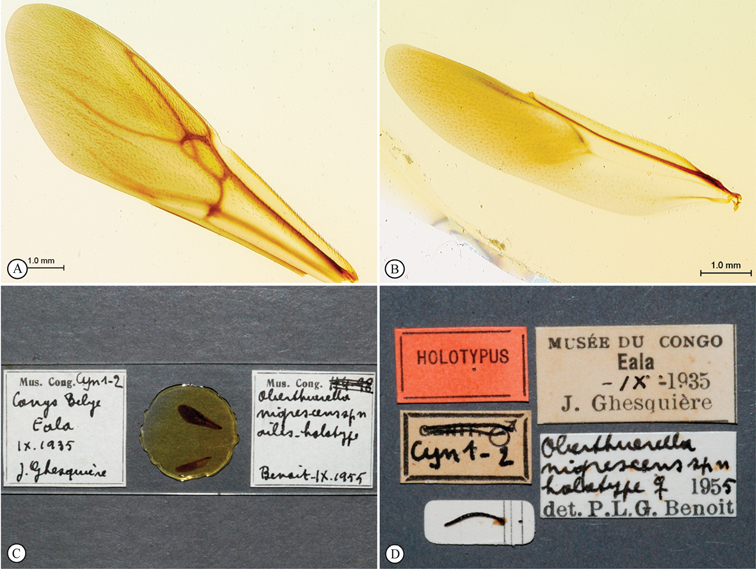
*Oberthuerella nigrescens* Benoit, holotype **A** forewing **B** hindwing **C** fore and hind wing slide overview **D** labels.

##### Diagnosis.

Distinguished from other species of *Oberthuerella* by having the head, mesosoma and metasoma all dark brown/black (in other species, the metasoma is yellow/orange); the only other species that shares this trait is *Oberthuerella kibalensis*, and this species can be differentiated based on slight color differences in setae on the hind legs and metasoma, where in *Oberthuerella kibalensis* the setae are silvery-white, and in *Oberthuerella nigrescens*, the setae are a golden-yellow. Also, in *Oberthuerella kibalensis*, the lateral aspect of the pronotum is deeply costate, and in *Oberthuerella nigrescens*, the same is foveate with remnant traces of costae.

##### Distribution.

Democratic Republic of Congo. **Link to Distribution Map.** [http://hol.osu.edu/map-full.html?id=181560]

##### Material examined.

Holotype, female: **DEMOCRATIC REPUBLIC OF THE CONGO:** Eala, IX-1935, J. Ghesquière, Mus. Cong. Cyn1-2 (deposited in MRAC).

#### Oberthuerella pardolatus

Buffington & van Noort sp. n.

urn:lsid:zoobank.org:act:526BE663-C58A-4227-8465-8C02AD1DA58E

urn:lsid:biosci.ohio-state.edu:osuc_concepts:300218

Morphbank accession: 704646–704655

http://www.waspweb.org/Cynipoidea/Liopteridae/Oberthuerellinae/Oberthuerella/Oberthuerella_pardolatus.htm

http://species-id.net/wiki/Oberthuerella_pardolatus

Figures 26 –27

##### Description.

Coloration of head, mesosoma, and metasoma black to dark brown; legs reddish brown. Sculpture on vertex, lateral surface of pronotum and mesoscutum present, deeply foveate laterally on head; pronotum, mesoscutum striate-foveate.

*Head*. Broadly triangular, in anterior view. Pubescence on head present, sparse setae scattered over head. Sculpture along lateral margin of occiput absent. Gena (measured from compound eye to posterolateral margin of head) short, ratio of length of gena to length of compound eye in dorsal view < 0.3, in dorsal view. Sculpture of gena present, with distinct fovea. Lateral margin of occiput defined by distinctly angled, raised, sharp carina. Occiput (except extreme lateral margin) smooth. Ocelli small, ratio of maximum diameter of a lateral ocellus to shortest distance between lateral ocelli 0.2–0.4. Anterior ocellus close to posterior ocelli, posterior margin of anterior ocellus behind or subcontiguous with a transverse line running through anterior margins of posterior ocelli. Relative position of toruli close to ocelli, ratio of vertical distance between inner margin of torulus and ventral margin of clypeus to vertical distance between anterior ocellus and torulus < 2.0. Median keel of face present, extending to posterior margin of clypeus. Vertical carina adjacent to ventral margin of torulus present. Facial sculpture almost entirely foveate, slightly horizontally striate along median keel. Facial impression absent, face flat. Antennal scrobe absent. Anterior tentorial pits large. Vertical delineations on lower face absent. Ventral clypeal margin laterally, close to anterior mandibular articulation, straight. Ventral clypeal margin medially emarginate. Clypeus foveate-punctate; horizontally striate. Malar space adjacent to anterior articulation of mandible evenly rounded, foveate. Malar sulcus absent. Compound eye close to posterior ocellus, ratio of distance between compound eye and posterior mandibular articulation to distance between posterior ocellus and compound eye > 1.2. Compound eye, in dorsal view, distinctly protruding from the surface of the head, particularly laterally. Pubescence on compound eye absent. Orbital furrows absent. Lateral frontal carina of face absent. Dorsal aspect of vertex deeply foveate. Posterior aspect of vertex foveate. Hair punctures on lateral aspect of vertex absent. Posterior surface of head almost flat, not deeply impressed.

*Labial-maxillary complex*. Apical segment of maxillary palp with pubescence, consisting only of erect setae. First segment of labial palp shorter than apical segment. Labial palp composed of three segments. Apical seta on apical segment of maxillary palp shorter than twice length of second longest apical seta. Erect setae medially on apical segment of maxillary palp present. Maxillary palp composed of three segments. Last two segments of maxillary palp (in normal repose) curved inwards. Distal margin of subapical segment of maxillary palp slanting inwards, apical segment bending inwards. Apical segment of maxillary palp more than 1.5 times as long as preceding segment.

*Antenna*. Articulation between flagellomeres in antenna connate with articles broadly joined. Male antenna composed of 12 flagellomeres. Placoidal sensilla absent. Second flagellomere of male antenna cylindrical. Length of second flagellomere of male antenna longer than first flagellomere.

*Pronotum*. Macrosculpture on lateral surface of pronotum present, dorsomedially with fine transverse reticulation, dorso-laterally foveate-punctate. Pubescence on lateral surface of pronotum absent. Anterior flange of pronotal plate distinctly protruding anteriorly, transversely striate. Carinae extending posteriorly from lateral margin of pronotal plate absent. Lateral pronotal carina present. Pronotal crest absent. Dorsal margin of pronotal plate (in anterior view) rounded. Submedian pronotal depressions closed laterally, deep. Lateral margin of pronotal plate defined all the way to the dorsal margin of the pronotum. Pronotal plate wide, almost as wide as head.

*Mesoscutum*. Mesoscutal surface convex, evenly curved. Sculpture on mesoscutum present, foveate-punctate, with remnants of transverse costae. Notaulus present, marked by series of deep subcontiguous pits of uniform width. Median mesoscutal carina absent. Anterior admedial lines present, flat, indistinct, with adjacent cuticular surface foveate. Median mesoscutal impression present, long, reaching over 1/2 length of mesoscutum. Parascutal carina distinctly sinuate, posteriorly ending in posteroventrally directed projection.

*Mesopleuron*. Dorsally irregularly horizontally costate with occasional fovea, ventrally smooth. Subpleuron entirely smooth, glabrous. Lower mesopleuron micro-pitted anteriorly, smooth and glabrous posteriorly. Epicnemial carina present, running from mesoscutum to anterior margin of mesopleural carina, spread out ventrally, shagreened. Lateroventral mesopleural carina present, marking abrupt change of slope of mesopectus. Mesopleural triangle absent. Subalar pit large and well defined, lying in posterior end of subalar groove. Speculum present, smooth to micro-pitted. Mesopleural carina present, complete, composed of several long, parallel, straight carinae. Anterior end of mesopleural carina inserting above notch in anterior margin of mesopleuron.

*Scutellum*. Dorsal surface of scutellum foveate-areolate. Circumscutellar carina absent. Posterior margin of axillula marked by distinct ledge, axillula distinctly impressed adjacent to ledge. Lateroventral margin of scutellum posterior to auricula smooth, becoming dorsoventrally striate posteriorly. Dorsoposterior part of scutellum produced posteriorly into sharp spine, greater than 1.0× length of petiole. Dorsal part of scutellum entirely rugose. Scutellar plate absent. Scutellar foveae present, three, with lateral foveal bissected by longitudinal carina, resulting in five longitudinally elongate subfovea. Longitudinal scutellar carinae absent. Single longitudinal carina separating scutellar foveae absent. Posterolateral margin of scutellum drawn out into distinct protuberance. Lateral Lateral bar narrow, with strong strigate, foveate sculpture.

*Metapectal-propodeal complex*. Metapectal cavity anterodorsal to metacoxal base present, ill-defined. Anterior margin of metapectal-propodeal complex separated from mesopleuron by deep, broad, uninterrupted marginal impression. Posteroventral corner of metapleuron (in lateral view) rounded, not drawn out posteriorly. Anterior impression of metepimeron present, narrow, linear impression, not broadened ventrally. Posterior margin of metepimeron distinct, separating metepimeron from propodeum. Subalar area slightly broadened anteriorly, without longitudinal division indicated. Calyptra present, blunt, lobe-like, polished posteriorly with setiferous punctures anteriorly. Dorsellum present with two strong medial fovea, glabrous. Anterior impression of metepisternum, immediately beneath anterior end of metapleural carina, large and wide. Pubescence thin, evenly covering entire metapectal-propodeal complex. Propodeal spurs present, foveate. Lateral propodeal carinae present, not reaching scutellum. Ventral end of lateral propodeal carina terminating before reaching nucha. Inter propodeal carinae space lightly setose, horizontally striate; glabrous with bifurcating central carina, foveate. Petiolar foramen removed from metacoxae, directed posteriorly. Horizontal carina running anteriorly from lateral propodeal carina present. Lateral propodeal carina straight, sub-parallel. Calyptra, in lateral view, elongate. Propodeum relatively short, not drawn out posteriorly. Calyptra, in posterior view, dorsoventrally elongate.

*Legs*. Pubescence posterolaterally on metacoxa sparse to moderately dense, confined dense hair patch absent. Microsculpture on hind coxa absent. Longitudinal carina on the posterior surface of metatibia absent. Metafemoral spine present, elongate, extending distally as low keel along ventral femoral margin. Distal mesotibial spurs shorter than medial spurs. Distal metatibial spurs equal in length to medial spurs. Ratio of first metatibial segment to remaining 4 segments equal to 1.0. Pubescence on outer surface of metatarsal claw sparse, consisting of few setae. Outer surface of metatarsal claw entirely smooth. Apical seta of metatarsal claw positioned on outer surface below dorsal margin. Base of metatarsal claw weakly expanded, apex slightly bent, ratio width of base to length of apex <0.6.

*Forewing*. Pubescence of forewing absent on basal half of wing, sparse distally. Apical margin of female forewing rounded. Rs+M of forewing tubular. Mesal end of Rs+M vein situated closer to posterior margin of Forewing, directed towards posterior end of basalis. Vein R1 tubular along at least basal part of anterior margin of marginal cell. Basal abscissa of R1 (the abscissa between 2r and the Forewing margin) of forewing as broad as adjacent wing veins. Forewing entirely lightly infuscate. Marginal cell of forewing membranous, similar to other wing cells. Areolet present, incomplete, open posteriorly. Hair fringe along apical margin of forewing absent.

*Petiole*. Slightly elongate, 1.5–2× longer than wide. Surface of petiole longitudinally costate, ventral keel absent. Posterior part of male petiole not abruptly widened. Ventral flange of annulus of male petiole absent.

*Metasoma*. Setal band (hairy ring) at base of tergum 3 absent, base of metasoma glabrous. Tergum 3 distinctly smaller than tergum 4. Posterior margin of tergum 3 smoothly rounded. Posterior margin of tergum 4 straight. In lateral view, sternum 3 exposed, ventral border of T2–T7 visible. Sculpture on metasomal terga present, finely punctate laterally, dorsally; posteriorly with large setal pits. Syntergum absent, all postpetiolar terga free. Annulus absent. Peg-like setae on T6–T7 absent.

**Figure 26.**
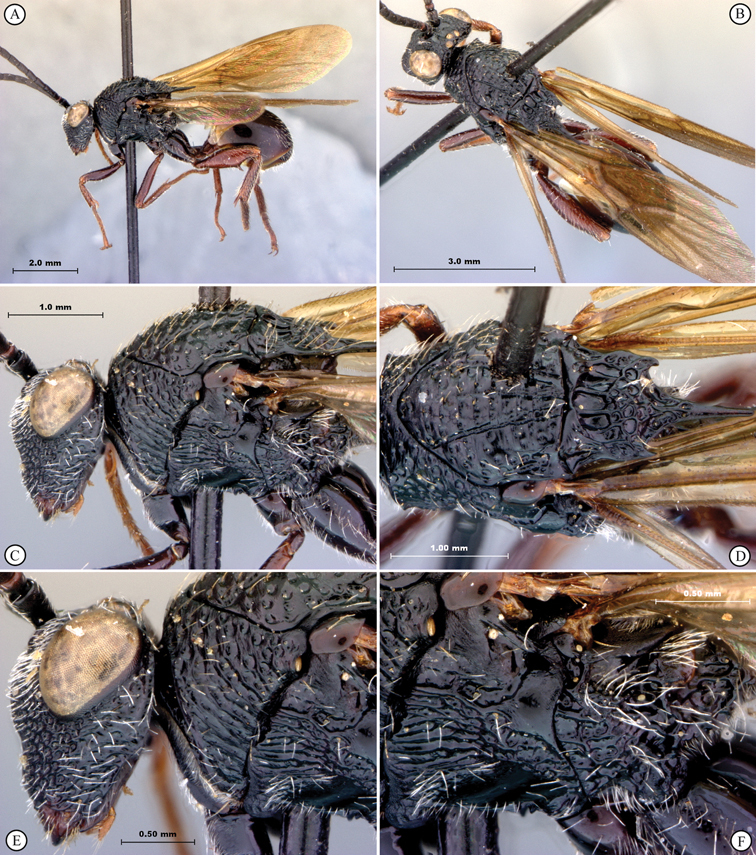
*Oberthuerella pardolatus* Buffington & van Noort, sp. n., holotype **A** lateral habitus **B** dorsal habitus **C** head and mesosoma, lateral view **D** mesosoma, dorsal view **E** head and pronotum, lateral view **F** meso and metapleurae, lateral view.

**Figure 27.**
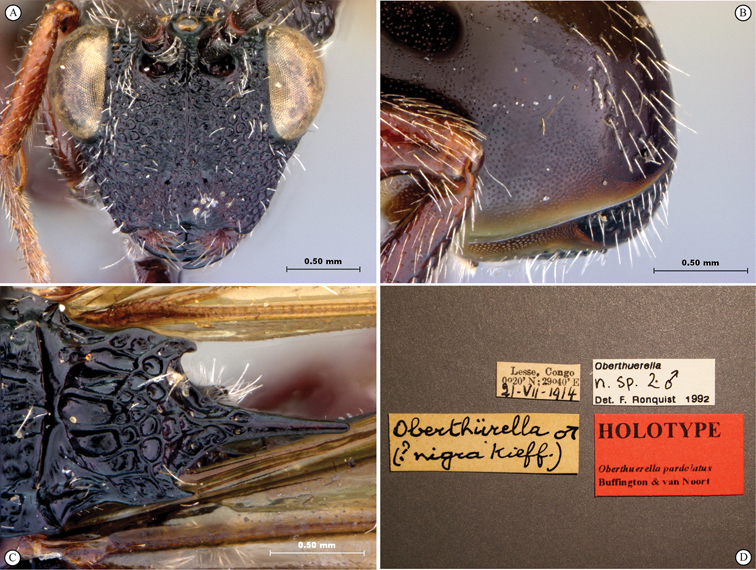
*Oberthuerella pardolatus* Buffington & van Noort, sp. n., holotype **A** head, anterior view **B** hind margin of metasoma, lateral view **C** scutellum, dorsal view **D** labels.

##### Diagnosis.

This species has a somewhat unique foveal pattern on the pronotum and mesoscutum, where the inter-foveal space is wide and shagreened; another species with this wide inter-foveal space is *Oberthuerella kibalensis*, but in that species, there are 5 subfovea at the anterior base of scutellum (three subfovea in *Oberthuerella pardolatus*).

##### Etymology.

Latin for *leopard*, in reference to the fovea that resemble leapard spots on the pronotum and mesoscutum.

##### Distribution.

Democratic Republic of Congo. **Link to Distribution Map.** [http://hol.osu.edu/map-full.html?id=300218]

##### Material examined.

Holotype, male: **DEMOCRATIC REPUBLIC OF THE CONGO:** Lesse, 00°20'N, 29°40'E, 21.VII.1914, USNM ENT 00764780 (deposited in MCZC).

#### Oberthuerella sharkeyi

Buffington & van Noort sp. n.

urn:lsid:zoobank.org:act:8E12458A-F38E-4383-9F13-7A3827AD1C27

urn:lsid:biosci.ohio-state.edu:osuc_concepts:300219

Morphbank accession: 704656–704668

http://www.waspweb.org/Cynipoidea/Liopteridae/Oberthuerellinae/Oberthuerella/Oberthuerella_sharkeyi.htm

http://species-id.net/wiki/Oberthuerella_sharkeyi

Figures 28 –29

##### Description.

Coloration of head and mesosoma, black to dark brown; metasoma and legs yellow-orange. Sculpture on vertex, lateral surface of pronotum and mesoscutum present, deeply foveate laterally on head, pronotum; deeply horizontally striate on mesoscutum.

*Head*. Broadly triangular, in anterior view. Pubescence on head present, sparse setae scattered over head. Sculpture along lateral margin of occiput absent. Gena (measured from compound eye to posterolateral margin of head) short, ratio of length of gena to length of compound eye in dorsal view < 0.3, in dorsal view. Sculpture of gena deeply striate with remnants of fovea. Lateral margin of occiput defined by distinctly angled, raised, sharp carina. Occiput (except extreme lateral margin) smooth. Ocelli small, ratio of maximum diameter of a lateral ocellus to shortest distance between lateral ocelli 0.2–0.4. Anterior ocellus close to posterior ocelli, posterior margin of anterior ocellus behind or subcontiguous with a transverse line running through anterior margins of posterior ocelli. Relative position of toruli close to ocelli, ratio of vertical distance between inner margin of torulus and ventral margin of clypeus to vertical distance between anterior ocellus and torulus < 2.0. Median keel of face present, short, not extending beyond toruli. Vertical carina adjacent to ventral margin of torulus absent. Facial sculpture present, punctate-rugose, transversely striate; striations meeting at midline of face. Facial impression absent, face flat. Antennal scrobe absent. Anterior tentorial pits large. Vertical delineations on lower face absent. Ventral clypeal margin laterally, close to anterior mandibular articulation, straight. Ventral clypeal margin medially straight, not projecting. Clypeus foveate-punctate. Malar space adjacent to anterior articulation of mandible evenly rounded, striate. Malar sulcus absent. Compound eye close to posterior ocellus, ratio of distance between compound eye and posterior mandibular articulation to distance between posterior ocellus and compound eye > 1.2. Compound eye, in dorsal view, distinctly protruding from the surface of the head, particularly laterally. Pubescence on compound eye absent. Orbital furrows absent. Lateral frontal carina of face absent. Dorsal aspect of vertex deeply foveate. Posterior aspect of vertex punctate. Hair punctures on lateral aspect of vertex absent. Posterior surface of head almost flat, not deeply impressed.

*Labial-maxillary complex*. Apical segment of maxillary palp with pubescence, consisting only of erect setae. Apical seta on apical segment of maxillary palp shorter than twice length of second longest apical seta. Erect setae medially on apical segment of maxillary palp present. Last two segments of maxillary palp (in normal repose) curved inwards. Apical segment of maxillary palp more than 1.5 times as long as preceding segment.

*Antenna*. Articulation between flagellomeres in antenna connate with articles broadly joined. Female antenna composed of 11 flagellomeres. Female F1 as long as F2. Flagellomeres of female antenna cylindrical, not widened towards apex, non-clavate. Placoidal sensilla absent. Distal flagellomeres of female antenna not conspicuously enlarged compared to proximal flagellomeres.

*Pronotum*. Macrosculpture on lateral surface of pronotum present, foveate. Pubescence on lateral surface of pronotum absent. Anterior flange of pronotal plate distinctly protruding anteriorly, transversely striate. Carinae extending posteriorly from lateral margin of pronotal plate absent. Lateral pronotal carina present. Pronotal crest absent. Dorsal margin of pronotal plate (in anterior view) straight; rounded. Submedian pronotal depressions closed laterally, deep. Lateral margin of pronotal plate defined all the way to the dorsal margin of the pronotum. Pronotal plate wide, almost as wide as head.

*Mesoscutum*. Mesoscutal surface convex, evenly curved. Sculpture on mesoscutum present, foveate-punctate, with remnants of transverse costae. Notaulus present, marked by series of deep subcontiguous pits of uniform width. Median mesoscutal carina absent. Anterior admedial lines present, flat, indistinct, with adjacent cuticular surface foveate. Median mesoscutal impression present, long, reaching over 1/2 length of mesoscutum. Parascutal carina distinctly sinuate, posteriorly ending in posteroventrally directed projection.

*Mesopleuron*. Dorsally irregularly horizontally costate with distinct midsclerite longitudinal impression, ventrally smooth. Subpleuron entirely smooth with long, white setae over entire surface. Lower mesopleuron micro-pitted anteriorly, smooth and glabrous posteriorly. Epicnemial carina present, running from mesoscutum to anterior margin of mesopleural carina, spread out ventrally, shagreened. Lateroventral mesopleural carina present, marking abrupt change of slope of mesopectus. Mesopleural triangle absent. Subalar pit large and well defined, lying in posterior end of subalar groove. Speculum present, distinctly foveate anteriorly; shagreened, trough-like posteriorly. Mesopleural carina present, complete, composed of one complete, straight main carina, with short subordinate carinae. Anterior end of mesopleural carina inserting above notch in anterior margin of mesopleuron.

*Scutellum*. Dorsal surface of scutellum foveate-areolate. Circumscutellar carina absent. Posterior margin of axillula marked by distinct ledge, axillula distinctly impressed adjacent to ledge. Lateroventral margin of scutellum posterior to auricula smooth, becoming dorsoventrally striate posteriorly. Dorsoposterior part of scutellum produced posteriorly into sharp spine, less than 1.0× length of petiole. Dorsal part of scutellum entirely rugose. Scutellar plate absent. Scutellar foveae present, two, each with one longitudinal division resulting in transverse row of 4 longitudinally elongate subfovea. Longitudinal scutellar carinae absent. Single longitudinal carina separating scutellar foveae present, short, ending at posterior margin of foveae. Posterolateral margin of scutellum drawn out into distinct protuberance. Lateral bar with strong strigate sculpture, narrow.

*Metapectal-propodeal complex*. Metapectal cavity anterodorsal to metacoxal base present, ill-defined. Anterior margin of metapectal-propodeal complex separated from mesopleuron by deep, broad, uninterrupted marginal impression. Posteroventral corner of metapleuron (in lateral view) rounded, not drawn out posteriorly. Anterior impression of metepimeron present, narrow, linear impression, not broadened ventrally. Posterior margin of metepimeron distinct, separating metepimeron from propodeum. Subalar area abruptly broadened anteriorly, with an indicated longitudinal division. Calyptra present, blunt, lobe-like, polished posteriorly with setiferous punctures anteriorly. Dorsellum present, two strong medial fovea, laterally strongly excavated with fine pubescence in lateral depressions. Anterior impression of metepisternum, immediately beneath anterior end of metapleural carina, large and wide. Pubescence thin, evenly covering entire metapectal-propodeal complex. Propodeal spurs present, foveate. Lateral propodeal carinae present, not reaching scutellum. Ventral end of lateral propodeal carina terminating before reaching nucha. Inter propodeal carinae space lightly setose, foveate. Petiolar foramen removed from metacoxae, directed posteriorly. Horizontal carina running anteriorly from lateral propodeal carina present. Lateral propodeal carina straight, sub-parallel. Calyptra, in lateral view, elongate. Propodeum relatively short, not drawn out posteriorly. Calyptra, in posterior view, dorsoventrally elongate.

*Legs*. Pubescence posterolaterally on metacoxa sparse to moderately dense, confined dense hair patch absent. Microsculpture on hind coxa absent. Longitudinal carina on the posterior surface of metatibia absent. Metafemoral spine present, elongate, extending distally as low keel along ventral femoral margin. Distal mesotibial spurs shorter than medial spurs. Distal metatibial spurs equal in length to medial spurs. Ratio of first metatibial segment to remaining 4 segments greater than 1.0. Pubescence on outer surface of metatarsal claw sparse, consisting of few setae. Outer surface of metatarsal claw microcarinate. Apical seta of metatarsal claw positioned on outer surface below dorsal margin. Base of metatarsal claw weakly expanded, apex slightly bent, ratio width of base to length of apex <0.6.

*Forewing*. Pubescence of forewing absent on basal half of wing, sparse distally. Apical margin of female forewing rounded. Rs+M of forewing tubular. Mesal end of Rs+M vein situated closer to posterior margin of forewing, directed towards posterior end of basalis. Vein R1 tubular along at least basal part of anterior margin of marginal cell. Basal abscissa of R1 (the abscissa between 2r and the forewing margin) of forewing as broad as adjacent wing veins. Forewing entirely lightly infuscate. Marginal cell of forewing membranous, similar to other wing cells. Areolet absent. Hair fringe along apical margin of forewing absent.

*Petiole*. About as long as wide. Surface of petiole longitudinally costate, ventral keel absent. Posterior part of female petiole not abruptly widened. Ventral flange of annulus of female petiole absent.

*Metasoma*. Setal band (hairy ring) at base of tergum 3 absent, base of metasoma glabrous. Tergum 3 distinctly smaller than tergum 4. Posterior margin of tergum 3 smoothly rounded. Posterior margin of tergum 4 arcuate. In lateral view, sternum 3 exposed, ventral border of T2–T7 visible. Sculpture on metasomal terga present, dorsally finely punctate, posteriorly with distinct bands of setiferous pits. Syntergum absent, all postpetiolar terga free. Annulus absent. Peg-like setae on T6–T7 present. Posteroventral cavities of female metasoma T7 present, glabrous save for few, long setae. Female posteroventral margin of T6–T7 distinctly sinuate. Terebrum and hypopygium (in lateral view) curved, pointing upwards.

*Ovipositor*. First valvula of ovipositor narrowing gradually, not broadened apically, serrate at tip. Ovipositor clip absent.

**Figure 28.**
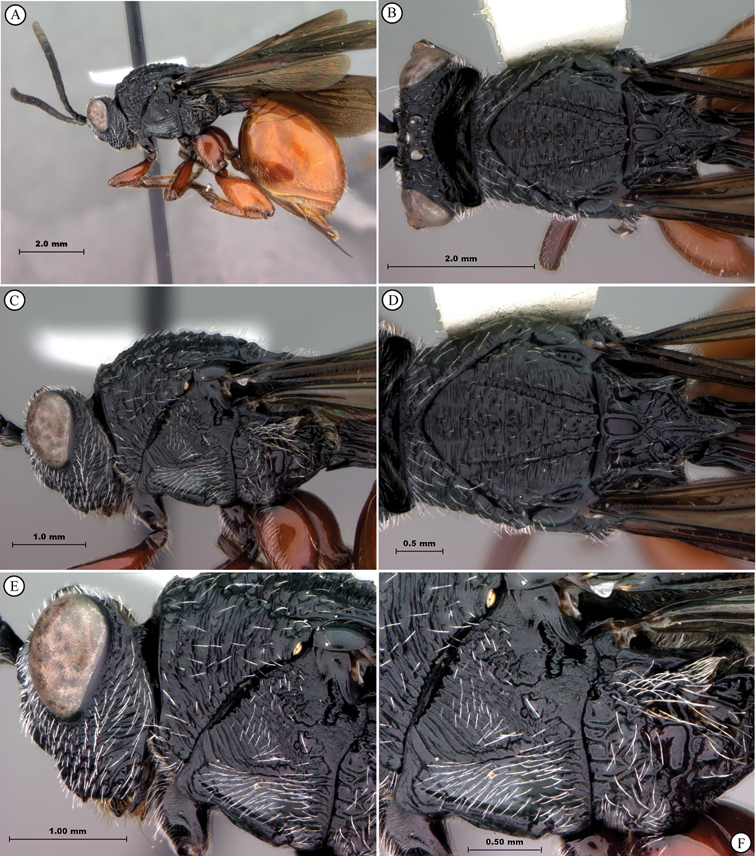
*Oberthuerella sharkeyi* Buffington & van Noort, sp. n., holotype **A** lateral habitus **B** head and mesosoma, dorsal view **C** head and mesosoma, lateral view **D** mesosoma, dorsal view **E** head and pronotum, lateral view **F** meso- and metapleurae.

**Figure 29.**
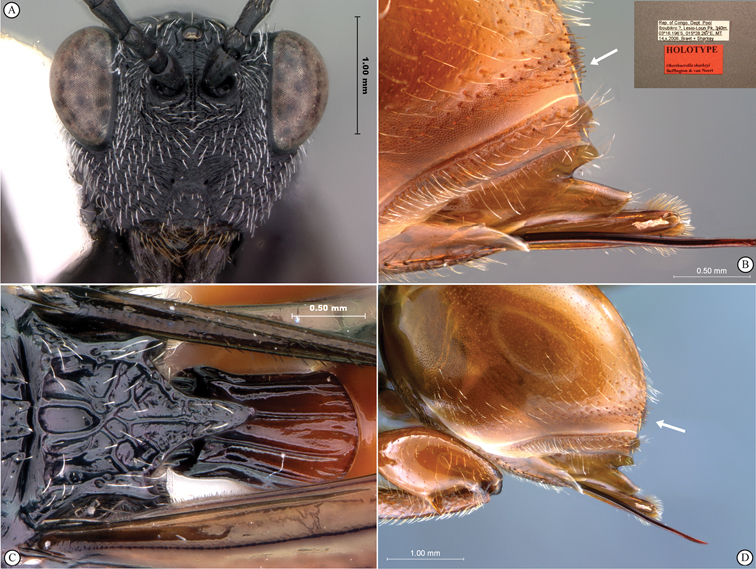
*Oberthuerella sharkeyi* Buffington & van Noort, sp. n., holotype **A** head, anterior view **B** hind margin of metasoma, lateral view; inset, labels **C** scutellum, dorsal view **D** metasoma, lateral view. Arrows indicate location of peglike setae.

##### Diagnosis.

This species has a distinctly striate lateral aspect of the pronotum, as well as a horizontally striate mesopleuron; these features are shared with *Oberthuerella kibalensis* and *Oberthuerella breviscutellaris*, but differs from the former by having an entirely orange metasoma (dark brown/black in *Oberthuerella kibalensis*), and differs from the latter having 4 subfovea present at the anterior base of the scutellum (10 subfovea in *Oberthuerella breviscutellaris*).

##### Etymology.

Named in honor of our friend and acclaimed hymenopterist Mike Sharkey (University of Kentucky, Lexington), collector of the type series for this species.

##### Distribution.

Congo. **Link to Distribution Map.** [http://hol.osu.edu/map-full.html?id=300219]

##### Material examined.

Holotype, female: **CONGO:** Pool Dépt., Iboubikro, Lesio Louna Reserve, 03°16.196'S, 15°28.267'E, 340m, 14.X.2008, malaise trap, Braet and Sharkey, USNM ENT 00764770 (deposited in USNM). *Paratypes*: (6 females) **CONGO:** Pool Dépt., Iboubikro, Lesio Louna Reserve, 03°16.196'S, 15°28.267'E, 340m, 27.X.2008, malaise trap, Braet and Sharkey (1 female, USNM ENT 00764767 (SAMC)). Pool Dépt., Iboubikro, MT 4, Lesio Louna Reserve, 03°16.196'S, 15°28.267'E, 340m, 13.X.2008, malaise trap, Braet and Sharkey (1 female, USNM ENT 00764771 (USNM)). Pool Dépt., Iboubikro, MT 4, Lesio Louna Reserve, 03°16.196'S, 15°28.267'E, 340m, 13.X.2008, malaise trap, Braet and Sharkey (1 female, USNM ENT 00764773 (USNM)). Pool Dépt., Iboubikro, MT 4, Lesio Louna Reserve, 03°16.196'S, 15°28.267'E, 340m, 20.X.2008, malaise trap, Braet and Sharkey (1 female, USNM ENT 00764772 (USNM)). Pool Dépt., Iboubikro, MT 5, Lesio Louna Reserve, 03°16.196'S, 15°28.267'E, 340m, 20.X.2008, malaise trap, Braet and Sharkey (1 female, USNM ENT 00764766 (MRAC)). Pool Dépt., Iboubikro, MT 5, Lesio Louna Reserve, 03°16.196'S, 15°28.267'E, 340m, 20.X.2008, malaise trap, Braet and Sharkey (1 female, USNM ENT 00764765 (USNM)).

#### Oberthuerella simba

Buffington & van Noort sp. n.

urn:lsid:zoobank.org:act:53FF7B99-D3E9-4CC6-9545-10D58E1C1220

urn:lsid:biosci.ohio-state.edu:osuc_concepts:300220

Morphbank accession: 704669–704682

http://www.waspweb.org/Cynipoidea/Liopteridae/Oberthuerellinae/Oberthuerella/Oberthuerella_simba.htm

http://species-id.net/wiki/Oberthuerella_simba

**Figure 30.**
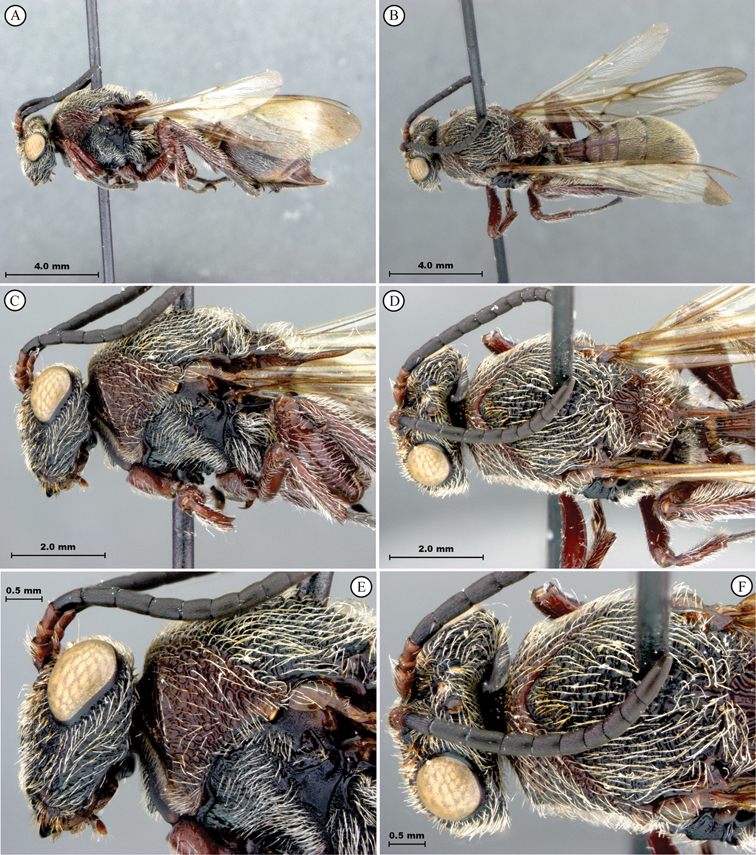
*Oberthuerella simba* Buffington & van Noort, sp. n., holotype **A** lateral habitus **B** dorsal habitus **C** head and mesosoma, lateral view **D** head and mesosoma, dorsal view **E** head and pronotum, lateral view **F** head and mesosoma, dorsal view.

Figures 30 [Fig F31] –32

##### Description.

Coloration of head, mesosoma, and metasoma, dark reddish brown; legs reddish brown. Sculpture on vertex, lateral surface of pronotum and mesoscutum present, deeply foveate laterally on head, pronotum; deeply horizontally striate on mesoscutum.

*Head*. Broadly triangular, in anterior view. Pubescence on head present, dense setae covering head. Sculpture along lateral margin of occiput many costulae. Gena (measured from compound eye to posterolateral margin of head) short, ratio of length of gena to length of compound eye in dorsal view < 0.3, in dorsal view. Sculpture of gena deeply striate with remnants of fovea. Lateral margin of occiput defined by distinctly angled, raised, sharp carina. Occiput (except extreme lateral margin) with distinct subvertical, slightly and evenly curved costulae. Ocelli small, ratio of maximum diameter of a lateral ocellus to shortest distance between lateral ocelli 0.2–0.4. Anterior ocellus close to posterior ocelli, posterior margin of anterior ocellus behind or subcontiguous with a transverse line running through anterior margins of posterior ocelli. Relative position of toruli close to ocelli, ratio of vertical distance between inner margin of torulus and ventral margin of clypeus to vertical distance between anterior ocellus and torulus < 2.0. Median keel of face present, extending to posterior margin of clypeus. Vertical carina adjacent to ventral margin of torulus absent. Facial sculpture present, punctate-rugose, transversely striate; striations meeting at medial keel. Facial impression absent, face flat. Antennal scrobe absent. Anterior tentorial pits large. Vertical delineations on lower face absent. Ventral clypeal margin laterally, close to anterior mandibular articulation, straight. Ventral clypeal margin medially emarginate. Clypeus foveate-punctate. Malar space adjacent to anterior articulation of mandible evenly rounded, striate-foveate. Malar sulcus absent. Compound eye close to posterior ocellus, ratio of distance between compound eye and posterior mandibular articulation to distance between posterior ocellus and compound eye > 1.2. Compound eye, in dorsal view, distinctly protruding from the surface of the head, particularly laterally. Pubescence on compound eye absent. Orbital furrows absent. Lateral frontal carina of face absent. Dorsal aspect of vertex deeply foveate. Posterior aspect of vertex foveate. Hair punctures on lateral aspect of vertex present, distinctly enlarged. Posterior surface of head deeply impressed around postocciput.

*Labial-maxillary complex*. Apical segment of maxillary palp with pubescence, consisting only of erect setae. First segment of labial palp shorter than apical segment. Apical seta on apical segment of maxillary palp shorter than twice length of second longest apical seta. Erect setae medially on apical segment of maxillary palp present. Maxillary palp composed of four segments. Last two segments of maxillary palp (in normal repose) straight. Distal margin of subapical segment of maxillary palp distinctly slanting outwards, apical segment bending outwards. Apical segment of maxillary palp more than 1.5 times as long as preceding segment.

*Antenna*. Articulation between flagellomeres in antenna connate with articles broadly joined. Female antenna composed of 11 flagellomeres. Female F1 shorter than F2; gold in color. Flagellomeres of female antenna cylindrical, not widened towards apex, non-clavate. Placoidal sensilla absent. Distal flagellomeres of female antenna not conspicuously enlarged compared to proximal flagellomeres.

*Pronotum*. Macrosculpture on lateral surface of pronotum present, foveate. Pubescence on lateral surface of pronotum present, long, dense. Anterior flange of pronotal plate distinctly protruding anteriorly, smooth. Carinae extending posteriorly from lateral margin of pronotal plate absent. Lateral pronotal carina present. Pronotal crest absent. Dorsal margin of pronotal plate (in anterior view) rounded. Submedian pronotal depressions closed laterally, deep. Lateral margin of pronotal plate defined all the way to the dorsal margin of the pronotum. Pronotal plate wide, almost as wide as head.

*Mesoscutum*. Mesoscutal surface convex, evenly curved. Sculpture on mesoscutum present, deeply transversely costate; densely setose. Notaulus present, marked by series of deep subcontiguous pits of uniform width. Median mesoscutal carina absent. Anterior admedial lines present, with adjacent cuticular surface foveate. Median mesoscutal impression present, long, reaching over 1/2 length of mesoscutum. Parascutal carina distinctly sinuate, posteriorly ending in posteroventrally directed projection.

*Mesopleuron*. Dorsally with strigae running dorsoventrally; ventrally smooth, medially densely setose. Subpleuron entirely smooth with long, white setae over entire surface. Lower mesopleuron medially smooth, setose; costate laterally, ventrally. Epicnemial carina present on ventral half of mesopleuron; shagreened, ventrally bulbous near mesosternum. Lateroventral mesopleural carina absent. Mesopleural triangle absent. Subalar pit large and well defined, lying in posterior end of subalar groove. Speculum present, shagreened. Mesopleural carina absent.

*Scutellum*. Dorsal surface of scutellum foveate-areolate. Circumscutellar carina present, incomplete, laterally delimiting dorsal and ventral halves of scutellum, not present posteriorly. Posterior margin of axillula marked by distinct ledge, axillula distinctly impressed adjacent to ledge. Lateroventral margin of scutellum posterior to auricula entirely smooth. Dorsoposterior part of scutellum produced posteriorly into sharp spine, greater than 1.0× length of petiole. Dorsal part of scutellum entirely rugose. Scutellar plate absent. Scutellar foveae present, two, each with four longitudinal divisions resulting in transverse row of 10 longitudinally elongate subfovea. Longitudinal scutellar carinae absent. Single longitudinal carina separating scutellar foveae absent. Posterolateral margin of scutellum drawn out into distinct protuberance. Lateral Lateral bar narrow, with strong strigate, foveate sculpture.

*Metapectal-propodeal complex*. Metapectal cavity anterodorsal to metacoxal base absent. Anterior margin of metapectal-propodeal complex separated from mesopleuron by deep, broad, uninterrupted marginal impression. Posteroventral corner of metapleuron (in lateral view) rounded, not drawn out posteriorly. Anterior impression of metepimeron present, triangular, with broadest part ventrally. Posterior margin of metepimeron distinct, separating metepimeron from propodeum. Subalar area abruptly broadened anteriorly, with an indicated longitudinal division. Calyptra present, blunt, lobe-like, polished posteriorly with setiferous punctures anteriorly. Dorsellum present with two strong medial fovea, glabrous. Anterior impression of metepisternum, immediately beneath anterior end of metapleural carina, large and wide. Pubescence long, dense, silvery on metapleuron; long, thin on propodeum. Propodeal spurs present, crenulate. Lateral propodeal carinae present, not reaching scutellum. Ventral end of lateral propodeal carina terminating before reaching nucha. Inter propodeal carinae space lightly setose with two distinct fovea at dorsal end. Petiolar foramen removed from metacoxae, directed posteriorly. Horizontal carina running anteriorly from lateral propodeal carina present. Lateral propodeal carina straight, sub-parallel. Calyptra, in lateral view, elongate. Propodeum relatively short, not drawn out posteriorly. Calyptra, in posterior view, dorsoventrally elongate.

*Legs*. Pubescence posterolaterally on metacoxa moderately dense, confined dense hair patch absent. Microsculpture on hind coxa absent. Longitudinal carina on the posterior surface of metatibia absent. Metafemoral spine present, elongate, extending distally as low keel along ventral femoral margin. Distal mesotibial spurs shorter than medial spurs. Distal metatibial spurs shorter than medial spurs. Ratio of first metatibial segment to remaining 4 segments greater than 1.0. Pubescence on outer surface of metatarsal claw sparse, consisting of few setae. Outer surface of metatarsal claw entirely smooth. Apical seta of metatarsal claw positioned on outer surface below dorsal margin. Base of metatarsal claw lammelate, with translucent cuticular flange.

*Forewing*. Pubescence of forewing absent on basal half of wing, sparse distally. Apical margin of female forewing rounded. Rs+M of forewing tubular. Mesal end of Rs+M vein situated closer to anterior margin of forewing, directed towards middle of basalis. Vein R1 tubular along at least basal part of anterior margin of marginal cell. Basal abscissa of R1 (the abscissa between 2r and the forewing margin) of forewing as broad as adjacent wing veins. Coloration of forewing absent, entire wing hyaline. Marginal cell of forewing membranous, similar to other wing cells. Areolet present, incomplete, open posteriorly. Hair fringe along apical margin of forewing absent.

*Petiole*. Slightly elongate, 1.5–2× longer than wide. Surface of petiole longitudinally costate, ventral keel absent, lateral patches of long white setae present. Posterior part of female petiole not abruptly widened. Ventral flange of annulus of female petiole absent.

*Metasoma*. Setal band (hairy ring) at base of tergum 3 present, interrupted dorsally, extending laterally to middle of sclerite. Tergum 3 distinctly smaller than tergum 4. Posterior margin of tergum 3 smoothly rounded. Posterior margin of tergum 4 straight. Sternum 3 exposed, ventral border of T2–T7 visible. Sculpture on metasomal terga present, composed to dense seta bearing punctures, interrupted dorsally on T3–T5; dense setae present across entire metasomal surface. Syntergum absent, all postpetiolar terga free. Annulus absent. Peg-like setae on T6–T7 absent. Posteroventral cavities of female metasoma T7 present, setose. Female posteroventral margin of T6–T7 straight, parallel. Terebrum and hypopygium (in lateral view) straight, pointing posteriorly.

**Figure 31. F31:**
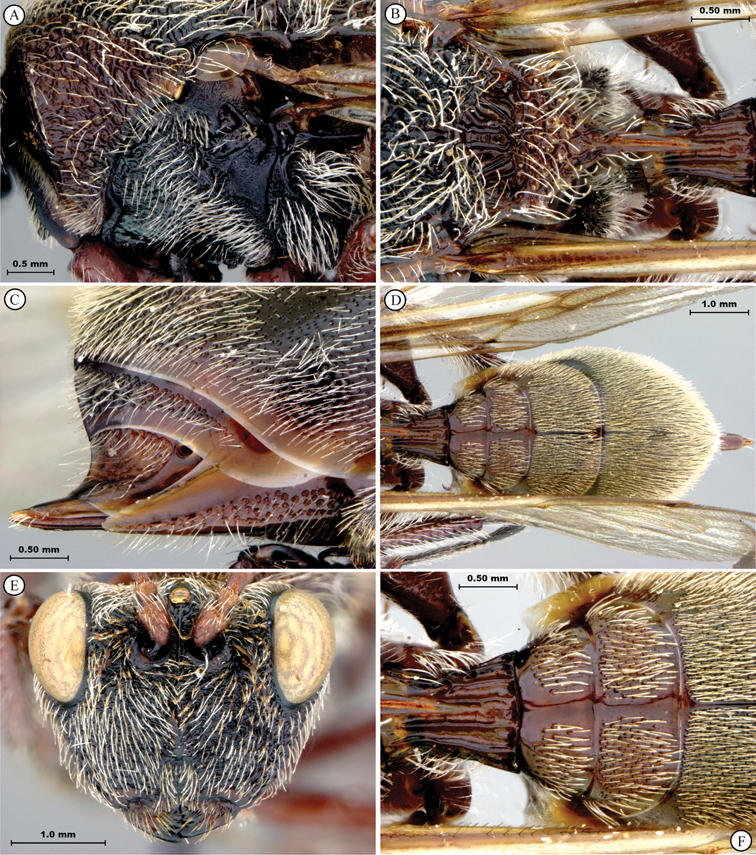
*Oberthuerella simba* Buffington & van Noort, sp. n., holotype **A** meso- and metapleurae **B** scutellum and petiole, dorsal view **C** posterior margin of metasoma, lateral view **D** metasoma, dorsal view **E** head, anterior view **F** petiole and base of metasoma, dorsal view.

**Figure 32.**
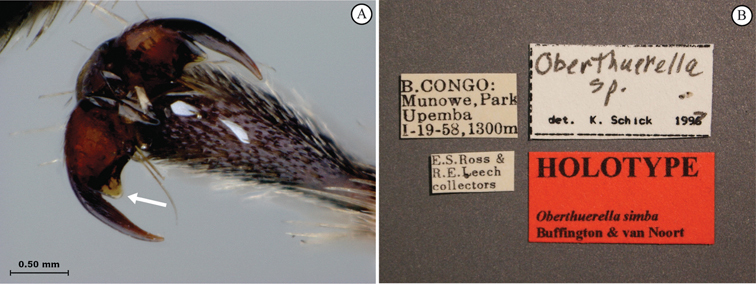
*Oberthuerella simba* Buffington & van Noort, sp. n., holotype **A** metatarsal claw, arrow indicates lobe **B** labels.

##### Diagnosis.

Easily distinguished from all other *Oberthuerella* by the predominance of golden setae on head, mesosoma and metasoma; this feature is only shared with *Oberthuerella aureopilosa*, but this latter species has the speculum smooth (shagreened in *Oberthuerella simba*).

##### Etymology.

Latin for *lion*, in reference to the large size and general setation patterns of this species.

##### Distribution.

Democratic Republic of Congo. **Link to Distribution Map.** [http://hol.osu.edu/map-full.html?id=300220]

##### Material examined.

Holotype, female: **DEMOCRATIC REPUBLIC OF THE CONGO:** Munowe River, Upemba National Park, 19.I.1958, E. S. Ross and R. E. Leech, USNM ENT 00764782 (deposited in CASC).

#### Oberthuerella tibialis

Kieffer

urn:lsid:biosci.ohio-state.edu:osuc_concepts:181561

Morphbank accession: 704683–704703

http://www.waspweb.org/Cynipoidea/Liopteridae/Oberthuerellinae/Oberthuerella/Oberthuerella_tibialis.htm

http://species-id.net/wiki/Oberthuerella_tibialis

Figures 33 –34

Oberthuerella tibialis Kieffer, 1904: 107

##### Description.

Coloration of head and mesosoma, black to dark brown; metasoma and legs yellow-orange. Sculpture on vertex, lateral surface of pronotum and mesoscutum present, deeply foveate laterally on head; pronotum, mesoscutum striate-foveate.

*Head*. Broadly triangular, in anterior view. Pubescence on head present, sparse setae scattered over head. Sculpture along lateral margin of occiput absent. Gena (measured from compound eye to posterolateral margin of head) short, ratio of length of gena to length of compound eye in dorsal view < 0.3, in dorsal view. Sculpture of gena present, with distinct fovea. Lateral margin of occiput defined by distinctly angled, raised, sharp carina. Occiput (except extreme lateral margin) smooth. Ocelli small, ratio of maximum diameter of a lateral ocellus to shortest distance between lateral ocelli 0.2–0.4. Anterior ocellus close to posterior ocelli, posterior margin of anterior ocellus behind or subcontiguous with a transverse line running through anterior margins of posterior ocelli. Relative position of toruli close to ocelli, ratio of vertical distance between inner margin of torulus and ventral margin of clypeus to vertical distance between anterior ocellus and torulus < 2.0. Median keel of face present, extending to middle of face, not reaching clypeus. Vertical carina adjacent to ventral margin of torulus absent. Facial sculpture present, punctate-rugose, transversely striate; striations meeting at medial keel. Facial impression absent, face flat. Antennal scrobe absent. Anterior tentorial pits large. Vertical delineations on lower face absent. Ventral clypeal margin laterally, close to anterior mandibular articulation, straight. Ventral clypeal margin medially emarginate. Clypeus horizontally striate. Malar space adjacent to anterior articulation of mandible evenly rounded, foveate. Malar sulcus absent. Compound eye close to posterior ocellus, ratio of distance between compound eye and posterior mandibular articulation to distance between posterior ocellus and compound eye > 1.2. Compound eye, in dorsal view, distinctly protruding from the surface of the head, particularly laterally. Pubescence on compound eye absent. Orbital furrows absent. Lateral frontal carina of face absent. Dorsal aspect of vertex deeply foveate. Posterior aspect of vertex foveate. Hair punctures on lateral aspect of vertex absent. Posterior surface of head almost flat, not deeply impressed.

*Labial-maxillary complex*. Apical segment of maxillary palp with pubescence, consisting only of erect setae. Apical seta on apical segment of maxillary palp shorter than twice length of second longest apical seta. Erect setae medially on apical segment of maxillary palp present. Maxillary palp composed of three segments. Last two segments of maxillary palp (in normal repose) straight. Apical segment of maxillary palp more than 1.5 times as long as preceding segment.

*Antenna*. Articulation between flagellomeres in antenna connate with articles broadly joined. Female antenna composed of 11 flagellomeres. Female F1 shorter than F2; black. Flagellomeres of female antenna cylindrical, not widened towards apex, non-clavate. Placoidal sensilla absent. Second flagellomere of male antenna cylindrical. Length of second flagellomere of male antenna longer than first flagellomere. Distal flagellomeres of female antenna not conspicuously enlarged compared to proximal flagellomeres.

*Pronotum*. Macrosculpture on lateral surface of pronotum present, dorsomedially with fine transverse reticulation, dorso-laterally foveate-punctate. Pubescence on lateral surface of pronotum absent. Anterior flange of pronotal plate distinctly protruding anteriorly, transversely striate. Carinae extending posteriorly from lateral margin of pronotal plate absent. Lateral pronotal carina present. Pronotal crest absent. Dorsal margin of pronotal plate (in anterior view) straight. Submedian pronotal depressions closed laterally, deep. Lateral margin of pronotal plate defined all the way to the dorsal margin of the pronotum. Pronotal plate wide, almost as wide as head.

*Mesoscutum*. Mesoscutal surface convex, evenly curved. Sculpture on mesoscutum present, foveate-punctate, with remnants of transverse costae. Notaulus present, marked by series of deep subcontiguous pits of uniform width. Median mesoscutal carina absent. Anterior admedial lines present, flat, indistinct, with adjacent cuticular surface foveate. Median mesoscutal impression present, long, reaching over 1/2 length of mesoscutum. Parascutal carina distinctly sinuate, posteriorly ending in posteroventrally directed projection.

*Mesopleuron*. Dorsally irregularly horizontally costate with occasional fovea, ventrally smooth. Subpleuron entirely smooth, glabrous. Lower mesopleuron micro-pitted anteriorly, smooth and glabrous posteriorly. Epicnemial carina present, running from mesoscutum to anterior margin of mesopleural carina, spread out ventrally, shagreened. Lateroventral mesopleural carina present, marking abrupt change of slope of mesopectus. Mesopleural triangle absent. Subalar pit large and well defined, lying in posterior end of subalar groove. Speculum present, smooth to micro-pitted. Mesopleural carina present, complete, composed of several long, irregular, curved carinae. Anterior end of mesopleural carina inserting above notch in anterior margin of mesopleuron.

*Scutellum*. Dorsal surface of scutellum foveate-areolate. Circumscutellar carina absent. Posterior margin of axillula marked by distinct ledge, axillula distinctly impressed adjacent to ledge. Lateroventral margin of scutellum posterior to auricula smooth, becoming dorsoventrally striate posteriorly. Dorsoposterior part of scutellum produced posteriorly into sharp spine, less than 1.0× length of petiole. Dorsal part of scutellum entirely rugose. Scutellar plate absent. Scutellar foveae present, two, each with two longitudinal divisions resulting in transverse row of 6 longitudinally elongate subfovea. Longitudinal scutellar carinae absent. Single longitudinal carina separating scutellar foveae present, short, ending at posterior margin of foveae. Posterolateral margin of scutellum drawn out into distinct protuberance. Lateral bar narrow, with strong strigate, foveate sculpture.

*Metapectal-propodeal complex*. Metapectal cavity anterodorsal to metacoxal base present, ill-defined. Anterior margin of metapectal-propodeal complex separated from mesopleuron by deep, broad, uninterrupted marginal impression. Posteroventral corner of metapleuron (in lateral view) rounded, not drawn out posteriorly. Anterior impression of metepimeron present, narrow, linear impression, not broadened ventrally. Posterior margin of metepimeron distinct, separating metepimeron from propodeum. Subalar area slightly broadened anteriorly, with distinct laterally protruding lobe ventrally. Calyptra present, blunt, lobe-like, polished posteriorly with setiferous punctures anteriorly. Dorsellum present with two strong medial fovea, glabrous. Anterior impression of metepisternum, immediately beneath anterior end of metapleural carina, present, small and narrow. Pubescence thin, evenly covering entire metapectal-propodeal complex. Propodeal spurs present, foveate. Lateral propodeal carinae present, not reaching scutellum. Ventral end of lateral propodeal carina terminating before reaching nucha. Inter propodeal carinae space lightly setose, horizontally striate. Petiolar foramen removed from metacoxae, directed posteriorly. Horizontal carina running anteriorly from lateral propodeal carina present. Lateral propodeal carina straight, sub-parallel. Calyptra, in lateral view, elongate. Propodeum relatively short, not drawn out posteriorly. Calyptra, in posterior view, dorsoventrally elongate.

*Legs*. Pubescence posterolaterally on metacoxa sparse to moderately dense, confined dense hair patch absent. Microsculpture on hind coxa absent. Longitudinal carina on the posterior surface of metatibia absent. Metafemoral spine present, elongate, extending distally as low keel along ventral femoral margin. Distal mesotibial spurs equal in length to medial spurs. Distal metatibial spurs equal in length to medial spurs. Ratio of first metatibial segment to remaining 4 segments equal to 1.0. Pubescence on outer surface of metatarsal claw sparse, consisting of few setae. Outer surface of metatarsal claw microcarinate. Apical seta of metatarsal claw positioned on outer surface below dorsal margin. Base of metatarsal claw weakly expanded, apex slightly bent, ratio width of base to length of apex < 0.6.

*Forewing*. Pubescence of forewing absent on basal half of wing, sparse distally. Apical margin of female forewing rounded. Rs+M of forewing tubular. Mesal end of Rs+M vein situated closer to posterior margin of forewing, directed towards posterior end of basalis. Vein R1 tubular along at least basal part of anterior margin of marginal cell. Basal abscissa of R1 (the abscissa between 2r and the forewing margin) of forewing as broad as adjacent wing veins. Forewing entirely lightly infuscate. Marginal cell of forewing membranous, similar to other wing cells. Areolet absent. Hair fringe along apical margin of forewing absent.

*Petiole*. Slightly elongate, 1.5–2× longer than wide. Surface of petiole longitudinally costate, ventral keel absent. Posterior part of female petiole not abruptly widened. Ventral flange of annulus of female petiole absent.

*Metasoma*. Setal band (hairy ring) at base of tergum 3 absent, base of metasoma glabrous. Tergum 3 distinctly smaller than tergum 4. Posterior margin of tergum 3 smoothly rounded. Posterior margin of tergum 4 arcuate. In lateral view, sternum 3 exposed, ventral border of T2–T7 visible. Sculpture on metasomal terga present, dorsally with broad setal pits, ventrally with dense, fine punctation. Syntergum absent, all postpetiolar terga free. Annulus absent. Peg-like setae on T6–T7 absent. Posteroventral cavities of female metasoma T7 present, glabrous save for few, long setae. Female posteroventral margin of T6–T7 gently sinuate. Terebrum and hypopygium (in lateral view) straight, pointing posteriorly.

*Ovipositor*. First valvula of ovipositor narrowing gradually, not broadened apically, serrate at tip. Ovipositor clip absent.

**Figure 33.**
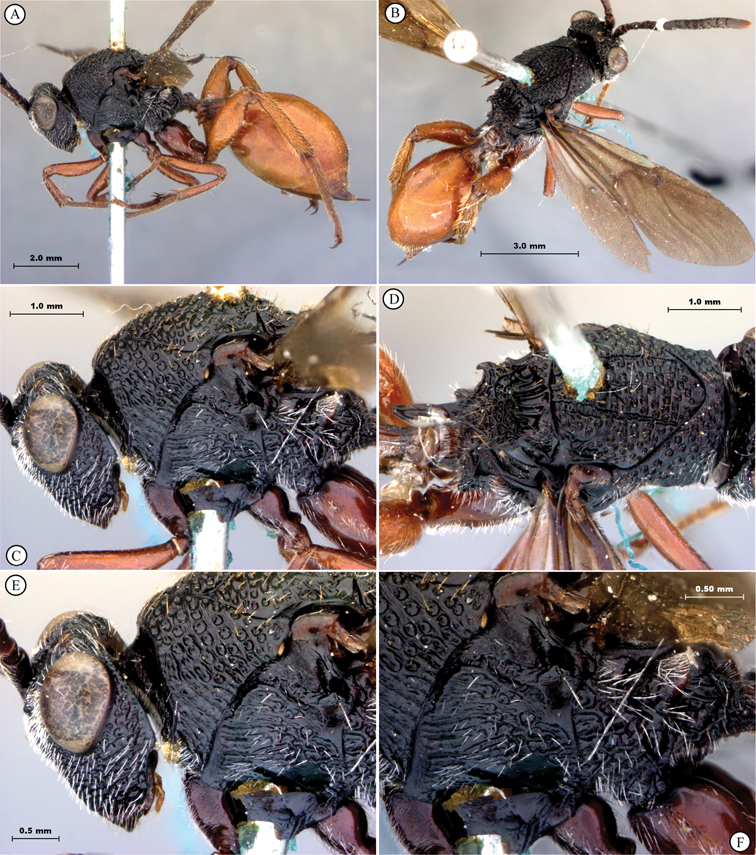
*Oberthuerella tibialis* Kieffer, holotype **A** lateral habitus **B** dorsal habitus **C** head and mesosoma, lateral view **D** mesosoma, dorsal view **E** head and pronotum, lateral view **F** meso- and metapleurae.

**Figure 34.**
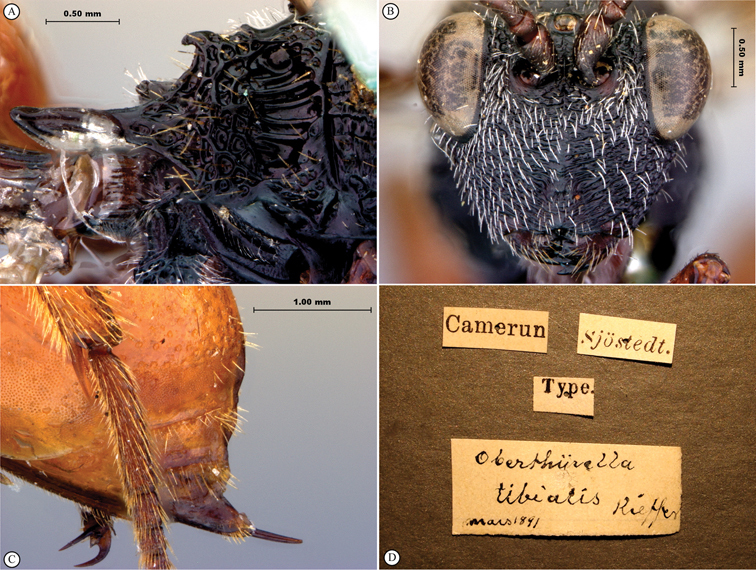
*Oberthuerella tibialis* Kieffer, holotype **A** scutellum, dorsal view **B** head, anterior view **C** posterior margin of metasoma, lateral view **D** labels.

##### Diagnosis.

This species is most easily confused with *Oberthuerella pardolatus*, but can seperated from that species by: 5 subfovea present at base of scutellum (7 in *Oberthuerella pardolatus*); admedial lines indistinct (distinct in *Oberthuerella pardolatus*); mesoscutum dominated by very shallow fovea (deep and distinct in *Oberthuerella pardolatus*).

##### Distribution.

Cameroon, South Africa; Zimbabwe. **Link to Distribution Map.** [http://hol.osu.edu/map-full.html?id=181561]

##### Material examined.

Holotype, female: **CAMEROON:** no date, Sjostedt, USNM ENT 00764779 (deposited in NHRS). *Other material*: (2 females, 1 male) **SOUTH AFRICA:** KwaZulu-Natal Prov., Sodwana Nature Reserve, 11.XI.1984, H. Howden (1 female, USNM ENT 00764777 (CNCI)). Transvaal, no date, Junod (1 male, USNM ENT 00764778 (MHNG)). **ZIMBABWE:** Harare (Salisbury), 21.I.1915 (1 female, SAM-HYM-P003004 (SAMC)).

#### Oberthuerella transiens

(Benoit)

urn:lsid:biosci.ohio-state.edu:osuc_concepts:181562

Morphbank accession: 704819–704840

http://www.waspweb.org/Cynipoidea/Liopteridae/Oberthuerellinae/Oberthuerella/Oberthuerella_transiens.htm

http://species-id.net/wiki/Oberthuerella_transiens

Figures 35 –36

Tessmannella transiens Benoit, 1955: 283. New combination by Ronquist (1995).

##### Description.

Coloration of head, mesosoma, and metasoma black to dark brown; legs reddish brown. Sculpture on vertex, lateral surface of pronotum and mesoscutum present, deeply striate on head, costate with foveae on pronotum, mesoscutum.

*Head*. Broadly triangular, in anterior view. Pubescence on head present, sparse setae scattered over head. Sculpture along lateral margin of occiput absent. Gena (measured from compound eye to posterolateral margin of head) short, ratio of length of gena to length of compound eye in dorsal view < 0.3, in dorsal view. Sculpture of gena deeply striate with remnants of fovea. Lateral margin of occiput defined by distinctly angled, raised, sharp carina. Occiput (except extreme lateral margin) smooth. Ocelli small, ratio of maximum diameter of a lateral ocellus to shortest distance between lateral ocelli 0.2–0.4. Anterior ocellus close to posterior ocelli, posterior margin of anterior ocellus behind or subcontiguous with a transverse line running through anterior margins of posterior ocelli. Relative position of toruli close to ocelli, ratio of vertical distance between inner margin of torulus and ventral margin of clypeus to vertical distance between anterior ocellus and torulus < 2.0. Median keel of face present, extending to middle of face, not reaching clypeus. Vertical carina adjacent to ventral margin of torulus absent. Facial sculpture almost entirely foveate, slightly horizontally striate along median keel. Facial impression absent, face flat. Antennal scrobe absent. Anterior tentorial pits large. Vertical delineations on lower face absent. Ventral clypeal margin laterally, close to anterior mandibular articulation, straight. Ventral clypeal margin medially emarginate. Clypeus horizontally striate. Malar space adjacent to anterior articulation of mandible evenly rounded, striate. Malar sulcus absent. Compound eye close to posterior ocellus, ratio of distance between compound eye and posterior mandibular articulation to distance between posterior ocellus and compound eye > 1.2. Compound eye, in dorsal view, distinctly protruding from the surface of the head, particularly laterally. Pubescence on compound eye absent. Orbital furrows absent. Lateral frontal carina of face absent. Dorsal aspect of vertex deeply foveate. Posterior aspect of vertex foveate. Hair punctures on lateral aspect of vertex absent. Posterior surface of head almost flat, not deeply impressed.

*Labial-maxillary complex*. Apical segment of maxillary palp with pubescence, consisting only of erect setae. Apical seta on apical segment of maxillary palp shorter than twice length of second longest apical seta. Erect setae medially on apical segment of maxillary palp present. Last two segments of maxillary palp (in normal repose) straight. Apical segment of maxillary palp more than 1.5 times as long as preceding segment.

*Antenna*. Articulation between flagellomeres in antenna connate with articles broadly joined. Male antenna composed of 12 flagellomeres. Placoidal sensilla absent. Second flagellomere of male antenna cylindrical. Length of second flagellomere of male antenna longer than first flagellomere.

*Pronotum*. Macrosculpture on lateral surface of pronotum present, dorsomedially foveate, laterally foveate-costate. Pubescence on lateral surface of pronotum present, sparse, composed of few short hairs. Anterior flange of pronotal plate distinctly protruding anteriorly, centrally smooth, longitudinally striate laterally. Carinae extending posteriorly from lateral margin of pronotal plate absent. Lateral pronotal carina present. Pronotal crest absent. Dorsal margin of pronotal plate (in anterior view) straight. Submedian pronotal depressions closed laterally, deep. Lateral margin of pronotal plate defined all the way to the dorsal margin of the pronotum. Pronotal plate wide, almost as wide as head.

*Mesoscutum*. Mesoscutal surface convex, evenly curved. Sculpture on mesoscutum present, foveate-punctate, with remnants of transverse costae. Notaulus present, marked by series of deep subcontiguous pits of uniform width. Median mesoscutal carina absent. Anterior admedial lines present, with adjacent cuticular surface horizontally striate. Median mesoscutal impression present, long, reaching over 1/2 length of mesoscutum. Parascutal carina distinctly sinuate, posteriorly ending in posteroventrally directed projection.

*Mesopleuron*. Horizontally strigulate, with striae converging along posterior margin of sclerite. Subpleuron entirely smooth with few long white setae anteriorly, posteriorly. Lower mesopleuron micro-pitted anteriorly, smooth and glabrous posteriorly. Epicnemial carina present, running from mesoscutum to anterior margin of mesopleural carina, narrow ventrally, costate. Lateroventral mesopleural carina present, not marking abrupt change of slope of mesopectus. Mesopleural triangle absent. Subalar pit large and well defined, lying in posterior end of subalar groove. Speculum present, microcarinate. Mesopleural carina present, complete, composed of several long, parallel, straight carinae. Anterior end of mesopleural carina inserting above notch in anterior margin of mesopleuron.

*Scutellum*. Dorsal surface of scutellum foveate-areolate. Circumscutellar carina absent. Posterior margin of axillula marked by distinct ledge, axillula distinctly impressed adjacent to ledge. Lateroventral margin of scutellum posterior to auricula smooth ventrally, obliquely longtidinally striate dorsally, entirely striate posteriorly. Dorsoposterior part of scutellum produced posteriorly into sharp spine, less than 1.0× length of petiole. Dorsal part of scutellum entirely rugose. Scutellar plate absent. Scutellar foveae present, three, with lateral foveal bissected by longitudinal carina, resulting in five longitudinally elongate subfovea. Longitudinal scutellar carinae absent. Single longitudinal carina separating scutellar foveae absent. Posterolateral margin of scutellum drawn out into distinct protuberance. Lateral Lateral bar narrow, with strong strigate, foveate sculpture.

*Metapectal-propodeal complex*. Metapectal cavity anterodorsal to metacoxal base present, ill-defined. Anterior margin of metapectal-propodeal complex separated from mesopleuron by deep, broad, uninterrupted marginal impression. Posteroventral corner of metapleuron (in lateral view) rounded, not drawn out posteriorly. Anterior impression of metepimeron present, narrow, linear impression, not broadened ventrally. Posterior margin of metepimeron distinct, separating metepimeron from propodeum. Subalar area slightly broadened anteriorly, without longitudinal division indicated. Calyptra present, blunt, lobe-like, polished posteriorly with setiferous punctures anteriorly. Dorsellum present with two strong medial fovea, glabrous. Anterior impression of metepisternum, immediately beneath anterior end of metapleural carina, large and wide. Pubescence consisting of few scattered hairs on posterior part of metapleuron and lateral part of propodeum. Propodeal spurs present, crenulate. Lateral propodeal carinae present, not reaching scutellum. Ventral end of lateral propodeal carina reaching nucha, carinae separated from each other. Inter propodeal carinae space glabrous with bifurcating central carina, foveate. Petiolar foramen removed from metacoxae, directed posteriorly. Horizontal carina running anteriorly from lateral propodeal carina present. Lateral propodeal carina uniformly curved inward. Calyptra, in lateral view, elongate. Propodeum relatively short, not drawn out posteriorly. Calyptra, in posterior view, rounded.

*Legs*. Pubescence posterolaterally on metacoxa sparse to moderately dense, confined dense hair patch absent. Microsculpture on hind coxa absent. Longitudinal carina on the posterior surface of metatibia absent. Metafemoral spine present, elongate, extending distally as low keel along ventral femoral margin. Distal mesotibial spurs shorter than medial spurs. Distal metatibial spurs equal in length to medial spurs. Ratio of first metatibial segment to remaining 4 segments less than 1.0. Pubescence on outer surface of metatarsal claw dense, consisting of numerous setae. Outer surface of metatarsal claw entirely smooth. Apical seta of metatarsal claw positioned on outer surface below dorsal margin. Base of metatarsal claw weakly expanded, apex slightly bent, ratio width of base to length of apex < 0.6.

*Forewing*. Pubescence of forewing absent on basal half of wing, sparse distally. Apical margin of female forewing rounded. Rs+M of forewing tubular. Mesal end of Rs+M vein situated closer to posterior margin of forewing, directed towards posterior end of basalis. Vein R1 tubular along at least basal part of anterior margin of marginal cell. Basal abscissa of R1 (the abscissa between 2r and the forewing margin) of forewing as broad as adjacent wing veins. Coloration of forewing hyaline with slight infuscation covering marginal cell, area posterior to marginal cell. Marginal cell of forewing membranous, similar to other wing cells. Areolet absent. Hair fringe along apical margin of forewing absent.

*Petiole*. Distinctly elongate, >5–6× longer than broad. Surface of petiole longitudinally costate, ventral keel absent.

*Metasoma*. Setal band (hairy ring) at base of tergum 3 absent, base of metasoma glabrous. Tergum 3 distinctly smaller than tergum 4. Posterior margin of tergum 3 smoothly rounded. Posterior margin of tergum 4 arcuate. In lateral view, sternum 3 exposed, ventral border of T2–T7 visible. Sculpture on metasomal terga present, finely punctate laterally, dorsally; posteriorly with large setal pits. Syntergum absent, all postpetiolar terga free. Annulus absent. Peg-like setae on T6–T7 absent.

**Figure 35.**
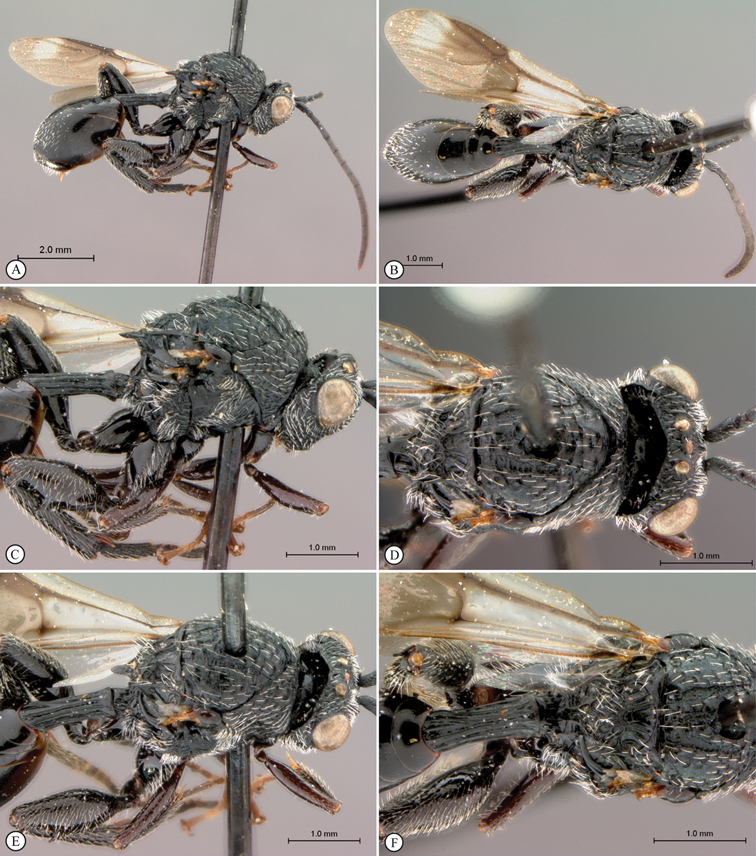
*Oberthuerella transiens* (Benoit), holotype **A** lateral habitus **B** dorsal habitus **C** head and mesosoma, lateral view **D** head and mesosoma, dorsal view **E** head and mesosoma, dorsolateral view **F** scutellum and petiole, dorsal view.

**Figure 36.**
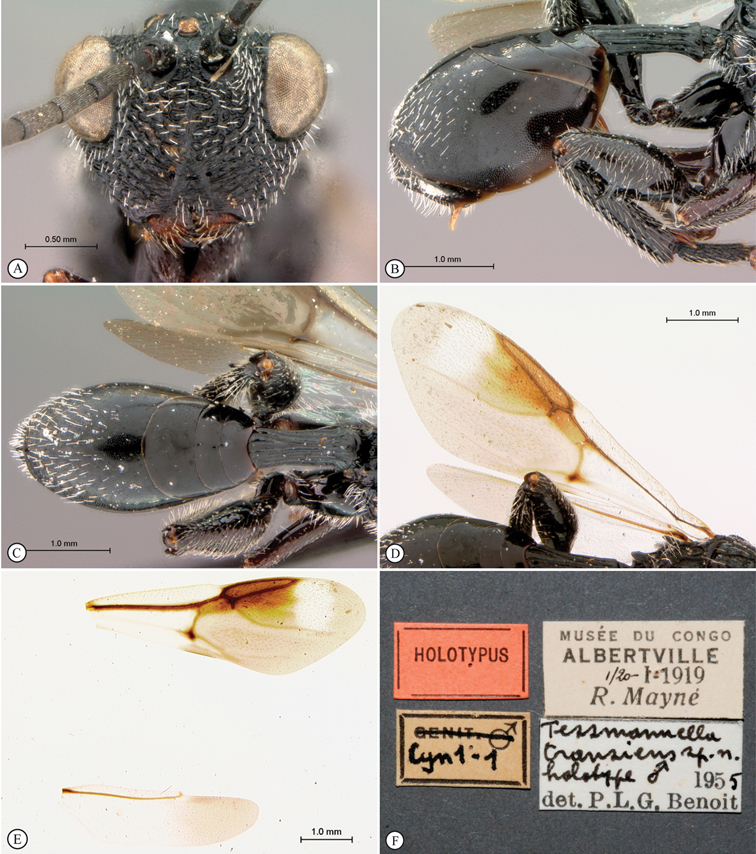
*Oberthuerella transiens* (Benoit), holotype **A** head, anterior view **B** metasoma, lateral view **C** metasoma, dorsal view **D** fore and hind wings **E** fore and hind wings **F** labels.

##### Diagnosis.

The petiole on this species is unusually long, about 3x longer than its width; other species of *Oberthuerella* have petioles 2× longer than wide, or less. In addition, *Oberthuerella transiens* has light and dark patches on the forewing, rather than entirely infuscate or entirely hyaline as in other species.

##### Distribution.

Democratic Republic of Congo. **Link to Distribution Map.** [http://hol.osu.edu/map-full.html?id=181562]

##### Material examined.

Holotype, male, *Tessmannella transiens*: **DEMOCRATIC REPUBLIC OF THE CONGO:** Kalemie (Albertville), 1.I-20.I.1919, R. Mayné, Mus. Cong. Cyn1-1 (deposited in MRAC).

#### Oberthuerella triformis

Quinlan

urn:lsid:biosci.ohio-state.edu:osuc_concepts:181564

http://www.waspweb.org/Cynipoidea/Liopteridae/Oberthuerellinae/Oberthuerella/Oberthuerella_triformis.htm

http://species-id.net/wiki/Oberthuerella_triformis

Figures 37 –38

Oberthuerella triformis Quinlan, 1979: 115.

##### Description.

Coloration of head and mesosoma black to dark brown; metasoma orange; fore- and midlegs black to dark brown, hindlegs with coxae dark brown, femora and tibia orange, tarsi dark brown.. Sculpture on vertex, lateral surface of pronotum and mesoscutum present, gently striate on vertex, pronotum, mesoscutum striate-foveate.

*Head*. Broadly triangular, in anterior view. Pubescence on head present, sparse setae scattered over head. Sculpture along lateral margin of occiput many costulae. Gena (measured from compound eye to posterolateral margin of head) short, ratio of length of gena to length of compound eye in dorsal view < 0.3, in dorsal view. Sculpture of gena deeply striate. Lateral margin of occiput defined by distinctly angled, raised, sharp carina. Occiput (except extreme lateral margin) with distinct subvertical, slightly and evenly curved costulae. Ocelli small, ratio of maximum diameter of a lateral ocellus to shortest distance between lateral ocelli 0.2–0.4. Anterior ocellus close to posterior ocelli, posterior margin of anterior ocellus behind or subcontiguous with a transverse line running through anterior margins of posterior ocelli. Relative position of toruli close to ocelli, ratio of vertical distance between inner margin of torulus and ventral margin of clypeus to vertical distance between anterior ocellus and torulus < 2.0. Median keel of face present, short, not extending beyond toruli. Vertical carina adjacent to ventral margin of torulus absent. Facial sculpture transversely striate with remnants of foveae. Facial impression absent, face flat. Antennal scrobe absent. Anterior tentorial pits small. Vertical delineations on lower face absent. Ventral clypeal margin laterally, close to anterior mandibular articulation, distinctly angled. Ventral clypeal margin medially emarginate. Clypeus horizontally striate. Malar space adjacent to anterior articulation of mandible evenly rounded, striate. Malar sulcus absent. Compound eye close to posterior ocellus, ratio of distance between compound eye and posterior mandibular articulation to distance between posterior ocellus and compound eye > 1.2. Compound eye, in dorsal view, distinctly protruding from the surface of the head, particularly laterally. Pubescence on compound eye absent. Orbital furrows absent. Lateral frontal carina of face absent. Dorsal aspect of vertex variously strigate. Posterior aspect of vertex with parallel or slightly radiating, longitudinal strigae. Hair punctures on lateral aspect of vertex present, distinctly enlarged. Posterior surface of head deeply impressed around postocciput.

*Labial-maxillary complex*. Apical segment of maxillary palp with pubescence, consisting only of erect setae. First segment of labial palp shorter than apical segment. Labial palp composed of three segments. Apical seta on apical segment of maxillary palp shorter than twice length of second longest apical seta. Erect setae medially on apical segment of maxillary palp present. Maxillary palp composed of four segments. Last two segments of maxillary palp (in normal repose) curved inwards. Distal margin of subapical segment of maxillary palp slanting inwards, apical segment bending inwards. Apical segment of maxillary palp more than 1.5× as long as preceding segment.

*Antenna*. Articulation between flagellomeres in antenna connate with articles broadly joined. Female antenna composed of 11 flagellomeres. Female F1 shorter than F2; black. Flagellomeres of female antenna cylindrical, not widened towards apex, non-clavate. Placoidal sensilla absent. Distal flagellomeres of female antenna not conspicuously enlarged compared to proximal flagellomeres.

*Pronotum*. Macrosculpture on lateral surface of pronotum present, deeply costulate. Pubescence on lateral surface of pronotum present, sparse, composed of few short hairs. Anterior flange of pronotal plate distinctly protruding anteriorly, longitudinally striate. Carinae extending posteriorly from lateral margin of pronotal plate absent. Lateral pronotal carina present. Pronotal crest absent. Dorsal margin of pronotal plate (in anterior view) rounded. Submedian pronotal depressions closed laterally, shallow. Lateral margin of pronotal plate defined all the way to the dorsal margin of the pronotum. Width of pronotal plate wide, almost as wide as head.

*Mesoscutum*. Mesoscutal surface convex, evenly curved. Sculpture on mesoscutum present, deeply transversely costate. Notaulus present, wide, transversely striate, distinctly wider posteriorly. Median mesoscutal carina absent. Anterior admedial lines present, with adjacent cuticular surface horizontally striate. Median mesoscutal impression present, long, reaching over 1/2 length of mesoscutum. Parascutal carina distinctly sinuate, posteriorly ending in posteroventrally directed projection.

*Mesopleuron*. Horizontally strigulate, with striae converging on remnant fovea along posterior margin of sclerite. Subpleuron entirely smooth with few long white setae along ventral, posterior margins. Lower mesopleuron medially smooth, setose; costate laterally, ventrally. Epicnemial carina present on ventral half of mesopleuron; shagreen, ventrally bulbous near mesosternum. Lateroventral mesopleural carina present, marking abrupt change of slope of mesopectus. Mesopleural triangle absent. Subalar pit large and well defined, lying in posterior end of subalar groove. Speculum present, striate, with distinct smooth, glabrous ventral cavity. Mesopleural carina absent.

*Scutellum*. Dorsal surface of scutellum foveate-areolet. Circumscutellar carina absent. Posterior margin of axillula marked by distinct ledge, axillula distinctly impressed adjacent to ledge. Latero-ventral margin of scutellum posterior to auricula entirely smooth. Dorsoposterior part of scutellum produced posteriorly into blunt spine. Dorsal part of scutellum entirely areolate. Scutellar plate absent. Scutellar foveae present, three, with lateral foveal bissected by longitudinal carina, resulting in five longitudinally elongate subfovea. Longitudinal scutellar carinae absent. Single longitudinal carina separating scutellar foveae absent. Postero-lateral margin of scutellum drawn out into distinct protuberance. Lateral bar with strong strigate sculpture, narrow.

*Metapectal-propodeal complex*. Metapectal cavity anterodorsal to metacoxal base absent. Anterior margin of metapectal-propodeal complex seperated from mesopleuron by distinct dorso-ventral ledge. Posteroventral corner of metapleuron (in lateral view) rounded, not drawn out posteriorly. Anterior impression of metepimeron absent. Posterior margin of metepimeron distinct, separating metepimeron from propodeum. Subalar area slightly broadened anteriorly, without longitudinal division indicated. Calyptra present, blunt, lobe-like, polished posteriorly with setiferous punctures anteriorly. Dorsellum present with two strong medial fovea, glabrous. Anterior impression of metepisternum, immediately beneath anterior end of metapleural carina, absent. Pubescence consisting of few scattered hairs on posterior part of metapleuron and lateral part of propodeum. Propodeal spurs present, foveate. Lateral propodeal carinae present, not reaching scutellum. Ventral end of lateral propodeal carina terminating before reaching nucha. Inter propodeal carinae space glabrous, costulate. Petiolar foramen removed from metacoxae, directed posteriorly. Horizontal carina running anteriorly from lateral propodeal carina present. Lateral propodeal carina distinctly angled. Calyptra, in lateral view, rounded. Propodeum relatively short, not drawn out posteriorly. Calyptra, in posterior view, rounded.

*Legs*. Pubescence posterolaterally on metacoxa sparse to moderately dense, confined dense hair patch absent. Microsculpture on hind coxa absent. Longitudinal carina on the posterior surface of metatibia absent. Metafemoral spine present, elongate, extending distally as low keel along ventral femoral margin. Distal mesotibial spurs shorter than medial spurs. Distal metatibial spurs longer than medial spurs. Ratio of first metatibial segment to remaining 4 segments greater than 1.0. Pubescence on outer surface of metatarsal claw sparse, consisting of few setae. Outer surface of metatarsal claw microcarinate. Apical seta of metatarsal claw positioned on outer surface below dorsal margin. Base of metatarsal claw strongly expanded, apex strongly bent, ratio width of base to length of apex > 0.6; lammelate, with translucent cuticular flange.

*Forewing*. Pubescence of forewing present, sparse across entire wing surface. Apical margin of female forewing rounded. Rs+M of forewing tubular. Mesal end of Rs+M vein situated closer to posterior margin of forewing, directed towards posterior end of basalis. Vein R1 tubular along at least basal part of anterior margin of marginal cell. Basal abscissa of R1 (the abscissa between 2r and the forewing margin) of forewing as broad as adjacent wing veins. Coloration of forewing entirely lightly infuscate. Marginal cell of fore wing membranous, similar to other wing cells. Areolet absent. Hair fringe along apical margin of forewing absent.

*Petiole*. About as long as wide. Surface of petiole longitudinally costate, ventral keel absent. Posterior part of female petiole not abruptly widened. Ventral flange of annulus of female petiole absent.

*Metasoma*. Setal band (hairy ring) at base of tergum 3 absent, base of metasoma glabrous. Tergum 3 distinctly smaller than tergum 4. Posterior margin of tergum 3 smoothly rounded. Posterior margin of tergum 4 arcuate. In lateral view, sternum 3 encompassed by T6. Sculpture on metasomal terga present, dorsally finely punctate, posteriorly with distinct bands of setiferous pits. Syntergum absent, all postpetiolar terga free. Annulus absent. Peg-like setae on T6–T7 present. Postero-ventral cavities of female metasoma T7 present, setose. Female posteroventral margin of T6–T7 distinctly sinuate. Terebrum and hypopygium (in lateral view) straight, pointing posteriorly.

*Ovipositor*. First valvula of ovipositor narrowing gradually, not broadened apically, serrate at tip. Ovipositor clip absent.

**Figure 37.**
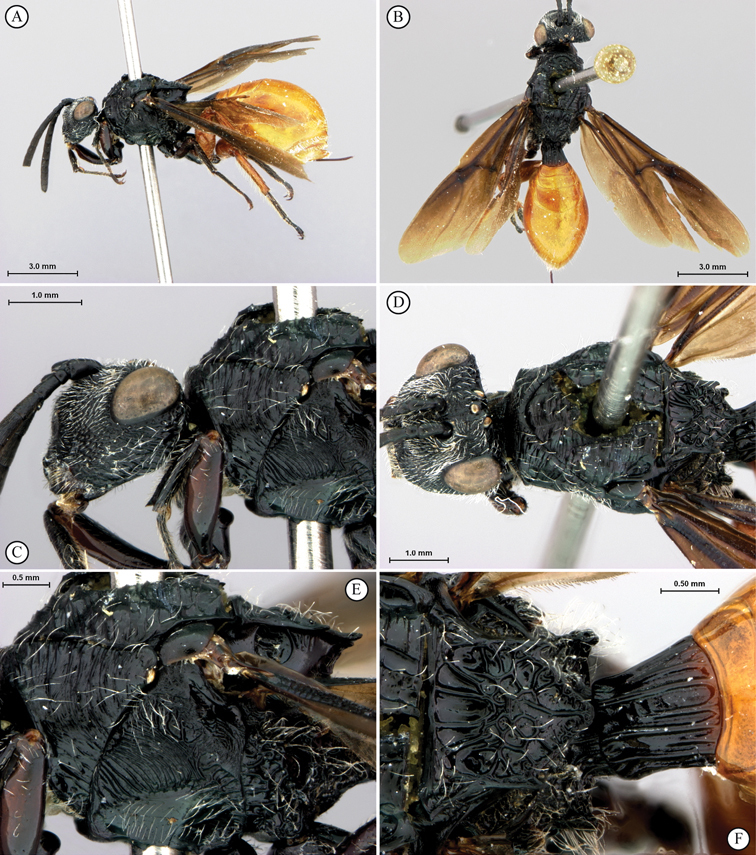
*Oberthuerella triformis* Quinlan, holotype **A** lateral habitus **B** dorsal habitus **C** head and mesosoma, lateral view **D** head and mesosoma, dorsal view **E** mesosoma, lateral view **F** scutellum and petiole, dorsal view.

**Figure 38.**
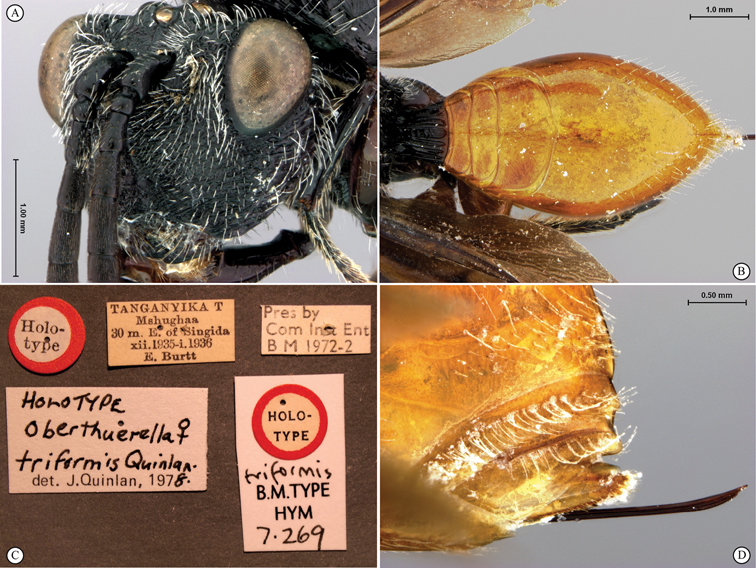
*Oberthuerella triformis* Quinlan, holotype **A** head, anterolateral view **B** metasoma, dorsal view **C** holotype labels **D** metasoma and ovipositor, lateral view.

##### Diagnosis.

Distinguished from other species of *Oberthuerella* by the extremely short median keel of the face, not reaching beyond the toruli; this is shared only with *Oberthuerella lenticularis*, but differs from this latter species by the very short scutellar spine, much shorter than the petiole (as long as the petiole in *Oberthuerella lenticularis*).

##### Distribution.

Tanzania. **Link to Distribution Map.** [http://hol.osu.edu/map-full.html?id=181564]

##### Material examined.

Holotype, female: **TANZANIA:** Singida Reg., Misughaa (Mshughaa), 30mi E Singida, XII-1935 - I-1936, E. Burtt, BMNH HT 0010 (deposited in BMNH).

#### Tessmannella

Hedicke

http://www.waspweb.org/Cynipoidea/Liopteridae/Oberthuerellinae/Tessmannella/index.htm

http://species-id.net/wiki/Tessmannella

Tessmannella Hedicke, 1912: 303. Type species: *Tessmannella spinosa* Hedicke, by original designation.

##### Diagnosis.

Female antenna 13-segmented, subclavate; male 14-segmented. Face with reticulate to rugose sculpture and scattered pubescence. Pronotum coarsely rugose with median tooth or spine viewed laterally. Mesonotum with coarse variable sculpture, propodeum without pronounced side margins. Petiole three times as long as broad, terga 2–4 short (viewed laterally and dorsally), terga 5 largest. Metafemora with a rounded lobe between medial area and apex, tooth on metafemur angled, hind tibia with a distinct lobe apically, opposite the tibial spines. Scutellum with three foveae.

##### Distribution.

Central African Republic; Congo; Democratic Republic of Congo (Zaire); Equatorial Guinea; Gabon; Kenya.

##### Biology.

Unknown.

##### Comments.

Only four species were previously known in this genus, two described by Hedicke (1912), one by Benoit (1955), and one by Quinlan (1979). *Tessmannella transiens* Benoit (1955) was transferred to *Oberthuerella* by Ronquist (1995) leaving three species in the genus. Here we describe three new species, elevating the total to six.

##### Included species.

*Tessmannella copelandi* Buffington & van Noort, sp. n.

*Tessmannella expansa* Quinlan, 1979: 116

*Tessmannella kiplingi* Buffington & van Noort, sp. n.

*Tessmannella nigra* Hedicke, 1912: 304

*Tessmannella roberti* Buffington & van Noort, sp. n.

*Tessmannella spinosa* Hedicke, 1912: 303

##### Key to species of *Tessmannella* (both sexes)

(Available online at http://www.waspweb.org/Cynipoidea/Keys/index.htm)

**Table:** 

	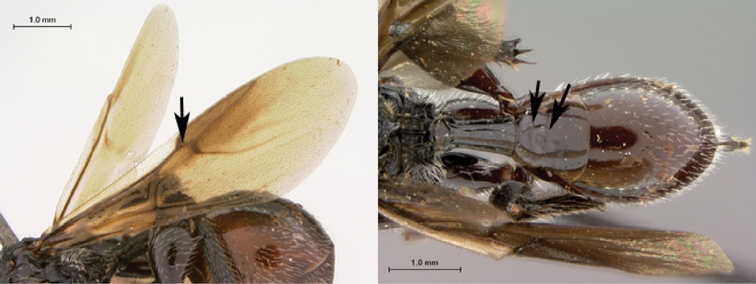
1A	Forewing uniformly infuscate; posterior margins of T3 and T4 broadly emarginate	2
	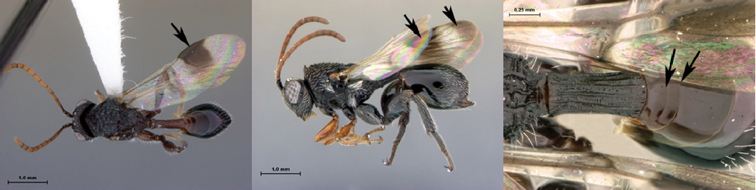
1B	Forewing basally hyaline, with either one or two infuscate patches posterior and distal to the marginal cell; posterior margins of T3 and T4 straight, not emarginate	4
	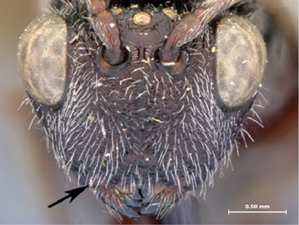
2A	Ventral malar margin, immediately adjacent to mandibular base, distinctly striate	3
	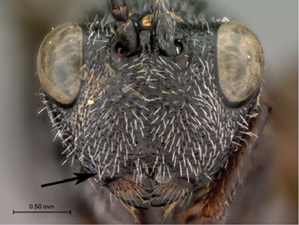
2B	Ventral malar margin, immediately adjacent to mandibular base, gently shagreened	*Tessmannella nigra* Hedicke
	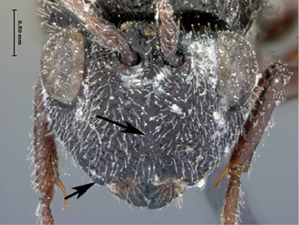
3A	Distinct clypeo-pleurostomal line present; face shagreened medially	*Tessmannella spinosa* Hedicke
	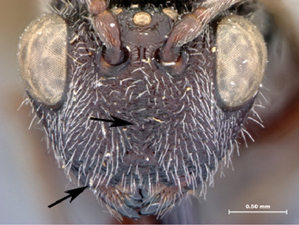
3B	Clypo-pleurostomal line absent; face horizontally striate medially	*Tessmannella expansa* Quinlan
	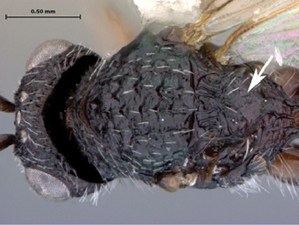
4A	Central scutellar area smooth, with a very faint central carina	*Tessmannella copelandi* sp. n.
	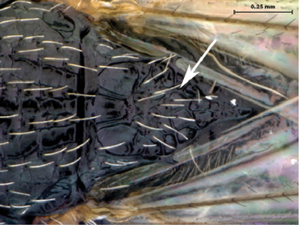
4B	Central scutellar area areolate to foveate	5
	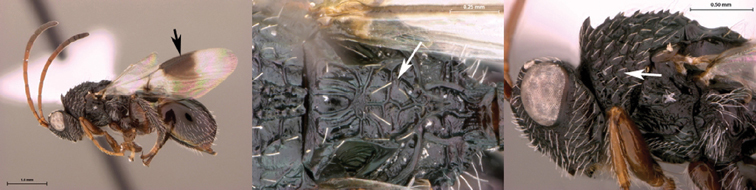
5A	Forewing with single distinct infuscate patch throughout marginal cell and immediately posterior; central scutellar area areolate; dorsal and lateral pronotum areolate	*Tessmannella roberti* sp. n.
	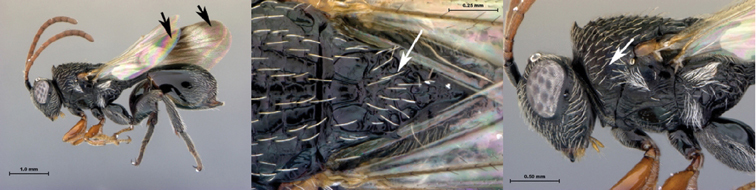
5B	Forewing with two infuscate patches, one including the marginal cell and space immediately posterior, and a second faint patch that encompasses the entire distal margin of the forewing (leaving a small, hyaline area between the distal margin and the marginal cell); central scutellar area foveolate; dorsal and lateral margins of pronotum horizontally striate, resulting in a ‘wave-like' texture	*Tessmannella kiplingi* sp. n.

#### Tessmannella copelandi

Buffington & van Noort sp. n.

urn:lsid:zoobank.org:act:4AC8FB6A-F43C-4F26-9EB7-ECBDCF06BAF3

urn:lsid:biosci.ohio-state.edu:osuc_concepts:300221

Morphbank accession: 704841–704850

http://www.waspweb.org/Cynipoidea/Liopteridae/Oberthuerellinae/Tessmannella/Tessmannella_copelandi.htm

http://species-id.net/wiki/Tessmannella_copelandi

Figures 39 –40

##### Description.

Coloration of head, mesosoma, and metasoma, dark reddish brown; legs reddish brown. Sculpture on vertex, lateral surface of pronotum and mesoscutum present, deeply foveate laterally on head, pronotum; deeply horizontally striate on mesoscutum.

*Head*. Broadly triangular, in anterior view. Pubescence on head present, sparse setae scattered over head. Sculpture along lateral margin of occiput absent. Gena (measured from compound eye to posterolateral margin of head) short, ratio of length of gena to length of compound eye in dorsal view < 0.3, in dorsal view. Sculpture of gena present, with distinct fovea. Lateral margin of occiput defined by distinctly angled, raised, sharp carina. Occiput (except extreme lateral margin) smooth. Ocelli small, ratio of maximum diameter of a lateral ocellus to shortest distance between lateral ocelli 0.2–0.4. Anterior ocellus close to posterior ocelli, posterior margin of anterior ocellus behind or subcontiguous with a transverse line running through anterior margins of posterior ocelli. Relative position of toruli close to ocelli, ratio of vertical distance between inner margin of torulus and ventral margin of clypeus to vertical distance between anterior ocellus and torulus < 2.0. Median keel of face present, extending to middle of face, not reaching clypeus. Vertical carina adjacent to ventral margin of torulus present. Facial sculpture almost entirely foveate, slightly horizontally striate along median keel. Facial impression absent, face flat. Antennal scrobe absent. Anterior tentorial pits large. Vertical delineations on lower face absent. Ventral clypeal margin laterally, close to anterior mandibular articulation, distinctly angled. Ventral clypeal margin medially emarginate. Clypeus smooth, evenly rounded. Malar space adjacent to anterior articulation of mandible evenly rounded, striate-foveate. Malar sulcus absent. Compound eye close to posterior ocellus, ratio of distance between compound eye and posterior mandibular articulation to distance between posterior ocellus and compound eye > 1.2. Compound eye, in dorsal view, distinctly protruding from the surface of the head, particularly laterally. Pubescence on compound eye absent. Orbital furrows absent. Lateral frontal carina of face absent. Dorsal aspect of vertex deeply foveate. Posterior aspect of vertex foveate. Hair punctures on lateral aspect of vertex present, indistinct. Posterior surface of head almost flat, not deeply impressed.

*Labial-maxillary complex*. Apical segment of maxillary palp with pubescence, consisting only of erect setae. Apical seta on apical segment of maxillary palp shorter than twice length of second longest apical seta. Erect setae medially on apical segment of maxillary palp present. Maxillary palp composed of four segments. Last two segments of maxillary palp (in normal repose) straight. Distal margin of subapical segment of maxillary palp straight, apical segment bending outwards. Apical segment of maxillary palp more than 1.5 times as long as preceding segment.

*Antenna*. Articulation between flagellomeres in antenna connate with articles broadly joined. Female antenna composed of 11 flagellomeres. Female F1 shorter than F2; black. Flagellomeres of female antenna cylindrical, not widened towards apex, non-clavate. Placoidal sensilla absent. Distal flagellomeres of female antenna not conspicuously enlarged compared to proximal flagellomeres.

*Pronotum*. Macrosculpture on lateral surface of pronotum present, dorsomedially foveate, laterally foveate-costate. Pubescence on lateral surface of pronotum present, sparse, composed of few short hairs. Anterior flange of pronotal plate distinctly protruding anteriorly, longitudinally striate. Carinae extending posteriorly from lateral margin of pronotal plate absent. Lateral pronotal carina present. Pronotal crest present, raised into a distinct process projecting above anterior margin of mesoscutum. Submedian pronotal depressions absent, represented by shallow depression. Lateral margin of pronotal plate defined all the way to the dorsal margin of the pronotum. Pronotal plate wide, almost as wide as head.

*Mesoscutum*. Mesoscutal surface convex, evenly curved. Sculpture on mesoscutum present, transversely costate with dorsally projected serrations. Notaulus present, wide, transversely striate, distinctly wider posteriorly. Median mesoscutal carina absent. Anterior admedial lines present, with adjacent cuticular surface smooth. Median mesoscutal impression present, long, reaching over 1/2 length of mesoscutum. Parascutal carina nearly straight anteriorly, posteriorly curved mesally.

*Mesopleuron*. Dorsally with strigae running dorsoventrally; ventrally smooth, medially sparsely setose. Subpleuron anteriorly strigate, posteriorly smooth; medially with sparse, long setae. Lower mesopleuron medially smooth, glabrous; costate laterally, ventrally. Epicnemial carina absent. Lateroventral mesopleural carina present, marking abrupt change of slope of mesopectus. Mesopleural triangle present, distinctly impressed with distinct dorsal and ventral border, glabrous. Subalar pit large and well defined, lying in posterior end of mesopleural triangle. Speculum present, smooth anteriorly, microcarinate posteriorly. Mesopleural carina absent.

*Scutellum*. Dorsal surface of scutellum smooth centrally, peripherally areolate-punctate. Circumscutellar carina absent. Posterior margin of axillula marked by distinct ledge, axillula distinctly impressed adjacent to ledge. Lateroventral margin of scutellum posterior to auricula smooth ventrally, obliquely longitudinally striate dorsally, entirely striate posteriorly. Dorsoposterior part of scutellum produced posteriorly into sharp spine, less than 1.0× length of petiole. Dorsal part of scutellum entirely rugose. Scutellar plate absent. Scutellar foveae present, three, each lateral fovea with two longitudinal divisions, central fovea smooth, resulting in transverse row of 7 longitudinally elongate subfovea. Longitudinal scutellar carinae present as single central carina. Single longitudinal carina separating scutellar foveae absent. Posterolateral margin of scutellum drawn out into distinct protuberance. Lateral bar narrow, with strong strigate, foveate sculpture.

*Metapectal-propodeal complex*. Metapectal cavity anterodorsal to metacoxal base absent. Anterior margin of metapectal-propodeal complex seperated from mesopleuron by distinct dorso-ventral ledge. Posteroventral corner of metapleuron (in lateral view) extended posteriorly. Anterior impression of metepimeron present, triangular, with broadest part ventrally. Posterior margin of metepimeron distinct, separating metepimeron from propodeum. Subalar area slightly broadened anteriorly, without longitudinal division indicated. Calyptra present, blunt, lobe-like, polished. Dorsellum absent. Anterior impression of metepisternum, immediately beneath anterior end of metapleural carina, large and wide. Pubescence consisting of few scattered hairs on posterior part of metapleuron and lateral part of propodeum. Propodeal spurs absent. Lateral propodeal carinae present, not reaching scutellum. Ventral end of lateral propodeal carina reaching nucha, carinae separated from each other. Inter propodeal carinae space glabrous, costulate. Petiolar foramen removed from metacoxae, directed posteriorly. Horizontal carina running anteriorly from lateral propodeal carina absent. Lateral propodeal carina straight, sub-parallel. Calyptra, in lateral view, elongate. Propodeum ‘neck-like', drawn out posteriorly. Calyptra, in posterior view, dorsoventrally elongate.

*Legs*. Pubescence posterolaterally on metacoxa sparse to moderately dense, confined dense hair patch absent. Microsculpture on hind coxa absent. Longitudinal carina on the posterior surface of metatibia absent. Metafemoral spine present, elongate, extending distally as low keel along ventral femoral margin. Distal mesotibial spurs shorter than medial spurs. Distal metatibial spurs shorter than medial spurs. Ratio of first metatibial segment to remaining 4 segments less than 1.0. Pubescence on outer surface of metatarsal claw sparse, consisting of few setae. Outer surface of metatarsal claw almost entirely smooth. Apical seta of metatarsal claw positioned on outer surface below dorsal margin. Base of metatarsal claw weakly expanded, apex slightly bent, ratio width of base to length of apex < 0.6.

*Forewing*. Pubescence of forewing absent on basal half of wing, sparse distally. Apical margin of female forewing rounded. Rs+M of forewing tubular. Mesal end of Rs+M vein situated closer to posterior margin of forewing, directed towards posterior end of basalis. Vein R1 tubular along at least basal part of anterior margin of marginal cell. Basal abscissa of R1 (the abscissa between 2r and the forewing margin) of forewing as broad as adjacent wing veins. Coloration of forewing hyaline with slight infuscation covering marginal cell, area posterior to marginal cell. Marginal cell of forewing membranous, similar to other wing cells. Areolet absent. Hair fringe along apical margin of forewing absent.

*Petiole*. Distinctly elongate, >5–6× longer than broad. Surface of petiole longitudinally costate, ribbed, ventral keel absent. Posterior part of female petiole not abruptly widened. Ventral flange of annulus of female petiole absent.

*Metasoma*. Setal band (hairy ring) at base of tergum 3 absent, base of metasoma glabrous. Tergum 3 distinctly smaller than tergum 4. Posterior margin of tergum 3 smoothly rounded. Posterior margin of tergum 4 evenly rounded. In lateral view, sternum 3 exposed, ventral border of T2–T7 visible. Sculpture on metasomal terga present, dorsally smooth, posteroventrally micropunctate. Syntergum absent, all postpetiolar terga free. Annulus absent. Peg-like setae on T6–T7 absent. Posteroventral cavities of female metasoma T7 present, glabrous save for few, long setae. Female posteroventral margin of T6–T7 straight, parallel, with medial carina. Terebrum and hypopygium (in lateral view) straight, pointing posteriorly.

**Figure 39.**
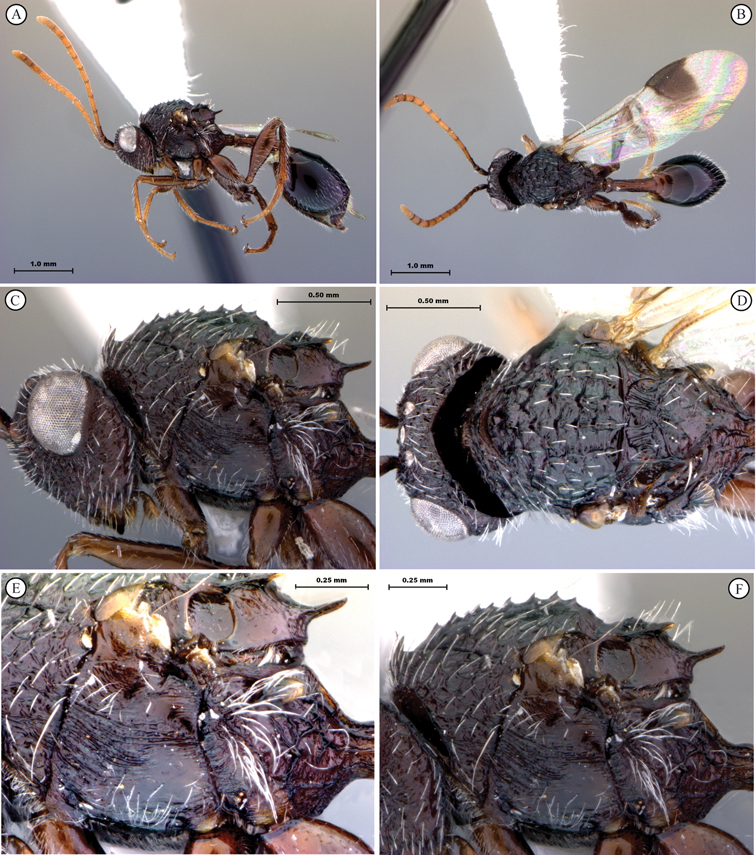
*Tessmannella copelandi* Buffington & van Noort, sp. n., holotype **A** lateral habitus **B** dorsal habitus **C** head and mesosoma, lateral view **D** head and mesosoma, dorsal view **E** meso- and metapleurae, scutellum, lateral view **F** mesosoma, lateral view.

**Figure 40.**
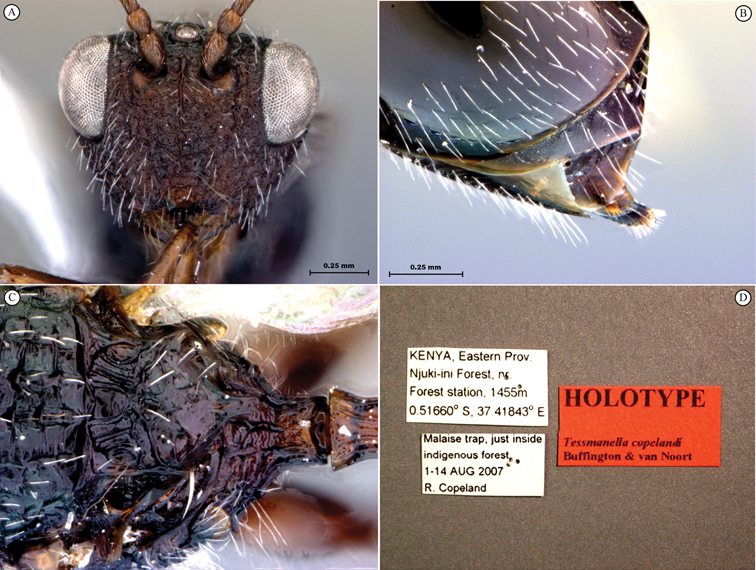
*Tessmannella copelandi* Buffington & van Noort, sp. n., holotype **A** head, anterior view **B** hind margin of metasoma, lateral view **C** scutellum and petiole, dorsal view **D** labels.

##### Diagnosis.

This species is similar to *Tessmannella kiplingi* and *Tessmannella roberti*, which have the forewing basally hyaline and the posterior margins of metasoma T3–T4 straight. This species can be distinguished from *Tessmannella kiplingi* and *Tessmannella roberti* by having the central area of the scutellum completely smooth (areolate to foveate in the latter species).

##### Etymology.

Named after our friend and acclaimed African entomologist Robert Copeland (ICIPE, Kenya), collector of the type series for this species.

##### Distribution.

Kenya. **Link to Distribution Map.** [http://hol.osu.edu/map-full.html?id=300221]

##### Material examined.

Holotype, female: **KENYA:** Central Prov., nr. forest station, indigenous forest margin, Njukini Forest, 0.51660°S, 37.41843°E, 1455m, 1.VIII–14.VIII.2007, malaise trap, R. Copeland, USNM ENT 00764801 (deposited in NMKE). *Paratypes*: (2 females) **KENYA:** Central Prov., nr. forest station, indigenous forest margin, Njukini Forest, 0.51660°S, 37.41843°E, 1455m, 19.VII-1.VIII.2007, malaise trap, R. Copeland (1 female, USNM ENT 00764802 (USNM)). Central Prov., relict indigenous forest, Njukini Forest, 00°31.15'S, 37°25.19'E, 1455m, no date, malaise trap, R. Copeland (1 female, USNM ENT 00764803 (SAMC)).

#### Tessmannella expansa

Quinlan

urn:lsid:biosci.ohio-state.edu:osuc_concepts:181549

Morphbank accession: 704851–704858

http://www.waspweb.org/Cynipoidea/Liopteridae/Oberthuerellinae/Tessmannella/Tessmannella_expansa.htm

http://species-id.net/wiki/Tessmannella_expansa

Figures 41 –42

Tessmannella expansa Quinlan, 1979: 116.

##### Description.

Coloration of head and mesosoma, black to dark brown; metasoma and legs reddish brown. Sculpture on vertex, lateral surface of pronotum and mesoscutum present, deeply striate on head, costate with foveae on pronotum, mesoscutum.

*Head*. Broadly triangular, in anterior view. Pubescence on head present, dense setae covering head. Sculpture along lateral margin of occiput absent. Gena (measured from compound eye to posterolateral margin of head) short, ratio of length of gena to length of compound eye in dorsal view < 0.3, in dorsal view. Sculpture of gena smooth with remnants of costulae along posterior margin. Lateral margin of occiput defined by distinctly angled, raised, sharp carina. Occiput (except extreme lateral margin) smooth. Ocelli small, ratio of maximum diameter of a lateral ocellus to shortest distance between lateral ocelli 0.2–0.4. Anterior ocellus close to posterior ocelli, posterior margin of anterior ocellus behind or subcontiguous with a transverse line running through anterior margins of posterior ocelli. Relative position of toruli close to ocelli, ratio of vertical distance between inner margin of torulus and ventral margin of clypeus to vertical distance between anterior ocellus and torulus < 2.0. Median keel of face present, extending to middle of face, not reaching clypeus. Vertical carina adjacent to ventral margin of torulus absent. Facial sculpture almost entirely foveate, slightly horizontally striate along median keel. Facial impression absent, face flat. Antennal scrobe absent. Anterior tentorial pits small. Vertical delineations on lower face absent. Ventral clypeal margin laterally, close to anterior mandibular articulation, distinctly angled. Ventral clypeal margin medially emarginate. Clypeus horizontally striate. Malar space adjacent to anterior articulation of mandible evenly rounded, striate-foveate. Malar sulcus absent. Compound eye close to posterior ocellus, ratio of distance between compound eye and posterior mandibular articulation to distance between posterior ocellus and compound eye > 1.2. Compound eye, in dorsal view, distinctly protruding from the surface of the head, particularly laterally. Pubescence on compound eye absent. Orbital furrows absent. Lateral frontal carina of face absent. Dorsal aspect of vertex smooth to lightly punctate. Posterior aspect of vertex foveate. Hair punctures on lateral aspect of vertex absent. Posterior surface of head deeply impressed around postocciput.

*Labial-maxillary complex*. Apical segment of maxillary palp with pubescence, consisting only of erect setae. First segment of labial palp shorter than apical segment. Labial palp composed of three segments. Apical seta on apical segment of maxillary palp shorter than twice length of second longest apical seta. Erect setae medially on apical segment of maxillary palp present. Maxillary palp composed of four segments. Last two segments of maxillary palp (in normal repose) straight. Distal margin of subapical segment of maxillary palp slanting inwards, apical segment bending inwards. Apical segment of maxillary palp more than 1.5 times as long as preceding segment.

*Antenna*. Articulation between flagellomeres in antenna connate with articles broadly joined. Female antenna composed of 11 flagellomeres. Female F1 shorter than F2; black. Flagellomeres of female antenna cylindrical, not widened towards apex, non-clavate. Placoidal sensilla absent. Distal flagellomeres of female antenna not conspicuously enlarged compared to proximal flagellomeres.

*Pronotum*. Macrosculpture on lateral surface of pronotum present, foveate. Pubescence on lateral surface of pronotum present, sparse, consisting of few short hairs. Anterior flange of pronotal plate distinctly protruding anteriorly, longitudinally striate. Carinae extending posteriorly from lateral margin of pronotal plate absent. Lateral pronotal carina present. Pronotal crest present, raised into a distinct process projecting above anterior margin of mesoscutum. Submedian pronotal depressions absent, represented by shallow depression. Lateral margin of pronotal plate defined all the way to the dorsal margin of the pronotum. Pronotal plate wide, almost as wide as head.

*Mesoscutum*. Mesoscutal surface convex, evenly curved. Sculpture on mesoscutum present, transversely costate with dorsally projected serrations. Notaulus present, wide, smooth, distinctly wider posteriorly. Median mesoscutal carina absent. Anterior admedial lines present, with adjacent cuticular surface wrinkled. Median mesoscutal impression present, long, reaching over 1/2 length of mesoscutum. Parascutal carina nearly straight anteriorly, posteriorly curved mesally.

*Mesopleuron*. Dorsally with strigae running dorsoventrally; ventrally smooth, medially sparsely setose. Subpleuron anteriorly strigate, posteriorly smooth; medially with sparse, long setae. Lower mesopleuron medially smooth, glabrous; costate laterally, ventrally. Epicnemial carina absent. Lateroventral mesopleural carina present, marking abrupt change of slope of mesopectus. Mesopleural triangle present, distinctly impressed into longitudinal trough ventrally; dorsally striate, glabrous. Subalar pit large and well defined, lying in posterior end of mesopleural triangle. Speculum present, striate. Mesopleural carina absent.

*Sctuellum*. Dorsal surface of scutellum foveate-areolate. Circumscutellar carina absent. Posterior margin of axillula marked by distinct ledge, axillula distinctly impressed adjacent to ledge. Lateroventral margin of scutellum posterior to auricula smooth ventrally, obliquely longtidinally striate dorsally, entirely striate posteriorly. Dorsoposterior part of scutellum produced posteriorly into sharp spine, less than 1.0× length of petiole. Dorsal part of scutellum entirely rugose. Scutellar plate absent. Scutellar foveae present, three. Longitudinal scutellar carinae absent. Single longitudinal carina separating scutellar foveae absent. Posterolateral margin of scutellum drawn out into distinct protuberance. Lateral bar narrow, with strong strigate, foveate sculpture.

*Metapectal-propodeal complex*. Metapectal cavity anterodorsal to metacoxal base absent. Anterior margin of metapectal-propodeal complex seperated from mesopleuron by distinct dorso-ventral ledge. Posteroventral corner of metapleuron (in lateral view) extended posteriorly. Anterior impression of metepimeron present, triangular, with broadest part ventrally. Posterior margin of metepimeron distinct, separating metepimeron from propodeum. Subalar area slightly broadened anteriorly, without longitudinal division indicated. Calyptra present, blunt, lobe-like, polished posteriorly, strigate anteriorly with setiferous punctures. Dorsellum present, horizontally striate. Anterior impression of metepisternum, immediately beneath anterior end of metapleural carina, large and wide. Pubescence consisting of few scattered hairs on posterior part of metapleuron and lateral part of propodeum. Propodeal spurs present, striate. Lateral propodeal carinae present, not reaching scutellum. Ventral end of lateral propodeal carina reaching nucha, carinae separated from each other. Inter propodeal carinae space lightly setose, smooth. Petiolar foramen removed from metacoxae, directed posteriorly. Horizontal carina running anteriorly from lateral propodeal carina present. Lateral propodeal carina straight, sub-parallel. Calyptra, in lateral view, elongate. Propodeum ‘neck-like', drawn out posteriorly. Calyptra, in posterior view, dorsoventrally elongate.

*Legs*. Pubescence posterolaterally on metacoxa sparse to moderately dense, confined dense hair patch absent. Microsculpture on hind coxa absent. Longitudinal carina on the posterior surface of metatibia absent. Metafemoral spine present, elongate, with adjacent serrate ridge posteriorly. Distal mesotibial spurs shorter than medial spurs. Distal metatibial spurs shorter than medial spurs. Ratio of first metatibial segment to remaining 4 segments less than 1.0. Pubescence on outer surface of metatarsal claw sparse, consisting of few setae. Outer surface of metatarsal claw entirely smooth. Apical seta of metatarsal claw positioned on outer surface below dorsal margin. Base of metatarsal claw weakly expanded, apex slightly bent, ratio width of base to length of apex < 0.6.

*Forewing*. Pubescence of forewing present, sparse across entire wing surface. Apical margin of female forewing rounded. Rs+M of forewing tubular. Mesal end of Rs+M vein situated closer to posterior margin of forewing, directed towards posterior end of basalis. Vein R1 tubular along at least basal part of anterior margin of marginal cell. Basal abscissa of R1 (the abscissa between 2r and the forewing margin) of forewing as broad as adjacent wing veins. Coloration of forewing hyaline with slight infuscation covering marginal cell, area posterior to marginal cell. Marginal cell of forewing membranous, similar to other wing cells. Areolet absent. Hair fringe along apical margin of forewing absent.

*Petiole*. Distinctly elongate, > 5–6x longer than broad. Surface of petiole longitudinally costate, ventral keel absent, ventral costulae ribbed. Posterior part of female petiole not abruptly widened. Ventral flange of annulus of female petiole absent.

*Metasoma*. Setal band (hairy ring) at base of tergum 3 absent, base of metasoma glabrous. Tergum 3 distinctly smaller than tergum 4. Posterior margin of tergum 3 smoothly rounded. Posterior margin of tergum 4 straight. In lateral view, sternum 3 exposed, ventral border of T2–T7 visible. Sculpture on metasomal terga present, dorsally smooth, posteroventrally micropunctate. Syntergum absent, all postpetiolar terga free. Annulus absent. Peg-like setae on T6–T7 absent. Posteroventral cavities of female metasoma T7 present, setose. Female posteroventral margin of T6–T7 straight, parallel, with medial carina. Terebrum and hypopygium (in lateral view) straight, pointing posteriorly.

*Ovipositor*. First valvula of ovipositor narrowing gradually, not broadened apically, serrate at tip. Ovipositor clip absent.

**Figure 41.**
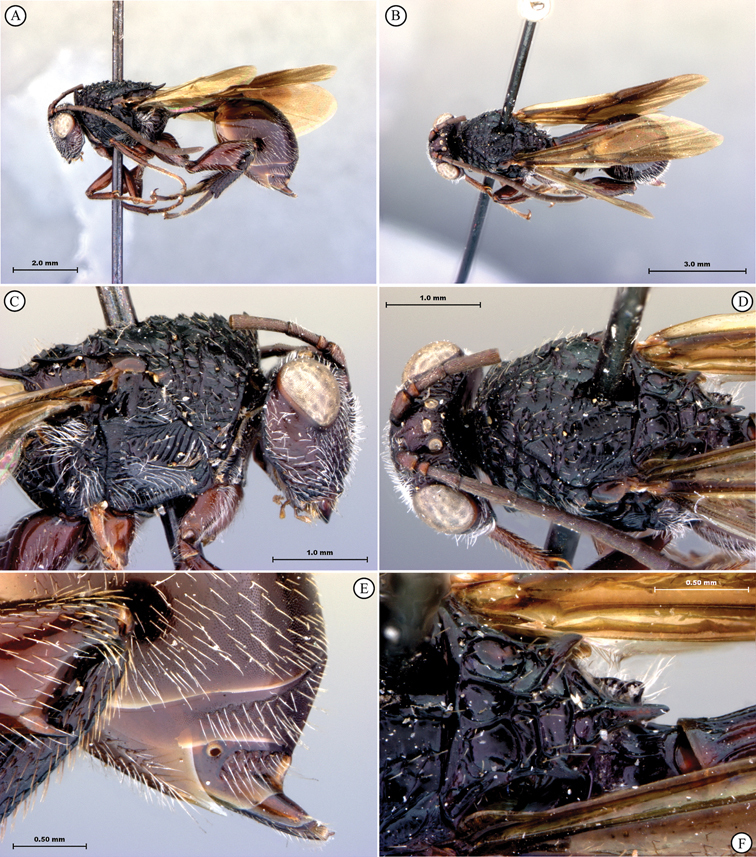
*Tessmannella expansa* Quinlan, holotype **A** lateral habitus **B** dorsal habitus **C** head and mesosoma, lateral view **D** head and mesosoma, dorsal view **E** hind margin for metasoma, lateral view **F** scutellum, dorsolateral view.

**Figure 42.**
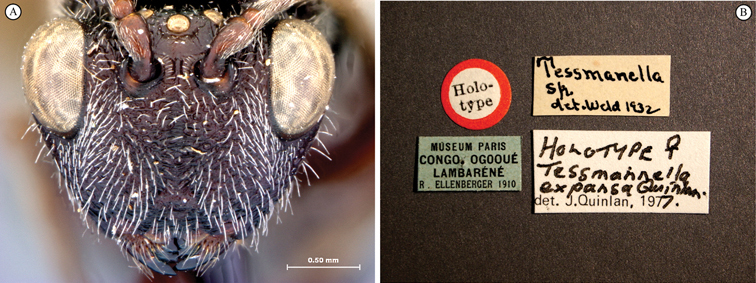
*Tessmannella expansa* Quinlan, holotype **A** head, anterior view **B** labels.

##### Diagnosis.

Closely resembles *Tessmannella nigra* and *Tessmannella spinosa* in having fully infuscate forewings; differented from these species having the medial facial area horizontally striate (shagreened in *Tessmannella spinosa*), and by having the ventral malar margin distinctly striate (gently shagreened in *Tessmannella nigra*).

##### Distribution.

Gabon. **Link to Distribution Map.** [http://hol.osu.edu/map-full.html?id=181549]

##### Material examined.

Holotype, female: **GABON:** Moyen-Ogooué Prov., Lambaréné, 1910, R. Ellenberger, USNM ENT 00764789 (deposited in MNHN).

#### Tessmannella kiplingi

Buffington & van Noort sp. n.

urn:lsid:zoobank.org:act:7C7527BA-CA8D-4765-885E-B4F4E316AC11

urn:lsid:biosci.ohio-state.edu:osuc_concepts:300222

Morphbank accession: 704859–704867

http://www.waspweb.org/Cynipoidea/Liopteridae/Oberthuerellinae/Tessmannella/Tessmannella_kiplingi.htm

http://species-id.net/wiki/Tessmannella_kiplingi

Figures 43 –44

##### Description.

Coloration of head, mesosoma, and metasoma black to dark brown; fore, mid legs lighter brown, hind legs dark brown to black. Sculpture on vertex, lateral surface of pronotum and mesoscutum present, moderately striate laterally on head, vertex; pronotum, mesoscutum horizontally striate with interspersed crests.

*Head*. Broadly triangular, in anterior view. Pubescence on head present, sparse setae scattered over head. Sculpture along lateral margin of occiput absent. Gena (measured from compound eye to posterolateral margin of head) short, ratio of length of gena to length of compound eye in dorsal view < 0.3, in dorsal view. Sculpture of gena deeply striate with remnants of fovea. Lateral margin of occiput defined by evenly rounded, raised, sharp carina. Occiput (except extreme lateral margin) smooth. Ocelli large, ratio of maximum diameter of a lateral ocellus to shortest distance between lateral ocelli > 0.4. Anterior ocellus between lateral ocelli. Relative position of toruli close to ocelli, ratio of vertical distance between inner margin of torulus and ventral margin of clypeus to vertical distance between anterior ocellus and torulus < 2.0. Median keel of face present, short, not extending beyond toruli. Vertical carina adjacent to ventral margin of torulus absent. Facial sculpture present, surface evenly foveate. Facial impression absent, face flat. Antennal scrobe absent. Anterior tentorial pits large. Vertical delineations on lower face absent. Ventral clypeal margin laterally, close to anterior mandibular articulation, distinctly angled. Ventral clypeal margin medially emarginate. Clypeus foveate-punctate; horizontally striate. Malar space adjacent to anterior articulation of mandible evenly rounded, foveate, with striate raised berm along mandibular base. Malar sulcus absent. Compound eye close to posterior ocellus, ratio of distance between compound eye and posterior mandibular articulation to distance between posterior ocellus and compound eye > 1.2. Compound eye, in dorsal view, distinctly protruding from the surface of the head, particularly laterally. Pubescence on compound eye absent. Orbital furrows absent. Lateral frontal carina of face absent. Dorsal aspect of vertex variously strigate. Posterior aspect of vertex punctate; foveate. Hair punctures on lateral aspect of vertex present, distinctly enlarged. Posterior surface of head almost flat, not deeply impressed.

*Labial-maxillary complex*. Apical segment of maxillary palp with pubescence, consisting only of erect setae. First segment of labial palp as long as apical segment. Labial palp composed of three segments. Apical seta on apical segment of maxillary palp shorter than twice length of second longest apical seta. Erect setae medially on apical segment of maxillary palp present. Maxillary palp composed of four segments. Last two segments of maxillary palp (in normal repose) straight. Distal margin of subapical segment of maxillary palp straight, apical segment bending outwards. Apical segment of maxillary palp more than 1.5 times as long as preceding segment.

*Antenna*. Articulation between flagellomeres in antenna connate with articles broadly joined. Female antenna composed of 11 flagellomeres. Female F1 shorter than F2; light brown in color. Flagellomeres of female antenna cylindrical, distinctly widened towards apex, non-clavate. Placoidal sensilla absent. Distal flagellomeres of female antenna not conspicuously enlarged compared to proximal flagellomeres.

*Pronotum*. Macrosculpture on lateral surface of pronotum present, dorsomedially costate with remnants of foveae, ventro-laterally gently striate to smooth. Pubescence on lateral surface of pronotum present, sparse, composed of few short hairs. Anterior flange of pronotal plate distinctly protruding anteriorly, centrally smooth, longitudinally striate laterally. Carinae extending posteriorly from lateral margin of pronotal plate absent. Lateral pronotal carina present. Pronotal crest present, raised into a distinct process projecting above anterior margin of mesoscutum. Submedian pronotal depressions closed laterally, deep. Lateral margin of pronotal plate defined all the way to the dorsal margin of the pronotum. Pronotal plate wide, almost as wide as head.

*Mesoscutum*. Mesoscutal surface convex, evenly curved. Sculpture on mesoscutum present, transversely costate with dorsally projected serrations. Notaulus present, wide, transversely striate, distinctly wider posteriorly. Median mesoscutal carina absent. Anterior admedial lines absent. Median mesoscutal impression present, medium in length, reaching 1/4 length of mesoscutum. Parascutal carina nearly straight anteriorly, posteriorly curved mesally.

*Mesopleuron*. Horizontally strigate dorsally, with single deep longitudinal trough at midline, ventrally slightly smoother, with gentle, parallel horizontal striae. Subpleuron gently smooth to shagreened, anteriorly and posteriorly with long, white setae; medially with single longitudinal carina. Lower mesopleuron medially smooth, setose; costate laterally, ventrally. Epicnemial carina absent. Lateroventral mesopleural carina present, not marking abrupt change of slope of mesopectus. Mesopleural triangle present, distinctly impressed into longitudinal trough ventrally; anteriorly setose, dorsally striate, ventrally smooth. Subalar pit large and well defined, lying in posterior end of mesopleural triangle. Speculum present, striate, with distinct smooth, glabrous ventral cavity. Mesopleural carina absent.

*Scutellum*. Dorsal surface of scutellum foveate-areolate. Circumscutellar carina absent. Posterior margin of axillula marked by distinct ledge, axillula distinctly impressed adjacent to ledge. Lateroventral margin of scutellum posterior to auricula smooth ventrally, obliquely longtidinally striate dorsally, entirely striate posteriorly. Dorsoposterior part of scutellum produced posteriorly into sharp spine, less than 1.0× length of petiole. Dorsal part of scutellum entirely rugose. Scutellar plate absent. Scutellar foveae present, three. Longitudinal scutellar carinae absent. Single longitudinal carina separating scutellar foveae absent. Posterolateral margin of scutellum drawn out into distinct protuberance. Lateral bar weakly strigate, narrow.

*Metapectal-propodeal complex*. Metapectal cavity anterodorsal to metacoxal base absent. Anterior margin of metapectal-propodeal complex seperated from mesopleuron by distinct dorso-ventral ledge. Posteroventral corner of metapleuron (in lateral view) extended posteriorly. Anterior impression of metepimeron present, triangular, with broadest part ventrally. Posterior margin of metepimeron distinct, separating metepimeron from propodeum. Subalar area slightly broadened anteriorly, without longitudinal division indicated. Calyptra present, blunt, lobe-like, polished posteriorly, strigate anteriorly with setiferous punctures. Dorsellum present, shagreened, glabrous. Anterior impression of metepisternum, immediately beneath anterior end of metapleural carina, large and wide. Pubescence long, dense, silvery on metapleuron; long, thin on propodeum. Propodeal spurs absent. Lateral propodeal carinae present, not reaching scutellum. Ventral end of lateral propodeal carina reaching nucha, carinae separated from each other. Inter propodeal carinae space glabrous, horizontally striate with central, bifurcating keel. Petiolar foramen removed from metacoxae, directed posteriorly. Horizontal carina running anteriorly from lateral propodeal carina present. Lateral propodeal carina straight, sub-parallel. Calyptra, in lateral view, elongate. Propodeum drawn out posteriorly, with nucha in line with terminus of scutellar spine. Calyptra, in posterior view, dorsoventrally elongate.

*Legs*. Pubescence posterolaterally on metacoxa present, evenly covering anterior aspect. Microsculpture on hind coxa present antero-laterally, smooth posterolaterally. Longitudinal carina on the posterior surface of metatibia present, well developed. Metafemoral spine present, elongate, with adjacent setal knob posteriorly. Distal mesotibial spurs shorter than medial spurs. Distal metatibial spurs shorter than medial spurs. Ratio of first metatibial segment to remaining 4 segments greater than 1.0. Pubescence on outer surface of metatarsal claw sparse, consisting of few setae. Outer surface of metatarsal claw entirely smooth. Apical seta of metatarsal claw positioned on outer surface below dorsal margin. Base of metatarsal claw weakly expanded, apex slightly bent, ratio width of base to length of apex < 0.6.

*Forewing*. Pubescence of forewing absent on basal half of wing, sparse distally. Apical margin of female forewing rounded. Rs+M of forewing tubular. Mesal end of Rs+M vein situated closer to posterior margin of forewing, directed towards posterior end of basalis. Vein R1 tubular along at least basal part of anterior margin of marginal cell. Basal abscissa of R1 (the abscissa between 2r and the forewing margin) of forewing as broad as adjacent wing veins. Coloration of forewing hyaline with distinct infuscation covering marginal cell, area posterior to marginal cell, and small patch at distally. Marginal cell of forewing membranous, similar to other wing cells. Areolet absent. Hair fringe along apical margin of forewing absent.

*Petiole*. Longitudinally costate, ribbed, ventral keel absent. Posterior part of female petiole not abruptly widened. Ventral flange of annulus of female petiole absent.

*Metasoma*. Setal band (hairy ring) at base of tergum 3 absent, base of metasoma glabrous. Tergum 3 distinctly smaller than tergum 4. Posterior margin of tergum 3 smoothly rounded. Posterior margin of tergum 4 straight. Sternum 3 exposed, ventral border of T2–T7 visible. Sculpture on metasomal terga present, finely punctate laterally, dorsally; posteriorly with large setal pits. Syntergum absent, all postpetiolar terga free. Annulus absent. Peg-like setae on T6–T7 absent. Posteroventral cavities of female metasoma T7 present, setose. Female posteroventral margin of T6–T7 straight, parallel. Terebrum and hypopygium (in lateral view) straight, pointing posteriorly.

*Ovipositor*. First valvula of ovipositor narrowing gradually, not broadened apically, smooth at tip. Ovipositor clip absent.

**Figure 43.**
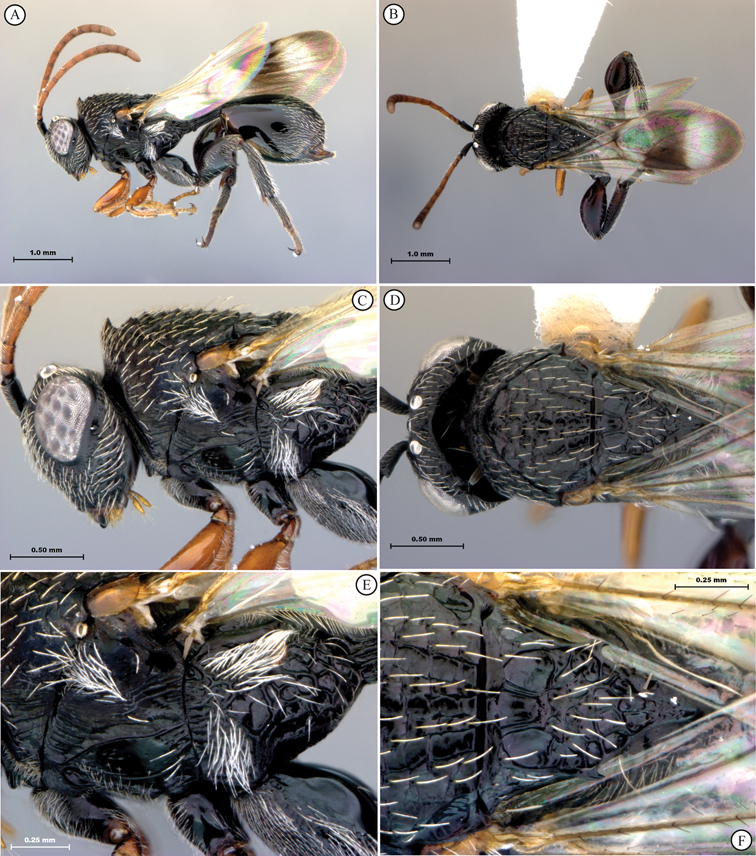
*Tessmannella kiplingi* Buffington & van Noort, sp. n., holotype **A** lateral habitus **B** dorsal habitus **C** head and mesosoma, lateral view **D** head and mesosoma, dorsal view **E** meso- and metapleurae, lateral view **F** scutellum, dorsal view.

**Figure 44.**
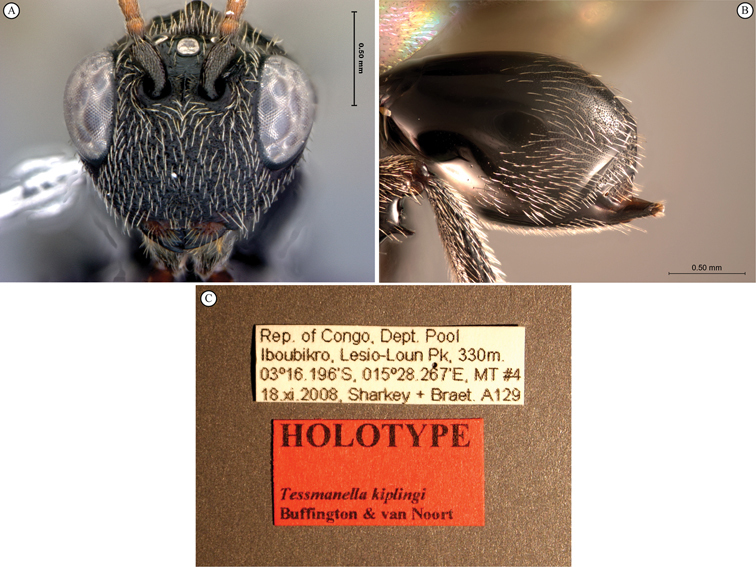
*Tessmannella kiplingi* Buffington & van Noort, sp. n., holotype **A** head, anterior view **B** metasoma, lateral view **C** labels.

##### Diagnosis.

Closely resembles *Tessmannella roberti* and *Tessmannella copelandi* in having the medial area of the forewing hyaline; distinguished from these species by having two distinct infuscate patches on the forewing. This species is further distinguished by the very distinctive wave-like texturing of the cuticle on the lateral aspects of the pronotum (areolate in other species of *Tessmannella*).

##### Etymology.

Named in honor of Rudyard Kipling, author of *Just So Stories* and others about Africa.

##### Distribution.

Congo. **Link to Distribution Map.**[http://hol.osu.edu/map-full.html?id=300222]

##### Material examined.

Holotype, female: **CONGO:** Pool Dépt., Iboubikro, MT 4, Lesio Louna Reserve, 03°16.196'S, 15°28.267'E, 340m, 18.X.2008, malaise trap, Braet and Sharkey, USNM ENT 00764795 (deposited in USNM). *Paratypes*: (4 females): **CONGO:** Pool Dépt., Iboubikro, MT 4, Lesio Louna Reserve, 03°16.196'S, 15°28.267'E, 340m, 1.X–8.X.2008, malaise trap, Braet and Sharkey (1 female, USNM ENT 00764796 (USNM)). Pool Dépt., Iboubikro, MT 4, Lesio Louna Reserve, 03°16.196'S, 15°28.267'E, 340m, 15.X–22.X.2008, malaise trap, Braet and Sharkey (1 female, USNM ENT 00764797 (USNM)). Pool Dépt., Iboubikro, MT 4, Lesio Louna Reserve, 03°16.196'S, 15°28.267'E, 340m, 15.X–22.X.2008, malaise trap, Braet and Sharkey (1 female, USNM ENT 00764798 (USNM)). Pool Dépt., Iboubikro, MT 4, Lesio Louna Reserve, 03°16.196'S, 15°28.267'E, 340m, 15.X–22.X.2008, malaise trap, Braet and Sharkey (1 female, USNM ENT 00764799 (USNM)).

#### Tessmannella nigra

Hedicke

urn:lsid:biosci.ohio-state.edu:osuc_concepts:181550

Morphbank accession: 704868–704875

http://www.waspweb.org/Cynipoidea/Liopteridae/Oberthuerellinae/Tessmannella/Tessmannella_nigra.htm

http://species-id.net/wiki/Tessmannella_nigra

Figures 45 –48

Tessmannella nigra Hedicke, 1912: 304.

##### Description.

Coloration of head, mesosoma, and metasoma black to dark brown; legs reddish brown. Sculpture on vertex, lateral surface of pronotum and mesoscutum present, gently striate on vertex, pronotum, mesoscutum striate-foveate.

*Head*. Broadly triangular, in anterior view. Pubescence on head present, sparse setae scattered over head. Sculpture along lateral margin of occiput absent. Gena (measured from compound eye to posterolateral margin of head) short, ratio of length of gena to length of compound eye in dorsal view < 0.3, in dorsal view. Sculpture of gena smooth with remnants of costulae along posterior margin. Lateral margin of occiput defined by distinctly angled, raised, sharp carina. Occiput (except extreme lateral margin) smooth. Ocelli small, ratio of maximum diameter of a lateral ocellus to shortest distance between lateral ocelli 0.2–0.4. Anterior ocellus close to posterior ocelli, posterior margin of anterior ocellus behind or subcontiguous with a transverse line running through anterior margins of posterior ocelli. Relative position of toruli close to ocelli, ratio of vertical distance between inner margin of torulus and ventral margin of clypeus to vertical distance between anterior ocellus and torulus < 2.0. Median keel of face present, short, not extending beyond toruli. Vertical carina adjacent to ventral margin of torulus absent. Facial sculpture transversely striate with remnants of foveae. Facial impression absent, face flat. Antennal scrobe absent. Anterior tentorial pits small. Vertical delineations on lower face absent. Ventral clypeal margin laterally, close to anterior mandibular articulation, straight. Ventral clypeal margin medially emarginate. Clypeus horizontally striate. Malar space adjacent to anterior articulation of mandible evenly rounded, striate. Malar sulcus absent. Compound eye close to posterior ocellus, ratio of distance between compound eye and posterior mandibular articulation to distance between posterior ocellus and compound eye > 1.2. Compound eye, in dorsal view, distinctly protruding from the surface of the head, particularly laterally. Pubescence on compound eye absent. Orbital furrows absent. Lateral frontal carina of face absent. Dorsal aspect of vertex smooth to lightly punctate. Posterior aspect of vertex punctate. Hair punctures on lateral aspect of vertex present, distinctly enlarged. Posterior surface of head deeply impressed around postocciput.

*Labial-maxillary complex*. Apical segment of maxillary palp with pubescence, consisting only of erect setae. First segment of labial palp shorter than apical segment. Labial palp composed of three segments. Apical seta on apical segment of maxillary palp shorter than twice length of second longest apical seta. Erect setae medially on apical segment of maxillary palp present. Maxillary palp composed of four segments. Last two segments of maxillary palp (in normal repose) curved inwards. Distal margin of subapical segment of maxillary palp slanting inwards, apical segment bending inwards. Apical segment of maxillary palp more than 1.5 times as long as preceding segment.

*Antenna*. Articulation between flagellomeres in antenna connate with articles broadly joined. Female antenna composed of 11 flagellomeres. Female F1 as long as F2. Flagellomeres of female antenna cylindrical, not widened towards apex, non-clavate. Placoidal sensilla absent. Distal flagellomeres of female antenna not conspicuously enlarged compared to proximal flagellomeres.

*Pronotum*. Macrosculpture on lateral surface of pronotum present, dorsomedially foveate, laterally foveate-costate. Pubescence on lateral surface of pronotum present, sparse, consisting of few short hairs. Anterior flange of pronotal plate distinctly protruding anteriorly, longitudinally striate. Carinae extending posteriorly from lateral margin of pronotal plate absent. Lateral pronotal carina present. Pronotal crest present, raised into a distinct process projecting above anterior margin of mesoscutum. Submedian pronotal depressions difficult to see, appear to be closed laterally, deep. Lateral margin of pronotal plate defined all the way to the dorsal margin of the pronotum. Pronotal plate wide, almost as wide as head.

*Mesoscutum*. Mesoscutal surface convex, evenly curved. Sculpture on mesoscutum present, transversely costate with dorsally projected serrations. Notaulus present, composed of deep furrows, transversely costate, distinctly wider anteriorly. Median mesoscutal carina absent. Anterior admedial lines absent. Median mesoscutal impression present, long, reaching over 1/2 length of mesoscutum. Parascutal carina distinctly sinuate, posteriorly ending in posteroventrally directed projection.

*Mesopleuron*. Dorsally with strigae running dorsoventrally; ventrally smooth, medially sparsely setose. Subpleuron anteriorly strigate, posteriorly smooth; medially with sparse, long setae. Lower mesopleuron medially smooth, glabrous; costate laterally, ventrally. Epicnemial carina absent. Lateroventral mesopleural carina present, marking abrupt change of slope of mesopectus. Mesopleural triangle present, distinctly impressed into longitudinal trough ventrally; dorsally striate, glabrous. Subalar pit large and well defined, lying in posterior end of mesopleural triangle. Speculum present, striate. Mesopleural carina absent.

*Scutellum*. Dorsal surface of scutellum foveate-areolate. Circumscutellar carina absent. Posterior margin of axillula marked by distinct ledge, axillula distinctly impressed adjacent to ledge. Lateroventral margin of scutellum posterior to auricula smooth, becoming dorsoventrally striate posteriorly. Dorsoposterior part of scutellum produced posteriorly into sharp spine, less than 1.0× length of petiole. Dorsal part of scutellum entirely rugose. Scutellar plate absent. Scutellar foveae present, three. Longitudinal scutellar carinae present as single central carina. Single longitudinal carina separating scutellar foveae absent. Posterolateral margin of scutellum drawn out into distinct protuberance. Lateral bar with strong strigate sculpture, narrow.

*Metapectal-propodeal complex*. Metapectal cavity anterodorsal to metacoxal base absent. Anterior margin of metapectal-propodeal complex seperated from mesopleuron by distinct dorso-ventral ledge. Posteroventral corner of metapleuron (in lateral view) extended posteriorly. Anterior impression of metepimeron present, triangular, with broadest part ventrally. Posterior margin of metepimeron distinct, separating metepimeron from propodeum. Subalar area slightly broadened anteriorly, without longitudinal division indicated. Calyptra present, blunt, v polished posteriorly with setiferous punctures anteriorly. Dorsellum present, horizontally striate. Anterior impression of metepisternum, immediately beneath anterior end of metapleural carina, large and wide. Pubescence consisting of few scattered hairs on posterior part of metapleuron and lateral part of propodeum. Propodeal spurs present, striate. Lateral propodeal carinae present, not reaching scutellum. Ventral end of lateral propodeal carina reaching nucha, carinae separated from each other. Inter propodeal carinae space lightly setose, foveate. Petiolar foramen removed from metacoxae, directed posteriorly. Horizontal carina running anteriorly from lateral propodeal carina present. Lateral propodeal carina straight, sub-parallel. Calyptra, in lateral view, rounded. Propodeum ‘neck-like', drawn out posteriorly. Calyptra, in posterior view, rounded.

*Legs*. Pubescence posterolaterally on metacoxa sparse to moderately dense, confined dense hair patch absent. Microsculpture on hind coxa absent. Longitudinal carina on the posterior surface of metatibia absent. Metafemoral spine present, elongate, with adjacent serrate ridge posteriorly. Distal mesotibial spurs longer than medial spurs. Distal metatibial spurs shorter than medial spurs. Ratio of first metatibial segment to remaining 4 segments less than 1.0. Pubescence on outer surface of metatarsal claw sparse, consisting of few setae. Outer surface of metatarsal claw microcarinate. Apical seta of metatarsal claw positioned on outer surface below dorsal margin. Base of metatarsal claw weakly expanded, apex slightly bent, ratio width of base to length of apex < 0.6.

*Forewing*. Pubescence of forewing absent on basal half of wing, sparse distally. Apical margin of female forewing rounded. Rs+M of forewing tubular. Mesal end of Rs+M vein situated closer to posterior margin of forewing, directed towards posterior end of basalis. Vein R1 tubular along at least basal part of anterior margin of marginal cell. Basal abscissa of R1 (the abscissa between 2r and the forewing margin) of forewing as broad as adjacent wing veins. Forewing entirely lightly infuscate. Marginal cell of forewing membranous, similar to other wing cells. Areolet absent. Hair fringe along apical margin of forewing absent.

*Petiole*. Distinctly elongate, > 5–6× longer than broad. Surface of petiole longitudinally costate, ventral keel absent, ventral costulae ribbed. Posterior part of female petiole not abruptly widened. Ventral flange of annulus of female petiole absent.

*Metasoma*. Setal band (hairy ring) at base of tergum 3 absent, base of metasoma glabrous. Tergum 3 distinctly smaller than tergum 4. Posterior margin of tergum 3 smoothly rounded. Posterior margin of tergum 4 straight. In lateral view, sternum 3 exposed, ventral border of T2–T7 visible. Sculpture on metasomal terga present, dorsally smooth, posteroventrally micropunctate. Syntergum absent, all postpetiolar terga free. Annulus absent. Peg-like setae on T6–T7 absent. Posteroventral cavities of female metasoma T7 present, setose. Female posteroventral margin of T6–T7 straight, parallel, with medial carina. Terebrum and hypopygium (in lateral view) straight, pointing posteriorly.

*Ovipositor*. First valvula of ovipositor narrowing gradually, not broadened apically, smooth at tip. Ovipositor clip absent.

**Figure 45.**
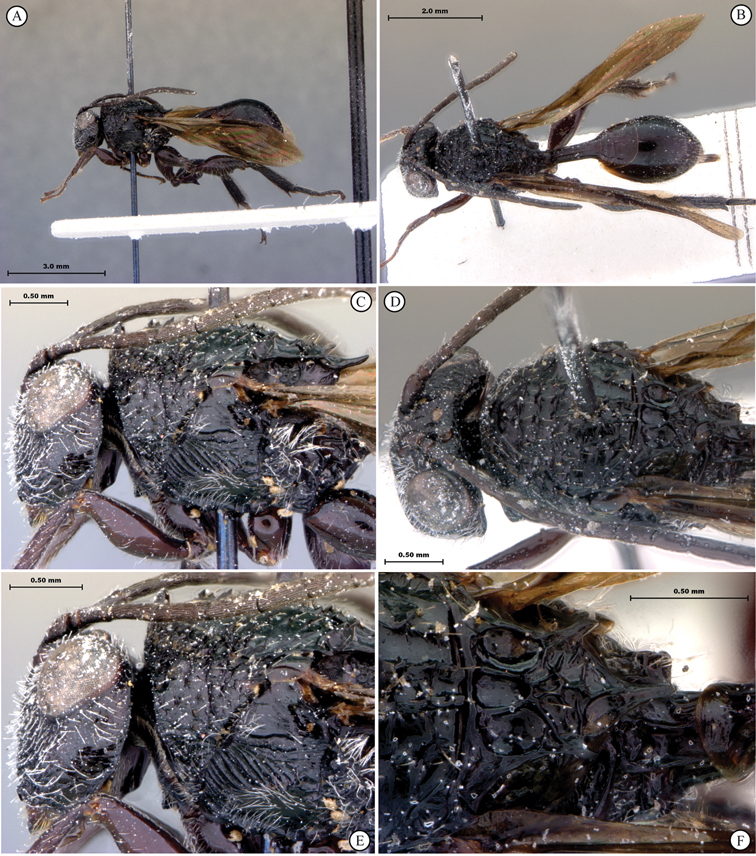
*Tessmannella nigra* Hedicke, holotype **A** lateral habitus **B** dorsal habitus **C** head and mesosoma, lateral view **D** head and mesosoma, dorsal view **E** head and pronotum, lateral view **F** scutellum, dorsal view.

**Figure 46.**
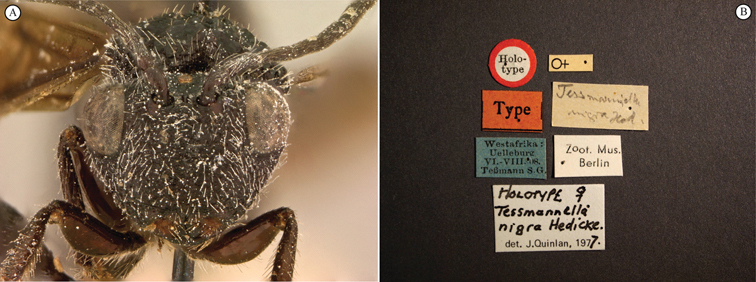
*Tessmannella nigra* Hedicke, holotype **A** head, anterior view **B** labels.

**Figure 47.**
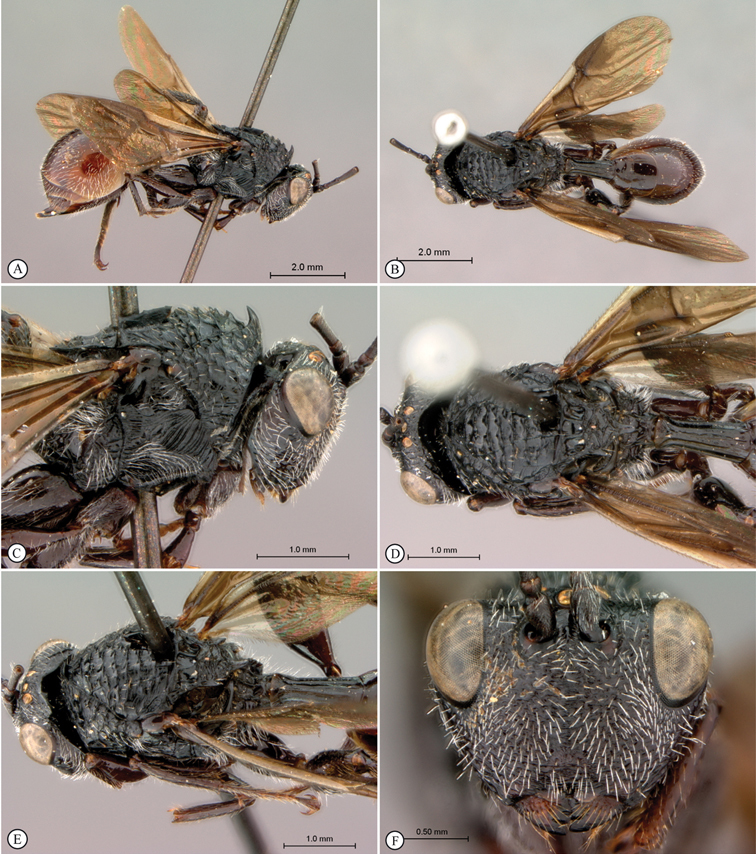
*Tessmannella nigra* Hedicke, non-type **A** lateral habitus **B** dorsal habitus **C** head and mesosoma, lateral view **D** head and mesosoma, dorsal view **E** head, mesosoma and petiole, dorsal view **F** head, anterior view.

**Figure 48.**
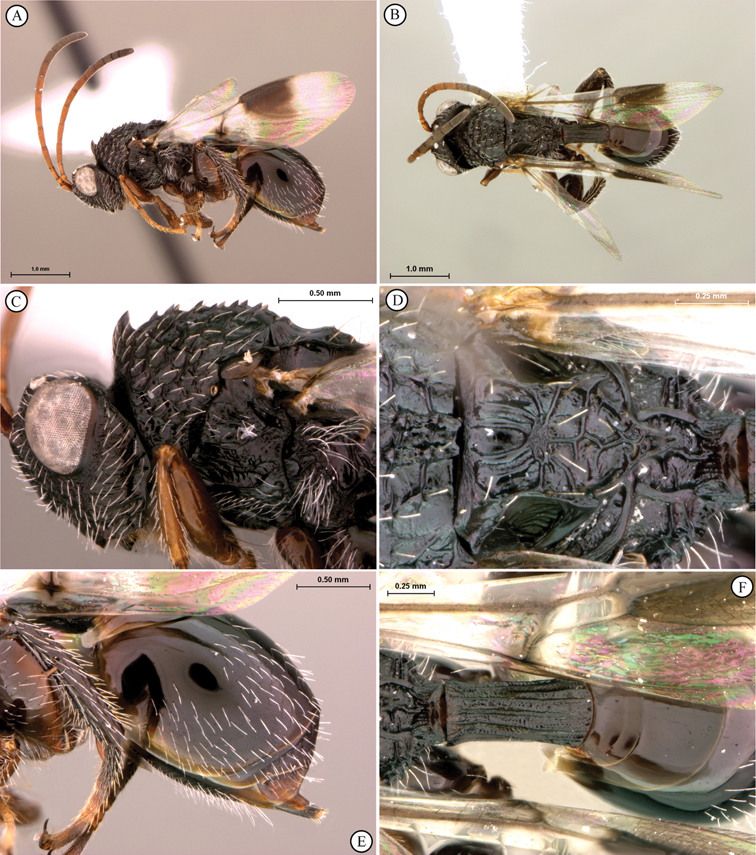
*Tessmannella roberti* Buffington & van Noort, sp. n., holotype **A** lateral habitus **B**  dorsal habitus **C** head and mesosoma, lateral view **D** scutellum, dorsal view **E** metasoma, lateral view **F** petiole, dorsal view.

##### Diagnosis.

Closely resembles *Tessmannella spinosa* and *Tessmannella expansa* in having uniform infuscation of the forewings (basally hyaline in *Tessmannella copelandi*, *Tessmannella kiplingi*, and *Tessmannella roberti*); distinguished from *Tessmannella spinosa* and *Tessmannella expansa* by having the ventral malar space, immediately adjacent to the mandibular base, gently shagreened (distinctly striate in the latter two species).

##### Distribution.

Equatorial Guinea, Democratic Republic of Congo. **Link to Distribution Map.** [http://hol.osu.edu/map-full.html?id=181550]

##### Material examined.

Holotype, female: **EQUATORIAL GUINEA:** Uelleburg, VI-1908 - VIII-1908, von Tessman, USNM ENT 00764790 (deposited in ZMHU). *Other material*: **DEMOCRATIC REPUBLIC OF THE CONGO:** Kibombo, 2.XI.1910, Bequaert (1 female, USNM ENT 00764793 (MRAC)).

#### Tessmannella roberti

Buffington & van Noort sp. n.

urn:lsid:zoobank.org:act:20C62448-FC96-4729-BF6F-43B90AB41C5C

urn:lsid:biosci.ohio-state.edu:osuc_concepts:300223

Morphbank accession: 704876–704883

http://www.waspweb.org/Cynipoidea/Liopteridae/Oberthuerellinae/Tessmannella/Tessmannella_roberti.htm

http://species-id.net/wiki/Tessmannella_roberti

Figures 48 –49

##### Description.

Coloration of head, mesosoma, and metasoma black to dark brown; fore and mid legs lighter brown, hind legs dark brown to black. Sculpture on vertex, lateral surface of pronotum and mesoscutum present, deeply striate on head, costate with foveae on pronotum, mesoscutum.

*Head*. Broadly triangular, in anterior view. Pubescence on head present, sparse setae scattered over head. Sculpture along lateral margin of occiput absent. Gena (measured from compound eye to posterolateral margin of head) short, ratio of length of gena to length of compound eye in dorsal view < 0.3, in dorsal view. Sculpture of gena deeply striate. Lateral margin of occiput defined by evenly rounded, raised, sharp carina. Occiput (except extreme lateral margin) smooth. Ocelli small, ratio of maximum diameter of a lateral ocellus to shortest distance between lateral ocelli 0.2–0.4. Anterior ocellus between lateral ocelli. Relative position of toruli close to ocelli, ratio of vertical distance between inner margin of torulus and ventral margin of clypeus to vertical distance between anterior ocellus and torulus < 2.0. Median keel of face present, extending to posterior margin of clypeus. Vertical carina adjacent to ventral margin of torulus absent. Facial sculpture present, evenly areolate. Facial impression absent, face flat. Antennal scrobe absent. Anterior tentorial pits large. Vertical delineations on lower face absent. Ventral clypeal margin laterally, close to anterior mandibular articulation, distinctly angled. Ventral clypeal margin medially emarginate. Clypeus horizontally striate. Malar space adjacent to anterior articulation of mandible evenly rounded, areolate. Malar sulcus absent. Compound eye close to posterior ocellus, ratio of distance between compound eye and posterior mandibular articulation to distance between posterior ocellus and compound eye > 1.2. Compound eye, in dorsal view, distinctly protruding from the surface of the head, particularly laterally. Pubescence on compound eye absent. Orbital furrows absent. Lateral frontal carina of face absent. Dorsal aspect of vertex variously strigate. Posterior aspect of vertex with parallel or slightly radiating, longitudinal strigae. Hair punctures on lateral aspect of vertex absent. Posterior surface of head almost flat, not deeply impressed.

*Labial-maxillary complex*. Apical segment of maxillary palp with pubescence, consisting of small number of erect setae and shorter, more appressed setae. Apical seta on apical segment of maxillary palp shorter than twice length of second longest apical seta. Erect setae medially on apical segment of maxillary palp present. Last two segments of maxillary palp (in normal repose) straight. Distal margin of subapical segment of maxillary palp straight, apical segment bending outwards. Apical segment of maxillary palp more than 1.5 times as long as preceding segment.

*Antenna*. Articulation between flagellomeres in antenna connate with articles broadly joined. Female antenna composed of 11 flagellomeres. Female F1 shorter than F2; light brown in color. Flagellomeres of female antenna cylindrical, distinctly widened towards apex, non-clavate. Placoidal sensilla absent. Distal flagellomeres of female antenna not conspicuously enlarged compared to proximal flagellomeres.

*Pronotum*. Macrosculpture on lateral surface of pronotum present, areolate. Pubescence on lateral surface of pronotum present, sparse, composed of few short hairs. Anterior flange of pronotal plate distinctly protruding anteriorly, longitudinally striate. Carinae extending posteriorly from lateral margin of pronotal plate absent. Lateral pronotal carina present. Pronotal crest present, raised into a distinct process projecting above anterior margin of mesoscutum. Submedian pronotal depressions closed laterally, shallow. Lateral margin of pronotal plate defined all the way to the dorsal margin of the pronotum. Pronotal plate wide, almost as wide as head.

*Mesoscutum*. Mesoscutal surface convex, evenly curved. Sculpture on mesoscutum present, transversely costate with dorsally projected serrations. Notaulus present, wide, transversely striate, distinctly wider posteriorly. Median mesoscutal carina absent. Anterior admedial lines present, with adjacent cuticular surface costate. Median mesoscutal impression present, long, reaching over 1/2 length of mesoscutum. Parascutal carina nearly straight anteriorly, posteriorly curved mesally.

*Mesopleuron*. Horizontally strigate dorsally, with single deep longitudinal trough at midline, ventrally slightly smoother, with gentle, parallel horizontal striae. Subpleuron entirely smooth with long, white setae on ventral half. Lower mesopleuron medially smooth, setose; costate laterally, ventrally. Epicnemial carina absent. Lateroventral mesopleural carina present, not marking abrupt change of slope of mesopectus. Mesopleural triangle present, distinctly impressed into longitudinal trough ventrally; anteriorly setose, dorsally striate, ventrally smooth. Subalar pit large and well defined, lying in posterior end of mesopleural triangle. Speculum present, striate, with distinct smooth, glabrous ventral cavity. Mesopleural carina absent.

*Scutellum*. Dorsal surface of scutellum areolate, inter-areol space shagreened. Circumscutellar carina absent. Posterior margin of axillula marked by distinct ledge, axillula distinctly impressed adjacent to ledge. Lateroventral margin of scutellum posterior to auricula dorsoventrally striate. Dorsoposterior part of scutellum produced posteriorly into sharp spine, less than 1.0× length of petiole. Dorsal part of scutellum entirely areolate. Scutellar plate absent. Scutellar foveae present, three, with lateral foveal bissected by longitudinal carina, resulting in five longitudinally elongate subfovea. Longitudinal scutellar carinae absent. Single longitudinal carina separating scutellar foveae absent. Posterolateral margin of scutellum drawn out into distinct protuberance. Lateral bar weakly strigate, narrow.

*Metapectal-propodeal complex*. Metapectal cavity anterodorsal to metacoxal base present, ill-defined. Anterior margin of metapectal-propodeal complex seperated from mesopleuron by distinct dorso-ventral ledge. Posteroventral corner of metapleuron (in lateral view) extended posteriorly. Anterior impression of metepimeron present, triangular, with broadest part ventrally. Posterior margin of metepimeron with single large, smooth, flat fovea separating metepimeron from propodeum. Subalar area slightly broadened anteriorly, without longitudinal division indicated. Calyptra present, blunt, lobe-like, polished posteriorly with setiferous punctures anteriorly. Dorsellum present, smooth, glabrous. Anterior impression of metepisternum, immediately beneath anterior end of metapleural carina, large and wide. Pubescence long, dense, silvery on metapleuron; long, thin on propodeum. Propodeal spurs present, reduced, composed of single crenulate carina. Lateral propodeal carinae present, not reaching scutellum. Ventral end of lateral propodeal carina reaching nucha, carinae separated from each other. Inter propodeal carinae space glabrous, horizontally striate with central, irregular keel. Petiolar foramen removed from metacoxae, directed posteriorly. Horizontal carina running anteriorly from lateral propodeal carina present. Lateral propodeal carina uniformly curved inward. Calyptra, in lateral view, elongate. Propodeum drawn out posteriorly, with nucha in line with terminus of scutellar spine. Calyptra, in posterior view, dorsoventrally elongate.

*Legs*. Pubescence posterolaterally on metacoxa sparse to moderately dense, confined dense hair patch absent. Microsculpture on hind coxa absent. Longitudinal carina on the posterior surface of metatibia present, well developed. Metafemoral spine present, elongate, with adjacent serrate ridge posteriorly. Distal mesotibial spurs shorter than medial spurs. Distal metatibial spurs shorter than medial spurs. Ratio of first metatibial segment to remaining 4 segments less than 1.0. Pubescence on outer surface of metatarsal claw sparse, consisting of few setae. Outer surface of metatarsal claw almost entirely smooth. Apical seta of metatarsal claw positioned on outer surface below dorsal margin. Base of metatarsal claw weakly expanded, apex slightly bent, ratio width of base to length of apex < 0.6.

*Forewing*. Pubescence of forewing absent on basal half of wing, sparse distally. Apical margin of female forewing rounded. Rs+M of forewing tubular. Mesal end of Rs+M vein situated closer to posterior margin of forewing, directed towards posterior end of basalis. Vein R1 tubular along at least basal part of anterior margin of marginal cell. Basal abscissa of R1 (the abscissa between 2r and the forewing margin) of forewing as broad as adjacent wing veins. Coloration of forewing hyaline with distinct infuscation covering marginal cell, area posterior to marginal cell. Marginal cell of forewing membranous, similar to other wing cells. Areolet absent. Hair fringe along apical margin of forewing absent.

*Petiole*. Moderately elongate, 2×–3× longer than wide. Surface of petiole longitudinally costate, ribbed, ventral keel absent. Posterior part of female petiole not abruptly widened. Ventral flange of annulus of female petiole absent.

*Metasoma*. Setal band (hairy ring) at base of tergum 3 absent, base of metasoma glabrous. Tergum 3 distinctly smaller than tergum 4. Posterior margin of tergum 3 smoothly rounded. Posterior margin of tergum 4 evenly rounded. In lateral view, sternum 3 exposed, ventral border of T2–T7 visible. Sculpture on metasomal terga present, finely punctate laterally, dorsally; posteriorly with large setal pits. Syntergum absent, all postpetiolar terga free. Annulus absent. Peg-like setae on T6–T7 absent. Posteroventral cavities of female metasoma T7 present, glabrous save for few, long setae. Female posteroventral margin of T6–T7 straight, parallel, with medial carina. Terebrum and hypopygium (in lateral view) straight, pointing posteriorly.

**Figure 49.**
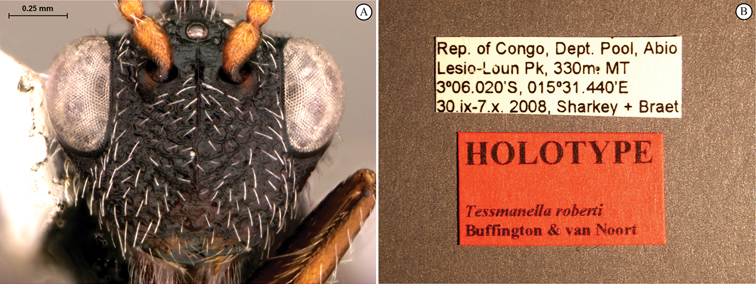
*Tessmannella roberti* Buffington & van Noort, sp. n., holotype **A** head, anterior view **B** labels.

##### Diagnosis.

Closely resembling *Tessmannella kiplingi* and *Tessmannella copelandi* in having the medial portion of the forewing hyaline; distinguished from these species by having a single infuscate patch in the marginal cell and immediately posterior (two patches in *Tessmannella kiplingi*), and having the central scutellar area areolate (smooth in *Tessmannella copelandi*).

##### Etymology

**.** Named in honor of the first author's father, Dr. Robert Buffington.

##### Distribution.

Central African Republic, Congo. **Link to Distribution Map.** [http://hol.osu.edu/map-full.html?id=300223]

##### Material examined.

Holotype, female: **CONGO:** Pool Dépt., Abio, Lesio Louna Reserve, 03°06.020'S, 15°31.440'E, 330m, 9.X–15.X.2008, malaise trap, Braet and Sharkey, USNM ENT 00764794 (deposited in USNM). Paratype: 1 female: - **CENTRAL AFRICAN REPUBLIC:** Sangha-Mbaéré Prefecture, Dzanga-Ndoki National Park, Mabéa Bai, 21.4km (53°) NE Bayanga, 03°02.01'N, 16°24.57'E, 510m, 7.V.2001, S. van Noort, sweep, CAR01-S90, lowland rainforest, marsh clearing, (1 female, SAM-HYM-P024410; USNM ENT 00764800 (SAMC)).

#### Tessmannella spinosa

Hedicke

urn:lsid:biosci.ohio-state.edu:osuc_concepts:181551

Morphbank accession: 704884–704893

http://www.waspweb.org/Cynipoidea/Liopteridae/Oberthuerellinae/Tessmannella/Tessmannella_spinosa.htm

http://species-id.net/wiki/Tessmannella_spinosa

Figures 50 –51

Tessmannella spinosa Hedicke, 1912: 303.

##### Description.

Coloration of head and mesosoma black to dark brown; metasoma and legs reddish brown. Sculpture on vertex, lateral surface of pronotum and mesoscutum present, deeply striate on head, costate with foveae on pronotum, mesoscutum.

*Head*. Broadly triangular, in anterior view. Pubescence on head present, dense setae covering head. Sculpture along lateral margin of occiput absent. Gena (measured from compound eye to posterolateral margin of head) short, ratio of length of gena to length of compound eye in dorsal view < 0.3. Sculpture of gena smooth with remnants of costulae along posterior margin. Lateral margin of occiput defined by distinctly angled, raised, sharp carina. Occiput (except extreme lateral margin) smooth. Ocelli small, ratio of maximum diameter of a lateral ocellus to shortest distance between lateral ocelli 0.2–0.4. Anterior ocellus close to posterior ocelli, posterior margin of anterior ocellus behind or subcontiguous with a transverse line running through anterior margins of posterior ocelli. Relative position of toruli close to ocelli, ratio of vertical distance between inner margin of torulus and ventral margin of clypeus to vertical distance between anterior ocellus and torulus < 2.0. Median keel of face present, short, not extending beyond toruli. Vertical carina adjacent to ventral margin of torulus absent. Facial sculpture present, punctate-rugose, transversely striate; striations meeting at midline of face. Facial impression absent, face flat. Antennal scrobe absent. Anterior tentorial pits small. Vertical delineations on lower face absent. Ventral clypeal margin laterally, close to anterior mandibular articulation, distinctly angled. Ventral clypeal margin medially emarginate. Clypeus circumscribed by clypeal carina; surface striate, converging ventro-medially. Malar space adjacent to anterior articulation of mandible evenly rounded, striate-foveate. Malar sulcus absent. Compound eye close to posterior ocellus, ratio of distance between compound eye and posterior mandibular articulation to distance between posterior ocellus and compound eye > 1.2. Compound eye, in dorsal view, distinctly protruding from the surface of the head, particularly laterally. Pubescence on compound eye absent. Orbital furrows absent. Lateral frontal carina of face absent. Dorsal aspect of vertex deeply foveate. Posterior aspect of vertex foveate. Hair punctures on lateral aspect of vertex present, indistinct. Posterior surface of head almost flat, not deeply impressed.

*Labial-maxillary complex*. Apical segment of maxillary palp with pubescence, consisting only of erect setae. First segment of labial palp shorter than apical segment. Labial palp composed of three segments. Apical seta on apical segment of maxillary palp shorter than twice length of second longest apical seta. Erect setae medially on apical segment of maxillary palp present. Maxillary palp composed of four segments. Last two segments of maxillary palp (in normal repose) straight. Distal margin of subapical segment of maxillary palp straight, apical segment bending outwards. Apical segment of maxillary palp more than 1.5 times as long as preceding segment.

*Antenna*. Articulation between flagellomeres in antenna connate with articles broadly joined. Female antenna composed of 11 flagellomeres. Female F1 as long as F2. Flagellomeres of female antenna cylindrical, not widened towards apex, non-clavate. Placoidal sensilla absent. Distal flagellomeres of female antenna not conspicuously enlarged compared to proximal flagellomeres.

*Pronotum*. Macrosculpture on lateral surface of pronotum present, dorsomedially foveate, laterally foveate-costate. Pubescence on lateral surface of pronotum present, sparse, consisting of few short hairs. Anterior flange of pronotal plate distinctly protruding anteriorly, transversely striate. Carinae extending posteriorly from lateral margin of pronotal plate absent. Lateral pronotal carina present. Pronotal crest present, raised into a distinct process projecting above anterior margin of mesoscutum. Submedian pronotal depressions absent, represented by shallow depression. Lateral margin of pronotal plate defined all the way to the dorsal margin of the pronotum. Pronotal plate wide, almost as wide as head.

*Mesoscutum*. Mesoscutal surface convex, evenly curved. Sculpture on mesoscutum present, transversely costate with dorsally projected serrations. Notaulus present, wide, transversely striate, distinctly wider posteriorly. Median mesoscutal carina absent. Anterior admedial lines present, with adjacent cuticular surface wrinkled. Median mesoscutal impression present, short, indicated by notch. Parascutal carina nearly straight anteriorly, posteriorly curved mesally.

*Mesopleuron*. Dorsally with strigae running dorsoventrally; ventrally smooth, medially sparsely setose. Subpleuron anteriorly strigate, posteriorly smooth; medially with sparse, long setae. Lower mesopleuron medially smooth, glabrous; costate laterally, ventrally. Epicnemial carina absent. Lateroventral mesopleural carina present, marking abrupt change of slope of mesopectus. Mesopleural triangle present, distinctly impressed into longitudinal trough ventrally; dorsally striate, glabrous. Subalar pit large and well defined, lying in posterior end of mesopleural triangle. Speculum present, striate. Mesopleural carina absent.

*Scutellum*. Dorsal surface of scutellum foveate-areolate. Circumscutellar carina absent. Posterior margin of axillula marked by distinct ledge, axillula distinctly impressed adjacent to ledge. Lateroventral margin of scutellum posterior to auricula smooth ventrally, obliquely longtidinally striate dorsally, entirely striate posteriorly. Dorsoposterior part of scutellum produced posteriorly into sharp spine, less than 1.0× length of petiole. Dorsal part of scutellum entirely rugose. Scutellar plate absent. Scutellar foveae present, three. Longitudinal scutellar carinae absent. Single longitudinal carina separating scutellar foveae absent. Posterolateral margin of scutellum drawn out into distinct protuberance. Lateral Lateral bar narrow, with strong strigate, foveate sculpture.

*Metapectal-propodeal complex*. Metapectal cavity anterodorsal to metacoxal base absent. Anterior margin of metapectal-propodeal complex seperated from mesopleuron by distinct dorso-ventral ledge. Posteroventral corner of metapleuron (in lateral view) extended posteriorly. Anterior impression of metepimeron present, triangular, with broadest part ventrally. Posterior margin of metepimeron distinct, separating metepimeron from propodeum. Subalar area slightly broadened anteriorly, without longitudinal division indicated. Calyptra present, blunt, lobe-like, polished posteriorly, strigate anteriorly with setiferous punctures. Dorsellum present, horizontally striate. Anterior impression of metepisternum, immediately beneath anterior end of metapleural carina, large and wide. Pubescence consisting of few scattered hairs on posterior part of metapleuron and lateral part of propodeum. Propodeal spurs present, striate. Lateral propodeal carinae present, not reaching scutellum. Ventral end of lateral propodeal carina reaching nucha, carinae separated from each other. Inter propodeal carinae space lightly setose, smooth. Petiolar foramen removed from metacoxae, directed posteriorly. Horizontal carina running anteriorly from lateral propodeal carina present. Lateral propodeal carina straight, sub-parallel. Calyptra, in lateral view, elongate. Propodeum ‘neck-like', drawn out posteriorly. Calyptra, in posterior view, dorsoventrally elongate.

*Legs*. Pubescence posterolaterally on metacoxa sparse to moderately dense, confined dense hair patch absent. Microsculpture on hind coxa absent. Longitudinal carina on the posterior surface of metatibia absent. Metafemoral spine present, elongate, with adjacent serrate ridge posteriorly. Distal mesotibial spurs shorter than medial spurs. Distal metatibial spurs shorter than medial spurs. Ratio of first metatibial segment to remaining 4 segments equal to 1.0. Pubescence on outer surface of metatarsal claw sparse, consisting of few setae. Outer surface of metatarsal claw almost entirely smooth. Apical seta of metatarsal claw positioned on outer surface below dorsal margin. Base of metatarsal claw weakly expanded, apex slightly bent, ratio width of base to length of apex < 0.6.

*Forewing*. Pubescence of forewing absent on basal half of wing, sparse distally. Apical margin of female forewing rounded. Rs+M of forewing tubular. Mesal end of Rs+M vein situated closer to posterior margin of forewing, directed towards posterior end of basalis. Vein R1 tubular along at least basal part of anterior margin of marginal cell. Basal abscissa of R1 (the abscissa between 2r and the forewing margin) of forewing as broad as adjacent wing veins. Coloration of forewing hyaline with slight infuscation covering marginal cell, area posterior to marginal cell. Marginal cell of forewing membranous, similar to other wing cells. Areolet absent. Hair fringe along apical margin of forewing absent.

*Petiole*. Distinctly elongate, > 5–6× longer than broad. Surface of petiole longitudinally costate, ventral keel absent, ventral costulae ribbed. Posterior part of female petiole not abruptly widened. Ventral flange of annulus of female petiole absent.

*Metasoma*. Setal band (hairy ring) at base of tergum 3 absent, base of metasoma glabrous. Tergum 3 distinctly smaller than tergum 4. Posterior margin of tergum 3 smoothly rounded. Posterior margin of tergum 4 straight. Sternum 3 exposed, ventral border of T2–T7 visible. Sculpture on metasomal terga present, dorsally smooth, posteroventrally micropunctate. Syntergum absent, all postpetiolar terga free. Annulus absent. Peg-like setae on T6–T7 absent. Posteroventral cavities of female metasoma T7 present, glabrous save for few, long setae. Female posteroventral margin of T6–T7 straight, parallel, with medial carina. Terebrum and hypopygium (in lateral view) straight, pointing posteriorly.

**FIgure 50.**
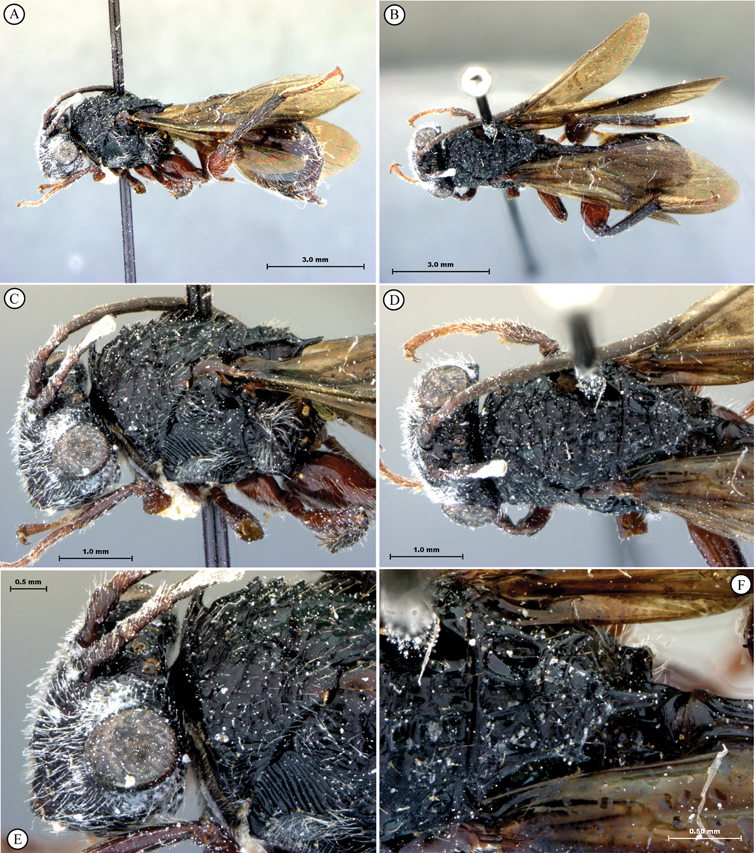
*Tessmannella spinosa* Hedicke, holotype **A** lateral habitus **B** dorsal habitus **C** head and mesosoma, lateral view **D** head and mesosoma, dorsal view **E** head and pronotum, dorsolateral view **F** scutellum, dorsal view.

**Figure 51.**
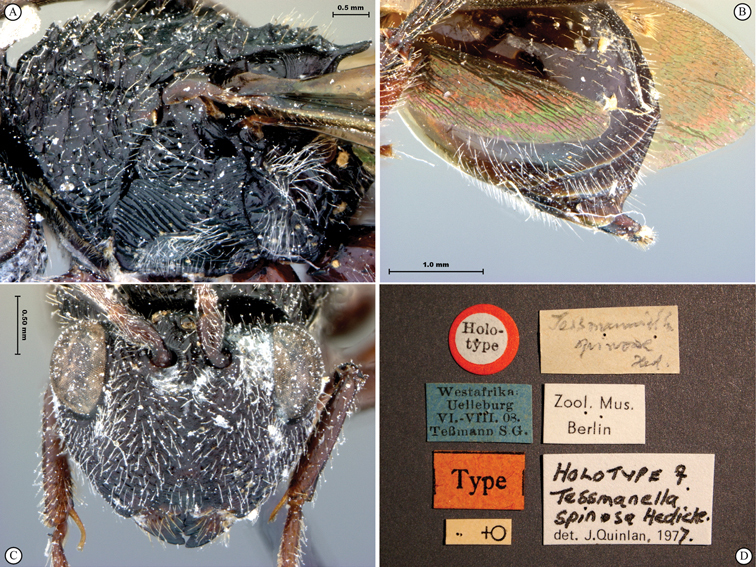
*Tessmannella spinosa* Hedicke, holotype **A** meso- and metapleurae, lateral view **B** metasoma, lateral view **C** head, anterior view **D** labels.

##### Diagnosis.

Closely resembles *Tessmannella expansa* and *Tessmannella nigra* by having the entire forewing uniformly infuscate (medially hyaline in *Tessmannella copelandi*, *Tessmannella kiplingi* and *Tessmannella roberti*); distinguished from *Tessmannella expansa* and *Tessmannella nigra* by the possession of a circum clypeal carina (lacking in these latter species).

##### Link to distribution map.

[http://hol.osu.edu/map-full.html?id=181551]

##### Material examined.

Holotype, female: **EQUATORIAL GUINEA:** Uelleburg, VI-1908 - VIII-1908, von Tessman, USNM ENT 00764791 (deposited in ZMHU). *Paralectotype*: **EQUATORIAL GUINEA:** Uelleburg, VI-1908 - VIII-1908, von Tessman (1 female, USNM ENT 00764792 (ZMHU)).

#### Xenocynips

Kieffer

http://www.waspweb.org/Cynipoidea/Liopteridae/Oberthuerellinae/Xenocynips/index.htm

http://species-id.net/wiki/Xenocynips

Xenocynips Kieffer, 1910b:340.Type species: *Xenocynips subsquamata* Kiefferby monotypy.

##### Diagnosis.

Metasomal terga 3–5 fused, with inter tergal sutures partially visible; lower mesopleuron horizontally striate. *Tessmannella* is most easily confused with *Xenocynips*; the fusion of terga in *Xenocynips* is a very reliable and clearly visible character. Additionally, most species of *Xenocynips* possess a dorsoventrally striate lateral aspect of the scutellum, posterior to the auricula; this is useful for specimens in which the metasoma is missing.

##### Distribution.

Cameroon, Central African Republic, Congo, Democratic Republic of Congo.

##### Biology.

Unknown.

##### Comments.

This little known genus was treated by both Quinlan (1979) and Ronquist (1995), the latter providing a proper redescription of the genus as well as apomorphies supporting its monophyly (included here as diagnostic characters). Quinlan (1979) reported the holotype of *Xenocynips subsquamata* as missing, but Ronquist (1995) reported the type in DEIC, and it is figured here (Fig. 56). This species, in addition to the two described here as new, bring the total number of *Xenocynips* species to three.

##### Included species.

*Xenocynips rhothion* Buffington & van Noort, sp. n.

*Xenocynips ronquisti* Buffington & van Noort, sp. n.

*Xenocynips subsquamata* Kieffer, 1910b: 340

##### Key to species of *Xenocynips* (both sexes)

(Available online at http://www.waspweb.org/Cynipoidea/Keys/index.htm)

**Table:** 

	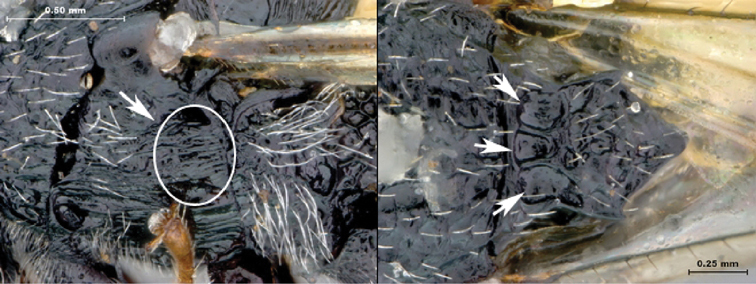
1A	Speculum entirely striate; three Scutellar foveae present (no subfovea); propodeal spurs absent	*Xenocynips ronquisti* sp. n.
	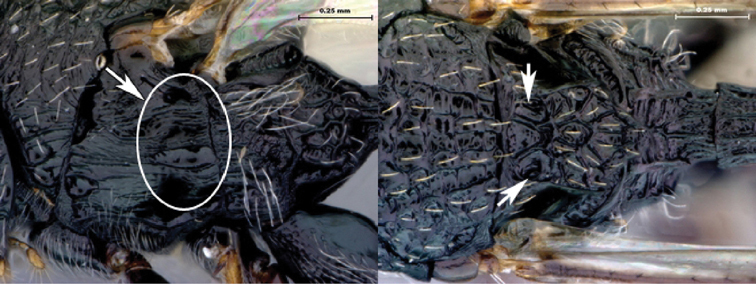
1B	Speculum dorsally striate, ventrally smooth; Scutellar foveae variously subdivided (subfovea present); propodeal spurs present	2
	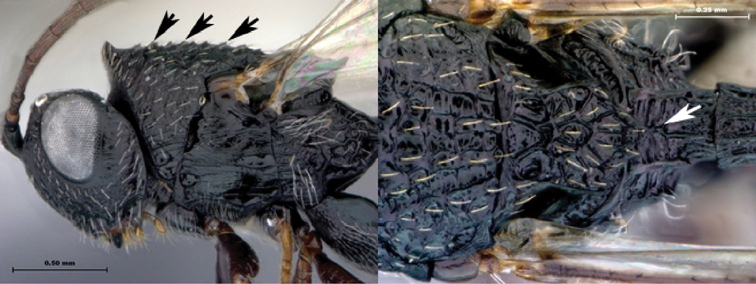
2A	Mesoscutal surface, when viewed laterally, with minute wave-like crests present, aligned with costae running horizontally; central propodeal keel forked medially	*Xenocynips rhothion* sp. n.
	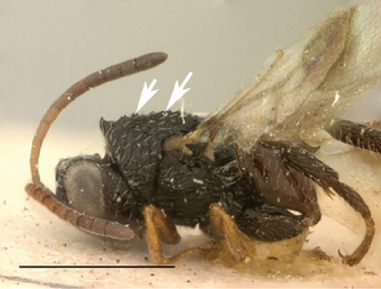
2B	Mesoscutal surface transversely costate, but wave-like crests not present (when viewed laterally); central propodeal keel, unforked medially	*Xenocynips subsquamata* Kieffe*r*

#### Xenocynips rhothion

Buffington & van Noort sp. n.

urn:lsid:zoobank.org:act:C2BECED8-575F-4D0C-BBE3-541B4D3E32E1

urn:lsid:biosci.ohio-state.edu:osuc_concepts:300224

Morphbank accession: 704919–704927

http://www.waspweb.org/Cynipoidea/Liopteridae/Oberthuerellinae/Xenocynips/Xenocynips_rhothion.htm

http://species-id.net/wiki/Xenocynips_rhothion

Figures 52 –53

##### Description.

Coloration of head, mesosoma, and metasoma black to dark brown; fore and mid legs lighter brown, hind legs dark brown to black. Sculpture on vertex, lateral surface of pronotum and mesoscutum present, moderately striate laterally on head, vertex; pronotum, mesoscutum horizontally striate with interspersed crests.

*Head*. Broadly triangular, in anterior view. Pubescence on head present, sparse setae scattered over head. Sculpture along lateral margin of occiput absent. Gena (measured from compound eye to posterolateral margin of head) short, ratio of length of gena to length of compound eye in dorsal view < 0.3, in dorsal view. Sculpture of gena deeply striate. Lateral margin of occiput defined by evenly rounded, raised, sharp carina. Occiput (except extreme lateral margin) smooth. Ocelli small, ratio of maximum diameter of a lateral ocellus to shortest distance between lateral ocelli 0.2–0.4. Anterior ocellus between lateral ocelli. Relative position of toruli close to ocelli, ratio of vertical distance between inner margin of torulus and ventral margin of clypeus to vertical distance between anterior ocellus and torulus < 2.0. Median keel of face present, short, not extending beyond toruli. Vertical carina adjacent to ventral margin of torulus absent. Facial sculpture present, punctate-rugose, transversely striate; striations meeting at midline of face; present, microcoriaceous. Facial impression absent, face flat. Antennal scrobe present, smooth with minute punctation. Anterior tentorial pits small. Vertical delineations on lower face absent. Ventral clypeal margin laterally, close to anterior mandibular articulation, distinctly angled. Ventral clypeal margin medially straight, not projecting. Clypeus foveate-punctate. Malar space adjacent to anterior articulation of mandible evenly rounded, striate-foveate. Malar sulcus absent. Compound eye close to posterior ocellus, ratio of distance between compound eye and posterior mandibular articulation to distance between posterior ocellus and compound eye > 1.2. Compound eye, in dorsal view, not distinctly protruding from the surface of the head. Pubescence on compound eye absent. Orbital furrows absent. Lateral frontal carina of face absent. Dorsal aspect of vertex variously strigate. Posterior aspect of vertex with parallel or slightly radiating, longitudinal strigae. Hair punctures on lateral aspect of vertex absent. Posterior surface of head deeply impressed around postocciput.

*Labial-maxillary complex*. Apical segment of maxillary palp with pubescence, consisting only of erect setae. First segment of labial palp as long as apical segment. Labial palp composed of two segments. Apical seta on apical segment of maxillary palp shorter than twice length of second longest apical seta. Erect setae medially on apical segment of maxillary palp present. Maxillary palp composed of four segments. Last two segments of maxillary palp (in normal repose) curved inwards. Distal margin of subapical segment of maxillary palp distinctly slanting outwards, apical segment bending outwards. Apical segment of maxillary palp more than 1.5 times as long as preceding segment.

*Antenna*. Articulation between flagellomeres in antenna connate with articles broadly joined. Female antenna composed of 11 flagellomeres. Female F1 shorter than F2; dark brown in color. Flagellomeres of female antenna cylindrical, distinctly widened towards apex, non-clavate. Placoidal sensilla absent. Distal flagellomeres of female antenna not conspicuously enlarged compared to proximal flagellomeres.

*Pronotum*. Macrosculpture on lateral surface of pronotum present, deeply costulate with remnants of foveae. Pubescence on lateral surface of pronotum present, sparse, consisting of few short hairs. Anterior flange of pronotal plate distinctly protruding anteriorly, longitudinally striate. Carinae extending posteriorly from lateral margin of pronotal plate absent. Lateral pronotal carina present. Pronotal crest present, raised into a distinct process projecting above anterior margin of mesoscutum. Submedian pronotal depressions closed laterally, deep. Lateral margin of pronotal plate defined all the way to the dorsal margin of the pronotum. Pronotal plate wide, almost as wide as head.

*Mesoscutum*. Mesoscutal surface convex, evenly curved. Sculpture on mesoscutum present, transversely costate-foveate with dorsally projected serrations. Notaulus present, wide, transversely striate, distinctly wider posteriorly. Median mesoscutal carina absent. Anterior admedial lines absent. Median mesoscutal impression present, long, reaching over 1/2 length of mesoscutum. Parascutal carina nearly straight anteriorly, posteriorly curved mesally.

*Mesopleuron*. Horizontally strigate dorsally, ventrally smooth with gentle, parallel horizontal striae. Subpleuron anteriorly with two deep cavities, centrally gently striate, posteriorly deeply strigate. Lower mesopleuron medially smooth, setose; costate laterally, ventrally. Epicnemial carina present on ventral half of mesopleuron; shagreened, ventrally bulbous near mesosternum. Lateroventral mesopleural carina present, marking abrupt change of slope of mesopectus. Mesopleural triangle present, distinctly impressed with distinct dorsal and ventral border, glabrous. Subalar pit large and well defined, lying in posterior end of mesopleural triangle. Speculum present, striate, with distinct smooth, glabrous ventral cavity. Mesopleural carina absent.

*Scutellum*. Dorsal surface of scutellum foveate-areolate. Circumscutellar carina present, complete, delimiting dorsal and ventral halves of scutellum. Posterior margin of axillula marked by distinct ledge, axillula distinctly impressed adjacent to ledge. Lateroventral margin of scutellum posterior to auricula smooth ventrally, longitudinally striate dorsally. Dorsoposterior part of scutellum produced posteriorly into blunt spine. Dorsal part of scutellum entirely rugose. Scutellar plate absent. Scutellar foveae present, three. Longitudinal scutellar carinae absent. Single longitudinal carina separating scutellar foveae absent. Posterolateral margin of scutellum drawn out into distinct protuberance. Lateral bar weakly strigate, narrow.

*Metapectal-propodeal complex*. Metapectal cavity anterodorsal to metacoxal base absent. Anterior margin of metapectal-propodeal complex seperated from mesopleuron by distinct dorso-ventral ledge. Posteroventral corner of metapleuron (in lateral view) rounded, not drawn out posteriorly. Anterior impression of metepimeron present, triangular, with broadest part ventrally. Posterior margin of metepimeron distinct, separating metepimeron from propodeum. Subalar area slightly broadened anteriorly, without longitudinal division indicated. Calyptra present, blunt, lobe-like, polished posteriorly, strigate anteriorly with setiferous punctures. Dorsellum present with two strong medial fovea, glabrous. Anterior impression of metepisternum, immediately beneath anterior end of metapleural carina, absent. Pubescence consisting of few scattered hairs on posterior part of metapleuron and lateral part of propodeum. Propodeal spurs present, foveate. Lateral propodeal carinae present, not reaching scutellum. Ventral end of lateral propodeal carina reaching nucha, carinae separated from each other. Inter propodeal carinae space glabrous, horizontally striate with central, bifurcating keel. Petiolar rim of uniform width along entire circumference. Petiolar foramen removed from metacoxae, directed posteriorly. Horizontal carina running anteriorly from lateral propodeal carina present. Lateral propodeal carina straight, sub-parallel. Calyptra, in lateral view, elongate. Propodeum ‘neck-like', drawn out posteriorly. Calyptra, in posterior view, dorsoventrally elongate.

*Legs*. Pubescence posterolaterally on metacoxa moderately dense, confined dense hair patch absent. Microsculpture on hind coxa present antero-laterally, smooth posterolaterally. Longitudinal carina on the posterior surface of metatibia present, well developed. Metafemoral spine present, elongate, with adjacent serrate ridge posteriorly. Distal mesotibial spurs shorter than medial spurs. Distal metatibial spurs shorter than medial spurs. Ratio of first metatibial segment to remaining 4 segments less than 1.0. Pubescence on outer surface of metatarsal claw sparse, consisting of few setae. Outer surface of metatarsal claw entirely smooth. Apical seta of metatarsal claw positioned on outer surface below dorsal margin. Base of metatarsal claw weakly expanded, apex slightly bent, ratio width of base to length of apex < 0.6.

*Forewing*. Pubescence of forewing absent on basal half of wing, sparse distally. Apical margin of female forewing rounded. Rs+M of forewing tubular. Mesal end of Rs+M vein situated closer to posterior margin of forewing, directed towards posterior end of basalis. Vein R1 tubular along at least basal part of anterior margin of marginal cell. Basal abscissa of R1 (the abscissa between 2r and the forewing margin) of forewing as broad as adjacent wing veins. Coloration of forewing hyaline with slight infuscation covering marginal cell, area posterior to marginal cell. Marginal cell of forewing membranous, similar to other wing cells. Areolet absent. Hair fringe along apical margin of forewing absent.

*Petiole*. Slightly elongate, 1.5–2× longer than wide. Surface of petiole longitudinally costate, ribbed, ventral keel absent. Posterior part of female petiole not abruptly widened. Ventral flange of annulus of female petiole absent.

*Metasoma*. Setal band (hairy ring) at base of tergum 3 absent, base of metasoma glabrous. Tergum 3 indistinct, fused with syntergum. Posterior margin of tergum 3 indistinct, fused with tergum 4 in syntergum. In lateral view, sternum 3 encompassed by syntergum. Sculpture on metasomal terga present, finely punctate laterally, dorsally; posteriorly with large setal pits. Syntergum present with terga 3–5 fused, ventral margin rounded. Annulus absent. Peg-like setae on T6–T7 absent. Posteroventral cavities of female metasoma T7 present, glabrous save for few, long setae. Female posteroventral margin of T6–T7 gently sinuate. Terebrum and hypopygium (in lateral view) straight, pointing posteriorly.

*Ovipositor*. First valvula of ovipositor narrowing gradually, not broadened apically, smooth at tip. Ovipositor clip absent.

**Figure 52.**
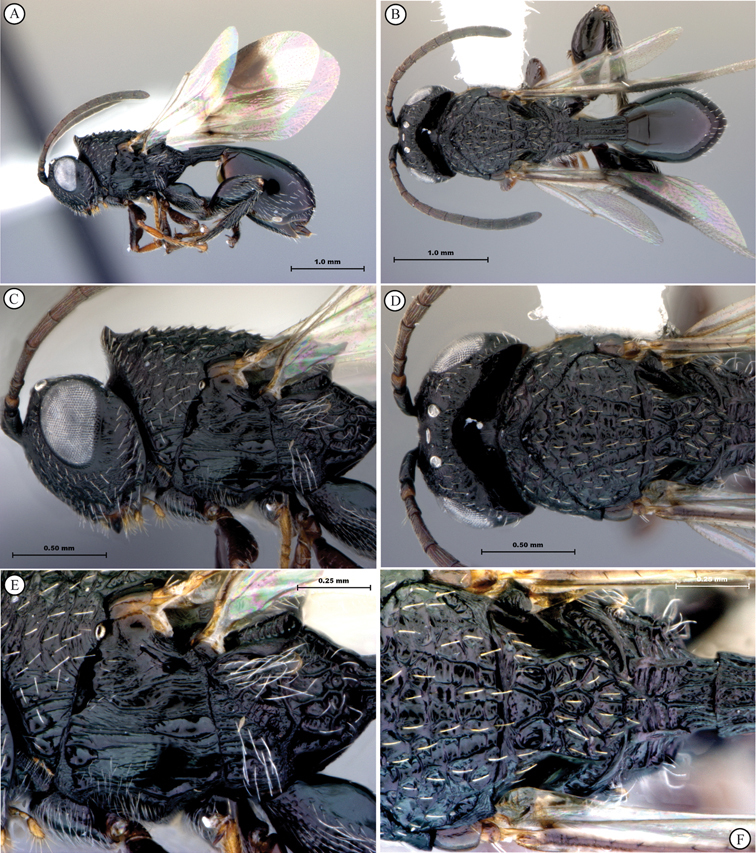
*Xenocynips rhothion* Buffington & van Noort, sp. n., holotype **A** lateral habitus **B** dorsal habitus **C** head and mesosoma, lateral view **D** head and mesosoma, dorsal view **E** meso- and metapleurae, lateral view **F** scutellum, dorsal view.

**Figure 53.**
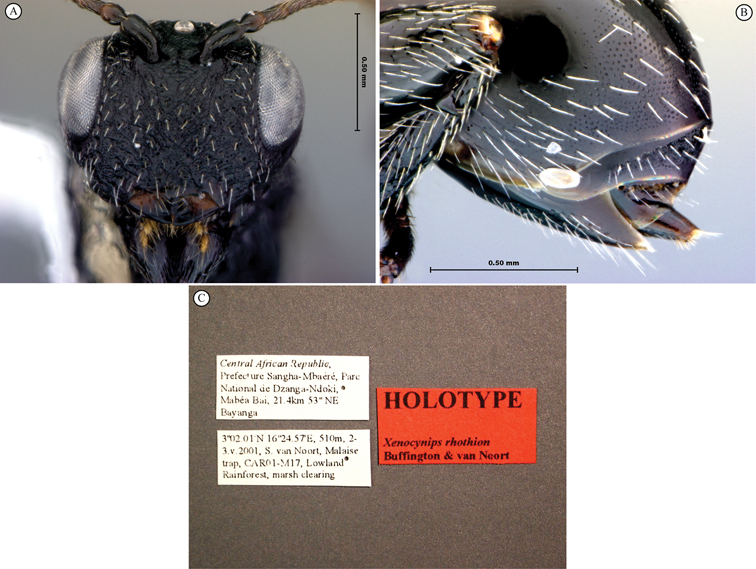
*Xenocynips rhothion* Buffington & van Noort, sp. n., holotype **A** head, anterior view **B** hind margin of metasoma, lateral view **C** labels.

##### Diagnosis.

This species differs from all other *Xenocynips* by the morphology of the pronotum being wave-like, reminiscent of waves on the ocean; in other species, the pronotum is horizontally costate. Also unique is the forked central propodeal keel; in other species, this keel is entire.

##### Etymology.

Greek for wave or surf, in reference to the wave-like striations of the pronotum.

##### Distribution.

Central African Republic, Congo. **Link to Distribution Map.** [http://hol.osu.edu/map-full.html?id=300224]

##### Material examined.

Holotype, female: **CENTRAL AFRICAN REPUBLIC:** Sangha-Mbaéré Préf. Écon., 21.4km (53°) NE Bayanga, Mabéa Bai, lowland rainforest / marsh clearing, CAR01-M17, Dzanga-Ndoki National Park, 03°02.01'N, 16°24.57'E, 510m, 2.V–3.V.2001, malaise trap, S. van Noort, USNM ENT 00764785 (deposited in SAMC). *Paratypes*: (3 females) **CENTRAL AFRICAN REPUBLIC:** Nana-Mambéré Préf., 60km W Bouar, 05°45'N, 15°13'E, 23.III.2010, yellow pan trap, J. Halada (1 female, USNM ENT 00764788 (CNCI)). **CONGO:** Pool Dépt., Iboubikro, MT 4, Lesio Louna Reserve, 03°16.196'S, 15°28.267'E, 340m, 13.X.2008, malaise trap, Braet and Sharkey (1 female, USNM ENT 00764786 (USNM)). Pool Dépt., Iboubikro, MT 4, Lesio Louna Reserve, 03°16.196'S, 15°28.267'E, 340m, 9.X–15.X.2008, malaise trap, Braet and Sharkey (1 female, USNM ENT 00764787 (USNM)).

#### Xenocynips ronquisti

Buffington & van Noort sp. n.

urn:lsid:zoobank.org:act:1BB28DD3-5290-471C-B4AF-08B34980FF9F

urn:lsid:biosci.ohio-state.edu:osuc_concepts:300225

Morphbank accession: 704908–704918

http://www.waspweb.org/Cynipoidea/Liopteridae/Oberthuerellinae/Xenocynips/Xenocynips_ronquisti.htm

http://species-id.net/wiki/Xenocynips_ronquisti

Figures 54 –55

##### Description.

Coloration of head, mesosoma, and metasoma, dark reddish brown; legs reddish brown. Sculpture on vertex, lateral surface of pronotum and mesoscutum present, deeply foveate laterally on head, pronotum, mesoscutum.

*Head*. Broadly triangular, in anterior view. Pubescence on head present, sparse setae scattered over head. Sculpture along lateral margin of occiput absent. Gena (measured from compound eye to posterolateral margin of head) short, ratio of length of gena to length of compound eye in dorsal view < 0.3. Sculpture of gena present, with distinct fovea. Lateral margin of occiput defined by distinctly angled, raised, sharp carina. Occiput (except extreme lateral margin) smooth. Ocelli small, ratio of maximum diameter of a lateral ocellus to shortest distance between lateral ocelli 0.2–0.4. Anterior ocellus between lateral ocelli. Relative position of toruli close to ocelli, ratio of vertical distance between inner margin of torulus and ventral margin of clypeus to vertical distance between anterior ocellus and torulus < 2.0. Median keel of face present, extending to middle of face, not reaching clypeus. Vertical carina adjacent to ventral margin of torulus present. Facial sculpture present, punctate-rugose, transversely striate; striations meeting at midline of face; present, microcoriaceous. Facial impression absent, face flat. Antennal scrobe absent. Anterior tentorial pits small. Vertical delineations on lower face present, single orbital furrow along inner margin of compound eye. Ventral clypeal margin laterally, close to anterior mandibular articulation, distinctly angled. Ventral clypeal margin medially straight, not projecting. Clypeus horizontally striate; circumscribed by clypeal carina; surface striate, converging ventro-medially. Malar space adjacent to anterior articulation of mandible evenly rounded, striate-foveate. Malar sulcus absent. Compound eye close to posterior ocellus, ratio of distance between compound eye and posterior mandibular articulation to distance between posterior ocellus and compound eye > 1.2. Compound eye, in dorsal view, distinctly protruding from the surface of the head, particularly laterally. Pubescence on compound eye absent. Orbital furrows rounded, ill-defined, running from lateral edge of torulus to ventral margin of compound eye. Lateral frontal carina of face present. Dorsal aspect of vertex with carinae extending from each torulus, defining outer margin of scrobe toward lateral ocelli, reaching posteriorly to median ocellus. Posterior aspect of vertex foveate. Hair punctures on lateral aspect of vertex absent. Posterior surface of head deeply impressed around postocciput.

*Labial-maxillary complex*. Apical segment of maxillary palp with pubescence, consisting only of erect setae. First segment of labial palp shorter than apical segment. Apical seta on apical segment of maxillary palp shorter than twice length of second longest apical seta. Erect setae medially on apical segment of maxillary palp present. Last two segments of maxillary palp (in normal repose) curved inwards. Apical segment of maxillary palp more than 1.5 times as long as preceding segment.

*Antenna*. Articulation between flagellomeres in antenna connate with articles broadly joined. Male antenna composed of 12 flagellomeres. Placoidal sensilla absent. Second flagellomere of male antenna slightly asymmetric basally. Length of second flagellomere of male antenna longer than first flagellomere.

*Pronotum*. Macrosculpture on lateral surface of pronotum present, foveate. Pubescence on lateral surface of pronotum present, sparse, composed of few short hairs. Anterior flange of pronotal plate distinctly protruding anteriorly, longitudinally striate. Carinae extending posteriorly from lateral margin of pronotal plate absent. Lateral pronotal carina present. Pronotal crest present, raised into a distinct process projecting above anterior margin of mesoscutum. Submedian pronotal depressions absent, represented by shallow depression. Lateral margin of pronotal plate defined all the way to the dorsal margin of the pronotum. Pronotal plate wide, almost as wide as head.

*Mesoscutum*. Mesoscutal surface convex, evenly curved. Sculpture on mesoscutum present, transversely costate-foveate with dorsally projected serrations. Notaulus present, composed of series of deep subcontiguous pits of uniform width. Median mesoscutal carina absent. Anterior admedial lines absent. Median mesoscutal impression present, long, reaching over 1/2 length of mesoscutum. Parascutal carina nearly straight anteriorly, posteriorly curved mesally.

*Mesopleuron*. Horizontally strigate dorsally, with single deep longitudinal trough at midline, ventrally slightly smoother, with gentle, parallel horizontal striae. Subpleuron anteriorly with two deep cavities, centrally gently striate, posteriorly deeply strigate. Lower mesopleuron entirely striate. Epicnemial carina present on ventral half of mesopleuron; shagreened, ventrally bulbous near mesosternum. Lateroventral mesopleural carina present, marking abrupt change of slope of mesopectus. Mesopleural triangle present, gently impressed lacking dorsal and ventral border; deeply striate. Subalar pit large and well defined, lying in posterior end of mesopleural triangle. Speculum present, striate. Mesopleural carina absent.

*Scutellum*. Dorsal surface of scutellum foveate-areolate. Circumscutellar carina present, complete, delimiting dorsal and ventral halves of scutellum. Posterior margin of axillula marked by distinct ledge, axillula distinctly impressed adjacent to ledge. Lateroventral margin of scutellum posterior to auricula smooth ventrally, longitudinally striate dorsally. Dorsoposterior part of scutellum produced posteriorly into blunt spine. Dorsal part of scutellum entirely rugose. Scutellar plate absent. Scutellar foveae present, three. Longitudinal scutellar carinae absent. Single longitudinal carina separating scutellar foveae absent. Posterolateral margin of scutellum drawn out into distinct protuberance. Lateral bar with strong strigate sculpture, conspicuously widened ventrally into lobe.

*Metapectal-propodeal complex*. Metapectal cavity anterodorsal to metacoxal base absent. Anterior margin of metapectal-propodeal complex seperated from mesopleuron by distinct dorso-ventral ledge. Posteroventral corner of metapleuron (in lateral view) extended posteriorly. Anterior impression of metepimeron present, triangular, with broadest part ventrally. Posterior margin of metepimeron distinct, separating metepimeron from propodeum. Subalar area slightly broadened anteriorly, without longitudinal division indicated. Calyptra present, blunt, lobe-like, polished posteriorly with setiferous punctures anteriorly. Dorsellum present, horizontally striate. Anterior impression of metepisternum, immediately beneath anterior end of metapleural carina, absent. Pubescence present on metapleuron, long, not dense; absent of propodeum. Propodeal spurs absent. Lateral propodeal carinae present, not reaching scutellum. Ventral end of lateral propodeal carina reaching nucha, carinae separated from each other. Inter propodeal carinae space glabrous, costulate. Petiolar foramen removed from metacoxae, directed posteriorly. Horizontal carina running anteriorly from lateral propodeal carina present. Lateral propodeal carina straight, sub-parallel. Calyptra, in lateral view, elongate. Propodeum ‘neck-like', drawn out posteriorly. Calyptra, in posterior view, dorsoventrally elongate.

*Legs*. Pubescence posterolaterally on metacoxa sparse to moderately dense, confined dense hair patch absent. Microsculpture on hind coxa present antero-laterally, smooth posterolaterally. Longitudinal carina on the posterior surface of metatibia absent. Metafemoral spine present, elongate, extending distally as low keel along ventral femoral margin. Distal mesotibial spurs longer than medial spurs. Distal metatibial spurs shorter than medial spurs. Ratio of first metatibial segment to remaining 4 segments greater than 1.0. Pubescence on outer surface of metatarsal claw sparse, consisting of few setae. Outer surface of metatarsal claw entirely smooth. Apical seta of metatarsal claw positioned on outer surface below dorsal margin. Base of metatarsal claw weakly expanded, apex slightly bent, ratio width of base to length of apex < 0.6.

*Forewing*. Pubescence of forewing absent on basal half of wing, sparse distally. Apical margin of female forewing rounded. Rs+M of forewing tubular. Mesal end of Rs+M vein situated closer to posterior margin of forewing, directed towards posterior end of basalis. Vein R1 tubular along at least basal part of anterior margin of marginal cell. Basal abscissa of R1 (the abscissa between 2r and the forewing margin) of forewing as broad as adjacent wing veins. Coloration of forewing hyaline with slight infuscation covering marginal cell, area posterior to marginal cell. Marginal cell of forewing membranous, similar to other wing cells. Areolet absent. Hair fringe along apical margin of forewing absent.

*Petiole*. Slightly elongate, 1.5–2× longer than wide. Surface of petiole longitudinally costate, ribbed, ventral keel absent. Posterior part of female petiole not abruptly widened. Ventral flange of annulus of female petiole absent.

*Metasoma*. Setal band (hairy ring) at base of tergum 3 absent, base of metasoma glabrous. Tergum 3 indistinct, fused with syntergum. Posterior margin of tergum 3 indistinct, fused with tergum 4 in syntergum. Sternum 3 encompassed by syntergum. Sculpture on metasomal terga present, finely punctate laterally, dorsally; posteriorly with large setal pits. Syntergum present with terga 3–5 fused, ventral margin rounded. Annulus absent. Peg-like setae on T6–T7 absent.

**Figure 54.**
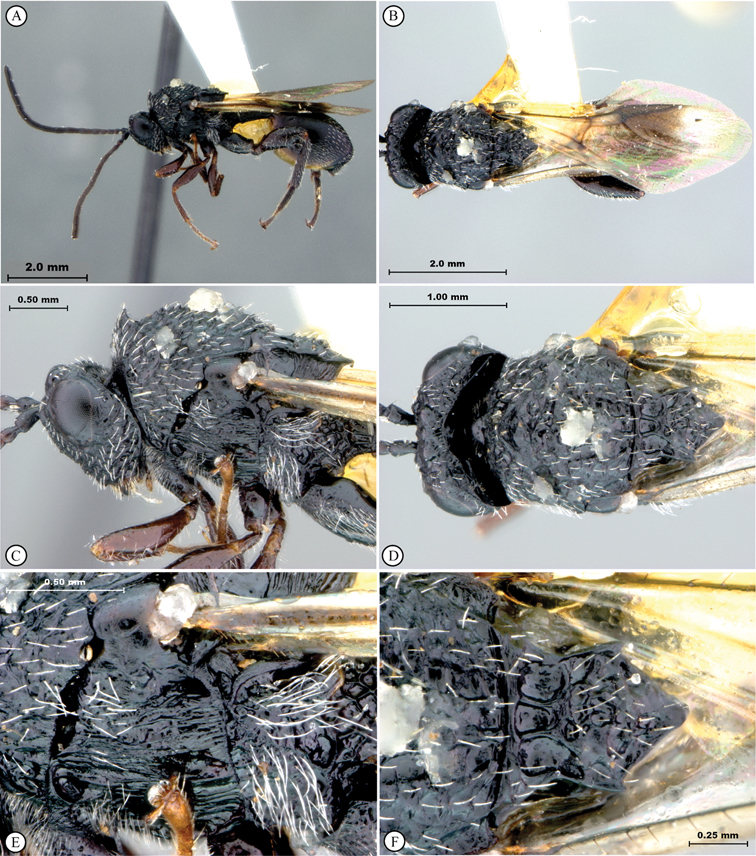
*Xenocynips ronquisti* Buffington & van Noort, sp. n., holotype **A** lateral habitus **B** dorsal habitus **C** head and mesosoma, lateral view **D** head and mesosoma, dorsal view **E** meso- and metapleurae, lateral view **F** scutellum, dorsal view.

**Figure 55.**
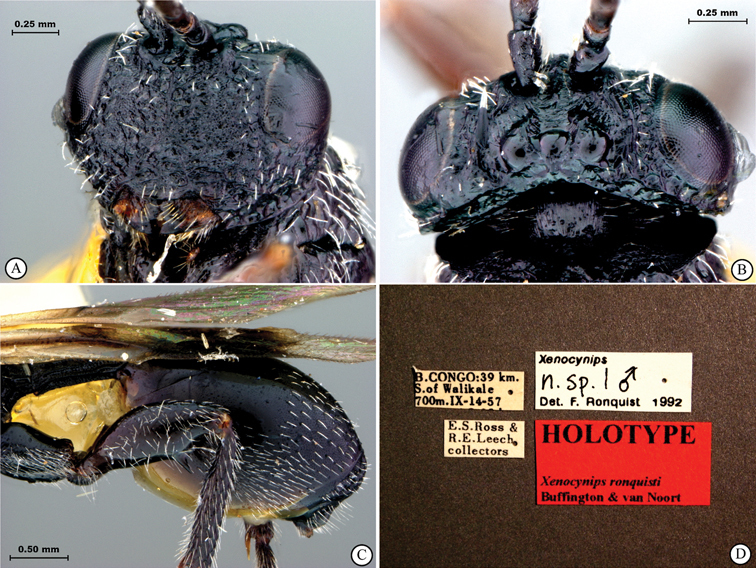
*Xenocynips ronquisti* Buffington & van Noort, sp. n., holotype **A** head, anterior view **B** head, dorsal view **C** metasoma, lateral view **D** labels.

##### Diagnosis.

**Figure 56.**
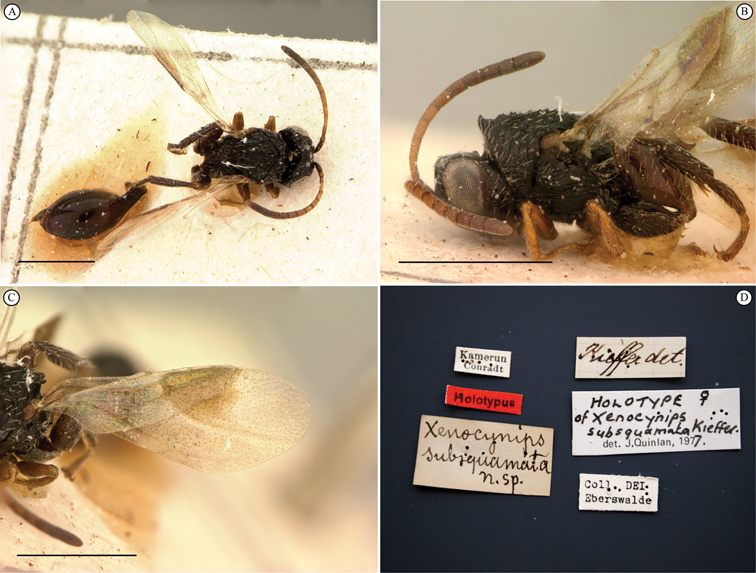
*Xenocynips subsquamata* Kieffer, holotype **A** dorsal habitus **B** head and mesosoma, lateral view **C** fore and hind wings **D** labels.

The entirely striate speculum (Fig. 54C) sets this species apart from other *Xenocynips*, which have a dorsally striate, ventrally smooth speculum (Figs 52C and 56B). Another significant feature is the absence of the propodeal spurs, which both *Xenocynips subsquamata* and *Xenocynips rhothion* both possess.

##### Etymology.

Named in honor of our friend and acclaimed hymenopterist Fredrik Ronquist (Natural History Museum, Stockholm), who determined this represented an undescribed species in his monograph of Liopteridae (Ronquist, 1995).

##### Distribution.

Democratic Republic of Congo. **Link to Distribution Map.** [http://hol.osu.edu/map-full.html?id=300225]

##### Material examined.

Holotype, female: **DEMOCRATIC REPUBLIC OF THE CONGO:** 39km S Walikale, 700m, 14.IX.1957, E. S. Ross and R. E. Leech, USNM ENT 00764784 (deposited in CASC).

#### Xenocynips subsquamata

Kieffer

urn:lsid:biosci.ohio-state.edu:osuc_concepts:181548

Morphbank accession: 704904–704907

http://www.waspweb.org/Cynipoidea/Liopteridae/Oberthuerellinae/Xenocynips/Xenocynips_subsquamata.htm

http://species-id.net/wiki/Xenocynips_subsquamata

Figure 56

Xenocynips subsquamata Kieffer, 1910b: 340.

##### Description.

Coloration of head, mesosoma, and metasoma, dark reddish brown; legs reddish brown. Sculpture on vertex, lateral surface of pronotum and mesoscutum present, deeply striate on head, costate with foveae on pronotum, mesoscutum.

*Head*. Broadly triangular, in anterior view. Pubescence on head present, sparse setae scattered over head. Sculpture along lateral margin of occiput with one costula. Gena (measured from compound eye to posterolateral margin of head) short, ratio of length of gena to length of compound eye in dorsal view < 0.3, in dorsal view. Sculpture of gena present, gently striate. Lateral margin of occiput defined by evenly rounded, raised, sharp carina. Occiput (except extreme lateral margin) with few weak strigae along peripheral margin. Ocelli small, ratio of maximum diameter of a lateral ocellus to shortest distance between lateral ocelli 0.2–0.4. Anterior ocellus close to posterior ocelli, posterior margin of anterior ocellus behind or subcontiguous with a transverse line running through anterior margins of posterior ocelli. Relative position of toruli close to ocelli, ratio of vertical distance between inner margin of torulus and ventral margin of clypeus to vertical distance between anterior ocellus and torulus < 2.0. Median keel of face absent. Vertical carina adjacent to ventral margin of torulus absent. Facial sculpture present, microcoriaceous. Facial impression absent, face flat. Antennal scrobe absent. Vertical delineations on lower face absent. Malar space adjacent to anterior articulation of mandible evenly rounded, striate-foveate. Malar sulcus absent. Compound eye close to posterior ocellus, ratio of distance between compound eye and posterior mandibular articulation to distance between posterior ocellus and compound eye > 1.2. Compound eye, in dorsal view, distinctly protruding from the surface of the head, particularly laterally. Pubescence on compound eye absent. Orbital furrows absent. Lateral frontal carina of face absent. Dorsal aspect of vertex variously strigate; shagreened with faint remnants of carinae. Posterior aspect of vertex foveate. Hair punctures on lateral aspect of vertex absent. Posterior surface of head deeply impressed around postocciput.

*Antenna*. Articulation between flagellomeres in antenna connate with articles broadly joined. Female antenna composed of 11 flagellomeres. Female F1 shorter than F2; black. Flagellomeres of female antenna cylindrical, distinctly widened towards apex, non-clavate. Placoidal sensilla absent. Distal flagellomeres of female antenna not conspicuously enlarged compared to proximal flagellomeres.

*Pronotum*. Macrosculpture on lateral surface of pronotum present, deeply costulate with remnants of foveae. Pubescence on lateral surface of pronotum absent. Anterior flange of pronotal plate distinctly protruding anteriorly, longitudinally striate. Carinae extending posteriorly from lateral margin of pronotal plate distinct but short, not extending to the dorsal margin of pronotum. Lateral pronotal carina present. Pronotal crest present, raised into a distinct process projecting above anterior margin of mesoscutum. Submedian pronotal depressions absent, represented by shallow depression. Lateral margin of pronotal plate defined all the way to dorsal margin of the pronotum. Pronotal plate wide, almost as wide as head.

*Mesoscutum*. Mesoscutal surface convex, evenly curved. Sculpture on mesoscutum present, transversely costate with dorsally projected serrations. Notaulus present, wide, transversely striate, distinctly wider posteriorly. Median mesoscutal carina absent. Anterior admedial lines absent. Median mesoscutal impression absent. Parascutal carina nearly straight anteriorly, posteriorly curved mesally.

*Mesopleuron*. Horizontally strigate dorsally, ventrally smooth with gentle, parallel horizontal striae. Subpleuron transversely striate, glabrous. Lower mesopleuron entirely striate. Epicnemial carina present on ventral half of mesopleuron; shagreened, ventrally bulbous near mesosternum. Lateroventral mesopleural carina present, marking abrupt change of slope of mesopectus. Mesopleural triangle present, gently impressed lacking dorsal and ventral border; deeply striate. Subalar pit large and well defined, lying in posterior end of mesopleural triangle. Speculum present, striate, with distinct smooth, glabrous ventral cavity. Mesopleural carina absent.

*Scutellum*. Dorsal surface of scutellum foveate-areolate. Circumscutellar carina absent. Posterior margin of axillula marked by distinct ledge, axillula distinctly impressed adjacent to ledge. Lateroventral margin of scutellum posterior to auricula smooth ventrally, longitudinally striate dorsally. Dorsoposterior part of scutellum produced posteriorly into blunt spine. Dorsal part of scutellum entirely rugose. Scutellar plate absent. Scutellar foveae present, three, each lateral fovea with two longitudinal divisions, central fovea smooth, resulting in transverse row of 7 longitudinally elongate subfovea. Longitudinal scutellar carinae absent. Single longitudinal carina separating scutellar foveae absent. Posterolateral margin of scutellum drawn out into distinct protuberance. Lateral bar weakly strigate, narrow.

*Metapectal-propodeal complex*. Metapectal cavity anterodorsal to metacoxal base absent. Anterior margin of metapectal-propodeal complex seperated from mesopleuron by distinct dorso-ventral ledge. Posteroventral corner of metapleuron (in lateral view) extended posteriorly. Anterior impression of metepimeron present, triangular, with broadest part ventrally. Posterior margin of metepimeron distinct, separating metepimeron from propodeum. Subalar area slightly broadened anteriorly, without longitudinal division indicated. Calyptra present, blunt, lobe-like, polished posteriorly, strigate anteriorly with setiferous punctures. Dorsellum absent. Anterior impression of metepisternum, immediately beneath anterior end of metapleural carina, absent. Pubescence consisting of few scattered hairs on posterior part of metapleuron and lateral part of propodeum. Propodeal spurs present, striate. Lateral propodeal carinae present, not reaching scutellum. Ventral end of lateral propodeal carina reaching nucha, carinae separated from each other. Inter propodeal carinae space glabrous, costulate. Petiolar rim of uniform width along entire circumference. Petiolar foramen removed from metacoxae, directed posteriorly. Horizontal carina running anteriorly from lateral propodeal carina present. Lateral propodeal carina straight, sub-parallel. Calyptra, in lateral view, elongate. Propodeum ‘neck-like', drawn out posteriorly. Calyptra, in posterior view, dorsoventrally elongate.

*Legs*. Pubescence posterolaterally on metacoxa sparse to moderately dense, confined dense hair patch absent. Microsculpture on hind coxa absent. Longitudinal carina on the posterior surface of metatibia absent. Metafemoral spine present, elongate, extending distally as low keel along ventral femoral margin. Distal mesotibial spurs shorter than medial spurs. Distal metatibial spurs shorter than medial spurs. Ratio of first metatibial segment to remaining 4 segments less than 1.0. Pubescence on outer surface of metatarsal claw sparse, consisting of few setae. Outer surface of metatarsal claw entirely smooth. Apical seta of metatarsal claw positioned on outer surface below dorsal margin. Base of metatarsal claw weakly expanded, apex slightly bent, ratio width of base to length of apex < 0.6.

*Forewing*. Pubescence of forewing absent on basal half of wing, sparse distally. Apical margin of female forewing rounded. Rs+M of forewing tubular. Mesal end of Rs+M vein situated closer to posterior margin of forewing, directed towards posterior end of basalis. Vein R1 tubular along at least basal part of anterior margin of marginal cell. Basal abscissa of R1 (the abscissa between 2r and the forewing margin) of forewing as broad as adjacent wing veins. Coloration of forewing hyaline with slight infuscation covering marginal cell, area posterior to marginal cell. Marginal cell of forewing membranous, similar to other wing cells. Areolet absent. Hair fringe along apical margin of forewing absent.

*Petiole*. Slightly elongate, 1.5–2× longer than wide. Surface of petiole longitudinally costate, ribbed, ventral keel absent. Posterior part of female petiole not abruptly widened. Ventral flange of annulus of female petiole absent. Ventral and lateral parts of petiolar rim narrow.

*Metasoma*. Setal band (hairy ring) at base of tergum 3 absent, base of metasoma glabrous. Tergum 3 indistinct, fused with syntergum. Posterior margin of tergum 3 indistinct, fused with tergum 4 in syntergum. Sternum 3 encompassed by syntergum. Sculpture on metasomal terga present, finely punctate laterally, dorsally; posteriorly with large setal pits. Syntergum present with terga 3–5 fused, ventral margin rounded. Annulus absent. Peg-like setae on T6–T7 absent. Posteroventral cavities of female metasoma T7 present, glabrous save for few, long setae. Female posteroventral margin of T6–T7 straight, parallel, with medial carina. Terebrum and hypopygium (in lateral view) straight, pointing posteriorly.

*Ovipositor*. First valvula of ovipositor narrowing gradually, not broadened apically, smooth at tip. Ovipositor clip absent.

##### Diagnosis.

This species is most easily confused with *Xenocynips rhothion*, but the sculpture of the pronotum is distinct from that species; in *Xenocynips subsquamata*, the pronotum is horizontally costate (Fig. 56B), but the costa do not result in the wave-like crests found in *Xenocynips rhothion* (Fig. 52C). Additionally, the central propodeal keel is entire in *Xenocynips subsquamata*, and forked in *Xenocynips rhothion*.

##### Distribution.

Cameroon. **Link to Distribution Map.** [http://hol.osu.edu/map-full.html?id=181548]

##### Material examined.

Holotype, female: **CAMEROON:** no date, Conradt, USNM ENT 00764783 (deposited in DEIC).

## Discussion

Besides the inferred association with Coleoptera and the rearing of one species, *Paramblynotus yangambicolous*, from a rotten log of Euphorbiaceae (Benoit 1955), and observations suggesting that *Paramblynotus* species are parasitoids of beetle larva (Liu et al. 2007), nothing is known about the biology of the Afrotropical Liopteridae. The extensive posteriorly directed ridges on the pronotum and mesoscutum in a number of species suggest an adaption for exiting from (or burrowing in to find) concealed hosts in a confined substrate such as dense leaf litter or rotten logs. Ronquist (1995) proposed that these structures help with host tunnel negotiation. These effective, posteriorly directed teeth would facilitate the negotiation of such substrates, preventing slippage and promoting forward movement down the tunnels or through the substrate.

**Figure 57.**
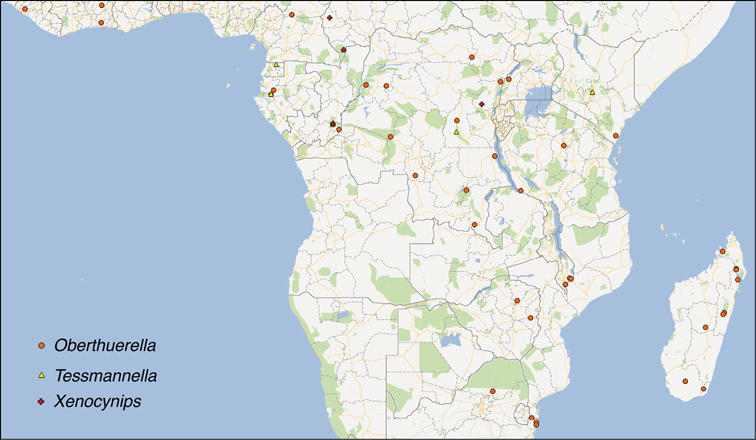
Distribution map of Oberthuerellinae. Circles, *Oberthuerella*; triangles, *Tessmannella*; crosses, *Xenocynips*. All occurrence data are available from Hymenoptera On-line: http://hol.osu.edu/map-large.html?id=125302.

All of the fresh material examined here that was collected by R. Copeland, M. Sharkey, and the second author of this work, was taken in Malaise traps in densely forested areas. Specimens collected by the second author in Central African Republic and Tanzania were taken in yellow pan traps, as well as by fogging *Acacia reficiens* Wawra, *Acacia nilotica* (L.) Delile, and *Cammiphora campestris* Engl. (Fabaceae). To date, Malaise traps, running continuously for extended periods of time in forested areas, are the most cost-effective and efficient way to collect these elusive wasps, but unfortunately adds little to the knowledge of their biology and host associations. The future of Oberthuerellinae research needs to proceed in three directions: the first being additional field work in parts of Africa not yet thoroughly documented (Fig. 57); second, the phylogenetic relationships among these wasps need to be investigated, and prior to this more fresh material needs to be collected; and third, focus on rearing of potential hosts. Far too many oberthuerellines are known only from the type specimen, and none with biological data.

## Supplementary Material

XML Treatment for Oberthuerella

XML Treatment for Oberthuerella abscinda

XML Treatment for Oberthuerella aureopilosa

XML Treatment for Oberthuerella breviscutellaris

XML Treatment for Oberthuerella crassicornis

XML Treatment for Oberthuerella cyclopia

XML Treatment for Oberthuerella eschara

XML Treatment for Oberthuerella kibalensis

XML Treatment for Oberthuerella lenticularis

XML Treatment for Oberthuerella longicaudata

XML Treatment for Oberthuerella longispinosa

XML Treatment for Oberthuerella nigra

XML Treatment for Oberthuerella nigrescens

XML Treatment for Oberthuerella pardolatus

XML Treatment for Oberthuerella sharkeyi

XML Treatment for Oberthuerella simba

XML Treatment for Oberthuerella tibialis

XML Treatment for Oberthuerella transiens

XML Treatment for Oberthuerella triformis

XML Treatment for Tessmannella

XML Treatment for Tessmannella copelandi

XML Treatment for Tessmannella expansa

XML Treatment for Tessmannella kiplingi

XML Treatment for Tessmannella nigra

XML Treatment for Tessmannella roberti

XML Treatment for Tessmannella spinosa

XML Treatment for Xenocynips

XML Treatment for Xenocynips rhothion

XML Treatment for Xenocynips ronquisti

XML Treatment for Xenocynips subsquamata

## Appendix 1

List of morphological terms. (doi: 10.3897/zookeys.202.2136.app) File format: Excel spreadsheet (xls).

**Explanation note:** List of morphological terms, their definitions, and corresponding HAO concepts.
